# HYMET: a hybrid metagenomic pipeline for accurate and efficient taxonomic classification

**DOI:** 10.1093/gigascience/giag024

**Published:** 2026-03-02

**Authors:** Jorge Miguel Silva, Inês Martins, João Rafael Almeida

**Affiliations:** IEETA/DETI, LASI, University of Aveiro, Campus Universitário de Santiago, 3810-193 Aveiro, Portugal; IEETA/DETI, LASI, University of Aveiro, Campus Universitário de Santiago, 3810-193 Aveiro, Portugal; IEETA/DETI, LASI, University of Aveiro, Campus Universitário de Santiago, 3810-193 Aveiro, Portugal

**Keywords:** metagenomics, taxonomic classification, *k*-mer screening, alignment-based methods, computational efficiency, mutation resistance, hybrid pipeline

## Abstract

**Background:**

Reliable taxonomic classification of metagenomic sequences remains constrained by high mutation rates, fragmented assemblies, and large heterogeneous reference databases. HYMET (Hybrid Metagenomic Tool) was developed to overcome these challenges through a 2–stage hybrid design combining adaptive Mash–based screening with Minimap2 alignment and a coverage–weighted Lowest Common Ancestor classifier. Its sample–adaptive thresholds and on–the–fly reference database construction enable efficient, domain–agnostic classification while maintaining accuracy across divergent genomes.

**Results:**

Across 7 CAMI assembly datasets in contig mode, HYMET achieved a mean F1 of 83.89%, with genus–level F1 of 76.75% and species–level F1 of 60.18%, while averaging 115.93 s runtime and a mean peak memory of 6.24 GB. Performance remained stable under mutation rates up to 30% for most domains (F1 $\ge$ 0.8), with viral sequences showing the expected decline (F1 $\approx$ 0.5 at 30%). Read and contig inputs produced nearly identical results when sharing reference caches, and real–world datasets confirmed robustness with the human gut metagenome, which reproduced typical anaerobic profiles, while in the ZymoBIOMICS mock community, HYMET recovered all bacterial members; a further ground-truth evaluation on the ZymoBIOMICS Gut Microbiome Standard (D6331) yielded near-perfect genus-level concordance (Pearson $r = 0.998$, Bray–Curtis $= 0.04$) across bacteria, fungi, and archaea.

**Conclusions:**

HYMET achieves a practical balance of accuracy, efficiency, and scalability for metagenomic classification. Its adaptive candidate selection, alignment–anchored taxonomy, and reproducible reference caching collectively enhance performance across domains. HYMET source code is fully available at https://github.com/ieeta-pt/HYMET.

## Graphical Abstract

**Graphical Abstract:**
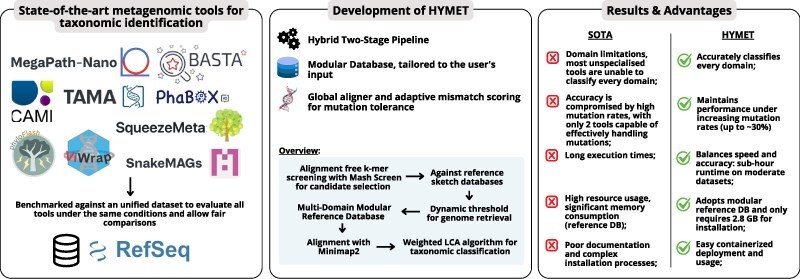

## Introduction

A significant challenge in metagenomics is the development of accurate methods for the taxonomic classification of organisms within a sample [1, 2]. Despite the creation of numerous general-purpose and specialized metagenomic tools, several significant issues persist. Particularly, computational demands pose a major constraint, as tools often require substantial memory and processing power, leading to impractical execution times for large datasets [1, 3, 4]. The sheer volume of metagenomic datasets demands highly efficient algorithms that can operate within reasonable requirements of compute power, which is particularly problematic when dealing with millions of sequencing reads [5–8]. Furthermore, taxonomic assignment remains a critical challenge in metagenomic analysis, especially at lower taxonomic levels [8]. This issue is exacerbated by the limitations of reference databases, which often exhibit significant sampling bias toward well-studied organisms, while underrepresenting species that are difficult to culture in laboratory settings [1, 3]. This discrepancy results in high rates of unclassified or misclassified reads, especially in complex environmental samples [2, 9, 10]. Furthermore, the lack of standardized benchmarking protocols and datasets hinder objective comparisons of tool performance, as researchers frequently test tools on non-uniform datasets with inconsistent evaluation metrics [10–12]. Addressing these issues is crucial for improving our understanding of complex microbial communities and developing efficient, user-friendly software solutions to analyze the enormous amounts of data generated by metagenomic research [1, 3]. These collective challenges directly motivate our core research question:

*How can a next-generation metagenomic classification tool be designed and implemented to accurately identify taxa across all domains while maintaining high performance and efficiency?*

HYMET (Hybrid Metagenomic Tool) was conceived in response to the recurrent bottlenecks observed when processing large and diverse metagenomic datasets, where existing profilers either failed to capture divergent taxa or consumed excessive resources. Its design draws inspiration from practical experience with real microbial communities, where rapid screening and selective reference database construction often proved more effective than relying on static, monolithic databases. HYMET follows this principle by integrating adaptive reference selection with precise alignment and a weighted taxonomic resolver. This hybrid, sample-aware approach enables the tool to dynamically tailor its search space to each dataset, thereby reducing memory usage and execution time while preserving classification accuracy across domains.

Specifically, HYMET introduces 3 main innovations. First, an adaptive Mash Screen step selects candidate references on-the-fly, ensuring that downstream analysis focuses only on the most relevant genomes under a fixed resource budget. Second, the selected references are combined into a temporary, sample-specific database that supports accurate alignment even in the presence of mutations or incomplete references. Third, a coverage-weighted Lowest Common Ancestor (LCA) algorithm integrates the breadth and depth of alignment evidence to improve taxonomic consistency, particularly at lower ranks. Together, these components allow HYMET to outperform static index-based approaches in both speed and precision, especially for complex or previously unseen samples.

## Background

In recent years, we have witnessed remarkable progress in metagenomics, particularly in the development of computational tools for taxonomic classification and functional analysis [1–3]. A dominant trend in current methodologies is the integration of established classification techniques into end-to-end pipelines, which streamline the entire analytical workflow, from raw sequencing data to biologically interpretable results [13]. Currently, the state-of-the-art landscape is populated by a rich ecosystem of interconnected tools, each offering unique capabilities and complementary approaches that collectively advance the field’s analytical power. Among these, SnakeMAGs [14] stands out for its specialized focus on reconstructing prokaryotic genomes from Illumina sequencing reads, while SqueezeMeta [15] offers a fully automated and comprehensive solution for metagenomic data analysis [13, 16]. The first tool uses the Genome Taxonomy Database (GTDB) toolkit [17] for taxonomic assignment, leveraging conserved marker genes for analysis. On the other hand, SqueezeMeta uses DIAMOND [18] for alignment and the LCA algorithm for taxonomic assignment [15].

Complementing these general-purpose pipelines, several lightweight tools have emerged to address specific needs in taxonomic assignment. The Basic Sequence Taxonomy Annotator (BASTA) [19] also employs the LCA algorithm for efficient sequence classification, while the Critical Assessment of Metagenome Interpretation Taxonomy (CAMITAX) [4] improves accuracy through the integration of multiple classification strategies for microbial genome assignment, including genome distance-based classification using Mash [20], Centrifuge [1], and Kaiju [21], that determines the interval-union LCA of gene-level assignments and 16S rRNA gene-based classification employing a naive Bayesian classifier method using Dada2 [22]. For more robust taxonomic profiling, the Taxonomy Analysis by Multiple Assignment (TAMA) tool [23] combines consensus classifications from established classifiers, including Kraken [8], CLARK [24], and Centrifuge, leveraging their complementary strengths.

In addition to these workflows, widely used read-level classifiers include KrakenUniq, Ganon (and ganon2), Centrifuge/Centrifuger, Taxor, and compositional MinHash methods such as sourmash gather [25–30]. These tools are often embedded within workflows (e.g., CAMITAX integrates Centrifuge/Kaiju, and TAMA combines Kraken, CLARK, and Centrifuge). In our CAMI contig benchmark, we therefore include representative stand–alone baselines that accept contig inputs and emit per–contig labels (Kraken 2, Centrifuge, Ganon 2, and sourmash gather) while citing [29] as a state–of–the–art long–read read–level classifier that falls outside this contig–based evaluation.

The field has also seen the development of specialized tools that target specific metagenomic applications. Viral genomics is particularly well served by PhaBOX [31, 32] for viral contig characterization and ViWrap [33] for prediction of viral–host relationship, both providing valuable information on viral diversity and ecological interactions [34–36]. The first tool, PhaBOX, developed by Shang et al., combines gene prediction and alignment (DIAMOND) with taxonomic classification by a semi-supervised learning method (PhaGCN [32]), based on sequence similarities and cluster sharing networks, and final assignments using the LCA. ViWrap, on the other hand, uses machine learning and sequence similarity searches to identify viral sequences and BLAST [37] to identify best hits against databases for taxonomic annotation and host prediction. For the analysis of the microbial community, phyloFlash [38], developed by Gruber-Vodicka et al., offers unique capabilities through its small subunit ribosomal RNA (SSU rRNA)-based approach, enabling both metagenomic profiling and high-resolution phylogenetic studies [39]. In the critical area of antimicrobial resistance surveillance, MegaPath-Nano [40] has emerged as an important tool for the comprehensive detection of resistance genes, directly supporting public health monitoring efforts; it couples hash-based *k*-mer mapping with Minimap2’s seed-chain-extend *local* alignment model [41], thereby covering broad and potentially divergent sequence segments [42, 43]. Related alignment-based profilers adopt a 2-stage design. For example, Metalign [44] first applies CMash to pre-filter the reference by containment and then aligns reads with Minimap2 to produce the profile.

Despite this technological progress, significant challenges impede the broader implementation of metagenomic tools in clinical and research settings. Implementation barriers represent a primary obstacle, with inadequate documentation and complex installation procedures frequently compromising tool accessibility and user adoption [45]. Computational constraints further limit practical application, as excessive memory and storage requirements hinder scalability. This is exemplified by SqueezeMeta, which stages more than 500 GB of on-disk reference data and exhibits prohibitively long processing times [15], and BASTA, whose dependence on BLAST-based alignments creates computational bottlenecks that render it inefficient for time-sensitive analyses [19, 37]. A fundamental limitation stems from reference database dependencies rather than inherent tool restrictions. For instance, TAMA demonstrates robust classification capabilities in principle, but its default bacterial reference database necessarily limits its taxonomic scope to bacterial identification while requiring hundreds of gigabytes of disk space for its bundled indices [23]. Similarly, independent evaluations of MegaPath-Nano confirm its strong performance in the detection of prokaryotic antimicrobial resistance, but note a reduced sensitivity when analyzing higher eukaryotes [40, 42]. This pattern of taxonomic bias is further evidenced in specialized tools such as PhaBOX and ViWrap, which, while excelling in virome analysis, lack versatility for broader metagenomic applications [31, 33]. phyloFlash’s reliance on small subunit rRNA analysis makes it fundamentally unsuitable for viral identification, as viruses lack ribosomal RNA genes [38].

## Materials and methods

### HYMET workflow overview

**Figure 1:**
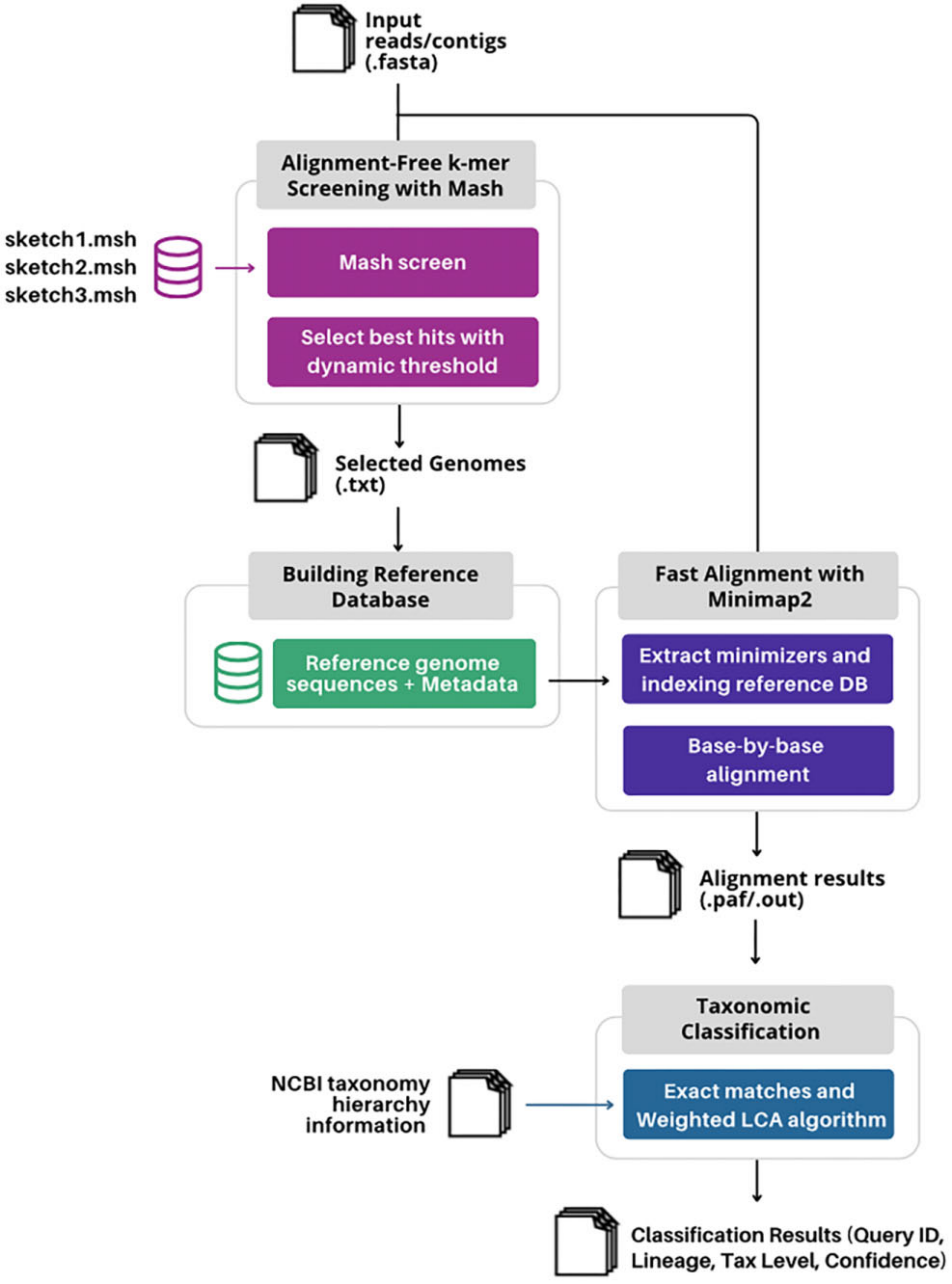
Overview of HYMET architecture.

HYMET, illustrated in Fig. 1, is driven by a unified Python CLI (hymet from Bioconda or bin/hymet in a source checkout) that orchestrates Mash (*k*-mer screening), Minimap2 (alignment), and the weighted–LCA classifier. For reproducible installs, we provide container images (Docker and Apptainer/Singularity) and a Bioconda package (Conda/Mamba). Legacy Perl components are bundled for reproducibility but are not the primary entry point. HYMET is designed for shotgun metagenomes in contig or genome form. It can classify 16S/SSU rRNA sequences when they occur in assembled contigs or shotgun reads, but targeted 16S rRNA amplicon libraries are out of scope and are better analyzed with rRNA–centric profilers (e.g., phyloFlash [38]). Installation and configuration details are provided in Supplementary Sections 1–4.

#### Alignment-free *k*-mer screening

The initial phase of HYMET utilizes Mash Screen [20] for rapid *k*-mer-based screening against pre-computed MinHash reference sketch databases [46–50]. Mash employs containment scores, as defined in Equation (1), to assess the proportion of a reference genome present in a query sequence [51–53]. The containment index $c_k(a, b)$ is estimated as


(1) \begin{eqnarray*}
c_k(a, b) \approx \frac{|S(A) \cap \pi (B)|}{|S(A)|},
\end{eqnarray*}

where $S(A)$ is the sketch of the reference genome *A*, and $\pi (B)$ represents the *k*-mers of the query sequence *B*. The containment index ranges from 0.0 to 1.0, with values closer to 1.0 indicating a higher proportion of *k*-mers from the reference genome present in the query. This metric is crucial for tasks such as contamination screening, reference genome selection, and the discovery of novel genomes, as it provides a rapid and unbiased estimate of sequence representation [46, 47, 49, 51, 54]. In this work, the screening process was optimized for computational efficiency by enabling parallel processing and applying a stringent 90% similarity threshold to retain only high-confidence matches, filtering out low-quality alignments. This containment-based approach prioritizes likely taxonomic candidates, reducing the search space and computational load for subsequent alignment-based stages [20, 46, 52]. As previously mentioned, to enable this screening, Mash relies on sketched databases, which are compact representations of genomic sequences. These databases are built using consistent *k*-mer hashing with MurmurHash3 [20, 47, 55, 56], which allows efficient comparison of query sequences against large collections of reference genomes.

HYMET requires fast per-reference containment ranking on unassembled reads under a fixed memory budget. Mash Screen provides fixed-size MinHash sketches, direct read-set $\rightarrow$ reference containment estimates, and per-hit *P*-values, which integrate cleanly with our adaptive cut-off and candidate budgeting. In contrast, sourmash uses scaled FracMinHash with an iterative gather (set-cover) procedure aimed at mixture decomposition rather than per-reference ranking, and CMash (as in Metalign [44]) focuses on multi-*k* containment estimation, which we do not require here. Consequently, HYMET applies a dynamic, sample–adaptive containment threshold that by default targets ~3.25 candidates per input sequence (rounded; minimum of 5) and enforces a floor threshold of 0.70, reducing the search space while preserving recall. The pre–filter is modular and could be replaced by sourmash or CMash without altering downstream alignment; we chose Mash for its simplicity, stable memory, and native significance testing [30, 44, 57].

##### Sketch and *k*-mer size

The construction of these sketch databases involves 2 key parameters: the *k*-mer size (*k*) and the sketch size (*s*). The choice of *k*-mer size is essential as it balances sensitivity and specificity. Smaller *k*-mers increase sensitivity for divergent genomes but may lead to random collisions, while larger *k*-mers reduce collisions but may miss subtle variations [20, 47, 50, 51, 58]. The optimal *k*-mer size is calculated as


(2) \begin{eqnarray*}
k = \log _{|\Sigma |}\left(\frac{n(1-q)}{q}\right),
\end{eqnarray*}

where $|\Sigma |$ is the alphabet size (4 for nucleotides), *n* is the genome size, and *q* is the desired probability of observing a random *k*-mer. For example, smaller genomes (e.g., viruses) and highly variable taxa require smaller *k*-mer sizes (e.g., *k*-mer $=$15) to ensure specificity, while larger genomes (e.g., vertebrates) benefit from moderate *k*-mer sizes (e.g., *k*-mer $=$21) to balance sensitivity and computational efficiency [53, 58]. The sketch size, which refers to the number of unique min-hashes retained for genomic sequence representation, also plays a critical role in determining the accuracy of distance and containment estimates [20, 50, 52, 58]. The error associated with containment estimation for a given sketch size, *s*, is proportional to


(3) \begin{eqnarray*}
\mathrm{Error} \approx \sqrt{\frac{1}{s}},
\end{eqnarray*}

indicating that larger sketch sizes improve precision, but at the expense of greater computational resources [58]. For instance, smaller or highly fragmented genomes typically require larger sketch sizes to ensure sufficient genomic information is captured, while larger or less fragmented genomes can achieve accurate containment estimates with smaller sketch sizes. This adaptive approach is supported by empirical evidence, with studies demonstrating that a sketch size of *s* = 1,000 is generally adequate for obtaining precise similarity estimates in well-assembled genomes [20]. In fact, Ondov et al. established *s* = 1,000 and *k*= 21 as the default parameters in Mash, as they provide precise similarity estimates for well-assembled genomes [20]. However, for more divergent genomes, increasing the sketch size (e.g., *s* = 5,000) can improve accuracy by capturing a more representative subset of genomic content [50, 53, 58, 59].

##### Reference sketched databases

Following these design principles, we implemented a comprehensive database strategy combining both established public resources and a custom-built collection:

- **RefSeq Nucleotide Release 88:** Pre-built sketch distributed by the Mash project [58], comprising organisms from RefSeq nucleotide release 88, compressed using $k=21$ and *s* = 1,000. This sketch serves as the primary screening database, totaling 1.2 GB.
- **GTDB r202 Assembly Set and NCBI Complete Genomes Database:** Combines 89,675 genomes from GTDB r202 and NCBI RefSeq (viruses, fungi, and bacteria/archaea), compressed with $k=21$ and *s* = 1,000 [56].
- **Custom Reference Database:** Enhances representation of underrepresented taxa by including 19,505 up-to-date genomes from NCBI RefSeq. For smaller genomes (e.g., archaea, fungi, protozoa, viruses), sketches were generated with $k=15$ and *s* = 5,000, while larger genomes (e.g., vertebrates, plants, invertebrates) used the default parameters ($k=21$, *s* = 1,000) [50].

These databases were grouped on the basis of shared hash seed values and parameters to optimize the screening efficiency. Table 1 summarizes their characteristics. All databases are publicly available through our project repository. For reproduction, detailed instructions are provided in Supplementary Material Section 2, subsection “Reproducing Sketched Databases.”

**Table 1:** Reference sketched databases. Different hash seed values reflect the provenance of each sketch set. RefSeq88 sketch from Mash with hash seed 0, GTDB and custom sketches generated locally using default hash seed 42

Sketch	Content	Sketch parameters	Sketch size	Hash seed
sketch1.msh	RefSeq nucleotide release 88	*k* = 21, *s* = 1,000	1.2 GB	0
sketch2.msh	GTDB r202 Assembly Set, NCBI Complete Genomes Database + Custom Databases (vertebrates, plants, invertebrates)	*k* = 21, *s* = 1,000	883.25 MB	42
sketch3.msh	Custom databases (fungi, protozoa, archaea, virus)	*k* = 15, *s* = 5,000	327.93 MB	42

#### Modular reference database download

##### Candidate selection with dynamic threshold

After running Mash Screen, the output can be extensive, potentially including a large number of candidate genomes with varying degrees of similarity to the query sequences. Downloading and analyzing this entire list would be computationally inefficient and could introduce noise into subsequent analyses. On the other hand, setting an arbitrarily high static threshold might exclude important reference genomes, leading to incomplete coverage of the query sequences. To address these challenges, HYMET introduces a dynamic Mash–Screen thresholding mechanism to identify the most relevant candidate genomes and to create a targeted, input–specific database. The algorithm iteratively lowers the containment threshold in 0.02 steps until a minimum candidate count is reached (~3.25 per input sequence, rounded; minimum 5), or until a floor of 0.70 is reached.

##### Genome retrieval

Following the selection of candidate genomes, their format was analyzed to enable efficient mapping and retrieval. These genomes were identified using RefSeq (GCF) and GenBank Assembly Genomes (GCA) accession numbers, unique identifiers assigned by NCBI. Thus, the NCBI Assembly database [60] was selected as the primary resource for constructing the reference database [10, 61]. To optimize the process, summary files from the NCBI Assembly database were downloaded, providing efficient access to metadata. A custom script was developed to map candidate genomes to these files using accession numbers. This script extracted the base accession number (e.g., “000169215”) to ensure compatibility between different assembly versions (e.g., GCF_000169215.1, GCF_000169215.2), preventing retrieval failures due to version updates [60].

During the initial mutation study (conducted with the systematic–review harness), genomes were retrieved from the NCBI FTP service and decompressed locally. In all subsequent benchmarks and case studies in this manuscript, genomes were retrieved over HTTPS from NCBI and decompressed locally. The downloader uses bounded retries with exponential backoff and records failures in the run logs. To enhance efficiency, the script employed ThreadPoolExecutor for parallel downloads, allowing up to 64 concurrent threads. The taxonomic identifiers (TaxIDs) of the assembly files were stored alongside the accession numbers and sequence identifiers, creating a comprehensive reference linking each genome to its taxonomic and sequence-level information [60, 61].

HYMET is designed to operate efficiently in network-constrained or offline environments. Users can supply a local directory containing mirrored NCBI assembly summaries to enable species-level candidate deduplication without requiring internet connectivity. Reference data are cached locally, and users have the option to preload these caches with curated FASTA files along with their corresponding sequence-to-TaxID mappings. Indices are automatically updated during subsequent analyses, ensuring reproducibility and consistency even in offline scenarios. Additional details can be found in “Reference retrieval policy and fallbacks” of the Supplementary Material.

#### Fast alignment

In the second processing stage, HYMET employs Minimap2 for efficient and precise sequence alignment. This choice was motivated by Minimap2’s adaptive scoring system and its seed-chain-extend *local* alignment strategy, which enables accurate mapping even with highly divergent sequences or incomplete reads, making the pipeline particularly resilient to common metagenomic challenges such as mutation-rich or fragmented samples [40, 41]. The pipeline uses minimizers to index reference sequences, enabling the rapid identification of alignment regions [62]. For contig/genome inputs, we use the -x asm10 preset, optimized for genome-to-genome alignment (~10% divergence, or 90% identity) [41, 63]; for read inputs, we use Minimap2’s short-read preset -x sr. The results are saved in a PAF file, providing essential alignment details such as sequence IDs, lengths, positions, and mapping quality [41, 64].

#### Taxonomic assignment

HYMET uses a hybrid taxonomic assignment strategy, combining the LCA algorithm with a weighted approach based on alignment coverage [8, 23, 24]. For exact matches, the reference’s taxonomic lineage is directly assigned with a confidence score of 1.0. On the other hand, for non-exact matches, the weights for each TaxID are calculated according to


(4) \begin{eqnarray*}
\mathrm{Weight} = \mathrm{Coverage} \times \mathrm{Abundance},
\end{eqnarray*}

where $\mathrm{ Coverage}$ is the proportion of the query aligned with the reference, and $\mathrm{ Abundance}$ is the reference’s frequency in the dataset. HYMET then computes a weighted consensus lineage across ranks and reports a single representative TaxID (the taxon with the highest cumulative weight across the supporting alignments). The confidence score is derived as the product of the per–rank consensus fractions.


(5) \begin{eqnarray*}
\text{Confidence Score} = \prod _{i=1}^{n} \text{Confidence at Rank}_i,
\end{eqnarray*}

where *n* is the number of ranks. This ensures higher consistency across ranks results in higher confidence scores. The final output includes the query identifier, a taxonomic lineage (kingdom to strain), the most specific rank, the representative NCBI TaxID, and a confidence score (0.0–1.0), reflecting the reliability of the classification [24, 65, 66]. The tab-delimited file classified_sequences.tsv contains the columns: Query, Lineage, Taxonomic Level, TaxID, and Confidence.

All analyses, including tool evaluation, development, and validation, were conducted on a high-performance Linux-based virtual machine with 2 TB storage and 250 GB RAM. HYMET’s performance was assessed using precision and F1 score metrics for classifying organisms across the 3 domains of life, considering taxonomic levels (kingdom to species) and mutation rates (0–30%). The analysis also examined the relationship between F1 scores, execution time, and resource usage (CPU and memory), ensuring a complete evaluation of precision and efficiency. The same methodology was applied to the other current state-of-the-art tools described in the “Introduction” section to ensure a consistent comparison. Benchmarking was performed by comparing HYMET with general-purpose cross-domain workflows that produce per–read taxonomic assignments. Component–level short–read classifiers (e.g., KrakenUniq, Ganon, Centrifuge/Centrifuger) and compositional search methods (e.g., sourmash gather) were out of scope for head–to–head benchmarking; their behavior is represented through the workflows that include them. Detailed instructions for reproducing the benchmarking of these tools are provided in Supplementary Section 5.

### Test and validation dataset

The test dataset was derived from the NCBI RefSeq Assembly database (last modified: 13 October 2024), chosen for its curated and validated sequences [10, 67, 68]. Assembly summary files for all biological domains and viruses were downloaded, and 10% of the entries were randomly selected based on GCF accession numbers. For each GCF, 10% of its genome sequences were further sampled to ensure proportional representation and mimic the fragmentation of metagenomic data  [69]. This approach resulted in a diverse and representative dataset, as detailed in Table 2. For dataset replication, complete instructions and scripts are provided in the Supplementary Material Section 3, subsection “Replicating the Benchmark Dataset.”

**Table 2:** Composition of the test and validation dataset

Domain/group	Number of GCFs	Size (GB)
Viruses	1,498	0.05
Other vertebrates	43	2.83
Vertebrate mammals	23	2.29
Protozoa	12	0.03
Plants	19	1.02
Invertebrates	43	1.14
Fungi	63	0.15
Bacteria	24,271	7.23
Archaea	231	0.05
Total	26,203	14.76

### CAMI benchmarking design

To assess the performance of HYMET alongside other baseline metagenomic classification tools, we performed standardized benchmarking using the CAMI (Critical Assessment of Metagenome Interpretation) datasets. Specifically, we selected 7 publicly available contig assemblies representing various levels of complexity and ecological contexts. These include the low, medium, and high complexity communities from CAMI I; the mouse gut, marine, and strain madness panels from CAMI II; and the CAMI reference sample_0 (details are provided in Table 3).

**Table 3:** CAMI benchmark samples and inputs used in the benchmark. All evaluations are contig-based and scored against CAMI truth profiles and contig maps

Sample ID	CAMI panel	Complexity/context	Input	Truth assets
cami_i_lc	CAMI I	Low complexity community	Contigs	Profile + contig truth
cami_i_mc	CAMI I	Medium complexity community	Contigs	Profile + contig truth
cami_i_hc	CAMI I	High complexity community	Contigs	Profile + contig truth
cami_ii_mousegut	CAMI II	Mouse gut metagenome	Contigs	Profile + contig truth
cami_ii_marine	CAMI II	Marine metagenome	Contigs	Profile + contig truth
cami_ii_strainmadness	CAMI II	Strain madness (strain variation)	Contigs	Profile + contig truth
cami_sample_0	CAMI reference	Reference assembly	Contigs	Profile + contig truth

We executed all tools strictly in contig mode to maintain consistent conditions across evaluations. To ensure fair comparisons, our benchmarking pipeline standardized input staging, fixed the number of computational threads, and maintained consistent input/output management. Additionally, it captured computational metrics such as wall-clock and CPU time, as well as peak memory usage.

The evaluation methodology adhered closely to established CAMI conventions. We reported metrics across taxonomic ranks from superkingdom down to species level, including both profile-based distances (L1 total variation and Bray–Curtis distance) and precision, recall, and F1-score for presence/absence classification (with a minimum abundance threshold of 0.1%). Contig-level accuracy was determined by directly comparing predicted taxonomic identifiers (TaxIDs) with the CAMI-provided ground truth. Results were summarized individually for each sample and aggregated by taxonomic rank across all 7 datasets.

Tools included in the benchmarking process were HYMET, Kraken 2, Centrifuge, Ganon 2, TAMA, SqueezeMeta, ViWrap, MegaPath-Nano, BASTA, CAMITAX, MetaPhlAn 4, sourmash gather, phyloFlash, SnakeMAGs, and PhaBOX. Each tool was configured using its recommended database or default settings suitable for contig-based inputs. The specific software versions and database sources utilized are fully documented in the supplementary materials.

### HYMET contig vs. read evaluation

To assess how input modality affects HYMET’s performance, each CAMI assembly dataset listed in Table 3 was analyzed under 2 conditions: first, using assembled contigs, and second, using synthetic reads generated from these same datasets. Both modes followed the same general workflow: initial sketch-based screening for candidate references, construction or reuse of reference caches, sequence alignment, and weighted lowest-common-ancestor taxonomic assignment. Conditions such as computational threading, file handling, and benchmarking instrumentation remained consistent between modes. The primary distinction between the 2 analyses involved the alignment parameters and input handling. For the contig mode, HYMET employed alignment settings optimized specifically for longer, genome-to-genome comparisons. In contrast, the read mode processed single-end reads with alignment parameters tailored for shorter, fragmented sequences. Importantly, steps such as candidate selection, reference caching, and taxonomic classification remained unchanged, ensuring comparability across modalities. For each dataset and input modality, the benchmarking framework calculated CAMI performance metrics at all taxonomic ranks, including L1 total variation, Bray–Curtis dissimilarity, precision, recall, and F1 scores. Additionally, wall-clock time and peak memory usage were recorded. Results were first summarized individually per sample, then aggregated as averages across all 7 datasets to enable a balanced comparison of performance between contig and read inputs.

### Case-study design: Gut, Zymo, and ZymoGut

To complement the CAMI benchmarks with real samples, we conducted 3 case studies that reflect common metagenomic contexts: a human gut metagenome assembly from MGnify, the ZymoBIOMICS mock community assembly curated by the Loman Lab, and the ZymoBIOMICS Gut Microbiome Standard (D6331), a manufactured gut mock community comprising 21 strains across 15 species of bacteria, fungi, and archaea. All 3 were processed in contig mode using the same workflow in the “HYMET workflow overview” section, with a fixed number of threads, shared taxonomy inputs, and identical reference caching policy. This ensured that any differences in outcomes arise from sample biology rather than methodological drift.

The Zymo mock community provides a laboratory-defined composition and a canonical set of reference genomes. Ground truth was established at 2 levels: (i) contig-level labels by mapping assembled contigs to the curated Zymo reference panel and assigning each contig to a species TaxID; and (ii) a CAMI-style abundance profile used to compute rank-wise precision, recall, F1, and abundance distances (L1 total variation and Bray–Curtis). The human gut assembly does not have a strict gold standard; evaluation therefore emphasized plausibility of dominant taxa and concordance with public annotations, together with resource measurements.

The ZymoGut D6331 standard provides a more complex, gut-relevant composition with known ground truth, bridging the gap between the descriptive gut evaluation and the rigorous quantitative Zymo mock assessment. Oxford Nanopore SUP basecalls (ERR14251410, MicroBench) were assembled with Flye in metagenome mode (--nano-hq --meta), yielding 535 contigs. Ground truth was established by mapping assembled contigs to the manufacturer’s reference genomes (minimap2, -x asm5); a CAMI-style abundance profile was derived from contig counts.

For all samples, we recorded wall–clock time and peak resident memory for the complete pipeline. Table 4 summarizes the inputs and available truth assets.

**Table 4:** Case-study samples and inputs. The Zymo mock community and ZymoGut standard are evaluated against curated contig-level labels and a CAMI-style profile; the gut assembly is assessed descriptively in the absence of a ground-truth profile

Sample ID	Source	Context	Input	Truth assets
zymo_mc	ZymoBIOMICS mock community (Loman Lab assembly)	Even bacterial/fungal mix	Contigs	Contig labels + CAMI profile
gut_case	MGnify MGYS00006849; SRS9791096; SRR15489027; ERZ24911249; MGYA00794604	Human stool metagenome	Contigs	None (top–taxa comparison)
zymogut	ZymoBIOMICS Gut Microbiome Standard D6331 (MicroBench; Flye assembly of ONT SUP reads, ERR14251410)	Gut mock community (bacteria + fungi + archaea)	Contigs	Contig labels + CAMI profile

In addition to the main Zymo case study, we conducted a targeted reference-ablation protocol to probe robustness to incomplete databases, an increasingly common scenario as public catalogs lag behind newly observed strains. Using the same reference panel employed in the case analysis, we progressively withheld the indexed sequences for each of the 10 constituent organisms (TaxIDs 562, 28901, 1423, 1639, 1351, 1280, 1613, 287, 4932, and 5207) at 0, 25, 50, 75, and 100% removal levels. After each removal step, we rebuilt the reference index and repeated the full HYMET classification pipeline without changing any parameters. For every level, we captured the distribution of contig assignments by taxonomic rank (species or strain, genus, family, and higher) alongside the standard rank-wise profile metrics (precision, recall, F1, L1 total variation, and Bray–Curtis) and runtime measurements. This procedure isolates the impact of reference incompleteness while keeping the sample and analytical settings fixed, thereby reflecting realistic deployments in which key genomes are missing or outdated.

## Results

### Benchmark scope and replicates

We report results over 7 CAMI assembly datasets (Table 3): CAMI I low/medium/high complexity; CAMI II mouse gut, marine, and strain madness; and the CAMI reference sample_0. Each dataset was processed once per tool under fixed threads, yielding 1 run per sample per tool; no technical replicates were used. Rank-wise summaries reflect means across the 7 datasets. The manifest that enumerates these samples is versioned in the repository.

For HYMET’s modality comparison, the same 7 datasets were analyzed twice: (i) contigs as provided and (ii) deterministic synthetic reads created by windowing contigs into 250 bp slices with a 125 bp minimum tail. Aside from input handling and Minimap2 presets, all pipeline stages (candidate selection, cache construction, alignment, and weighted-LCA) and resource controls were identical; again, 1 run per sample per mode (no replicates).

The mutation-sweep experiment spans 9 higher-level groups (viruses, archaea, bacteria, fungi, plants, protozoa, invertebrates, vertebrate mammals, and other vertebrates). For each group, we generated 1 mutated contig set per rate between 0 and 30% using reproducible seeds (substitutions with short indels), then computed precision/recall/F1 by rank from contig-level truth. No technical replicates were used in this sweep.

Real-data evaluations comprise 3 case studies (MGnify gut assembly, ZymoBIOMICS mock community, and ZymoBIOMICS Gut Microbiome Standard D6331), each run once in contig mode using the same workflow as above (“HYMET workflow overview” section).

### CAMI benchmark

All quantitative evaluations in this revision are contig-based. HYMET also accepts read inputs via the unified CLI (Minimap2 sr preset), but a dedicated raw–read benchmark is deferred to a follow–up to avoid mixing modalities. Against contemporary baselines, HYMET maintains high F1 across ranks and taxonomic groups (Supplementary Fig. S4); many competitors lose precision and recall at lower ranks, whereas HYMET preserves balanced performance into genus and species, particularly for microbes and small eukaryotes. Viruses remain the most sensitive to divergence, with modest drops at intermediate ranks, but species–level accuracy remains competitive.

**Figure 2:**
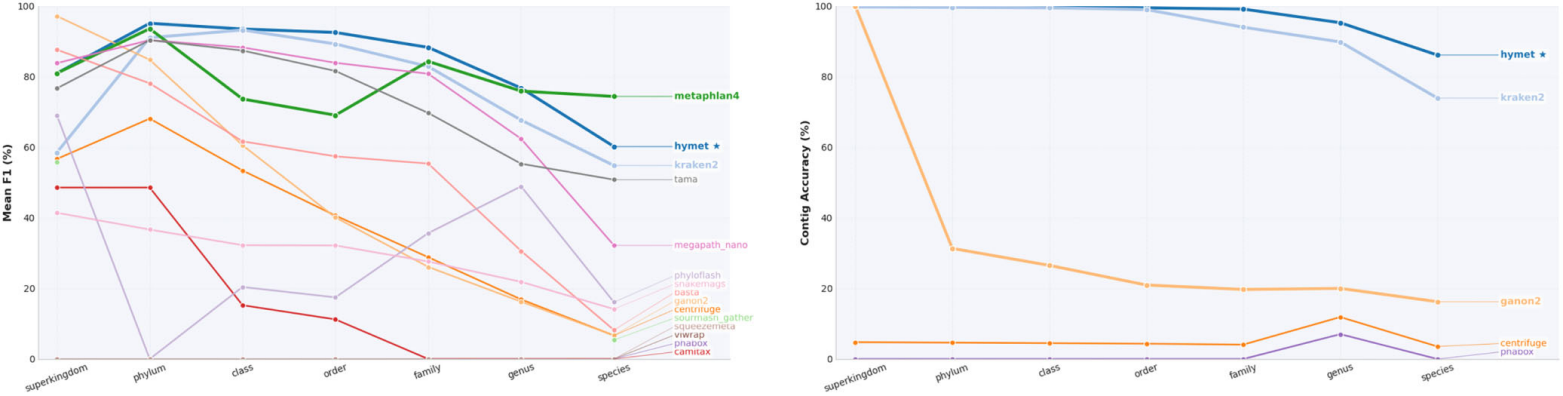
Left: mean F1 by rank (superkingdom to species) across 7 datasets. Right: mean contig-level accuracy by rank.

**Figure 3:**
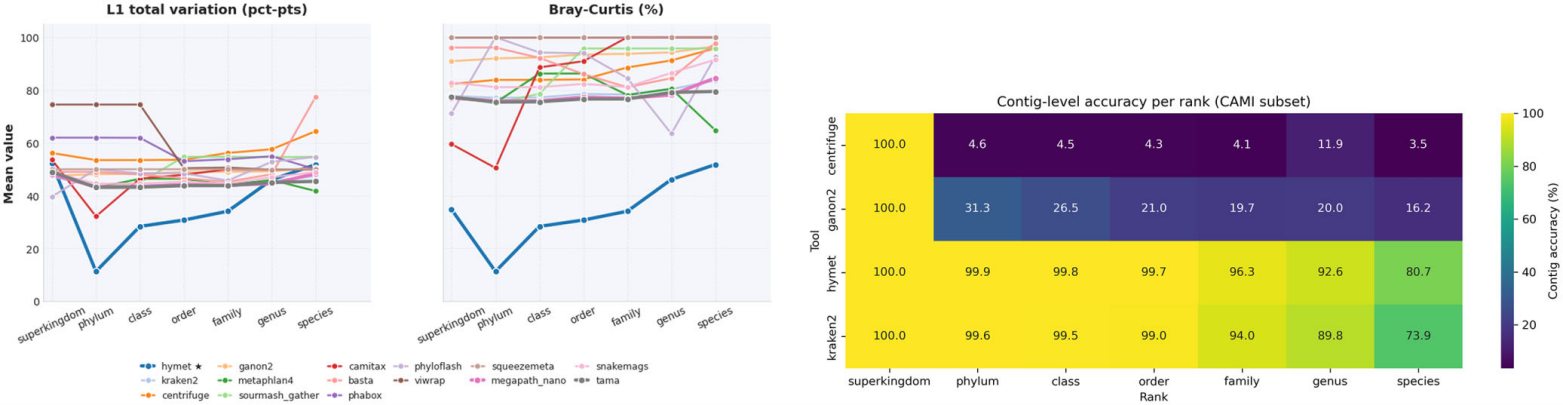
Left: mean L1 total variation and Bray–Curtis dissimilarity by rank across the 7 CAMI assembly datasets. Right: per-rank CAMI accuracy heatmap for the profilers benchmarked.

We benchmarked HYMET and several contig-mode classification tools using diverse CAMI assembly datasets. Figures 2 and 3 summarize the classification performance at different taxonomic ranks across these tools.

Figure 2(top) shows that HYMET consistently outperforms most other tools at various taxonomic ranks, particularly from family up to class. While MetaPhlAn 4 achieves high species-level accuracy, it underperforms at intermediate ranks. Index-based classifiers like Kraken 2 and Centrifuge demonstrate reduced accuracy at lower taxonomic ranks due to lower recall rates. Figure 2(bottom) highlights the stability and consistency of HYMET’s contig-level accuracy across ranks, contrasting sharply with tools relying solely on *k*-mer indices, which exhibit more variability at lower ranks. Only tools that emit per-contig classifications (HYMET, Kraken 2, Centrifuge, Ganon 2, etc.) appear in the contig-accuracy panel because marker-based profilers and read-focused workflows do not produce contig-level outputs.

Profile-distance metrics reinforce HYMET’s advantage. As shown in Fig. 3(top), HYMET attains the lowest Bray–Curtis dissimilarity at all ranks and the lowest or near-lowest L1 at most ranks (with genus, species, and superkingdom showing narrow leads by MegaPath-Nano, MetaPhlAn 4, and phyloFlash, respectively). This indicates tighter abundance estimates than competing profilers overall. The heatmap in Fig. 3(bottom) illustrates how this translates into rank-wise accuracy breadth, particularly below the family level, where index-only tools lose recall. The heatmap is restricted to tools that emit per-contig assignments (HYMET and the index-only classifiers Kraken 2, Centrifuge, and Ganon 2) because marker-based profilers (e.g., MetaPhlAn 4) and pipeline workflows (e.g., TAMA, SqueezeMeta) report profiles only and do not provide per-contig labels.

Table 5 complements these plots: HYMET lowers mean L1 deviation by roughly 9 percentage points relative to Kraken 2 and MetaPhlAn 4 while retaining the strongest average F1. Tools optimized for speed, such as MegaPath-Nano, exhibit higher profile distances despite competitive F1 values, highlighting the trade-off between coarse abundance estimates and precise community reconstruction.

**Table 5:** CAMI profile-distance summary (mean across taxonomic ranks and assemblies). Lower values indicate improved alignment with CAMI truth profiles

Tool	Mean L1 (pct pts)	Mean Bray–Curtis (%)	Mean F1 (%)
HYMET	36.35	33.88	83.89
MetaPhlAn 4	45.21	78.34	78.83
Kraken 2	45.21	78.96	76.76
TAMA	44.76	77.17	73.11
MegaPath-Nano	45.10	77.95	74.53

Table 6 summarizes these performance trends. Overall, HYMET delivered the highest genus-level F1 score (76.75%), a competitive species-level F1 score (60.18%), and the strongest overall average across ranks (83.89%) driven by balanced precision and recall (62.59%/62.00%). MetaPhlAn 4 excelled at species-level accuracy (74.38%, precision 75.46%, recall 77.92%) but lagged at intermediate ranks, yielding a lower overall F1 average (78.83%). Kraken 2 and MegaPath-Nano completed faster but showed reduced sensitivity at lower ranks (species F1: 54.81 and 32.19%; averages: 76.76 and 74.53%) with skewed precision/recall. TAMA balanced precision (79.52%) against higher memory usage (16.88 GB) and obtained an overall average of 73.11%. Together with Fig. 2, these results show that HYMET’s hybrid design sustains recall into lower ranks without sacrificing precision or inflating resource costs. Per-dataset breakdowns are provided in Supplementary Table S18.

**Table 6:** CAMI summary: mean F1 (%) at genus, species, and across all ranks (Avg F1), mean precision/recall at species, mean wall time (s), and mean peak memory (GB) across 7 datasets (canonical multi-tool suite; tight candidate cap)

Tool	Genus F1	Species F1	Avg F1	Species precision	Species recall	Wall time	Peak GB
HYMET	76.75	60.18	83.89	62.59	62.00	115.93	6.24
MetaPhlAn 4	75.90	74.38	78.83	75.46	77.92	146.54	18.76
Kraken 2	67.68	54.81	76.76	69.40	47.19	39.86	10.95
TAMA	55.31	50.81	73.11	79.52	40.14	55.61	16.88
MegaPath-Nano	62.38	32.19	74.53	45.63	25.54	24.11	10.20

### Computational efficiency

**Figure 4:**
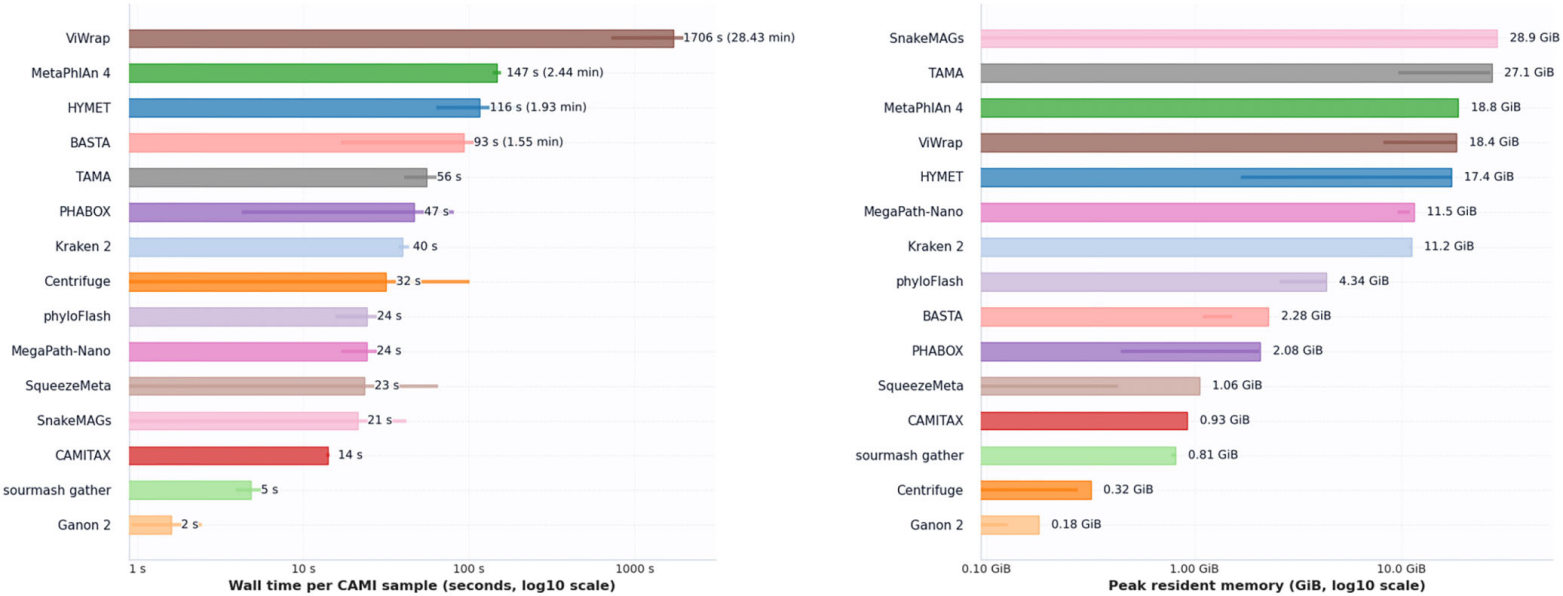
CAMI resource envelopes. Left: aggregated wall-clock time per tool. Right: peak resident memory per tool. Both across the 7 datasets (canonical multi-tool suite; tight candidate cap).

We report resource usage for 2 CAMI suites that share the same datasets but differ in the candidate-reference budget. In the canonical multi-tool suite (tight candidate cap with species deduplication), HYMET builds smaller caches and averages ~116 s wall clock and 6.2 GB peak resident memory across the 7 datasets (Fig. 4; Table 6). Baselines in this suite illustrate distinct speed–memory–accuracy trade-offs (e.g., MetaPhlAn 4:  147 s,  18.8 GB; Kraken 2:  40 s,  11.0 GB; MegaPath-Nano:  24 s,  10.2 GB; TAMA:  56 s,  16.9 GB).

To isolate input-modality effects, the HYMET-only reads-vs.-contigs suite deliberately expands the candidate budget so both modes reuse the same, larger cache. Under this regime, HYMET’s contig runs average 361 s and 17.37 GB peak memory, while the synthetic-read runs average 334 s and 17.36 GB (Supplementary Fig. S7; Table 7). The near-identical memory stems from the shared cache; the modest runtime delta arises primarily from Minimap2 presets (asm10 for contigs vs. sr for reads), not from differences in the search space.

**Table 7:** HYMET contig vs. read comparison across 7 CAMI assembly datasets: mean precision/recall/F1, mean L1 total variation (percentage points), Bray–Curtis (%), wall-clock time (s), and peak resident memory (GB) (HYMET-only suite; expanded candidate budget)

Mode	Precision	Recall	F1	L1	Bray–Curtis	Wall time	Peak GB
HYMET (contigs)	82.15	89.90	84.41	34.55	32.09	361.19	17.37
HYMET (reads)	80.05	90.57	83.37	28.63	26.49	333.61	17.36

The case studies align with the expanded-budget envelope. The MGnify gut assembly and the ZymoBIOMICS mock community each completed in  4–5 min with  17.4 GB peak memory on the reference machine (Table 10), consistent with the reads-vs.-contigs suite and illustrating predictable behavior on real assemblies. In practice, users can choose between these profiles by selecting a tighter candidate cap (minutes-scale, lower memory) or an expanded budget (longer runs with larger caches that can improve sensitivity). Install footprint remains 2.82 GB; dynamically downloaded references typically add 10–50 GB (disk-footprint details are provided in the Supplementary Material).

### Mutation resilience

**Figure 5:**
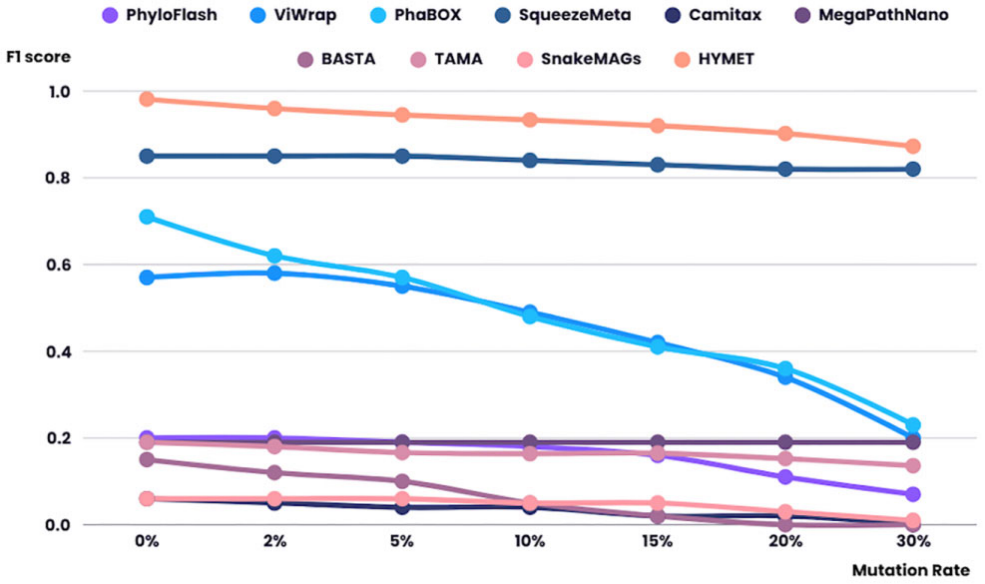
Performance of the state-of-the-art tools and HYMET as the mutation rate increases. The *x*-axis represents the mutation rate (ranging from 0 to 30%), while the *y*-axis shows the F1 score. Each curve on the graph corresponds to a different tool.

Beyond classification accuracy on unmodified references, robustness to sequence divergence is relevant for real-world metagenomes. HYMET’s performance proves exceptionally stable under varying mutation rates (0–30%), outperforming all benchmarked tools in both accuracy and consistency (Fig. 5). Viral classification shows a progressive decline at extreme mutations (F1 scores ~0.5 at 30%), while archaea, invertebrates, and fungi maintain F1 scores over 0.9. Other groups show only minor, non-significant reductions, staying above 0.8 (Supplementary Fig. S5). This contrasts with competing tools, where their scores decline as the mutation rate increases (Supplementary Figs S1 and S2).

### HYMET read vs. contig modes

Supplementary Figs S6 and S7 provide a detailed comparison between HYMET’s contig-based workflow and its synthetic-read workflow across CAMI assembly datasets, highlighting differences in rank-wise performance and computational resource usage. Aggregated metrics that support these visual comparisons are summarized comprehensively in Table 7.

As illustrated in Supplementary Fig. S6, both workflows exhibit closely matched F1 scores across taxonomic ranks. However, the synthetic-read approach demonstrates modestly improved abundance distance metrics at intermediate and lower ranks, as evidenced by lower L1 total variation and Bray–Curtis dissimilarity scores.

The resource usage depicted in Supplementary Fig. S7 confirms negligible differences between the 2 workflows in terms of wall-clock time and memory consumption. This similarity arises because both modes employ identical downstream reference database construction, alignment strategies, and classification methodologies.

Table 7 quantifies these observations, showing that while the synthetic-read workflow achieves comparable recall and overall F1 scores relative to the contig-based workflow (F1: 83.37 vs. 84.41), it provides slightly improved abundance distances (L1: 28.63 vs. 34.55; Bray–Curtis: 26.49 vs. 32.09). Furthermore, both workflows demonstrate similar resource efficiency in terms of execution time (333.61 vs. 361.19 s) and memory usage (17.36 vs. 17.37 GB), illustrating the robustness of HYMET’s pipeline across different input modalities.

### Gut, Zymo, and ZymoGut case-study results

**Figure 6:**
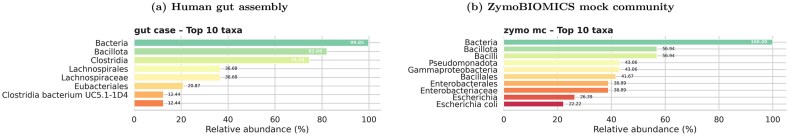
Top-taxa profiles for 2 of the 3 real-world case studies. (a) Human gut assembly: most abundant ranks from superkingdom through species. (b) ZymoBIOMICS mock community: most abundant ranks from superkingdom through species. The ZymoGut D6331 case study is presented separately in Fig. 7.

To extend the evaluation beyond CAMI benchmarks, we analyzed 3 real-world metagenomic datasets: the human gut assembly from MGnify, the ZymoBIOMICS mock community from the Loman Lab, and the ZymoBIOMICS Gut Microbiome Standard (D6331). Figure 6 illustrates the primary taxonomic profiles for the gut and Zymo samples; detailed abundance heatmaps are provided in Supplementary Figs S8 and S9. Computational resource usage for all 3 analyses is summarized in Table 10.

At the contig level, HYMET assigned species/strain labels to 82.82% of gut contigs; in the Zymo mock, HYMET produced species-level labels for 75.00% of predicted contigs (54/72), and among truth-matched contigs (*n* = 61), exactly matched the curated species for 44.26% (27/61; 56.25% of species-assigned), consistent with genus-level substitutions.

In the human gut sample, HYMET identified a microbiome predominantly composed of Bacillota (Firmicutes; 82.09%), followed by significant contributions from Pseudomonadota (9.59%) and Actinomycetota (8.17%) at the phylum level. At the class level, Clostridia strongly dominated (74.54%), particularly represented by the orders Lachnospirales (36.68%) and Eubacteriales (20.87%), with Lachnospiraceae notably prevalent at the family level. These findings reflect typical adult gut microbiomes characterized by obligate anaerobic bacteria. Among the identified species, the most abundant were *Clostridia bacterium UC5.1-1D4* (12.44%), *[Clostridium] scindens* (9.49%), *Coprococcus phoceensis* (6.85%), *Longicatena caecimuris* (6.53%), *Roseburia intestinalis* (6.21%), and *Ruthenibacterium lactatiformans* (5.61%). Additionally, lower-level signals such as *Escherichia* sp. *KTE172* (5.47%) were also detected (Fig. 6a). The gut community heatmap highlighted dense abundance clusters within the Lachnospiraceae and Oscillospiraceae families, reinforcing these results (Supplementary Fig. S8).

The Zymo mock community comprises 10 known organisms, including 8 bacterial species and 2 yeasts (*Saccharomyces cerevisiae* and *Cryptococcus neoformans*). Although yeasts represent 46.80% of the truth profile, in this run HYMET did not recover the eukaryotic component, and the reported profile is restricted to bacterial taxa (53.20%). Within bacteria, HYMET accurately identified major species such as *Escherichia coli* (22.22%), *Listeria monocytogenes* (13.89%), and *Salmonella enterica* (12.50%). Minor genus-level substitutions occurred: *Bacillus spizizenii* in place of *B. subtilis*, and *Pseudomonas* sp. in place of *P. aeruginosa*. Table 8 compares the bacterial abundances (renormalized within bacteria) to HYMET’s predictions, showing 6 exact species matches and 2 genus-level substitutions. The Zymo abundance patterns concentrate within Enterobacterales and Bacillales, consistent with Fig. 6b and Supplementary Fig. S9.

**Table 8:** Zymo bacterial species: truth (renormalized within bacteria) vs. HYMET profile. Notes indicate genus-level matches where the exact species differs

Species	Within-bacteria (%)		Note
	Truth	HYMET	
*Escherichia coli*	18.15	22.22	Exact
*Salmonella enterica*	21.20	12.50	Exact
*Bacillus subtilis*	16.33	15.28	Genus match (*B. spizizenii*)
*Listeria monocytogenes*	12.12	13.89	Exact
*Enterococcus faecalis*	11.54	8.33	Exact
*Staphylococcus aureus*	11.23	9.72	Exact
*Limosilactobacillus fermentum*	8.23	6.94	Exact
*Pseudomonas aeruginosa*	1.20	4.17	Genus match (*Pseudomonas* sp.)

**Figure 7:**
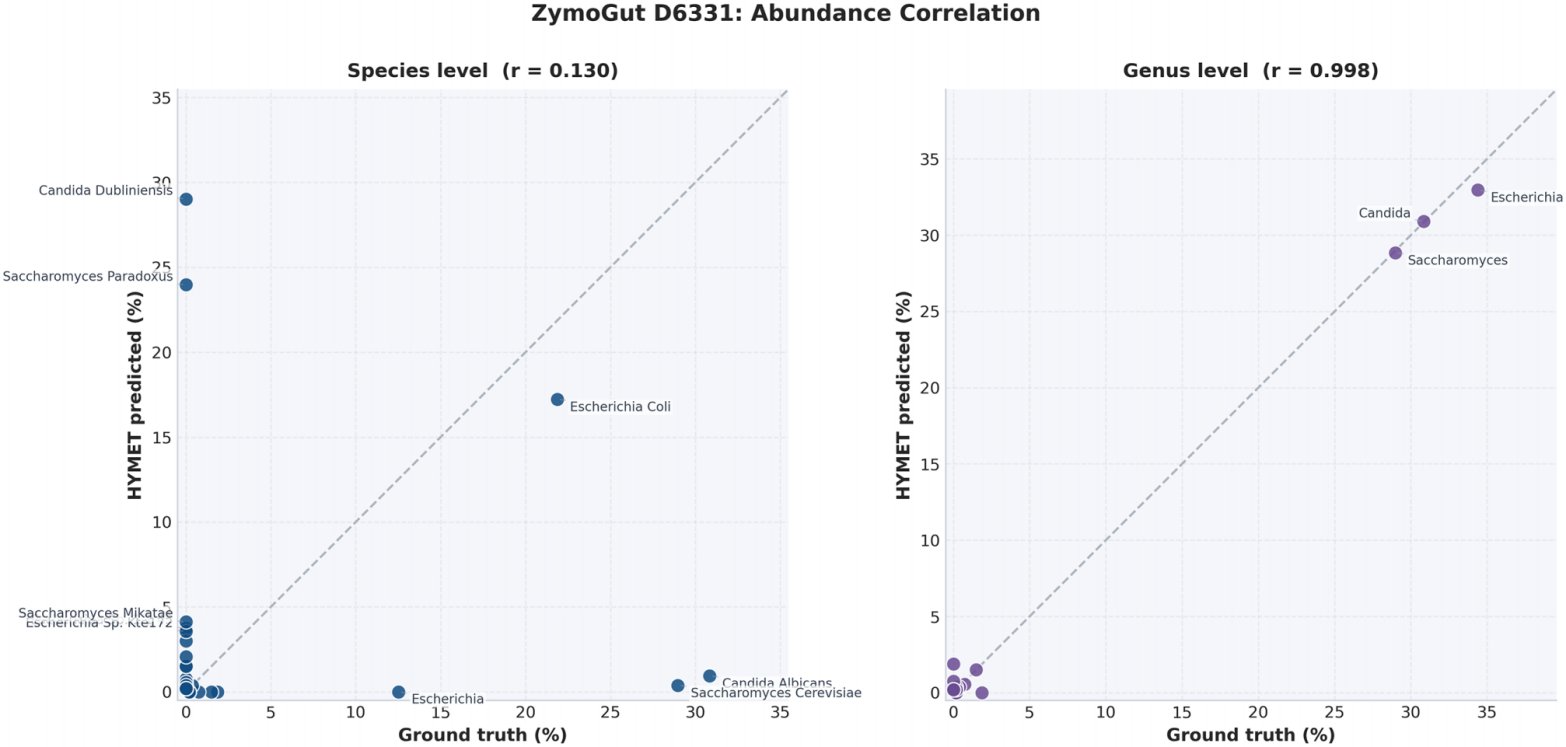
Abundance correlation between ground truth and HYMET predictions for the ZymoGut D6331 mock community. Left: species-level ($r = 0.130$), showing sister-species confusion (e.g., *C. albicans* misclassified as *C. dubliniensis*). Right: genus-level ($r = 0.998$), where all major genera fall on the identity line. Dashed line indicates perfect agreement.

The ZymoGut D6331 assembly (535 contigs, 534 classified) was evaluated against contig-derived ground truth spanning 15 species from 3 domains of life. At the genus level, HYMET achieved a Pearson correlation of $r = 0.998$ and a Bray–Curtis dissimilarity of 0.04 (L1 total variation $= 0.08$), indicating near-perfect abundance estimation. Of 14 expected genera, 11 were correctly detected (78.6% recall). The 3 dominant genera, *Escherichia* (34.4%), *Candida* (30.8%), and *Saccharomyces* (29.0%), were classified within 1.5 percentage points of the truth. The 3 undetected genera (*Clostridioides, Lactobacillus*, and *Roseburia*) were each represented by $\le$1 contig in the Flye assembly, making their detection stochastic at the assembly level. Notably, HYMET correctly identified taxa from all 3 domains (bacteria, eukaryota, archaea), including *Methanobrevibacter* at 1.5% abundance (Table 9, Fig. 7).

**Table 9:** ZymoGut D6331 genus-level comparison: ground truth (contig-derived) vs. HYMET predictions. Diff is predicted minus truth in percentage points

Genus	Truth (%)	HYMET (%)	Diff (pp)
*Escherichia*	34.39	32.96	–1.43
*Candida*	30.84	30.90	+0.06
*Saccharomyces*	28.97	28.84	–0.13
*Clostridioides*	1.87	0.00	–1.87
*Methanobrevibacter*	1.50	1.50	+0.00
*Prevotella*	0.75	0.56	–0.19
*Bifidobacterium*	0.37	0.37	+0.00
*Akkermansia*	0.19	0.19	+0.00
*Bacteroides*	0.19	0.19	+0.00
*Faecalibacterium*	0.19	0.19	+0.00
*Fusobacterium*	0.19	0.37	+0.19
*Veillonella*	0.19	0.19	+0.00

Species-level accuracy was lower ($r = 0.130$), driven by misassignment between closely related sister species within the same genus. *Candida albicans* contigs (165 in truth) were predominantly classified as *C. dubliniensis* (155 contigs), and *S. cerevisiae* contigs (155 in truth) were assigned to *S. paradoxus* (128 contigs). This sister-species confusion is a well-documented limitation across metagenomics classifiers for organisms sharing $>$95% average nucleotide identity and does not affect genus-level assignments: zero contigs were assigned to an incorrect genus (Supplementary Fig. S10).

Computationally, all 3 datasets exhibited similar performance metrics. HYMET completed the analyses efficiently in ~4–5 min each, with peak memory usage consistently around 17.4 GB, showcasing the robustness and scalability of the method across diverse metagenomic contexts (Table 10).

**Table 10:** Case-study runtime and memory summary on the reference machine

Sample	Wall time (s)	Peak RSS (GB)
gut_case	258.32	17.41
zymo_mc	255.73	17.42

**Table 11:** Zymo ablation summary. Levels indicate the ablation proportion; totals are the number of contigs classified

Level (%)	Total classified	Species/strain (%)	Higher/unknown (%)	Genus F1 (%)	Species F1 (%)
0	72	75.00	25.00	88.89	57.14
25	69	72.46	27.54	88.89	57.14
50	71	73.24	26.76	88.89	43.48
75	66	66.67	33.33	73.68	13.79
100	62	64.52	35.48	58.82	0.00

## Zymo ablation results

To assess robustness to incomplete references, we ran an ablation study on the Zymo dataset using the canonical suite, progressively removing species/strain-level entries and re-indexing at each level. Across ablation levels, runtime and memory remained stable ($\sim$4.2–4.5 min, $\sim$17.4 GB). Classification quality degrades as expected at finer ranks: the share of species/strain assignments falls from 75.00 to 64.52%, while higher/unknown rises from 25.00 to 35.48%; genus F1 drops from 88.89 to 58.82% and species F1 from 57.14 to 0.00% at full ablation (Table 11).

**Figure 8:**
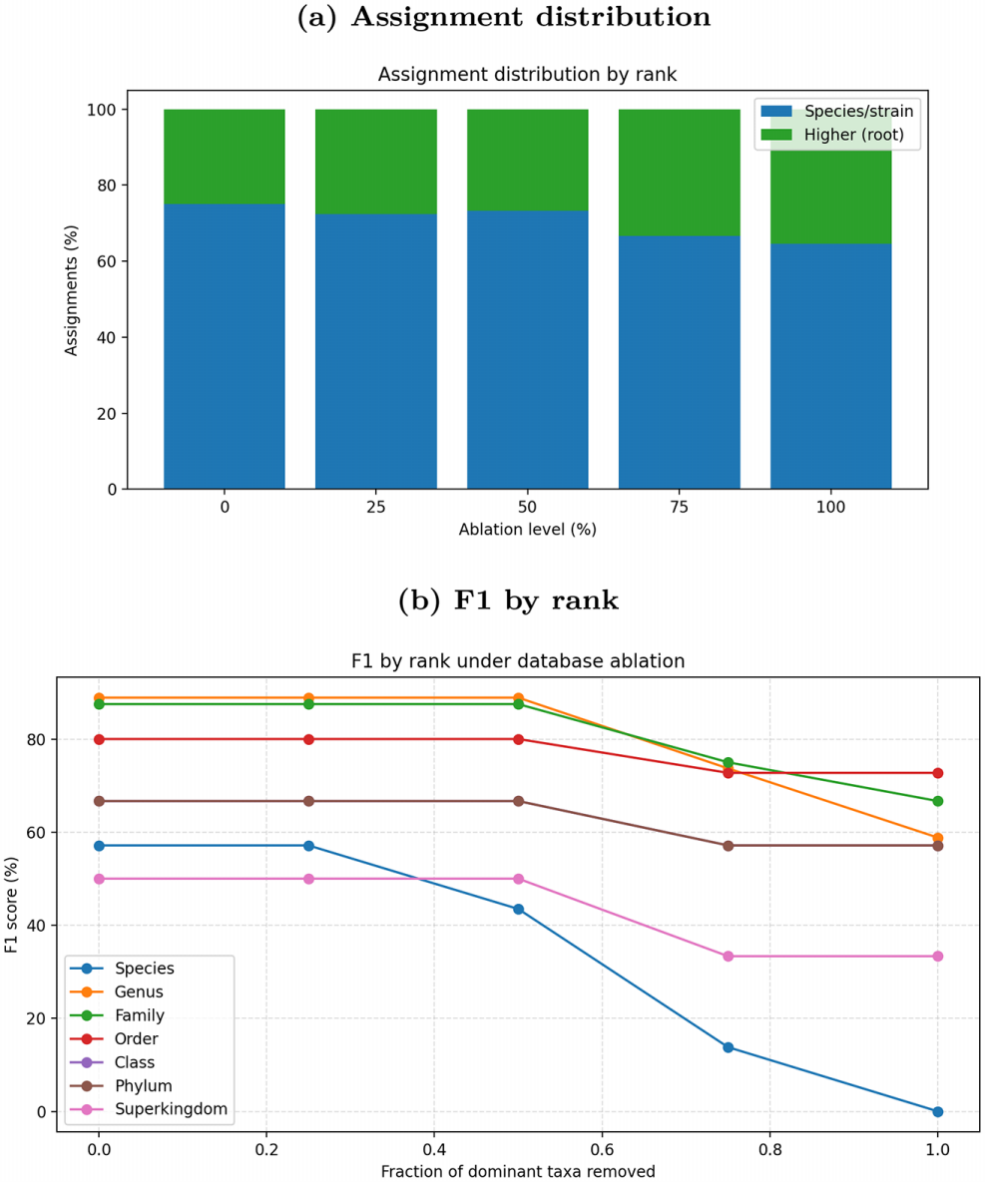
Zymo ablation experiment. (a) Distribution of contig assignments by rank group across ablation levels; species/strain share decreases, while higher/unknown rises. (b) F1 by taxonomic rank under progressive reference removal (0–100%).

Figure 8a shows a gradual reweighting from species/strain toward higher ranks as references are withheld: species/strain drops from 75.00 to 64.52%, while higher/unknown rises from 25.00 to 35.48%; the number of classified contigs declines modestly (72 $\rightarrow$ 62). Genus and family contributions remain stable through 50% ablation and only drift at $\ge$75%, indicating HYMET backs off to appropriate higher ranks rather than emitting incorrect fine-grained labels when exact references are missing.

Rank-wise accuracy (Fig. 8b) remains flat at coarse levels, while genus F1 stays high through 50% (88.89%) and then declines at 75% (73.68%) and 100% (58.82%). Species F1 degrades from 57.14 to 0.00% under full ablation, reflecting the intended removal of discriminative references. Throughout, runtime and memory are effectively unchanged ($\sim$4.2–4.5 min; $\sim$17.4 GB), isolating reference incompleteness as the driver of accuracy loss rather than compute differences.

## Discussion

HYMET effectively addresses significant challenges in metagenomic analysis by integrating adaptive MinHash-based pre-filtering with precise alignment and a coverage-weighted LCA classification. This hybrid design builds upon previous approaches such as Metalign’s combination of CMash and Minimap2, yet it uniquely introduces an adaptive candidate selection method, dynamically generated cross-domain reference databases, and an evidence-weighted LCA step. Compared to fixed-threshold methods, such as CAMITAX, HYMET’s adaptive strategy enhances sensitivity to divergent organisms, avoiding the exclusion of potentially relevant candidates and ensuring broader domain applicability [4, 70].

Our evaluations across diverse CAMI benchmarks demonstrate HYMET’s robust accuracy, computational efficiency, and stable performance across taxonomic ranks. Specifically, HYMET achieved a mean F1 of 83.89% across ranks, a strong genus-level F1 of 76.75%, and a competitive species-level F1 of 60.18%, while maintaining low computational resource demands (average runtime of 116 s and a mean peak memory of ~6.2 GB across samples, ranging from under 2 GB for low-complexity panels to ~17 GB for the most diverse communities). Unlike marker-based tools such as MetaPhlAn 4, which excel at species-level assignments but sacrifice intermediate-rank accuracy and per-contig labeling capabilities, HYMET balances precision and recall consistently across multiple ranks, which is crucial for downstream metagenomic workflows such as genome binning [15, 23, 71].

HYMET’s adaptive cache management strategy allows predictable and flexible resource usage. Both contig and synthetic-read analyses converged in memory usage when sharing reference caches, differing slightly only in runtime due to alignment parameter presets. Practically, users can thus optimize for rapid exploratory analyses or deeper comparative studies across related samples by adjusting candidate cache parameters without compromising accuracy.

Importantly, HYMET demonstrates resilience against genetic mutations, maintaining robust performance (F1 $\ge$ 0.8) for diverse taxonomic groups even at mutation rates up to 30%. While viral sequences experienced a notable decrease in accuracy (F1 $\approx$ 0.5 at 30%), this likely reflects genuine biological and database limitations rather than methodological shortcomings. The stability in performance arises primarily from the optimized choice of screening parameters (smaller *k*-mers and larger sketch sizes for divergent genomes) and the robust seed-chain-extend alignment method employed by Minimap2 [20, 41].

The ZymoBIOMICS case study provided additional insight into HYMET’s limitations. While bacterial taxa were accurately classified (6 exact matches, 2 genus-level substitutions), the method failed to recover yeast species, which represented a significant proportion of the mock community. The systematic ablation experiment further clarified this behavior, showing HYMET appropriately adjusts assignments to higher taxonomic ranks when species-level references are incomplete or missing. This indicates that future versions could benefit significantly from domain-specific adjustments to candidate thresholds, particularly enhancing sensitivity toward underrepresented groups like yeasts. The ZymoGut D6331 evaluation reinforced these findings on a more complex, gut-relevant community: genus-level classification was near-perfect ($r = 0.998$, Bray–Curtis $= 0.04$) across bacteria, fungi, and archaea, while species-level accuracy was limited by sister-species confusion between organisms sharing $>$95% nucleotide identity (*C. albicans*/*C. dubliniensis, S. cerevisiae*/*S. paradoxus*), a well-documented challenge across state-of-the-art classifiers.

A notable operational challenge identified was related to reference database completeness and retrieval reliability. The hybrid reference databases, combining older public sketches with newly generated local sketches, exhibited a non-negligible retrieval failure rate (6.04%), significantly impacting classification accuracy, particularly at lower taxonomic levels. This emphasizes the importance of maintaining continuously updated, comprehensive databases, systematically refreshing manifests, and implementing robust checksum and fallback protocols to mitigate data retrieval issues [17, 67].

HYMET demonstrated robust accuracy across bacteria, archaea, fungi, and small eukaryotes, emphasizing its suitability across diverse microbial communities. However, challenges remain in classifying more complex eukaryotic organisms due to inherent genome complexity, substantial intra-species diversity, and biases toward well-studied taxa in reference databases [72]. Similarly, the polyphyletic and rapidly evolving nature of viral genomes underscores the importance of regularly updated, comprehensive databases to maintain accurate viral taxonomic classification [73, 74].

Future improvements should focus on directly addressing identified limitations: enhancing domain-specific candidate thresholds (particularly for viruses and eukaryotes), implementing scheduled updates of sketch databases enriched with underrepresented taxa, refining the weighted LCA with explicit rules for resolving multi-mapped alignments and establishing minimum evidence thresholds. Integrating uncertainty quantification methods, such as bootstrapping or replicate analyses, will further enhance the reliability and practical applicability of HYMET.

## Conclusion

HYMET advances metagenomic classification by integrating adaptive MinHash screening, precise Minimap2 alignment, and a coverage-weighted LCA algorithm. Evaluations across diverse CAMI benchmarks demonstrate that HYMET achieves robust accuracy, consistently outperforming or matching existing methods at multiple taxonomic ranks, notably with an average F1 score of 83.89%, including 76.75% at the genus level and 60.18% at the species level. This high performance is maintained even under substantial genetic divergence, indicating strong mutation resilience.

Real-world validations further confirm HYMET’s practical value, successfully identifying expected bacterial communities from human gut and ZymoBIOMICS mock samples, and achieving near-perfect genus-level accuracy on the ZymoGut D6331 gut mock community. Computationally efficient and lightweight, HYMET’s adaptive and dynamic caching strategy ensures reproducible and resource-predictable analyses suitable for diverse deployment contexts.

While current results illustrate excellent overall accuracy and scalability, future enhancements could include domain-specific reference optimizations, explicit handling of multi-mapping ambiguities, and integration of machine learning classifiers to further improve lower-rank discrimination. Such developments would position HYMET as a versatile platform, supporting reliable, efficient, and scalable metagenomic investigations across various biological contexts.

## Availability of source code and requirements

- Project name: HYMET (Hybrid Metagenomic Tool).
- Project home page: https://github.com/ieeta-pt/HYMET.
- bio.tools ID: hymet.
- RRID: SCR_026916.
- Operating system(s): Linux.
- Programming language: Python (primary), Perl (legacy), Bash.
- Other requirements: Docker or Apptainer/Singularity; Conda/Mamba.
- License: MIT.

## Additional files

**Supplementary Table S1**: On-disk resource footprint (install + references) for every benchmarked tool in the canonical CAMI environment.

**Supplementary Table S2**: Provenance and composition of the reference databases used by each tool, including shared corpora and official releases.

**Supplementary Tables S3–S10**: Per-domain benchmarking tables (viruses, archaea, bacteria, fungi, protozoa, plants, invertebrates, and vertebrates) reporting precision, recall, and F1 across taxonomic ranks at 0% mutation.

**Supplementary Tables S11–S17**: HYMET-only benchmarking tables summarizing precision, recall, and F1 across taxonomic ranks under mutation rates from 0 to 30%.

**Supplementary Table S18**: Per-dataset evaluation summary (Avg F1, genus F1, species F1) for the 5 principal tools across each of the 7 CAMI assembly datasets.

**Supplementary Table S19**: Full genus-level comparison table for the ZymoGut D6331 case study, listing all 14 expected and 8 false-positive genera with truth and predicted abundances.

**Supplementary Figures S1 and S2**: Line plots showing how tool performance varies with mutation rate across taxonomic levels for each biological domain.

**Supplementary Figure S3**: Scatter plots relating execution time (hours) to F1 score (0.0–1.0) for all evaluated tools, presented per domain.

**Supplementary Figure S4**: F1 scores at 0% mutation for all tools from the mutation-study tool set, grouped by taxonomic domain.

**Supplementary Figure S5**: HYMET per-domain mutation resilience: F1 across taxonomic levels (kingdom to species) for each of 9 biological groups under mutation rates 0–30%.

**Supplementary Figure S6**: Comparison of HYMET contig vs. synthetic-read workflows: mean F1 by rank and abundance distances (L1, Bray–Curtis).

**Supplementary Figure S7**: Resource usage comparison between HYMET contig and read workflows: wall-clock time and peak memory.

**Supplementary Figure S8**: Abundance heatmap for the human gut case study, showing relative abundance across taxonomic ranks.

**Supplementary Figure S9**: Abundance heatmap for the ZymoBIOMICS mock community case study, showing relative abundance across taxonomic ranks.

**Supplementary Figure S10**: Genus-level abundance comparison (Cleveland dot plot) for the ZymoGut D6331 case study, showing ground truth and HYMET predicted abundances for all detected genera.

## List of abbreviations

BASTA: BAsic Sequence Taxonomy Annotation; BLAST: Basic Local Alignment Search Tool; CAMI: Critical Assessment of Metagenome Interpretation; CAMITAX: CAMI TAXonomy (tool for taxon labels); CMash: Containment MinHash; CPU: central processing unit; CSV: comma-separated values; DOI: digital object identifier; F1: Harmonic mean of precision and recall; FCT: Fundação para a Ciência e a Tecnologia; GCA: GenBank assembly accession; GCF: RefSeq assembly accession; GTDB: Genome Taxonomy Database; HYMET: Hybrid Metagenomic Tool; LCA: Lowest Common Ancestor; NCBI: National Center for Biotechnology Information; PAF: Pairwise mApping Format; RefSeq: NCBI Reference Sequence database; RSS: Resident Set Size; SSU rRNA: Small Subunit ribosomal RNA; TAMA: Taxonomy Analysis pipeline for metagenome using meta-analysis; TaxID: taxonomy identifier; TSV: tab-separated values.

## Supplementary Material

giag024_Supplemental_File

giag024_Authors_Response_To_Reviewer_Comments_original_submission

giag024_Authors_Response_To_Reviewer_Comments_revision_1

giag024_GIGA-D-25-00184_original_submission

giag024_GIGA-D-25-00184_Revision_1

giag024_GIGA-D-25-00184_Revision_2

giag024_Reviewer_1_Report_original_submission Reviewer 1 -- 6/8/2025

giag024_Reviewer_1_Report_revision_1 Reviewer 1 -- 11/12/2025

giag024_Reviewer_1_Report_revision_2 Reviewer 1 -- 2/24/2026

giag024_Reviewer_2_Report_original_submission Reviewer 2 -- 6/16/2025

giag024_Reviewer_2_Report_revision_1 Reviewer 2 -- 11/3/2025

giag024_Reviewer_2_Report_revision_2 Reviewer 2 -- 2/15/2026

giag024_Reviewer_3_Report_original_submission Reviewer 3 -- 6/18/2025

giag024_Reviewer_3_Report_revision_1 Reviewer 3 -- 11/10/2025

## Data Availability

The supplementary material describes the full reproducibility workflow (environment setup, scripts, execution logs, and additional figures). Digital resources used in the manuscript are listed below. HYMET source code, CAMI/reads-vs.-contigs/case manifests, runtime logs, and aggregated TSV/figure outputs are versioned in the project repository (see the “Availability of source code” section; results/, bench/, and case/ subdirectories). The Syst_Review repository retains the systematic benchmark harness for third-party tools, including installation recipes and evaluation scripts [75]. The Mash sketch databases used for candidate selection (sketch1.msh, sketch2.msh, sketch3.msh) are deposited at Zenodo [76]; checksums are mirrored in the repository (sketch_sha256.txt). Numerical data underlying Figs 2–8 and Supplementary Figs S6–S10 are deposited at Zenodo [77], with a detailed README describing each file. CAMI contig assemblies used in the benchmark are enumerated in bench/cami_manifest.tsv; running bench/fetch_cami.sh downloads the official CAMI I sample_0 bundle from the Publisso mirror [78], extracts the required assets into /data/cami/, caches the archive, and regenerates the lightweight subsets (cami_i_*, cami_ii_*) via tools/generate_cami_subsets.py so every benchmark run starts from the same inputs. The test and validation dataset (26,203 genomes, 14.76 GB) was derived from the NCBI RefSeq Assembly database [79]; assembly summary files and scripts for replication are provided in Supplementary Material Section 3. Reference genomes for dynamic database construction are retrieved from the NCBI Assembly database [80] using GCF and GCA accession numbers identified through Mash screening. GTDB r202 genomes included in the sketch databases are available from the Genome Taxonomy Database [81]. Case-study inputs can be fetched with case/fetch_case_data.sh, which retrieves the Zymo mock assembly [82] and the MGnify gut assembly [83] before staging them under /data/case/. The manifest case/manifest.tsv records the same paths for reference. Zymo ablation outputs (candidate lists, cached references, evaluation tables, and figures) are versioned under results/ablation/canonical/run_20251031T191804Z/; the workflow can be rerun with case/run_ablation.sh as documented in Supplementary Section 6. ZymoGut D6331 case-study inputs comprise Oxford Nanopore SUP basecalls from the MicroBench collection (ENA accession ERR14251410) assembled with Flye, and manufacturer reference genomes from the Zymo Research D6331 RefSeq package. Ground-truth labels and evaluation scripts are provided in case/zymogut/; analysis outputs are versioned under results/cases/zymogut/.

## References

[1] Kim D, Song L, Breitwieser F P, et al. Centrifuge: rapid and sensitive classification of metagenomic sequences. Genome Res. 2016;26:1721–29. 10.1101/gr.210641.116. 27852649 PMC5131823

[2] Simon H Y, Siddle K J, Park D J, et al. Benchmarking metagenomics tools for taxonomic classification. Cell. 2019;178:779–94. 10.1016/j.cell.2019.07.010. 31398336 PMC6716367

[3] Wood D E, Salzberg S L. Kraken: ultrafast metagenomic sequence classification using exact alignments. Genome Biol. 2014;15:1–12. 10.1186/gb-2014-15-3-r46. PMC4053813 24580807

[4] Bremges A, Fritz A, McHardy A C. CAMITAX: taxon labels for microbial genomes. GigaScience. 2020;9:giz154. 10.1093/gigascience/giz154. 31909794 PMC6946028

[5] Kim N, Ma J, Kim W, et al. Genome-resolved metagenomics: a game changer for microbiome medicine. Exp Mol Med. 2024;56:1501–12. 38945961 10.1038/s12276-024-01262-7 PMC11297344

[6] Mallawaarachchi V, Lin Y. Accurate binning of metagenomic contigs using composition, coverage, and assembly graphs. J Comput Biol. 2022;29:1357–76. 10.1089/cmb.2022.0262. 36367700

[7] Ayling M, Clark M D, Leggett R M. New approaches for metagenome assembly with short reads. Brief Bioinform. 2020;21:584–94. 10.1093/bib/bbz020. 30815668 PMC7299287

[8] Wood D E, Lu J, Langmead B. Improved metagenomic analysis with Kraken 2. Genome Biol. 2019;20:1–13. 10.1186/s13059-019-1891-0. 31779668 PMC6883579

[9] Lema N K, Gemeda M T, Woldesemayat A A. Recent advances in metagenomic approaches, applications, and challenges. Curr Microbiol. 2023;80:347. 10.1007/s00284-023-03451-5. 37733134

[10] Martins I B, Silva J M, Almeida J R. A comprehensive study of databases to assess the reliability of metagenomic tools. In: 2024 IEEE Conference on Computational Intelligence in Bioinformatics and Computational Biology (CIBCB). Natal, Brazil: IEEE; 2024:1–6. 10.1109/CIBCB58642.2024.10702118.

[11] Xu R, Rajeev S, Salvador L C. The selection of software and database for metagenomics sequence analysis impacts the outcome of microbial profiling and pathogen detection. PLoS One. 2023;18:e0284031. 10.1371/journal.pone.0284031. 37027361 PMC10081788

[12] Breitwieser F P, Lu J, Salzberg S L. A review of methods and databases for metagenomic classification and assembly. Brief Bioinform. 2019;20:1125–36. 10.1093/bib/bbx120. 29028872 PMC6781581

[13] Kieser S, Brown J, Zdobnov E M, et al. ATLAS: a Snakemake workflow for assembly, annotation, and genomic binning of metagenome sequence data. BMC Bioinformatics. 2020;21:1–8. 10.1186/s12859-020-03585-4. 32571209 PMC7310028

[14] Tadrent N, Dedeine F, Hervé V. SnakeMAGs: a simple, efficient, flexible and scalable workflow to reconstruct prokaryotic genomes from metagenomes. F1000Research. 2022;11:1522. 10.12688/f1000research.128091.2. 36875992 PMC9978240

[15] Tamames J, Puente-Sánchez F. SqueezeMeta, a highly portable, fully automatic metagenomic analysis pipeline. Front Microbiol. 2019;9:425882. 10.3389/fmicb.2018.03349. PMC6353838 30733714

[16] Clarke E L, Taylor L J, Zhao C, et al. Sunbeam: an extensible pipeline for analyzing metagenomic sequencing experiments. Microbiome. 2019;7:1–13. 10.1186/s40168-019-0658-x. 30902113 PMC6429786

[17] Chaumeil P A, Mussig A J, Hugenholtz P, et al. GTDB-Tk: a toolkit to classify genomes with the Genome Taxonomy Database. Bioinformatics. 2020;36:1925–27. 10.1093/bioinformatics/btz848. PMC7703759 31730192

[18] Buchfink B, Xie C, Huson D H. Fast and sensitive protein alignment using DIAMOND. Nat Methods. 2015;12:59–60. 10.1038/nmeth.3176. 25402007

[19] Kahlke T, Ralph P J. BASTA—taxonomic classification of sequences and sequence bins using last common ancestor estimations. Methods Ecol Evol. 2019;10:100–103. 10.1111/2041-210X.13095.

[20] Ondov B D, Treangen T J, Melsted P, et al. Mash: fast genome and metagenome distance estimation using MinHash. Genome Biol. 2016;17:1–14. 10.1186/s13059-016-0997-x. 27323842 PMC4915045

[21] Menzel P, Ng K L, Krogh A. Fast and sensitive taxonomic classification for metagenomics with Kaiju. Nat Commun. 2016;7:11257. 10.1038/ncomms11257. 27071849 PMC4833860

[22] Callahan B J, McMurdie P J, Rosen M J, et al. DADA2: high-resolution sample inference from Illumina amplicon data. Nat Methods. 2016;13:581–83. 10.1038/nmeth.3869. 27214047 PMC4927377

[23] Sim M, Lee J, Lee D, et al. TAMA: improved metagenomic sequence classification through meta-analysis. BMC Bioinformatics. 2020;21:1–17. 10.1186/s12859-020-3533-7. 32397982 PMC7218625

[24] Ounit R, Wanamaker S, Close T J, et al. CLARK: fast and accurate classification of metagenomic and genomic sequences using discriminative *k*-mers. BMC Genomics. 2015;16:1–13. 10.1186/s12864-015-1419-2. 25879410 PMC4428112

[25] Breitwieser F P, Baker D N, Salzberg S L. KrakenUniq: confident and fast metagenomics classification using unique *k*-mer counts. Genome Biol. 2018;19:198. 10.1186/s13059-018-1568-0. 30445993 PMC6238331

[26] Piro V C, Dadi T H, Seiler E, et al. ganon: precise metagenomics classification against large and up-to-date sets of reference sequences. Bioinformatics. 2020;36:i12–20. 10.1093/bioinformatics/btaa458. 32657362 PMC7355301

[27] Piro V C, Reinert K. ganon2: up-to-date and scalable metagenomics analysis. NAR Genom Bioinform. 2025;7:lqaf094. 10.1093/nargab/lqaf094. 40677913 PMC12267982

[28] Song L, Langmead B. Centrifuger: lossless compression of microbial genomes for efficient and accurate metagenomic sequence classification. Genome Biol. 2024;25:106. 10.1186/s13059-024-03244-4. 38664753 PMC11046777

[29] Ulrich J U, Renard B Y. Fast and space-efficient taxonomic classification of long reads with hierarchical interleaved XOR filters. Genome Res. 2024;34:914–24. 10.1101/gr.278623.123. 38886068 PMC11293544

[30] Brown C T, Irber L. sourmash: a library for MinHash sketching of DNA. J Open Source Softw. 2016;1:27. 10.21105/joss.00027.

[31] Shang J, Peng C, Liao H, et al. PhaBOX: a web server for identifying and characterizing phage contigs in metagenomic data. Bioinform Adv. 2023;3:vbad101. 10.1093/bioadv/vbad101. 37641717 PMC10460485

[32] Shang J, Jiang J, Sun Y. Bacteriophage classification for assembled contigs using graph convolutional network. Bioinformatics. 2021;37:i25–33. 10.1093/bioinformatics/btab293. 34252923 PMC8275337

[33] Zhou Z, Martin C, Kosmopoulos J C, et al. ViWrap: a modular pipeline to identify, bin, classify, and predict viral–host relationships for viruses from metagenomes. iMeta. 2023;2:e118. 10.1101/2023.01.30.526317. 38152703 PMC10751022

[34] Auslander N, Gussow A B, Benler S, et al. Seeker: alignment-free identification of bacteriophage genomes by deep learning. Nucleic Acids Res. 2020;48:e121. 10.1093/nar/gkaa856. 33045744 PMC7708075

[35] Gałan W, Bąk M, Jakubowska M. Host taxon predictor—a tool for predicting taxon of the host of a newly discovered virus. Sci Rep. 2019;9:3436. 10.1038/s41598-019-39847-2. 30837511 PMC6400966

[36] Jiang G, Zhang J, Zhang Y, et al. DCiPatho: deep cross-fusion networks for genome scale identification of pathogens. Brief Bioinform. 2023;24:bbad194. 10.1093/bib/bbad194. 37249547 PMC10359081

[37] Altschul S F, Gish W, Miller W, et al. Basic local alignment search tool. J Mol Biol. 1990;215:403–10. 10.1016/S0022-2836(05)80360-2. 2231712

[38] Gruber-Vodicka H R, Seah B K, Pruesse E. phyloFlash: rapid small-subunit rRNA profiling and targeted assembly from metagenomes. mSystems. 2020;5:10–1128. 10.1128/mSystems.00920-20. PMC7593591 33109753

[39] Truong D T, Franzosa E A, Tickle T L, et al. MetaPhlAn2 for enhanced metagenomic taxonomic profiling. Nat Methods. 2015;12:902–903. 10.1038/nmeth.3589. 26418763

[40] Lui W W, Leung A W, Leung H C, et al. MegaPath-Nano: accurate compositional analysis and drug-level antimicrobial resistance detection software for Oxford nanopore long-read metagenomics. In: 2020 IEEE International Conference on Bioinformatics and Biomedicine (BIBM). Seoul, South Korea: IEEE; 2020:329–36. 10.1109/BIBM49941.2020.9313313.

[41] Li H . Minimap2: pairwise alignment for nucleotide sequences. Bioinformatics. 2018;34:3094–100. 10.1093/bioinformatics/bty191. 29750242 PMC6137996

[42] Liang X, Zhang J, Kim Y, et al. ARGem: a new metagenomics pipeline for antibiotic resistance genes: metadata, analysis, and visualization. Front Genet. 2023;14:1219297. 10.3389/fgene.2023.1219297. 37811141 PMC10558085

[43] Prosperi M, Marini S. Karga: multi-platform toolkit for *k*-mer-based antibiotic resistance gene analysis of high-throughput sequencing data. In: 2021 IEEE EMBS International Conference on Biomedical and Health Informatics (BHI). Athens, Greece: IEEE; 2021:1–4. 10.1109/BHI50953.2021.9508479. PMC8383893 34447942

[44] LaPierre N, Alser M, Eskin E, et al. Metalign: efficient alignment-based metagenomic profiling via containment min hash. Genome Biol. 2020;21:242. 10.1186/s13059-020-02159-0. 32912225 PMC7488264

[45] Olawoye I B, Frost S D, Happi C T. The Bacteria Genome Pipeline (BAGEP): an automated, scalable workflow for bacteria genomes with Snakemake. PeerJ. 2020;8:e10121. 10.7717/peerj.10121. 33194387 PMC7597632

[46] Ondov B D, Starrett G J, Sappington A, et al. Mash Screen: high-throughput sequence containment estimation for genome discovery. Genome Biol. 2019;20:1–13. 10.1186/s13059-019-1841-x. 31690338 PMC6833257

[47] Baker D N, Langmead B. Dashing: fast and accurate genomic distances with HyperLogLog. Genome Biol. 2019;20:1–12. 10.1186/s13059-019-1875-0. 31801633 PMC6892282

[48] Besta M, Kanakagiri R, Mustafa H, et al. Communication-efficient Jaccard similarity for high-performance distributed genome comparisons. In: 2020 IEEE International Parallel and Distributed Processing Symposium (IPDPS). New Orleans, Louisiana USA: IEEE; 2020:1122–32. 10.1109/IPDPS47924.2020.00118.

[49] Zhao X . BinDash, software for fast genome distance estimation on a typical personal laptop. Bioinformatics. 2019;35:671–73. 10.1093/bioinformatics/bty651. 30052763

[50] Katz L S, Griswold T, Morrison S S, et al. Mashtree: a rapid comparison of whole genome sequence files. J Open Source Softw. 2019;4:10–21105. 10.21105/joss.01762. PMC9380445 35978566

[51] Broder A Z . On the resemblance and containment of documents. In: Proceedings. Compression and Complexity of SEQUENCES 1997 (Cat. No. 97TB100171). Salerno, Italy: IEEE; 1997:21–29. 10.1109/SEQUEN.1997.666900.

[52] Pierce N T, Irber L, Reiter T, et al. Large-scale sequence comparisons with sourmash. F1000Research. 2019;8:1006. 10.12688/f1000research.19675.1. 31508216 PMC6720031

[53] Hernández-Salmerón J E, Moreno-Hagelsieb G. FastANI, Mash and Dashing equally differentiate between *Klebsiella* species. PeerJ. 2022;10:e13784. 10.7717/peerj.13784. 35891643 PMC9308963

[54] Hera M R, Liu S, Wei W, et al. Metagenomic functional profiling: to sketch or not to sketch?. Bioinformatics. 2024;40:ii165–173. 10.1093/bioinformatics/btae397. 39230701 PMC11373326

[55] Wu W, Li B, Chen L, et al. A review for weighted minhash algorithms. IEEE Trans Knowl Data Eng. 2020;34:2553–73. 10.1109/TKDE.2020.3021067.

[56] Sánchez-Reyes A, Fernández-López M. Sketched reference databases for genome-based taxonomy and comparative genomics. Braz J Biol. 2022;84:e256673. 10.1590/1519-6984.256673. 36383786

[57] Liu S, Koslicki D. CMash: fast, multi-resolution estimation of *k*-mer-based Jaccard and containment indices. Bioinformatics. 2022;38:i28–35. 10.1093/bioinformatics/btac237. 35758788 PMC9235470

[58] Mash Development Team . Mash tutorials. 2023. https://mash.readthedocs.io/en/latest/tutorials.html. Accessed 1 October 2025.

[59] Irber L, Brooks P T, Reiter T, et al. Lightweight compositional analysis of metagenomes with FracMinHash and minimum metagenome covers. bioRxiv. 2022; 10.1101/2022.01.11.475838.

[60] Kitts P A, Church D M, Thibaud-Nissen F, et al. Assembly: a resource for assembled genomes at NCBI. Nucleic Acids Res. 2016;44:D73–80. 10.1093/nar/gkv1226. 26578580 PMC4702866

[61] Schoch C L, Ciufo S, Domrachev M, et al. NCBI Taxonomy: a comprehensive update on curation, resources and tools. Database. 2020;2020:baaa062. 10.1093/database/baaa062. 32761142 PMC7408187

[62] Li H . Minimap and miniasm: fast mapping and de novo assembly for noisy long sequences. Bioinformatics. 2016;32:2103–10. 10.1093/bioinformatics/btw152. 27153593 PMC4937194

[63] Dong J, Liu X, Sadasivan H, et al. mm2-gb: GPU accelerated Minimap2 for long read DNA mapping. In: Proceedings of the 15th ACM International Conference on Bioinformatics, Computational Biology and Health Informatics. Shenzhen China: ACM; 2024:1–9. 10.1145/3698587.3701366.

[64] Langmead B, Wilks C, Antonescu V, et al. Scaling read aligners to hundreds of threads on general-purpose processors. Bioinformatics. 2019;35:421–32. 10.1093/bioinformatics/bty648. 30020410 PMC6361242

[65] Rosen G, Garbarine E, Caseiro D, et al. Metagenome fragment classification using *N*-mer frequency profiles. Adv Bioinform. 2008;2008:205969. 10.1155/2008/205969. PMC2777009 19956701

[66] Liu B, Gibbons T, Ghodsi M, et al. Accurate and fast estimation of taxonomic profiles from metagenomic shotgun sequences. Genome Biol. 2011;12:1–27. 10.1186/1471-2164-12-S2-S4. PMC3194235 21989143

[67] Pruitt K D, Tatusova T, Maglott D R. NCBI reference sequences (RefSeq): a curated non-redundant sequence database of genomes, transcripts and proteins. Nucleic Acids Res. 2007;35:D61–65. 10.1093/nar/gkl842. 17130148 PMC1716718

[68] O’Leary N A, Wright M W, Brister J R, et al. Reference sequence (RefSeq) database at NCBI: current status, taxonomic expansion, and functional annotation. Nucleic Acids Res. 2016;44(D1):D733–45. 10.1093/nar/gkv1189. 26553804 PMC4702849

[69] Sayers E W, Beck J, Bolton E E, et al. Database resources of the national center for biotechnology information. Nucleic Acids Res. 2021;49:D10. 10.1093/nar/gkaa892. 33095870 PMC7778943

[70] Jesus T F, Ribeiro-Gonçalves B, Silva D N, et al. Plasmid ATLAS: plasmid visual analytics and identification in high-throughput sequencing data. Nucleic Acids Res. 2019;47:D188–94. 10.1093/nar/gky1073. 30395323 PMC6323984

[71] Blanco-Míguez A, Beghini F, Cumbo F, et al. Extending and improving metagenomic taxonomic profiling with uncharacterized species using MetaPhlAn 4. Nat Biotechnol. 2023;41:1633–44. 10.1038/s41587-023-01688-w. 36823356 PMC10635831

[72] Burki F, Roger A J, Brown M W, et al. The new tree of eukaryotes. Trends Ecol Evol. 2020;35:43–55. 10.1016/j.tree.2019.08.008. 31606140

[73] Simmonds P, Adams M J, Benkő M, et al. Virus taxonomy in the age of metagenomics. Nat Rev Microbiol. 2017;15:161–68. 10.1038/nrmicro.2016.177. 28134265

[74] Harris H M, Hill C. A place for viruses on the tree of life. Front Microbiol. 2021;11:604048. 10.3389/fmicb.2020.604048. 33519747 PMC7840587

[75] Martins I B, Silva J M, Almeida J R. Syst_Review: systematic benchmark harness for metagenomic classification tools. GitHub. 2024. https://github.com/inesbmartins02/Syst_Review.

[76] Silva J M, Martins I B, Almeida J R. HYMET Mash Sketch Databases (sketch1.msh, sketch2.msh, sketch3.msh). Zenodo. 2025. 10.5281/zenodo.17428354.

[77] Silva J M, Martins I B, Almeida J R. HYMET numerical data underlying figures 2–8 and supplementary figures S6–S10. Zenodo. 2025. 10.5281/zenodo.18772511.

[78] CAMI Challenge . CAMI I Sample 0 Contig Assemblies. 2017. https://frl.publisso.de/data/frl:6421672/dataset/2017.12.29_11.37.26_sample_0_contigs.tar.Accessed 15 September 2025.

[79] NCBI . NCBI RefSeq Genomes FTP. 2025. https://ftp.ncbi.nlm.nih.gov/genomes/refseq/. Accessed 15 September 2025.

[80] NCBI . NCBI Assembly Database. 2025. https://www.ncbi.nlm.nih.gov/assembly/.Accessed 15 September 2025.

[81] Genome Taxonomy Database . GTDB: Genome Taxonomy Database. 2025. https://gtdb.ecogenomic.org/. Accessed 20 December 2025.

[82] Loman Lab . ZymoBIOMICS Mock Community Nanopore Assembly. 2019. http://nanopore.s3.climb.ac.uk/mockcommunity/v3/7cd60d3b-eafb-48d1-9aab-c8701232f2f8.ctg.cns.fa.Accessed 20 December 2025.

[83] MGnify . MGnify Gut Metagenome Analysis MGYA00794604. 2025. https://www.ebi.ac.uk/metagenomics/api/v1/analyses/MGYA00794604/file/ERZ24911249_FASTA.fasta.gz.Accessed 20 December 2025.

