# Supplementary material for: HYMET: a hybrid metagenomic pipeline for accurate and efficient taxonomic classification
Source: Gigascience. 2026 Mar 2;15:giag024. doi: 10.1093/gigascience/giag024 (PMC13042306; doi:10.1093/gigascience/giag024)
Supplement: giag024_Supplemental_File [file giag024_supplemental_file.pdf]

Supplementary Material of: HYMET: A Hybrid Metagenomic Pipeline for Accurate and Efficient Taxonomic Classification

Jorge Miguel Silva[a*], Inês Martins[a], João Rafael Almeida[a]
[a]IEETA/DETI, LASI, University of Aveiro, Aveiro, Portugal
*jorge.miguel.ferreira.silva@ua.pt

HYMET is a hybrid metagenomic tool designed for taxonomic identification and analysis of metagenomic sequences. It combines multiple approaches to provide accurate and reliable results. This section describes the installation, configuration, and usage of HYMET.

1 Installation

HYMET is distributed via Bioconda and as an open-source repository hosted at https://github.com/ieeta-pt/HYMET. The commands below assume a Unix-like shell; substitute `conda` for `mamba` if Mamba is not available.

1.1 Install with Bioconda (Recommended)

```
mamba install -c bioconda hymet
hymet --help
```

The `hymet` executable exposes the unified CLI described in Section 4.

1.2 Source Checkout

```
git clone https://github.com/ieeta-pt/HYMET.git
cd HYMET
mamba env create -f environment.yml -n hymet
mamba activate hymet
bin/hymet --help
```

The CLI auto-detects the repository root; exporting `HYMET_ROOT=$(pwd)` may be necessary when running from other directories.

1.3 Container Images

```
docker build -t ieeta-hymet .
docker run --rm -it ieeta-hymet hymet --help
```

For HPC environments without Docker, use Singularity/Apptainer with the provided definition file (`Singularity.def`):

```
apptainer build hymet.sif Singularity.def
apptainer exec hymet.sif hymet --help
```

Bind-mount reference or output directories as needed when executing analyses inside the container.

2 Configuration

2.1 Reference Sketched Databases

HYMET relies on the Mash sketches published alongside this revision: **HYMET Sketch Databases v1** (doi:10.5281/zenodo.17428354). The repository provides a helper that downloads the three sketches directly from Zenodo and verifies their checksums:

```
cd HYMET
tools/fetch_sketches.sh          # downloads sketch{1,2,3}.msh into HYMET/data
tools/verify_sketches.sh         # optional: confirm hashes against the Zenodo
    record
```

Both scripts default to the Zenodo record above; override `-base-url` if the files are mirrored elsewhere. The verification step compares local SHA256 hashes with the published `sketch_sha256.txt`, prints a clear PASS/FAIL report including file sizes, and ensures the sketches used in analyses match the archival release.

2.2 Reproducing Sketched Databases (Optional)

HYMET ships with pre-computed Mash sketches (`data/sketch1.msh`, `sketch2.msh`, `sketch3.msh`) distributed alongside the Bioconda package and repository release assets. The unified CLI consumes these files directly. Nevertheless, augmenting the bundled sketches with a curated custom layer (`custom1.msh`, `custom2.msh`) substantially improved recall for underrepresented eukaryotic and viral clades in our experiments. The steps below recreate the exact database build used for the reported results.

Prerequisites.

- Mash (installed with the HYMET environment).
- A working directory (e.g., `build_sketches/`) with subfolders `genomes/` and `additional/` for downloaded FASTA files.

1. Base RefSeq sketch.

```
wget https://mash.readthedocs.io/en/latest/_downloads/RefSeq88n.msh.gz
gunzip RefSeq88n.msh.gz
mash paste sketch1.msh RefSeq88n.msh
```

2. Curated public sketches.

```
git clone https://github.com/ayixon/Mash-sketched-reference-databases.git refs
cp refs/GTDB_r202_assembly_set.msh .
cp refs/Bacteria_Archaea_type_assembly_set.msh .
cp refs/Fungi_type_assembly_set.msh .
cp refs/Virus_Sept21_GenBank_assembly_set.msh .
```

3. Download additional genomes.
Use the repository helper to pull FASTA files listed in an NCBI-derived manifest:

```
python3 scripts/downloadDB.py \
  selected_genomes.txt \
  build_sketches/genomes \
  build_sketches/taxonomy.tsv \
  build_sketches/cache
```

where `selected_genomes.txt` contains FTP prefixes from assembly summary files (vertebrate mammal/other, invertebrate, plant, archaea, protozoa, virus). For transparency, the lightweight Bash downloader used during early development is retained below:

```
#!/bin/bash
INPUT_FILE="$1"
OUTPUT_DIR="${2:-genomes}"
mkdir -p "$OUTPUT_DIR"

download_genome() {
  local ftp_path="$1"
  local filename=$(basename "$ftp_path")
  local url="${ftp_path}/${filename}_genomic.fna.gz"
  wget -q "$url" -O "${OUTPUT_DIR}/${filename}.fna.gz"
  gunzip -f "${OUTPUT_DIR}/${filename}.fna.gz"
}

while IFS=$'\t' read -r ftp_path _; do
  [[ "$ftp_path" =~ ^# ]] || [[ -z "$ftp_path" ]] && continue
  download_genome "$ftp_path"
done < "$INPUT_FILE"
```

4. Generate custom sketches.

```
mash sketch -k 21 -s 1000 -o custom1.msh build_sketches/genomes/*.fna
mash paste sketch2.msh \
  GTDB_r202_assembly_set.msh \
  Virus_Sept21_GenBank_assembly_set.msh \
  Fungi_type_assembly_set.msh \
  Bacteria_Archaea_type_assembly_set.msh \
  custom1.msh

mash sketch -k 15 -s 5000 -o custom2.msh build_sketches/additional/*.fna
mash paste sketch3.msh custom2.msh
```

Copy `sketch1.msh`, `sketch2.msh`, and `sketch3.msh` into `data/`. Retaining this custom supplement reproduces the uplift reported in the plant, protozoa, and viral benchmark tables.

2.3 On-disk Resources Used in Benchmark

This table reports the on-disk footprint we used when running the benchmarked tools (install + bundled/reference data as configured in our environment). These values are disk usage, not runtime resources; cross-tool wall-time and peak RAM are summarized in the CAMI figures/tables in the main manuscript.

Tool	On-disk resources (GB)	Notes
HYMET	2.82 (+10–50 at runtime)	Bioconda install; dynamic cache depends on candidate budget and sample composition.
Kraken 2	60	bench/db/kraken2 (full build used in CAMI runs).
Centrifuge	0.50	bench/db/centrifuge.
Ganon 2	0.04	bench/db/ganon2 (IBF/HIBF index).
MetaPhlAn 4	34	bench/db/metaphlan (mpa_vJun23_CHOCOPhlAnSGB_202307).
sourmash gather	0.07	bench/db/sourmash (scaled signatures used).
BASTA	40	As used in our environment.
CAMITAX	33	As used in our environment.
MegaPath-Nano	71	As used in our environment.
PhaBOX	2.8	As used in our environment.
PhyloFlash	16	As used in our environment.
SnakeMAGs	67	As used in our environment.
SqueezeMeta	403	As used in our environment.
ViWrap	2	As used in our environment.
TAMA	285	As used in our environment.

Table S1: On-disk resources (install + references) used in our canonical CAMI benchmark environment. Entries reflect the exact tool configurations invoked by the bench harness; values are disk usage, not peak RAM or wall time.

2.4 Initial Setup

The supported entry point is the Python CLI (`bin/hymet` from a source checkout or `hymet` when installed via Bioconda). The CLI bootstraps taxonomy data on demand: whenever `run_hymet_cami.sh` (invoked by the `bin/hymet run` and `bench` subcommands) detects missing files under `taxonomy_files/`, it executes `config.pl`, which downloads `names.dmp`, `nodes.dmp`, and produces `data/taxonomy_hierarchy.tsv`. The detailed accession to ,TaxID map (`data/detailed_taxonomy.tsv`) is generated per-run by `scripts/downloadDB.py` for the selected candidate set and is symlinked by the runner. No manual `chmod` or direct `./config.pl` invocation is required unless the legacy Perl workflow explicitly referenced in earlier releases is being reproduced.

2.5 HYMET's Structure

The repository layout combines the modern CLI, benchmark harness, and legacy scripts:

- **bin**/: Python entry points (`bin/hymet`) exposing `run`, `bench`, `case`, `ablation`, the truth-set helper `truth build-zymo`, and the legacy bridge `legacy` subcommands.
- **bench**/: CAMI benchmark harness, including database builders (`bench/db`), runners (`run_*.sh`), aggregated results (`bench/out/`), and plotting scripts (`bench/plot/make_figures.py`).
- **case**/: Real-data case study and ablation toolkit with manifests, fetch scripts, and evaluation helpers.
- **scripts**/: Legacy Perl/Python/Bash helpers (e.g., `mash.sh`, `downloadDB.py`, `classification.py`), retained for reproducibility and reused internally by the CLI.
- **data**/: Contains `sketch*.msh`, `taxonomy_hierarchy.tsv`, and symlinks to cached references; runtime caches live under `data/downloaded_genomes/cache/`.

- **taxonomy_files/**: NCBI taxonomy dump retrieved automatically by `config.pl`.
- **testdataset/**: Standalone scripts for constructing mini evaluation datasets.
- **output/**: Legacy Perl workflow output directory; the CLI writes to user-specified paths instead.

3 Test Data

Once the package is downloaded, input data must be prepared for analysis. HYMET requires input files in **FASTA format** (e.g., `.fna`/`.fa`/`.fasta`). Each file should contain metagenomic sequences with headers in the following format:

```
>read_id additional_info
SEQUENCE_DATA
```

3.1 Setting up input data

Workflow. Provide HYMET with a single multi-FASTA (or a directory of `.fna` files) when invoking the CLI:

```
bin/hymet run --contigs /path/to/sample.fna --out /path/to/output --threads 16
```

The command stages input files under `input/`. No manual editing of `main.pl` or contig splitting is required for the supported workflow.

Legacy recipe (optional). To reproduce experiments with the historical Perl entry point (`main.pl`), `.fna` files should be placed in the directory referenced by `$input_dir` inside `main.pl`. For a single combined FASTA, it may be split into uniform chunks:

```
split -n l/10 sample1.fna read_

for file in read_*; do
  read_number=${file#read_}
  sed -i "1i >read$read_number" "$file"
  mv "$file" "${file}.fna"
done
```

These steps are legacy guidance and should only be followed when running `./main.pl`. For all new analyses, prefer the CLI example above.

3.2 Replicating the Benchmark Dataset

The package includes scripts to reproduce the benchmark dataset (10% per domain sampling) and a bundled subset for quick validation. Install the helper dependencies before running the scripts:

```
pip install wget requests beautifulsoup4 biopython
```

These utilities reside in `testdataset/` and write outputs to `data/testdataset/`. The command-line `simulate_mutations.py` supersedes the older interactive mutation script, but both remain available for reproducibility.

To reproduce the miniature dataset:

```
# Navigate to test dataset directory
cd testdataset
```

Step 1: Download Assembly Files

```
# Download assembly files for each domain
wget "https://ftp.ncbi.nlm.nih.gov/genomes/refseq/archaea/assembly_summary.txt" # Archaea
wget "https://ftp.ncbi.nlm.nih.gov/genomes/refseq/bacteria/assembly_summary.txt" # Bacteria
wget "https://ftp.ncbi.nlm.nih.gov/genomes/refseq/fungi/assembly_summary.txt" # Fungi
wget "https://ftp.ncbi.nlm.nih.gov/genomes/refseq/invertebrate/assembly_summary.txt" # Invertebrate
wget "https://ftp.ncbi.nlm.nih.gov/genomes/refseq/plant/assembly_summary.txt" # Plant
wget "https://ftp.ncbi.nlm.nih.gov/genomes/refseq/protozoa/assembly_summary.txt" # Protozoa
wget "https://ftp.ncbi.nlm.nih.gov/genomes/refseq/vertebrate_mammalian/assembly_summary.txt" # Vertebrate Mammalian
wget "https://ftp.ncbi.nlm.nih.gov/genomes/refseq/vertebrate_other/assembly_summary.txt" # Vertebrate Other
wget "https://ftp.ncbi.nlm.nih.gov/genomes/refseq/viral/assembly_summary.txt" # Viral
```

Step 2: Download genomes

```
# Run create database script
python3 createDatabase.py
# You will be prompted for:
# - Path to assembly summaries directory
# - Path to destination directory for database sequences
# - Domain to process (e.g., archaea, bacteria, fungi)
```

Step 3: Filter genomes

```
# Filter 10% of each genome
python3 filterGCF.py
# You will be prompted for:
# - Path to input directory containing GCF files
# - Path to output directory
```

Step 4 (optional): Extract taxonomy metadata

```
# Extract taxonomy (optional)
python3 extractTaxonomy.py
# You will be prompted for:
# - Your email (required for NCBI E-utilities)
# - Path to directory containing domain .fna files
# - Path to assembly summary file
```

Step 5 (optional): Extract sequence IDs and map to each GCF

```
# Extract NC (optional)
python3 extractNC.py
# You will be prompted for:
# - Path to directory containing domain .fna files
# Output: .csv containing in the first column the genome accession (GCF) and in the second the corresponding sequence IDs (eg., NC_,NZ_)
```

Step 6 (optional): Introduce mutations

```
# Preferred CLI (deterministic)
python3 simulate_mutations.py \
  --fasta input.fna \
  --output mutated.fna \
```

```
  --sub-rate 0.1 \
  --indel-rate 0.01 \
  --max-indel-length 3 \
  --seed 1337

# Legacy interactive helper (still bundled)
python3 mutationGCF.py
```

Important Notes:

- Process each taxonomic group separately to avoid system overload
- The filtered genomes (10% of each) will be used as input for the tool
- Extracting taxonomy and sequence IDs is optional but can be useful for further analysis, especially performance evaluation and results comparison
- Introducing mutations is optional and can be used to simulate genetic variations
- When introducing mutations, the mutation rate must be a valid number between 0 and 1

4 Running HYMET

The maintained entry point for HYMET is the Python CLI exposed via `hymet` (Bioconda) or `bin/hymet` (source checkout). It orchestrates Mash candidate selection, reference caching, Minimap2 alignment, and the weighted-LCA classifier.

4.1 Single-Sample Classification

```
bin/hymet run \
  --contigs /path/to/sample.fna \
  --out /path/to/output \
  --threads 16
```

HYMET accepts a single multi-FASTA or many contigs within the same file, stages them under `input/`, and writes results to `<out>/classified_sequences.tsv`. The workflow keeps the contigs exactly as provided there is no automatic splitting or chunking so upstream preprocessing remains fully under user control.

Optional flags include `-cand-max` to cap Mash candidates and `-species-dedup` for species-level dereplication. No manual splitting of reads is required.

Reads example. HYMET also operates on reads (FASTQ/FASTA) when provided via `-reads`:

```
bin/hymet run \
  --reads /path/to/sample.fastq \
  --out /path/to/output \
  --threads 16
```

Reads mode maps with Minimap2 using the `sr` preset by default (overridable via `MINIMAP2_READS_PRESET`).

4.2 Benchmark and Reproducibility Runs

The benchmark harness is available through the same CLI:

```
bin/hymet bench \
  --manifest bench/cami_manifest.tsv \
  --tools hymet,kraken2,centrifuge,ganon2,metaphlan4,sourmash_gather \
  --threads 16
```

After the runner completes, regenerate the consolidated tables and figures:

```
python bench/aggregate_metrics.py --bench-root bench --outdir out
python bench/plot/make_figures.py --bench-root bench --outdir out
```

Keep the same `-outdir` value when comparing revisions so that existing TSVs and plots are overwritten in place. The aggregate script produces `summary_per_tool_per_sample.tsv`, `leaderboard_by_rank.tsv`, and `contig_accuracy_per_tool.tsv`. Runtime logs (`bench/out/runtime_memory.tsv`) are emitted during execution by `bench/lib/measure.sh`, and the plotting script writes figures under `bench/out/` (mirrored to `results/bench/` for convenience).

Alternatively, run `bin/hymet artifacts` to orchestrate these aggregation and plotting steps in one command when inputs already exist.

These aggregation and plotting steps run automatically at the end of `bench/run_all_cami.sh`; rerun the commands above manually only when regenerating outputs after ad hoc edits. For an end-to-end, reviewer-oriented protocol (environment creation, dataset acquisition, cache policy, figure regeneration, checksum verification), see `HYMET/docs/reproducibility.md`.

Behind the scenes, this command invokes `run_hymet_cami.sh`. To reproduce specific experiments verbatim, call the script directly while pinning its environment variables:

```
ROOT=$(pwd) CACHE_ROOT=data/downloaded_genomes/cache \
THREADS=16 CAND_MAX=200 SPECIES_DEDUP=1 \
bash run_hymet_cami.sh
```

Set `CACHE_ROOT` and related paths to control where downloaded references and Minimap2 indices are stored. Outputs appear under `bench/out/`; the aggregation/plotting commands above should be rerun if the workflow is modified and figures need to be regenerated.

Caching and reproducibility controls.

- `THREADS` specifies CPU parallelism used by Mash, minimap2, and the classifier; keeping it constant ensures comparable runtimes and peak-memory measurements.
- `CAND_MAX` and `SPECIES_DEDUP` limit the number of Mash candidates persisted to `output/selected_genomes.txt`. Keeping these values fixed guarantees identical candidate lists across reruns.
- `CACHE_ROOT` points to the directory where downloaded references, concatenated FASTA files, and Minimap2 indices are cached. The cache key is the SHA1 hash of `selected_genomes.txt`, so identical candidate lists reuse the same reference bundle.
- `FORCE_DOWNLOAD=1` forces a fresh download for the current candidate hash, ensuring consistency when upstream reference data changes.
- `ASSEMBLY_SUMMARY_DIR` provides mirrored NCBI assembly metadata. Supplying the same directory avoids non-determinism caused by remote fetches.

- MASH_THRESH controls Mash screening; SPLIT_IDX sets the Minimap2 index chunk size (-I) for memory control. For read-based runs, MINIMAP2_READS_PRESET (default sr) selects the Minimap2 preset.
- CAND_LIMIT_LOG optionally captures pruning diagnostics, documenting how many candidates were rejected at each stage.
- HYMET_REL_COV_THRESHOLD and HYMET_ABS_COV_THRESHOLD govern the minimum relative and absolute coverage required for a taxon to be reported by the classifier.
- HYMET_TAXID_MIN_SUPPORT and HYMET_TAXID_MIN_WEIGHT set the minimum supporting contig count and cumulative weight for a TaxID to be retained in the final classification.

These switches appear in the benchmark scripts with the values used in the manuscript (e.g., CAND_MAX=200, SPECIES_DEDUP=1). By default, the general runner uses CAND_MAX=5000; the CAMI harness wrapper defaults to CAND_MAX=1500 with SPECIES_DEDUP=1. Copying the manuscript values reproduces the reported tables without recalculating Mash thresholds or rebuilding caches.

Improving low-rank assignments. For genus/species-level calls in high-mutation or underrepresented taxa we recommend:

- Relax Mash screening (e.g., MASH_THRESH=0.85) and/or raise CAND_MAX so that divergent candidates are aligned before weighted-LCA resolution.
- Seed caches with curated panels via case/tools/preload_cache_from_fasta.py, which copies a user FASTA plus sequence-to-TaxID map into the cache, refreshes combined_genomes.fasta/detailed_taxonomy.tsv, and removes stale Minimap2 indices for reindexing.
- Tune low-rank filters by adjusting HYMET_REL_COV_THRESHOLD, HYMET_ABS_COV_THRESHOLD, HYMET_TAXID_MIN_SUPPORT, and HYMET_TAXID_MIN_WEIGHT; decreasing thresholds preserves low-coverage evidence, while higher values suppress spurious species calls.
- Use the ablation workflow (bin/hymet ablation) to quantify how removals of specific references alter genus/species recall; the resulting case/ablation_summary.tsv highlights taxa that merit targeted cache seeding.

Reference retrieval policy and fallbacks. HYMET's downloader retrieves assemblies over HTTPS using NCBI's RefSeq and GenBank catalogues and is resilient to transient failures. Specifically: (i) both RefSeq and GenBank assembly summaries are consulted so that records present in either catalogue can be resolved; (ii) downloads employ bounded retries with exponential backoff and switch to HTTPS to avoid legacy FTP issues; (iii) mirror/offline operation is supported by pointing ASSEMBLY_SUMMARY_DIR at a local copy of the assembly summaries and by pre-seeding CACHE_ROOT with curated panels (case/tools/preload_cache_from_fasta.py), after which HYMET rebuilds indices in place. Persistent failures are reported in the runtime log and do not halt classification when sufficient references are available.

When invoked through the CLI, HYMET stages the provided -contigs file internally, so no manual directory preparation is required beyond supplying a valid FASTA file with standard headers.

4.3 Case Study Workflows

Real-data evaluations and ablation experiments reside in `case/`. Replicate them with:

```
bin/hymet case \
  --manifest case/manifest.tsv \
  --out case/out \
  --threads 16
```

Optional helpers in `case/fetch_case_data.sh` download the MGnify gut assembly and Zymo mock community referenced in the manuscript. HYMET writes per-sample directories under `case/out/` (one subdirectory per sample) containing `classified_sequences.tsv`, `profile.cami.tsv`, and summary tables (`top_taxa.tsv`, runtime logs).

Manifest layout. The case manifest (`case/manifest.tsv`) is a tab-separated file with the following columns:

- `sample_id` – identifier used for output directories.
- `contigs_fa` – path or URL to the contig FASTA file.
- `truth_contigs_tsv` (optional) – contig-level truth table for evaluation.
- `truth_profile_tsv` (optional) – taxonomic abundance profile for evaluation.
- `expected_taxa`, `citation` (optional) – metadata recorded in `case/out/<sample>/metadata.json`.

Relative paths are resolved against `case/`.

Ablation experiments.

```
bin/hymet ablation \
  --sample zymo_mc \
  --taxa 1423,562 \
  --levels 0,0.5,1.0 \
  --threads 8 \
  --out case/ablation
```

This forwards to `case/run_ablation.sh`, which uses the same caching controls as the CAMI harness. Outputs populate `case/ablation/` with per-level HYMET runs, reference FASTA snapshots, and evaluation summaries (`case/ablation_summary.tsv`, `case/ablation_eval_summary.tsv`) when truth tables are provided. Each invocation honours `CACHE_ROOT`, `FORCE_DOWNLOAD`, and `THREADS`, so recording these values preserves reproducibility across case analyses.

Aggregating case outputs. Collect the generated TSVs directly from `case/out/` and `case/ablation/`. Each sample folder includes CAMI-formatted profiles, `top_taxa.tsv`, `metadata.json`, and runtime logs (`runtime_memory.tsv`). Ablation runs emit `case/ablation_summary.tsv`, per-level folders (for example, `case/ablation/zymo_mc/level_0.5/`), and, when truth data is present, `case/ablation_eval_summary.tsv`, matching the statistics reported in the supplementary figures.

Replicating the ZymoGut D6331 case study. The ZymoGut case study evaluates HYMET on the ZymoBIOMICS Gut Microbiome Standard (catalogue D6331), a 15-species mock community spanning bacteria, fungi, and archaea. All scripts reside in case/zymogut/; the master orchestrator run_zymogut.sh executes the six phases below in sequence:

1. **Download** (scripts/01_download.sh). Fetches Oxford Nanopore SUP basecalls from the MicroBench collection (ENA accession ERR14251410, barcode 13) and the 21 manufacturer reference genomes from NCBI using accessions listed in config/zymogut_d6331.yml.
2. **Assemble** (scripts/02_assemble.sh). Runs Flye in metagenomic mode (-nano-hq -meta) on the downloaded reads, producing a polished contig set.
3. **Build ground truth** (scripts/03_build_truth.sh). Aligns assembled contigs against the 21 reference genomes with minimap2 -x asm5, then derives contig-level taxonomic labels and a CAMI-format abundance profile from the best-hit mappings.
4. **Classify** (scripts/04_classify.sh). Runs the standard HYMET pipeline on the assembled contigs.
5. **Analyse** (scripts/05_analyse.sh). Computes evaluation metrics (Pearson correlation, Bray–Curtis dissimilarity, L1 distance) and generates all figures via plot_zymogut.py.
6. **Package** (scripts/06_package.sh). Copies final tables, figures, and metadata into results/cases/zymogut/ for integration with the manuscript repository.

To run the full pipeline end to end:

```
cd case/zymogut
bash run_zymogut.sh          # runs all six phases
```

Runtime is approximately 1.5–3 hours depending on available threads and network speed. Intermediate outputs are written to case/zymogut/work/; final results (tables, figures, metadata) are packaged under results/cases/zymogut/. The configuration file config/zymogut_d6331.yml records all reference accessions, expected taxa, and dataset metadata for provenance.

4.4 Additional CLI Utilities

The unified CLI also exposes utility subcommands beyond the main workflows. bin/hymet truth build-zymo provides a streamlined entry point to case/truth/build_zymo_truth.py, generating CAMI-compliant truth sets from the curated Zymo mock references when given contigs and a PAF alignment. Meanwhile, bin/hymet legacy - <args> forwards directly to main.pl, preserving the historical Perl pipeline for users who need complete parity with earlier releases. Use these helpers when reproducing the case-study truth tables or when validating results against the prior Perl implementation. For provenance capture, bin/hymet version prints the current HYMET commit hash.

4.5 Mash Candidate Control

HYMET still screens contigs with mash, but candidate limiting is handled by scripts/limit_candidates.py rather than a hard-coded multiplier. The workflow is:

1. mash screen is executed against the three sketches, producing score tables (output/*.tab).

2. `limit_candidates.py` merges the score tables, ranks accessions, and writes `output/selected_genomes.txt`.
3. The SHA1 of `selected_genomes.txt` seeds the reference cache used by `run_hymet_cami.sh`.

The following environment variables govern candidate breadth and reproducibility:

- `CAND_MAX` caps the number of accessions retained. The CAMI experiments set this to 200.
- `SPECIES_DEDUP=1` keeps only the top-scoring accession per species, reducing redundancy.
- `MASH_THRESH` (default 0.90) controls the minimum Mash score forwarded to the limiter. Lowering it increases sensitivity at the cost of additional mapping work.
- `CAND_LIMIT_LOG` records how many accessions pass each stage, providing an audit trail for supplementary tables.

Instead of tuning abstract multipliers, adjust `CAND_MAX`, `SPECIES_DEDUP`, and `MASH_THRESH` to trade off accuracy and runtime. Documenting these settings alongside benchmark results ensures the candidate pool can be recreated exactly.

4.6 Understanding HYMET Output

After running HYMET, the tool generates an output file with taxonomic classifications. The output is formatted as follows:

```
Query   Lineage   Taxonomic Level   TaxID   Confidence
seq1    superkingdom:Bacteria;phylum:Firmicutes;genus:Staphylococcus   genus   1279   0.9500
seq2    superkingdom:Bacteria;phylum:Proteobacteria;species:Escherichia_coli   species 562    0.8700
seq3    Unknown   root    -    0.0000
```

Each line in the output represents a classification result:

- **Query**: The identifier of the input sequence (e.g., seq1, seq2). These correspond to the sequence IDs in the input files (e.g., NC_...).
- **Lineage**: The taxonomic lineage assigned to the query sequence.
- **Taxonomic Level**: The most specific taxonomic level confidently assigned.
- **TaxID**: The NCBI TaxID associated with the reported lineage. Unresolved assignments appear as “-” when no confident taxon can be determined.
- **Confidence**: A score between 0 and 1 indicating the confidence of the classification.

Interpreting the Results:

- High confidence scores (close to 1) indicate more reliable classifications.
- "Unknown" in the Lineage column and "root" as Taxonomic Level indicate that the tool could not confidently classify the sequence.
- The Taxonomic Level shows the most specific classification the tool could make with confidence.

The Query column (seq1, seq2, etc.) corresponds to the sequence IDs in the input files (e.g., NC_...). These IDs can be cross-referenced with the original input to identify which sequences received which classifications. Supplementary DB-1 (Table S2) collates each tool's database provenance, build date, and size to make cross-tool comparisons reproducible.

Run outputs. Each HYMET run writes a consistent set of artefacts in the chosen output directory (--out):

- classified_sequences.tsv – one row per contig/read with lineage, rank, TaxID, and confidence.
- hymet.sample_0.cami.tsv (or case/out/<sample>/hymet/profile.cami.tsv in the case harness) – CAMI-formatted taxonomic profile for abundance analyses.
- resultados.paf – Minimap2 alignments for downstream inspection.
- metadata.json – reproducibility snapshot (HYMET commit, sketch checksums, cache key, parameters, tool versions).

5 State-of-the-art Benchmarking

All scripts and information required to reproduce the comprehensive evaluation of state-of-the-art tools, including installation, configuration, testing, and metric calculation, are available in the following GitHub repository: https://github.com/inesbmartins02/Syst_Review.git. The repository is organized as follows:

5.1 Directory Structure

- **Database**: Prepares input datasets for benchmarking.
- **Configuration**: Installs and configures tools.
- **Execution**: Runs tools with prepared inputs.
- **Outputs**: Processes results and calculates metrics.

5.2 Step 1: Prepare Test Dataset (Database Directory)

The dataset preparation follows Section 3.2, with every script executed, including the optional ones. The input file is then prepared as follows:

```
# Combine all GCFs into a single FASTA file (required for most tools)
python3 GCFtocombinedfasta.py
# - Path to directory containing domain .fna files
```

5.3 Step 2: Configure Tools (Configuration Directory)

- **Install Tools:** Only 9 of 31 tools were successfully validated. To install them:

  ```
  cd configuration
  chmod +x phabox.sh
  ./phabox.sh      # PhaBOX
  ...
  ```

  Each script installs dependencies and verifies tool functionality.

5.4 Step 3: Execute Tools (Execution Directory)

- Edit each tool's execution script (e.g., `run_metaphlan.sh`) to include:
  - Path to tool installation directory
  - Path to input FASTA file (from `GCFtocombinedfasta.py`)
  - Path for output files
- **Run Tools in Isolation:**

```
python3 phabox.py
...  # Run one tool at a time
```

  Tools should be executed sequentially to prevent system overload.

5.5 Step 4: Process Outputs (Outputs Directory)

- **Convert Outputs:** Navigate to `Outputs/Processing` and run tool-specific transformation scripts:

```
cd Outputs/Processing
python3 PhaBOX_output.py
```

- **Calculate Metrics:** To compute F1 score, precision, and recall:

```
cd Outputs/Metrics
python3 main.py
```

6 Supplementary Tables and Figures

6.1 Database Composition and Provenance

Ensuring reproducibility and comparability in metagenomic analyses depends heavily on accurately documenting the reference databases utilized by each classification tool. For HYMET, database selection is performed dynamically through Mash sketches, creating a tailored set of references per run. These sketches are publicly archived and version-controlled, facilitating exact reproducibility (Zenodo: doi:10.5281/zenodo.17428354). The resulting candidate genomes are combined into a per-run FASTA file, indexed by minimap2, and identified uniquely by the SHA1 hash of the candidate selection list.

Other benchmarking tools were configured either using a shared standard reference set or their officially distributed databases. Tools such as Kraken2, Bracken, Centrifuge and sourmash collection were provided with the same standardized corpus from RefSeq and GenBank, comprising 922 assemblies across viruses, bacteria, archaea and eukaryotes, totaling 1.22 Gbp. The taxonomic mappings were consistently regenerated from the NCBI taxonomic dumps to ensure uniformity across these tools.

Meanwhile, ganon2 currently references a smaller legacy subset, necessitating a planned upgrade to fully align with the standardized corpus. Other tools, including MetaPhlAn4, CAMITAX, phyloFlash, and ViWrap/geNomad, relied on their respective official database distributions. These proprietary datasets offer comprehensive reference materials specific to their operational methodologies, such as the CHOCOPhlAn SGB marker database for MetaPhlAn4 or SILVA SSU rRNA databases for phyloFlash. Detailed information, such as database sizes, exact

provenance, and special notes, have been recorded for precise tracking and future benchmarking clarity.

Tool	Database Source and Notes	Composition
**Shared Standard Databases**		
HYMET	Mash-selected RefSeq/GenBank assemblies; SHA1-keyed reproducibility.	922 assemblies; 50,178 contigs; 1.22 Gbp
Kraken2 + Bracken	NCBI RefSeq/GenBank (identical corpus as HYMET); standardized taxonomic dump.	∼60 GB index
Centrifuge	NCBI RefSeq/GenBank (identical corpus as HYMET); SeqID-TaxID mapping regenerated.	∼504 MB index
ganon2	Subset of NCBI RefSeq/GenBank (legacy subset).	42 MB; 1,000 contigs; 5 species
sourmash gather	MinHash signatures from RefSeq/GenBank corpus; consistent SeqID-TaxID mapping.	68 MB SBT signatures
**Official Tool-specific Databases**		
MetaPhlAn4	CHOCOPhlAn SGB vJun23; official release with no customization.	12 GB; ∼7.39M markers
CAMITAX	GTDB, SILVA, CheckM combined database; official upstream release.	>100 GB typical size
phyloFlash	SILVA SSU rRNA (release 138.1); official release with documented versioning.	∼16 GB
ViWrap (geNomad)	geNomad database v1.9; official release with documented version.	1.4 GB; various viral markers
SqueezeMeta	NCBI nr, SwissProt, NCBI taxdump; official build documented.	3.6 GB; protein and taxonomy data
MegaPath-Nano	RefSeq microbial/viral assemblies; build summary checksum logged.	781 MB; 18,916 assemblies
PhaBOX	Viral hallmark markers (v2.1); official release with checksum logged.	1.7 GB; 20,834 proteins
SnakeMAGs	GTDB-Tk reference (GTDB R214); official release with documented version.	∼30 GB

Table S2: Provenance and Database Specifications per Benchmarking Tool.

6.2 Per-domain benchmark performance

Tax. rank	Kingdom			Phylum			Class			Order			Family			Genus			Species		
Tool	P	R	F1	P	R	F1	P	R	F1	P	R	F1	P	R	F1	P	R	F1	P	R	F1
**HYMET**	**0.96**	**0.96**	**0.96**	**0.96**	**0.83**	**0.89**	**0.97**	**0.83**	**0.89**	**0.97**	**0.52**	**0.67**	**0.97**	**0.67**	**0.79**	**0.96**	**0.78**	**0.86**	**0.96**	**0.95**	**0.95**
BASTA	0.58	0.10	0.17	0.55	0.09	0.16	0.56	0.10	0.16	0.64	0.09	0.15	0.61	0.09	0.16	0.50	0.08	0.14	0.64	0.09	0.15
Camitax	-	-	-	-	-	-	-	-	-	-	-	-	-	-	-	-	-	-	-	-	-
MegaPath-Nano	-	-	-	-	-	-	-	-	-	-	-	-	-	-	-	0.52	0.28	0.36	0.45	0.27	0.34
SqueezeMeta	1.00	0.96	0.98	1.00	0.83	0.90	1.00	0.83	0.91	1.00	0.52	0.68	1.00	0.68	0.81	1.00	0.79	0.88	1.00	0.96	0.98
ViWrap	0.99	0.93	0.96	0.98	0.92	0.95	0.97	0.91	0.94	0.85	0.77	0.81	0.31	0.29	0.30	0.00	0.00	0.00	0.00	0.00	0.00
PhaBOX	1.00	0.79	0.88	1.00	0.77	0.87	1.00	0.78	0.87	0.00	0.00	0.00	0.98	0.71	0.82	0.95	0.72	0.82	-	-	-
SnakeMAGS	-	-	-	-	-	-	-	-	-	-	-	-	-	-	-	-	-	-	-	-	-
Tama	0.00	0.00	0.00	-	-	-	-	-	-	-	-	-	-	-	-	-	-	-	-	-	-
PhyloFlash	-	-	-	-	-	-	-	-	-	-	-	-	-	-	-	-	-	-	-	-	-

Table S3: Viral dataset performance metrics results.

Tax. rank	Kingdom			Phylum			Class			Order			Family			Genus			Species		
Tool	P	R	F1	P	R	F1	P	R	F1	P	R	F1	P	R	F1	P	R	F1	P	R	F1
**HYMET**	**1.00**	**1.00**	**1.00**	**1.00**	**1.00**	**1.00**	**1.00**	**1.00**	**1.00**	**1.00**	**1.00**	**1.00**	**1.00**	**1.00**	**1.00**	**1.00**	**1.00**	**1.00**	**0.97**	**0.97**	**0.97**
BASTA	0.41	0.30	0.45	0.40	0.30	0.34	0.40	0.30	0.34	0.40	0.29	0.34	0.28	0.21	0.24	0.15	0.11	0.13	0.02	0.01	0.02
Camitax	1.00	0.02	0.03	0.00	0.00	0.00	1.00	0.16	0.28	0.40	0.01	0.02	0.89	0.07	0.14	1.00	0.17	0.30	0.52	0.24	0.24
MegaPath-Nano	-	-	-	-	-	-	-	-	-	-	-	-	-	-	-	0.49	0.11	0.18	0.36	0.08	0.13
SqueezeMeta	1.00	1.00	1.00	1.00	1.00	1.00	1.00	1.00	1.00	1.00	1.00	1.00	1.00	1.00	1.00	1.00	1.00	1.00	1.00	1.00	1.00
ViWrap	0.00	0.00	0.00	0.00	0.00	0.00	0.00	0.00	0.00	0.00	0.00	0.00	0.00	0.00	0.00	0.00	0.00	0.00	0.00	0.00	0.00
PhaBOX	0.00	0.00	0.00	0.00	0.00	0.00	0.00	0.00	0.00	0.00	0.00	0.00	0.00	0.00	0.00	0.00	0.00	0.00	0.00	0.00	0.00
SnakeMAGS	1.00	0.07	0.12	0.40	0.03	0.05	0.67	0.04	0.08	1.00	0.07	0.12	0.93	0.06	0.11	0.73	0.05	0.09	0.38	0.02	0.04
Tama	1.00	0.35	0.52	-	-	-	-	-	-	-	-	-	-	-	-	-	-	-	-	-	-
PhyloFlash	0.98	0.37	0.53	0.01	0.00	0.01	0.87	0.33	0.47	0.36	0.13	0.29	0.75	0.28	0.41	0.84	0.32	0.46	0.14	0.05	0.08

Table S4: Archaea dataset performance metrics results.

Tax. rank	Kingdom			Phylum			Class			Order			Family			Genus			Species		
Tool	P	R	F1	P	R	F1	P	R	F1	P	R	F1	P	R	F1	P	R	F1	P	R	F1
**HYMET**	**1.00**	**1.00**	**1.00**	**0.99**	**0.99**	**0.99**	**0.99**	**0.99**	**0.99**	**0.98**	**0.98**	**0.98**	**0.98**	**0.98**	**0.98**	**0.95**	**0.95**	**0.95**	**0.79**	**0.79**	**0.79**
BASTA	0.89	0.00	0.01	0.86	0.00	0.01	0.76	0.00	0.01	0.54	0.00	0.01	0.42	0.00	0.01	0.25	0.00	0.01	0.09	0.00	0.01
Camitax	-	-	-	-	-	-	-	-	-	-	-	-	-	-	-	-	-	-	-	-	-
MegaPath-Nano	-	-	-	-	-	-	-	-	-	-	-	-	-	-	-	-	-	-	-	-	-
SqueezeMeta	1.00	0.00	0.00	1.00	0.00	0.00	1.00	0.00	0.00	1.00	0.00	0.00	1.00	0.00	0.00	1.00	0.00	0.00	1.00	0.00	0.00
ViWrap	0.00	0.00	0.00	0.00	0.00	0.00	0.00	0.00	0.00	0.00	0.00	0.00	0.00	0.00	0.00	0.00	0.00	0.00	0.00	0.00	0.00
PhaBOX	0.00	0.00	0.00	0.00	0.00	0.00	0.00	0.00	0.00	0.00	0.00	0.00	0.00	0.00	0.00	0.00	0.00	0.00	0.00	0.00	0.00
SnakeMAGS	-	-	-	-	-	-	-	-	-	-	-	-	-	-	-	-	-	-	-	-	-
Tama	1.00	0.70	0.83	-	-	-	-	-	-	-	-	-	-	-	-	-	-	-	-	-	-
PhyloFlash	0.99	0.71	0.83	0.05	0.04	0.05	0.84	0.60	0.70	0.65	0.47	0.54	0.90	0.65	0.75	0.66	0.47	0.55	0.27	0.19	0.22

Table S5: Bacteria dataset performance metrics results.

Tax. rank	Kingdom			Phylum			Class			Order			Family			Genus			Species		
Tool	P	R	F1	P	R	F1	P	R	F1	P	R	F1	P	R	F1	P	R	F1	P	R	F1
**HYMET**	**1.00**	**1.00**	**1.00**	**1.00**	**1.00**	**1.00**	**1.00**	**1.00**	**1.00**	**1.00**	**1.00**	**1.00**	**1.00**	**1.00**	**1.00**	**1.00**	**1.00**	**1.00**	**1.00**	**1.00**	**1.00**
BASTA	0.64	0.26	0.37	0.64	0.26	0.37	0.56	0.23	0.33	0.49	0.20	0.28	0.49	0.20	0.28	0.49	0.20	0.28	0.31	0.12	0.18
Camitax	0.00	0.00	0.00	0.00	0.00	0.00	0.00	0.00	0.00	0.00	0.00	0.00	0.00	0.00	0.00	0.00	0.00	0.00	0.00	0.00	0.00
MegaPath-Nano	-	-	-	-	-	-	-	-		-	-	-	-	-	-	0.72	0.29	0.41	0.36	0.14	0.20
SqueezeMeta	1.00	1.00	1.00	1.00	1.00	1.00	1.00	0.97	0.98	1.00	0.97	0.98	1.00	0.97	0.98	1.00	1.00	1.00	1.00	1.00	1.00
ViWrap	0.00	0.00	0.00	0.00	0.00	0.00	0.00	0.00	0.00	0.00	0.00	0.00	0.00	0.00	0.00	0.00	0.00	0.00	0.00	0.00	0.00
PhaBOX	0.00	0.00	0.00	0.00	0.00	0.00	0.00	0.00	0.00	0.00	0.00	0.00	0.00	0.00	0.00	0.00	0.00	0.00	0.00	0.00	0.00
SnakeMAGS	-	-	-	-	-	-	-	-	-	-	-	-	-	-	-	-	-	-	-	-	-
Tama	0.00	0.00	0.00	-	-	-	-	-	-	-	-	-	-	-	-	-	-	-	-	-	-
PhyloFlash	0.87	0.66	0.75	0.00	0.00	0.00	0.00	0.00	0.00	0.00	0.00	0.00	0.00	0.00	0.00	0.00	0.00	0.00	0.00	0.00	0.00

Table S6: Fungi dataset performance metrics results.

Tax. rank	Kingdom			Phylum			Class			Order			Family			Genus			Species		
Tool	P	R	F1	P	R	F1	P	R	F1	P	R	F1	P	R	F1	P	R	F1	P	R	F1
**HYMET**	**1.00**	**1.00**	**1.00**	**1.00**	**1.00**	**1.00**	**1.00**	**1.00**	**1.00**	**1.00**	**1.00**	**1.00**	**1.00**	**1.00**	**1.00**	**1.00**	**1.00**	**1.00**	**1.00**	**1.00**	**1.00**
BASTA	0.20	0.12	0.15	0.00	0.00	0.00	0.00	0.00	0.00	0.00	0.00	0.00	0.00	0.00	0.00	0.00	0.00	0.00	0.00	0.00	0.00
Camitax	0.00	0.00	0.00	0.00	0.00	0.00	0.00	0.00	0.00	0.00	0.00	0.00	0.00	0.00	0.00	0.00	0.00	0.00	0.00	0.00	0.00
MegaPath-Nano	-	-	-	-	-	-	-	-	-	-	-	-	-	-	-	0.89	0.67	0.76	0.67	0.50	0.57
SqueezeMeta	1.00	1.00	1.00	1.00	0.92	0.96	1.00	0.75	0.86	1.00	1.00	1.00	1.00	1.00	1.00	1.00	1.00	1.00	1.00	1.00	1.00
ViWrap	0.00	0.00	0.00	0.00	0.00	0.00	0.00	0.00	0.00	0.00	0.00	0.00	0.00	0.00	0.00	0.00	0.00	0.00	0.00	0.00	0.00
PhaBOX	0.00	0.00	0.00	0.00	0.00	0.00	0.00	0.00	0.00	0.00	0.00	0.00	0.00	0.00	0.00	0.00	0.00	0.00	0.00	0.00	0.00
SnakeMAGS	-	-	-	-	-	-	-	-	-	-	-	-	-	-	-	-	-	-	-	-	-
Tama	0.00	0.00	0.00	-	-	-	-	-	-	-	-	-	-	-	-	-	-	-	-	-	-
PhyloFlash	0.71	0.42	0.53	0.00	0.00	0.00	0.00	0.00	0.00	0.00	0.00	0.00	0.00	0.00	0.00	0.00	0.00	0.00	0.00	0.00	0.00

Table S7: Protozoa dataset performance metrics results.

Tax. rank	Kingdom			Phylum			Class			Order			Family			Genus			Species		
Tool	P	R	F1	P	R	F1	P	R	F1	P	R	F1	P	R	F1	P	R	F1	P	R	F1
**HYMET**	**1.00**	**1.00**	**1.00**	**1.00**	**1.00**	**1.00**	**1.00**	**1.00**	**1.00**	**1.00**	**1.00**	**1.00**	**0.94**	**0.94**	**0.94**	**0.94**	**0.94**	**0.94**	**0.94**	**0.94**	**0.94**
BASTA	0.88	0.53	0.67	0.73	0.44	0.55	0.69	0.42	0.52	0.19	0.14	0.16	0.19	0.14	0.16	0.19	0.14	0.16	0.00	0.00	0.00
Camitax	-	-	-	-	-	-	-	-	-	-	-	-	-	-	-	-	-	-	-	-	-
MegaPath-Nano	-	-	-	-	-	-	-	-	-	-	-	-	-	-	-	0.00	0.00	0.00	0.00	0.00	0.00
SqueezeMeta	1.00	1.00	1.00	1.00	1.00	1.00	1.00	1.00	1.00	1.00	1.00	1.00	1.00	1.00	1.00	1.00	1.00	1.00	1.00	1.00	1.00
ViWrap	0.00	0.00	0.00	0.00	0.00	0.00	0.00	0.00	0.00	0.00	0.00	0.00	0.00	0.00	0.00	0.00	0.00	0.00	0.00	0.00	0.00
PhaBOX	0.00	0.00	0.00	0.00	0.00	0.00	0.00	0.00	0.00	0.00	0.00	0.00	0.00	0.00	0.00	0.00	0.00	0.00	0.00	0.00	0.00
SnakeMAGS	-	-	-	-	-	-	-	-	-	-	-	-	-	-	-	-	-	-	-	-	-
Tama	0.00	0.00	0.00	-	-	-	-	-	-	-	-	-	-	-	-	-	-	-	-	-	-
PhyloFlash	0.98	0.97	0.97	0.00	0.00	0.00	0.00	0.00	0.00	0.00	0.00	0.00	0.00	0.00	0.00	0.00	0.00	0.00	0.00	0.00	0.00

Table S8: Plant dataset performance metrics results.

Tax. rank	Kingdom			Phylum			Class			Order			Family			Genus			Species		
Tool	P	R	F1	P	R	F1	P	R	F1	P	R	F1	P	R	F1	P	R	F1	P	R	F1
**HYMET**	**1.00**	**1.00**	**1.00**	**1.00**	**1.00**	**1.00**	**1.00**	**1.00**	**1.00**	**1.00**	**1.00**	**1.00**	**1.00**	**1.00**	**1.00**	**0.98**	**0.98**	**0.98**	**0.95**	**0.95**	**0.95**
BASTA	0.50	0.17	0.25	0.00	0.00	0.00	0.00	0.00	0.00	0.00	0.00	0.00	0.00	0.00	0.00	0.00	0.00	0.00	0.00	0.00	0.00
Camitax	-	-	-	-	-	-	-	-	-	-	-	-	-	-	-	-	-	-	-	-	-
MegaPath-Nano	-	-	-	-	-	-	-	-	-	-	-	-	-	-	-	0.00	0.00	0.00	0.00	0.00	0.00
SqueezeMeta	1.00	1.00	1.00	1.00	0.98	0.99	1.00	1.00	1.00	1.00	0.98	0.99	1.00	0.98	0.99	1.00	1.00	1.00	1.00	1.00	1.00
ViWrap	0.00	0.00	0.00	0.00	0.00	0.00	0.00	0.00	0.00	0.00	0.00	0.00	0.00	0.00	0.00	0.00	0.00	0.00	0.00	0.00	0.00
PhaBOX	0.00	0.00	0.00	0.00	0.00	0.00	0.00	0.00	0.00	0.00	0.00	0.00	0.00	0.00	0.00	0.00	0.00	0.00	0.00	0.00	0.00
SnakeMAGS	-	-	-	-	-	-	-	-	-	-	-	-	-	-	-	-	-	-	-	-	-
Tama	0.00	0.00	0.00	-	-	-	-	-	-	-	-	-	-	-	-	-	-	-	-	-	-
PhyloFlash	1.00	0.99	0.99	0.00	0.00	0.00	0.00	0.00	0.00	0.00	0.00	0.00	0.00	0.00	0.00	0.00	0.00	0.00	0.00	0.00	0.00

Table S9: Invertebrate dataset performance metrics results.

Tax. rank	Kingdom			Phylum			Class			Order			Family			Genus			Species		
Tool	P	R	F1	P	R	F1	P	R	F1	P	R	F1	P	R	F1	P	R	F1	P	R	F1
**HYMET**	**1.00**	**1.00**	**1.00**	**1.00**	**1.00**	**1.00**	**1.00**	**1.00**	**1.00**	**0.99**	**0.99**	**0.99**	**0.97**	**0.97**	**0.97**	**0.95**	**0.95**	**0.95**	**0.91**	**0.91**	**0.91**
BASTA	0.48	0.27	0.35	0.45	0.25	0.33	0.38	0.22	0.28	0.00	0.00	0.00	0.00	0.00	0.00	0.00	0.00	0.00	0.00	0.00	0.00
Camitax	-	-	-	-	-	-	-	-	-	-	-	-	-	-	-	-	-	-	-	-	-
MegaPath-Nano	-	-	-	-	-	-	-	-	-	-	-	-	-	-	-	0.00	0.00	0.00	0.00	0.00	0.00
SqueezeMeta	1.00	1.00	1.00	1.00	1.00	1.00	1.00	1.00	1.00	1.00	1.00	1.00	1.00	1.00	1.00	1.00	1.00	1.00	1.00	1.00	1.00
ViWrap	0.00	0.00	0.00	0.00	0.00	0.00	0.00	0.00	0.00	0.00	0.00	0.00	0.00	0.00	0.00	0.00	0.00	0.00	0.00	0.00	0.00
PhaBOX	0.00	0.00	0.00	0.00	0.00	0.00	0.00	0.00	0.00	0.00	0.00	0.00	0.00	0.00	0.00	0.00	0.00	0.00	0.00	0.00	0.00
SnakeMAGS	-	-	-	-	-	-	-	-	-	-	-	-	-	-	-	-	-	-	-	-	-
Tama	0.00	0.00	0.00	-	-	-	-	-	-	-	-	-	-	-	-	-	-	-	-	-	-
PhyloFlash	1.00	0.95	0.98	0.00	0.00	0.00	0.00	0.00	0.00	0.00	0.00	0.00	0.00	0.00	0.00	0.00	0.00	0.00	0.00	0.00	0.00

Table S10: Vertebrate dataset performance metrics results.

6.3 HYMET's performance under mutation rates

Tax. rank	Kingdom			Phylum			Class			Order			Family			Genus			Species		
Tax. group	P	R	F1	P	R	F1	P	R	F1	P	R	F1	P	R	F1	P	R	F1	P	R	F1
Archaea	1.00	1.00	1.00	1.00	1.00	1.00	1.00	1.00	1.00	1.00	1.00	1.00	1.00	1.00	1.00	1.00	1.00	1.00	0.97	0.97	0.97
Fungi	1.00	1.00	1.00	1.00	1.00	1.00	1.00	1.00	1.00	1.00	1.00	1.00	1.00	1.00	1.00	1.00	1.00	1.00	1.00	1.00	1.00
Protozoa	1.00	1.00	1.00	1.00	1.00	1.00	1.00	1.00	1.00	1.00	1.00	1.00	1.00	1.00	1.00	1.00	1.00	1.00	1.00	1.00	1.00
Virus	0.96	0.96	0.96	0.96	0.83	0.89	0.97	0.83	0.89	0.97	0.52	0.67	0.97	0.67	0.79	0.96	0.78	0.86	0.96	0.95	0.95
Plant	1.00	1.00	1.00	1.00	1.00	1.00	1.00	1.00	1.00	1.00	1.00	1.00	0.94	0.94	0.94	0.94	0.94	0.94	0.83	0.83	0.83
Vertebrate Mammal	1.00	1.00	1.00	1.00	1.00	1.00	1.00	1.00	1.00	1.00	1.00	1.00	0.96	0.96	0.96	0.91	0.91	0.91	0.83	0.83	0.83
Vertebrate Other	1.00	1.00	1.00	1.00	1.00	1.00	1.00	1.00	1.00	0.98	0.98	0.98	0.98	0.98	0.98	0.98	0.98	0.98	0.98	0.98	0.98
Invertebrate	1.00	1.00	1.00	1.00	1.00	1.00	1.00	1.00	1.00	1.00	1.00	1.00	1.00	1.00	1.00	0.98	0.98	0.98	0.95	0.95	0.95
Bacteria	1.00	1.00	1.00	0.99	0.99	0.99	0.99	0.99	0.99	0.98	0.98	0.98	0.98	0.98	0.98	0.95	0.95	0.95	0.79	0.79	0.79

Table S11: HYMET's performance under 0% mutation rate.

Tax. rank	Kingdom			Phylum			Class			Order			Family			Genus			Species		
Tax. group	P	R	F1	P	R	F1	P	R	F1	P	R	F1	P	R	F1	P	R	F1	P	R	F1
Archaea	1.00	1.00	1.00	1.00	1.00	1.00	1.00	1.00	1.00	1.00	1.00	1.00	1.00	1.00	1.00	0.99	0.99	0.99	0.94	0.94	0.94
Fungi	1.00	1.00	1.00	1.00	1.00	1.00	1.00	0.97	0.98	1.00	0.97	0.98	1.00	0.95	0.98	1.00	0.98	0.99	1.00	0.98	0.99
Protozoa	1.00	1.00	1.00	1.00	0.92	0.96	1.00	0.75	0.86	1.00	0.92	0.96	1.00	0.92	0.96	1.00	0.92	0.96	1.00	0.92	0.96
Virus	0.96	0.96	0.96	0.96	0.83	0.89	0.97	0.83	0.89	0.97	0.52	0.67	0.97	0.65	0.78	0.96	0.77	0.86	0.97	0.91	0.94
Plant	1.00	1.00	1.00	1.00	1.00	1.00	1.00	1.00	1.00	1.00	1.00	1.00	0.94	0.94	0.94	0.94	0.94	0.94	0.83	0.83	0.83
Vertebrate Mammal	1.00	1.00	1.00	1.00	1.00	1.00	1.00	1.00	1.00	1.00	1.00	1.00	0.96	0.96	0.96	0.91	0.91	0.91	0.83	0.83	0.83
Vertebrate Other	1.00	1.00	1.00	1.00	1.00	1.00	1.00	1.00	1.00	0.98	0.98	0.98	0.98	0.98	0.98	0.98	0.98	0.98	0.98	0.98	0.98
Invertebrate	1.00	1.00	1.00	1.00	1.00	1.00	1.00	1.00	1.00	1.00	1.00	1.00	1.00	1.00	1.00	0.98	0.98	0.98	0.95	0.95	0.95
Bacteria	1.00	1.00	1.00	0.99	0.99	0.99	0.99	0.99	0.99	0.98	0.98	0.98	0.98	0.98	0.98	0.95	0.95	0.95	0.79	0.79	0.79

Table S12: HYMET's performance under 2% mutation rate.

Tax. rank	Kingdom			Phylum			Class			Order			Family			Genus			Species		
Tax. group	P	R	F1	P	R	F1	P	R	F1	P	R	F1	P	R	F1	P	R	F1	P	R	F1
Archaea	1.00	1.00	1.00	1.00	1.00	1.00	1.00	1.00	1.00	1.00	1.00	1.00	1.00	1.00	1.00	1.00	1.00	1.00	0.92	0.92	0.92
Fungi	1.00	1.00	1.00	1.00	1.00	1.00	1.00	0.97	0.98	1.00	0.97	0.98	1.00	0.95	0.98	1.00	0.98	0.99	1.00	0.98	0.99
Protozoa	1.00	1.00	1.00	1.00	0.92	0.96	1.00	0.75	0.86	1.00	0.75	0.86	1.00	0.75	0.86	1.00	0.75	0.86	1.00	0.75	0.86
Virus	0.96	0.96	0.96	0.96	0.83	0.89	0.97	0.83	0.89	0.97	0.52	0.67	0.97	0.64	0.77	0.96	0.76	0.85	0.97	0.83	0.89
Plant	1.00	1.00	1.00	1.00	1.00	1.00	1.00	1.00	1.00	1.00	1.00	1.00	0.94	0.94	0.94	0.94	0.94	0.94	0.83	0.83	0.83
Vertebrate Mammal	1.00	1.00	1.00	1.00	1.00	1.00	1.00	1.00	1.00	0.96	0.96	0.96	0.91	0.91	0.91	0.83	0.83	0.83	0.78	0.78	0.78
Vertebrate Other	1.00	1.00	1.00	1.00	1.00	1.00	1.00	1.00	1.00	0.98	0.98	0.98	0.98	0.98	0.98	0.98	0.98	0.98	0.98	0.98	0.98
Invertebrate	1.00	1.00	1.00	1.00	1.00	1.00	1.00	1.00	1.00	1.00	1.00	1.00	1.00	1.00	1.00	0.98	0.98	0.98	0.95	0.95	0.95
Bacteria	0.99	0.99	0.99	0.97	0.97	0.97	0.96	0.96	0.96	0.95	0.94	0.95	0.94	0.93	0.94	0.91	0.91	0.91	0.76	0.76	0.76

Table S13: HYMET's performance under 5% mutation rate.

Tax. rank	Kingdom			Phylum			Class			Order			Family			Genus			Species		
Tax. group	P	R	F1	P	R	F1	P	R	F1	P	R	F1	P	R	F1	P	R	F1	P	R	F1
Archaea	1.00	1.00	1.00	1.00	1.00	1.00	1.00	1.00	1.00	1.00	1.00	1.00	1.00	1.00	1.00	0.99	0.99	0.99	0.90	0.90	0.90
Fungi	1.00	1.00	1.00	1.00	1.00	1.00	1.00	0.97	0.98	1.00	0.97	0.98	1.00	0.95	0.98	1.00	0.97	0.98	1.00	0.97	0.98
Protozoa	1.00	1.00	1.00	1.00	0.92	0.96	1.00	0.75	0.86	1.00	0.75	0.86	1.00	0.75	0.86	1.00	0.75	0.86	1.00	0.75	0.86
Virus	0.96	0.96	0.96	0.96	0.83	0.89	0.97	0.83	0.89	0.97	0.52	0.67	0.97	0.64	0.77	0.96	0.76	0.85	0.97	0.83	0.89
Plant	1.00	1.00	1.00	1.00	1.00	1.00	1.00	1.00	1.00	0.99	0.99	0.99	0.94	0.94	0.94	0.94	0.94	0.94	0.83	0.83	0.83
Vertebrate Mammal	1.00	1.00	1.00	1.00	1.00	1.00	1.00	1.00	1.00	0.95	0.95	0.95	0.83	0.83	0.83	0.78	0.78	0.78	0.78	0.78	0.78
Vertebrate Other	1.00	1.00	1.00	1.00	1.00	1.00	0.95	0.95	0.95	0.95	0.86	0.90	0.95	0.86	0.90	0.88	0.86	0.87	0.88	0.86	0.87
Invertebrate	1.00	1.00	1.00	1.00	1.00	1.00	0.98	0.98	0.98	0.98	0.98	0.98	0.98	0.98	0.98	0.95	0.95	0.95	0.95	0.95	0.95
Bacteria	0.99	0.99	0.99	0.97	0.97	0.97	0.96	0.96	0.96	0.95	0.94	0.95	0.94	0.93	0.94	0.91	0.91	0.91	0.76	0.76	0.76

Table S14: HYMET's performance under 10% mutation rate.

Tax. rank	Kingdom			Phylum			Class			Order			Family			Genus			Species		
Tax. group	P	R	F1	P	R	F1	P	R	F1	P	R	F1	P	R	F1	P	R	F1	P	R	F1
Archaea	1.00	1.00	1.00	1.00	1.00	1.00	1.00	1.00	1.00	1.00	1.00	1.00	1.00	1.00	1.00	1.00	1.00	1.00	0.90	0.90	0.90
Fungi	1.00	1.00	1.00	1.00	1.00	1.00	1.00	0.97	0.98	1.00	0.97	0.98	1.00	0.95	0.98	1.00	0.97	0.98	1.00	0.97	0.98
Protozoa	1.00	1.00	1.00	1.00	0.92	0.96	1.00	0.75	0.86	1.00	0.75	0.86	1.00	0.75	0.86	1.00	0.75	0.86	1.00	0.75	0.86
Virus	0.96	0.96	0.96	0.96	0.83	0.89	0.97	0.83	0.89	0.97	0.52	0.67	0.97	0.62	0.76	0.96	0.73	0.83	0.96	0.77	0.86
Plant	1.00	1.00	1.00	1.00	1.00	1.00	1.00	1.00	1.00	0.99	0.99	0.99	0.94	0.94	0.94	0.94	0.94	0.94	0.83	0.83	0.83
Vertebrate Mammal	1.00	1.00	1.00	1.00	1.00	1.00	1.00	1.00	1.00	0.95	0.95	0.95	0.83	0.83	0.83	0.78	0.78	0.78	0.78	0.78	0.78
Vertebrate Other	1.00	1.00	1.00	1.00	1.00	1.00	0.95	0.86	0.90	0.88	0.86	0.87	0.88	0.86	0.87	0.86	0.84	0.85	0.86	0.84	0.85
Invertebrate	1.00	1.00	1.00	0.98	0.98	0.98	0.95	0.95	0.95	0.95	0.95	0.95	0.91	0.91	0.91	0.91	0.91	0.91	0.91	0.91	0.91
Bacteria	0.99	0.91	0.95	0.97	0.89	0.93	0.96	0.88	0.92	0.95	0.87	0.91	0.94	0.86	0.89	0.89	0.75	0.81	0.71	0.60	0.65

Table S15: HYMET's performance under 15% mutation rate.

Tax. rank	Kingdom			Phylum			Class			Order			Family			Genus			Species		
Tax. group	P	R	F1	P	R	F1	P	R	F1	P	R	F1	P	R	F1	P	R	F1	P	R	F1
Archaea	1.00	1.00	1.00	1.00	1.00	1.00	1.00	1.00	1.00	1.00	1.00	1.00	1.00	1.00	1.00	1.00	1.00	1.00	0.95	0.95	0.95
Fungi	1.00	1.00	1.00	1.00	1.00	1.00	1.00	0.97	0.98	1.00	0.97	0.98	1.00	0.95	0.98	1.00	0.97	0.98	0.93	0.90	0.92
Protozoa	1.00	1.00	1.00	1.00	0.92	0.96	1.00	0.67	0.80	1.00	0.67	0.80	1.00	0.67	0.80	1.00	0.67	0.80	1.00	0.67	0.80
Virus	0.96	0.95	0.96	0.96	0.83	0.89	0.97	0.83	0.89	0.97	0.52	0.67	0.97	0.59	0.74	0.96	0.64	0.77	0.96	0.66	0.78
Plant	1.00	1.00	1.00	1.00	1.00	1.00	1.00	1.00	1.00	0.99	0.99	0.99	0.94	0.94	0.94	0.94	0.94	0.94	0.83	0.83	0.83
Vertebrate Mammal	1.00	1.00	1.00	1.00	1.00	1.00	1.00	1.00	1.00	0.95	0.91	0.93	0.70	0.70	0.70	0.70	0.70	0.70	0.70	0.70	0.70
Vertebrate Other	1.00	1.00	1.00	1.00	1.00	1.00	0.95	0.86	0.90	0.88	0.86	0.87	0.88	0.86	0.87	0.83	0.81	0.82	0.83	0.81	0.82
Invertebrate	1.00	1.00	1.00	0.95	0.95	0.95	0.93	0.93	0.93	0.93	0.93	0.93	0.91	0.91	0.91	0.91	0.91	0.91	0.91	0.91	0.91
Bacteria	0.98	0.84	0.91	0.95	0.81	0.88	0.94	0.81	0.87	0.94	0.79	0.86	0.93	0.78	0.85	0.89	0.75	0.81	0.71	0.60	0.65

Table S16: HYMET's performance under 20% mutation rate.

Tax. rank	Kingdom			Phylum			Class			Order			Family			Genus			Species		
Tax. group	P	R	F1	P	R	F1	P	R	F1	P	R	F1	P	R	F1	P	R	F1	P	R	F1
Archaea	1.00	0.98	0.99	1.00	0.98	0.99	1.00	0.98	0.99	1.00	0.98	0.99	1.00	0.98	0.99	1.00	0.98	0.99	0.93	0.91	0.92
Fungi	1.00	1.00	1.00	1.00	1.00	1.00	1.00	0.97	0.98	1.00	0.97	0.98	1.00	0.95	0.98	1.00	0.97	0.98	0.95	0.92	0.94
Protozoa	1.00	1.00	1.00	1.00	0.92	0.96	1.00	0.67	0.80	1.00	0.67	0.80	1.00	0.67	0.80	1.00	0.67	0.80	1.00	0.67	0.80
Virus	0.96	0.67	0.79	0.96	0.61	0.75	0.96	0.61	0.75	0.96	0.30	0.46	0.96	0.31	0.47	0.97	0.30	0.46	0.97	0.30	0.46
Plant	1.00	1.00	1.00	1.00	1.00	1.00	1.00	1.00	1.00	0.94	0.94	0.94	0.94	0.94	0.94	0.89	0.89	0.89	0.83	0.83	0.83
Vertebrate Mammal	1.00	1.00	1.00	1.00	1.00	1.00	1.00	1.00	1.00	0.95	0.91	0.93	0.70	0.70	0.70	0.70	0.70	0.70	0.70	0.70	0.70
Vertebrate Other	1.00	1.00	1.00	1.00	1.00	1.00	0.95	0.86	0.90	0.88	0.86	0.87	0.88	0.86	0.87	0.83	0.81	0.82	0.83	0.81	0.82
Invertebrate	1.00	1.00	1.00	0.95	0.95	0.95	0.93	0.93	0.93	0.91	0.91	0.91	0.88	0.88	0.88	0.88	0.88	0.88	0.88	0.88	0.88
Bacteria	0.98	0.84	0.91	0.95	0.81	0.88	0.94	0.81	0.87	0.94	0.79	0.86	0.93	0.78	0.85	0.89	0.75	0.81	0.71	0.60	0.65

Table S17: HYMET's performance under 30% mutation rate.

6.4 Per-dataset CAMI benchmark results

Dataset	Tool	Avg F1 (%)	Genus F1 (%)	Species F1 (%)
CAMI I LC	HYMET	88.57	80.00	60.00
	MetaPhlAn 4	78.29	87.50	75.00
	Kraken 2	80.46	61.54	46.15
	TAMA	71.02	40.00	40.00
	MegaPath-Nano	86.76	76.92	61.54
CAMI I MC	HYMET	74.98	76.92	55.56
	MetaPhlAn 4	73.26	77.78	75.00
	Kraken 2	68.39	71.43	44.44
	TAMA	74.30	71.43	52.63
	MegaPath-Nano	67.42	54.55	25.00
CAMI I HC	HYMET	83.14	86.96	52.94
	MetaPhlAn 4	90.47	100.00	100.00
	Kraken 2	85.87	78.26	78.26
	TAMA	61.56	63.16	60.00
	MegaPath-Nano	74.50	76.19	25.00
CAMI II Mouse	HYMET	83.27	72.73	60.87
	MetaPhlAn 4	81.43	81.48	64.29
	Kraken 2	85.30	80.00	61.54
	TAMA	72.01	52.63	40.00
	MegaPath-Nano	75.44	57.14	25.00
CAMI II Marine	HYMET	92.61	90.00	78.26
	MetaPhlAn 4	78.67	76.19	81.82
	Kraken 2	82.54	77.78	55.56
	TAMA	89.18	70.59	73.68
	MegaPath-Nano	72.48	66.67	25.00
CAMI II Strain	HYMET	83.20	66.67	50.00
	MetaPhlAn 4	80.86	62.50	77.78
	Kraken 2	86.28	66.67	70.59
	TAMA	72.31	46.15	53.33
	MegaPath-Nano	81.55	53.33	37.50
CAMI Ref	HYMET	81.49	64.00	63.64
	MetaPhlAn 4	68.85	45.83	46.75
	Kraken 2	48.48	38.10	27.12
	TAMA	71.41	43.24	36.00
	MegaPath-Nano	63.55	51.85	26.32

Table S18: Per-dataset evaluation summary for the five principal tools across the seven CAMI assembly datasets (canonical multi-tool suite; tight candidate cap). Avg F1 is the arithmetic mean of F1 scores across all seven taxonomic ranks (superkingdom to species). Genus and species F1 are reported separately.

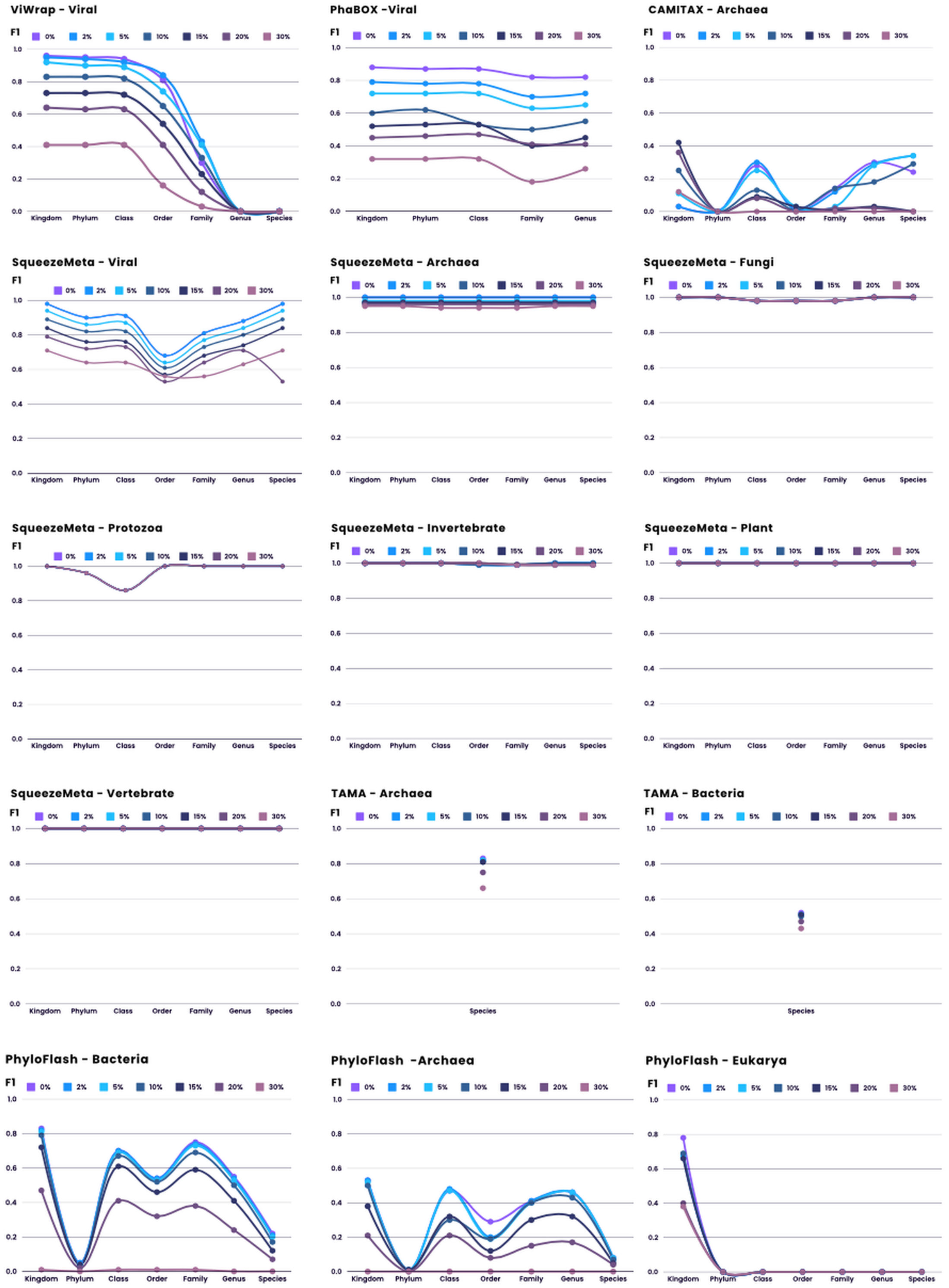

Figure S1: Performance of metagenomics classification tools under increasing mutation rates (0%–30%), across taxonomic levels (kingdom to species) for different biological domains (Part 1). Each panel shows one tool–domain combination; line colour indicates the mutation rate. Tools shown: HYMET, Kraken2, MetaPhlAn4, PhyloFlash, SqueezeMeta, and SnakeMAGs.

6.5 State-of-the-art performance under mutation rates

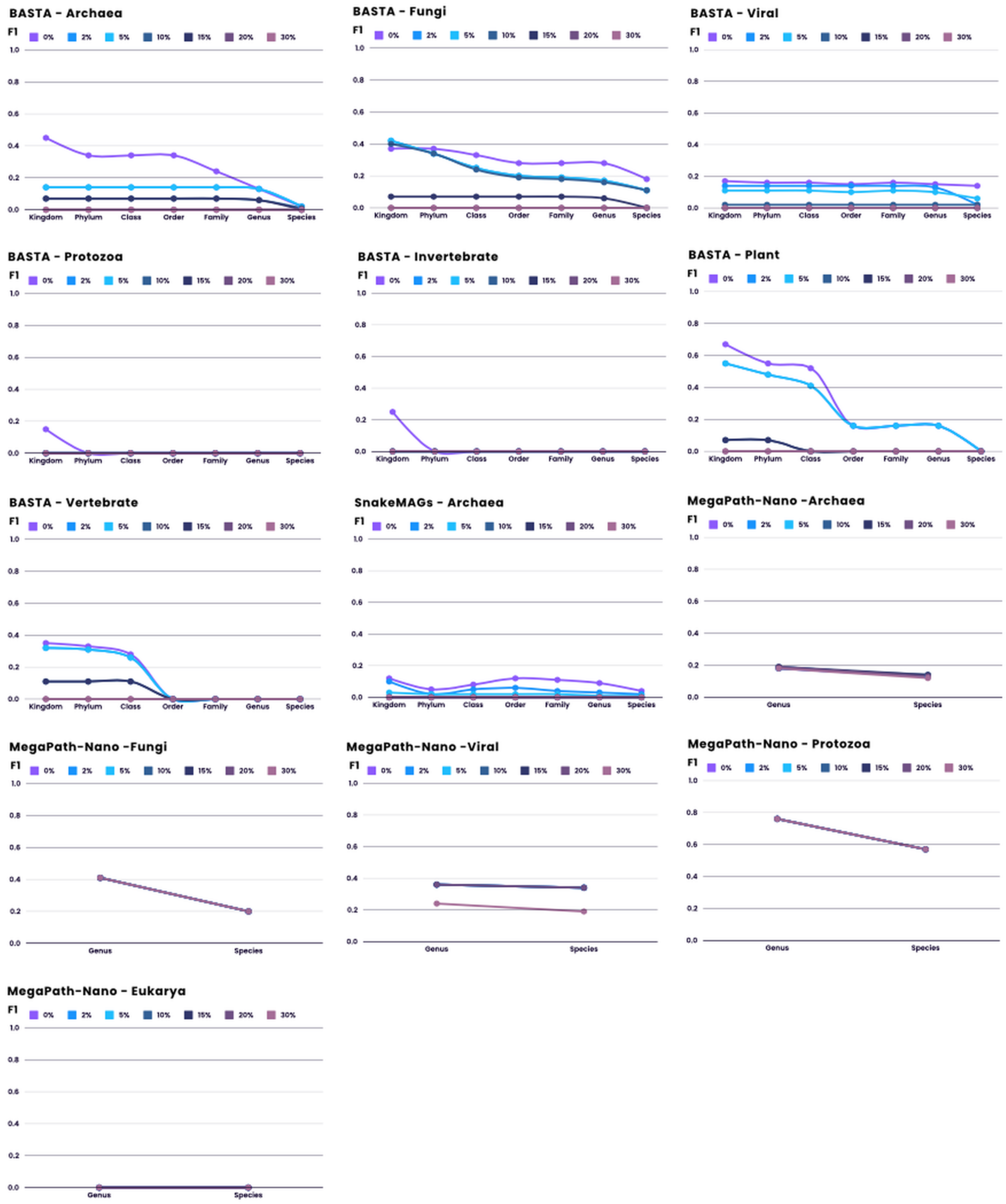

Figure S2: Performance of metagenomics classification tools under increasing mutation rates (0%–30%), across taxonomic levels (kingdom to species) for different biological domains (Part 2, continued from Figure S1). Tools shown: BASTA, SnakeMAGs (Archaea), and MegaPath-Nano.

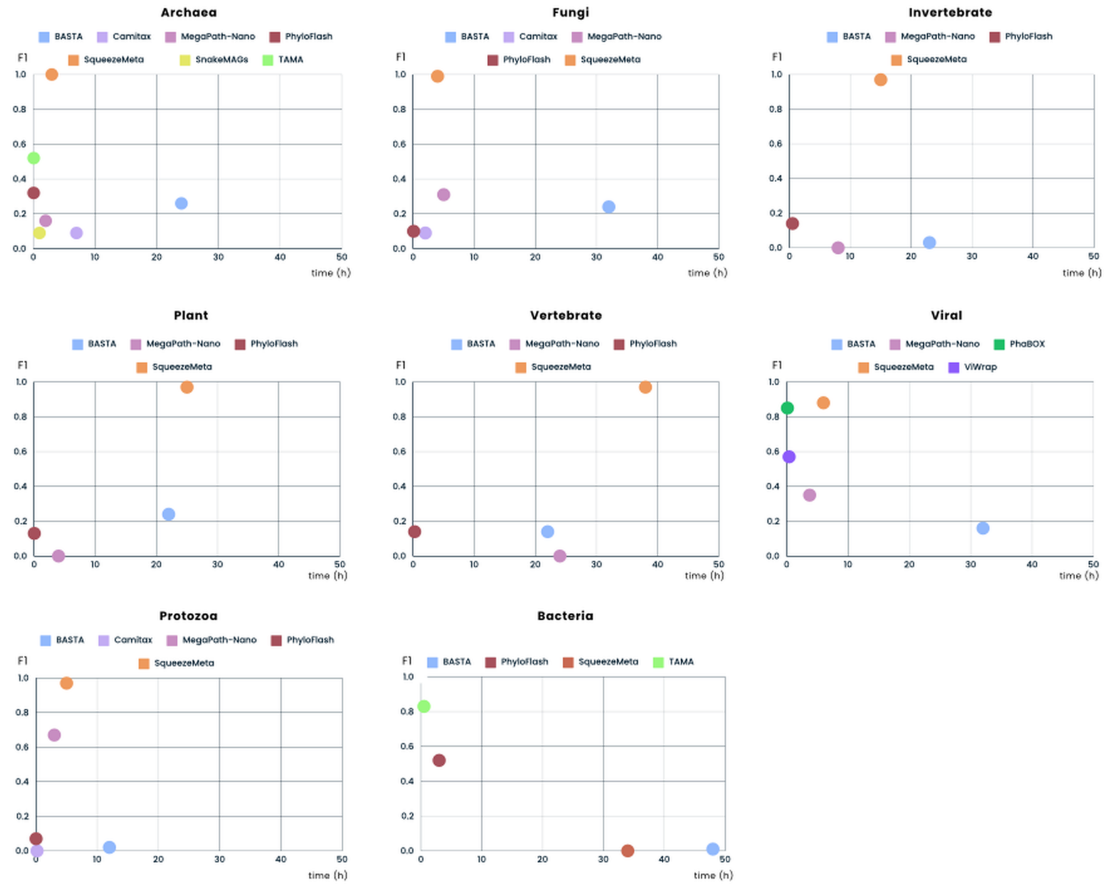

Figure S3: Relationship between execution time (hours) and F1 score (0.0–1.0) for various taxonomic classification tools at the species level. Each panel corresponds to a specific biological domain; each point represents one tool.

6.6 Benchmark and case-study figures

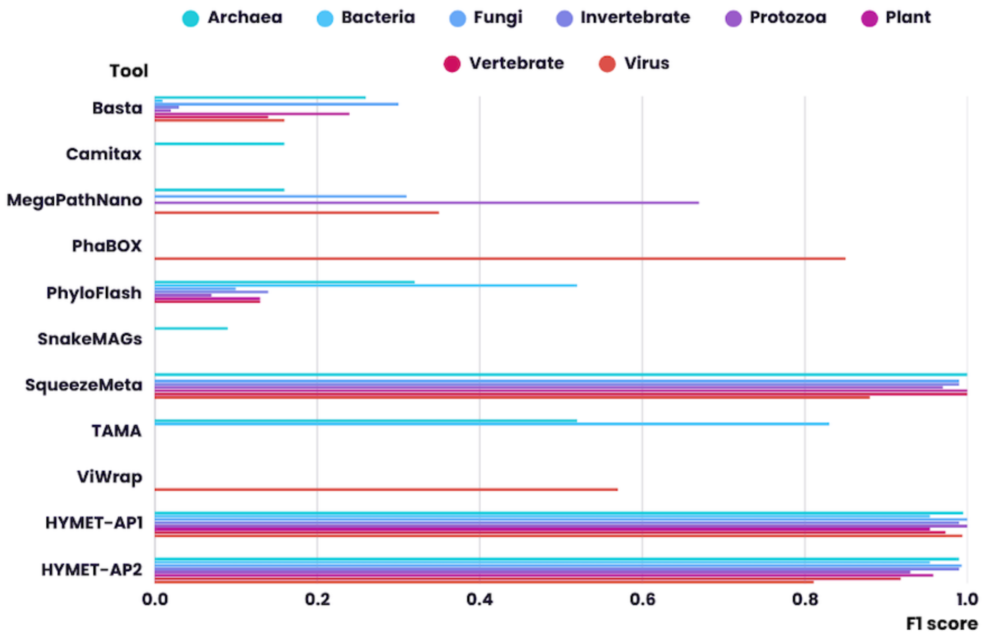

Figure S4: F1 scores achieved by various taxonomic classification tools, including both state-of-the-art tools and HYMET, across different taxonomic groups at 0% mutation rate. The x-axis represents the F1 scores (0–1), while the y-axis lists the evaluated tools. Each bar is colour-coded to indicate the corresponding taxonomic group.

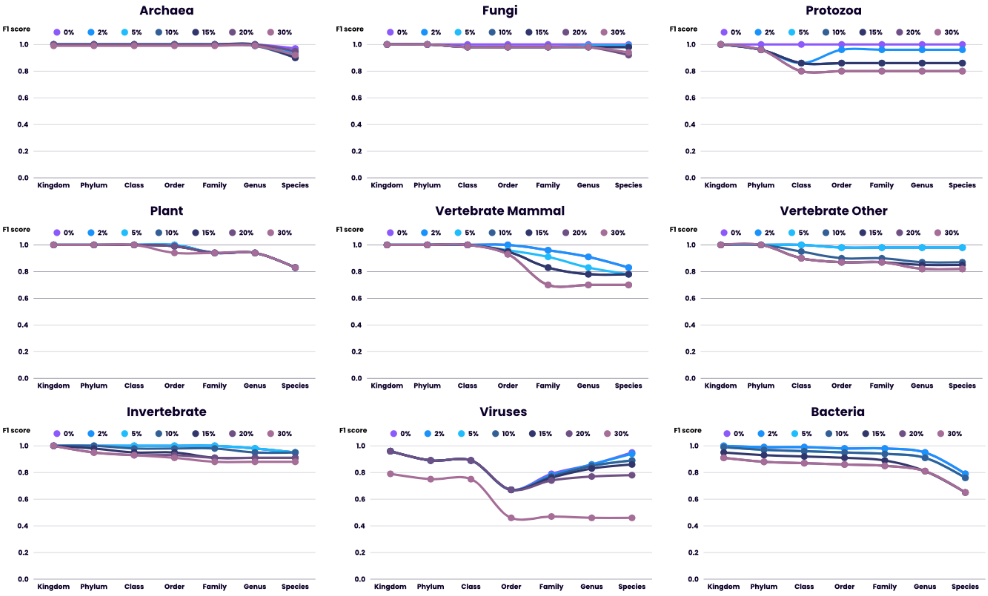

Figure S5: HYMET per-domain mutation resilience. Performance across taxonomic levels (kingdom to species) for each of nine biological groups as mutation rates increase from 0% to 30%. Each panel shows one domain; each curve corresponds to a specific mutation rate.

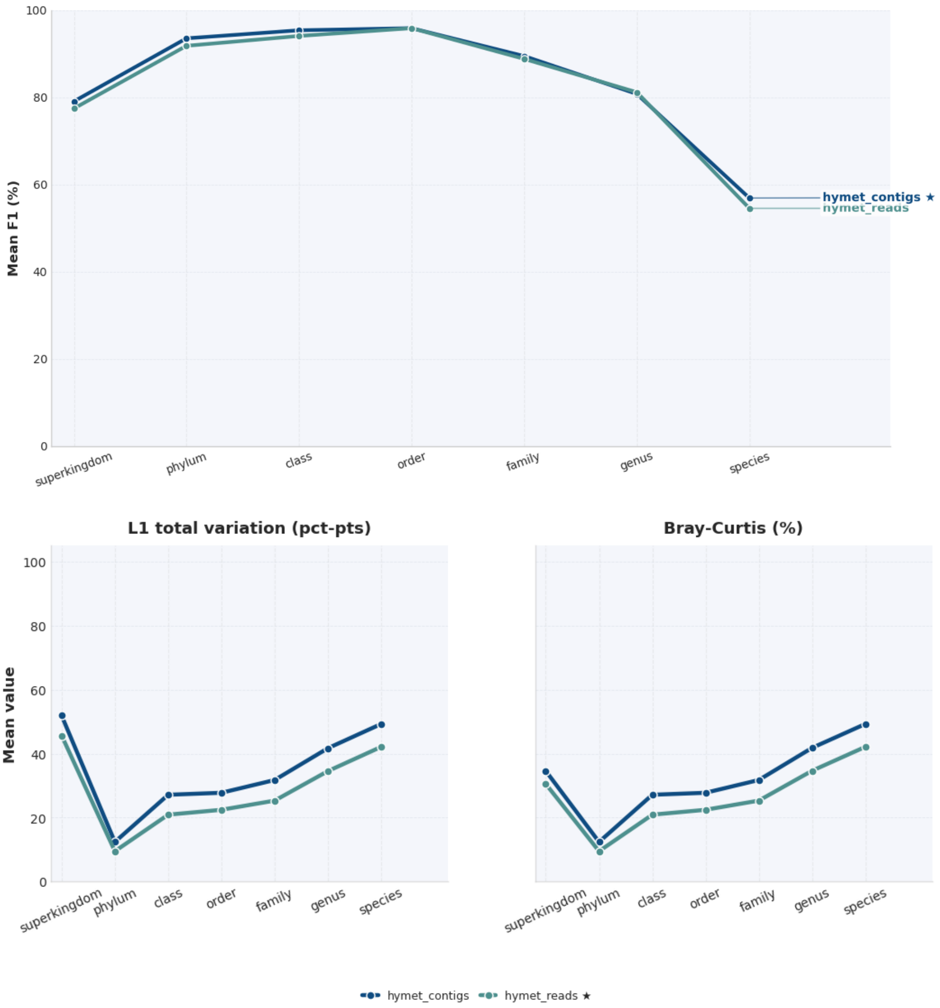

Figure S6: Comparison of HYMET's contig and synthetic-read workflows across CAMI assembly datasets. Top panel: Mean F1 scores across taxonomic ranks (superkingdom to species). Bottom panel: Mean abundance distances, with L1 total variation (solid lines) and Bray–Curtis dissimilarity (dashed lines).

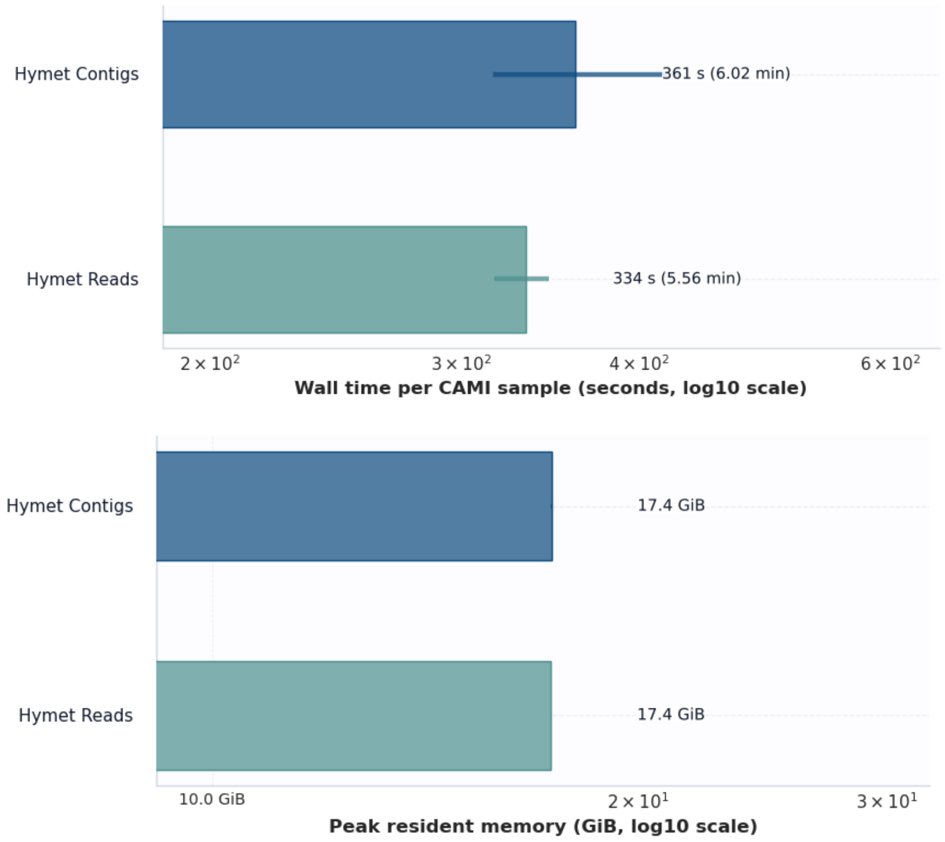

Figure S7: Resource usage comparison between HYMET contig and read workflows, measured across seven CAMI assembly datasets (HYMET-only suite; expanded candidate budget). Top: wall-clock execution time (seconds). Bottom: peak memory usage (GB).

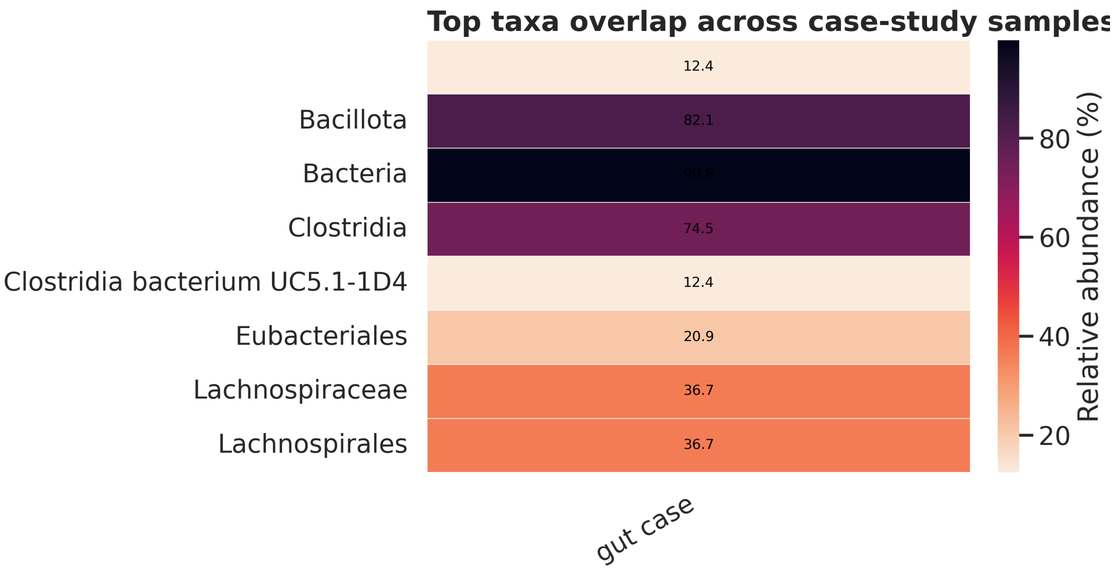

Figure S8: Abundance heatmap for the human gut case-study assembly. Colour intensity encodes relative abundance across taxonomic ranks; darker cells indicate higher contributions.

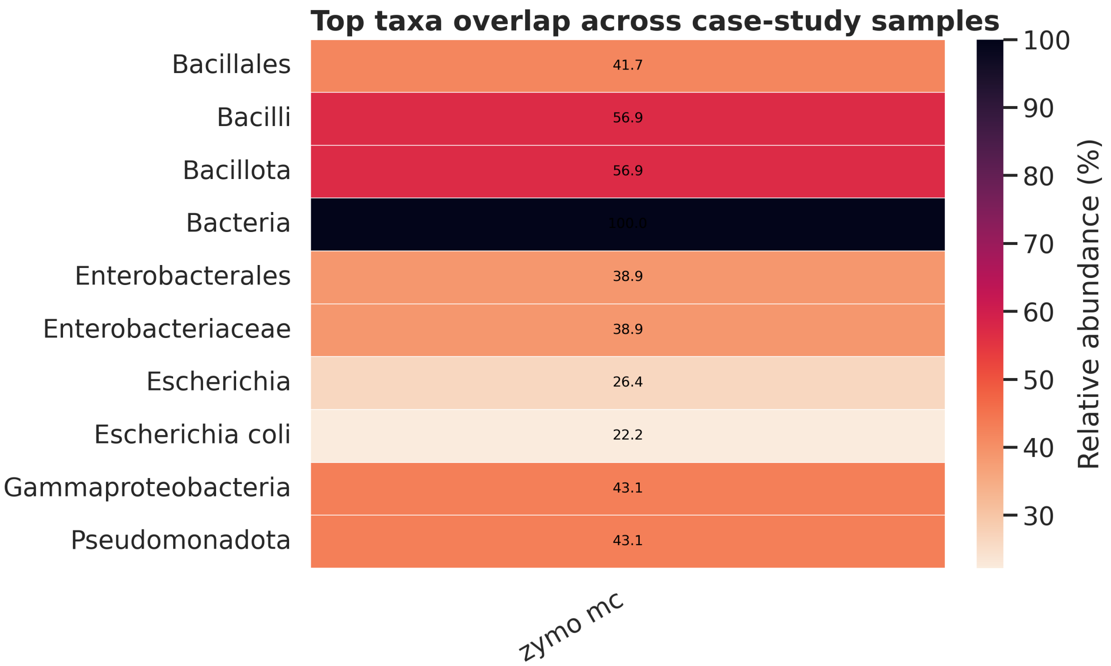

Figure S9: Abundance heatmap for the ZymoBIOMICS mock community case study. Colour intensity encodes relative abundance across taxonomic ranks; darker cells indicate higher contributions.

Genus	Truth (%)	HYMET (%)	Diff (pp)
Escherichia	34.39	32.96	−1.43
Candida	30.84	30.90	+0.06
Saccharomyces	28.97	28.84	−0.13
Clostridioides	1.87	0.00	−1.87
Methanobrevibacter	1.50	1.50	+0.00
Prevotella	0.75	0.56	−0.19
Bifidobacterium	0.37	0.37	+0.00
Akkermansia	0.19	0.19	+0.00
Bacteroides	0.19	0.19	+0.00
Faecalibacterium	0.19	0.19	+0.00
Fusobacterium	0.19	0.37	+0.19
Lactobacillus	0.19	0.00	−0.19
Roseburia	0.19	0.00	−0.19
Veillonella	0.19	0.19	+0.00
Citrobacter	0.00	0.19	+0.19
Enterobacter	0.00	0.75	+0.75
Hallella	0.00	0.19	+0.19
Klebsiella	0.00	0.19	+0.19
Lodderomyces	0.00	0.19	+0.19
Romboutsia	0.00	1.87	+1.87
Shigella	0.00	0.19	+0.19
Wujia	0.00	0.19	+0.19

Table S19: Full genus-level abundance comparison for the ZymoGut D6331 case study. Truth percentages are derived from contig-level mapping to manufacturer reference genomes; HYMET percentages are computed from classified contig counts. Diff is the arithmetic difference (HYMET − Truth) in percentage points. Genera with a truth abundance of 0.00% represent false-positive assignments.

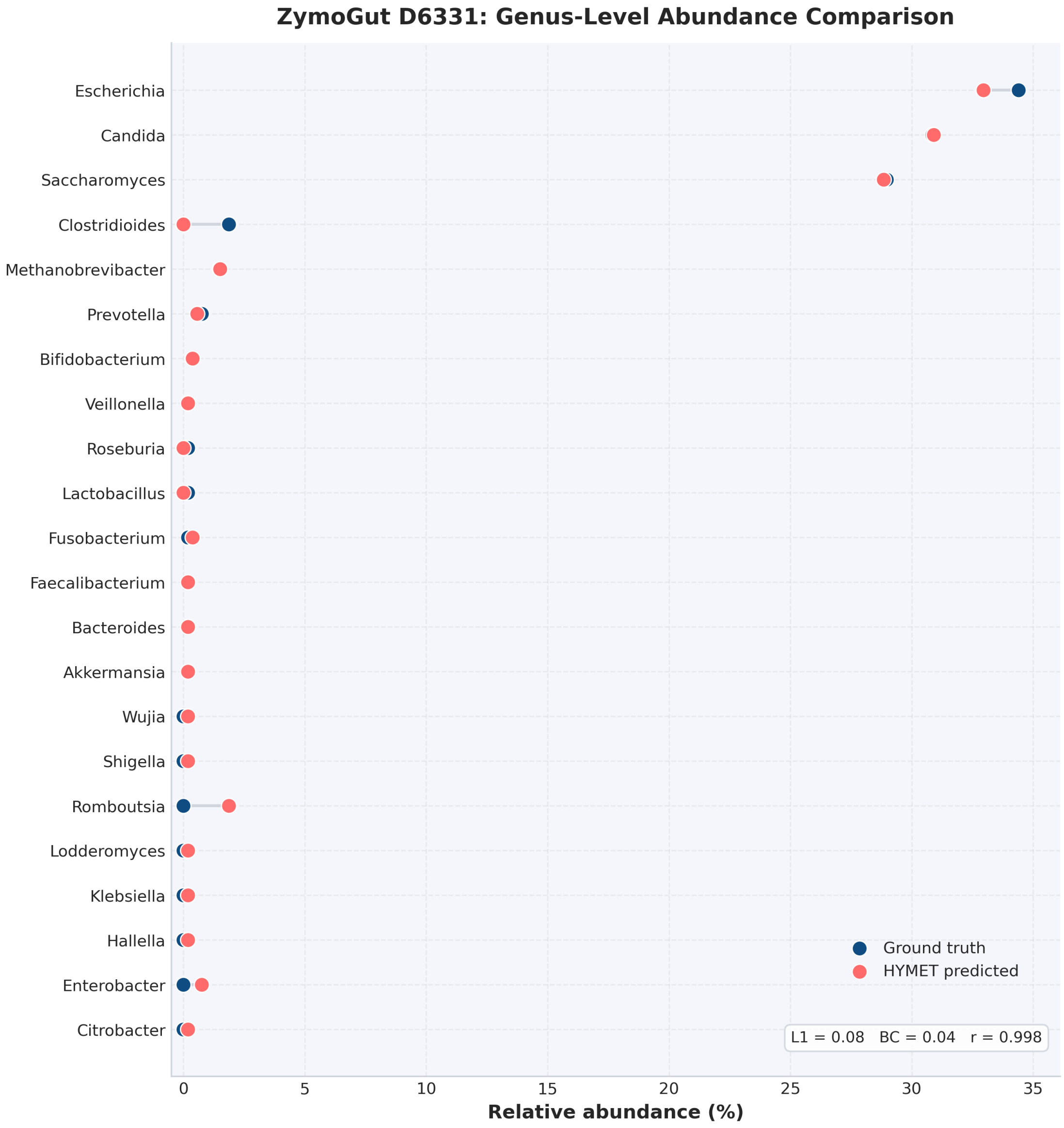

Figure S10: Genus-level abundance comparison for the ZymoGut D6331 case study. Cleveland dot plot showing ground-truth (blue) and HYMET-predicted (orange) relative abundances for all genera present in either the reference or the classification output. Genera are ordered by decreasing truth abundance.
