# Supplementary material for: HYMET: a hybrid metagenomic pipeline for accurate and efficient taxonomic classification
Source: Gigascience. 2026 Mar 2;15:giag024. doi: 10.1093/gigascience/giag024 (PMC13042306; doi:10.1093/gigascience/giag024)

# GigaScience

# HYMET: A Hybrid Metagenomic Pipeline for Accurate and Efficient Taxonomic Classification

--Manuscript Draft--

| | |
|---|---|
| **Manuscript Number:** | GIGA-D-25-00184 |
| **Full Title:** | HYMET: A Hybrid Metagenomic Pipeline for Accurate and Efficient Taxonomic Classification |
| **Article Type:** | Technical Note |
| **Funding Information:** | FCT Fundação para a Ciência e a Tecnologia (00127-IEETA) — Not applicable<br>European Commission (101081813) — Dr. Jorge Miguel Silva<br>FCCN Fundação para a Computação Científica Nacional (2023.14342.CPCA.A1) — Not applicable |
| **Abstract:** | Background: Metagenomics has revolutionized the study of microbial communities. Despite many advances, the field still faces many challenges, such as high computational demands, limited accessibility of tools, and the lack of standardized benchmarking protocols. To address some of these issues, we developed HYMET (Hybrid Metagenomic Tool), a lightweight, mutation-tolerant pipeline designed for accurate and efficient taxonomic classification across all biological domains. HYMET integrates k-mer screening and alignment-based methods to improve computational efficiency and accuracy, particularly in handling genetic mutations.<br>Findings:  HYMET demonstrates superior performance compared to existing tools, achieving high F1 scores across multiple taxonomic groups and taxonomic levels. It maintains robust accuracy even under mutation rates of up to 30\%, with F1 scores above 0.8 for most domains. The pipeline processes most datasets in less than an hour, significantly faster than current state-of-the-art tools, while requiring minimal computational resources (2.82 GB for installation). HYMET’s dynamic database construction and weighted Lowest Common Ancestor algorithm enhance its ability to handle diverse and fragmented metagenomic data, making it highly adaptable to different biological domains.<br>Conclusions: HYMET represents a significant advance in metagenomic analysis, offering a balance of precision, efficiency, and resistance to mutation. HYMET source code is fully available at https://github.com/ieeta-pt/HYMET. |
| **Corresponding Author:** | Inês Martins, M.D.<br>Universidade de Aveiro Departamento de Electrónica Telecomunicações e Informática: Universidade de Aveiro Departamento de Electronica Telecomunicacoes e Informatica<br>Aveiro, PORTUGAL |
| **Corresponding Author Secondary Information:** | |
| **Corresponding Author's Institution:** | Universidade de Aveiro Departamento de Electrónica Telecomunicações e Informática: Universidade de Aveiro Departamento de Electronica Telecomunicacoes e Informatica |
| **Corresponding Author's Secondary Institution:** | |
| **First Author:** | Inês Martins, M.D. |
| **First Author Secondary Information:** | |
| **Order of Authors:** | Inês Martins, M.D.<br>Jorge Miguel Silva<br>João Rafael Almeida |
| **Order of Authors Secondary Information:** | |
| **Additional Information:** | |

*Powered by Editorial Manager® and ProduXion Manager® from Aries Systems Corporation*

| Question | Response |
| --- | --- |
| Are you submitting this manuscript to a special series or article collection? | No |
| **Experimental design and statistics**<br><br>Full details of the experimental design and statistical methods used should be given in the Methods section, as detailed in our Minimum Standards Reporting Checklist. Information essential to interpreting the data presented should be made available in the figure legends.<br><br>Have you included all the information requested in your manuscript? | Yes |
| **Resources**<br><br>A description of all resources used, including antibodies, cell lines, animals and software tools, with enough information to allow them to be uniquely identified, should be included in the Methods section. Authors are strongly encouraged to cite Research Resource Identifiers (RRIDs) for antibodies, model organisms and tools, where possible.<br><br>Have you included the information requested as detailed in our Minimum Standards Reporting Checklist? | Yes |
| **Availability of data and materials**<br><br>All datasets and code on which the conclusions of the paper rely must be either included in your submission or deposited in publicly available repositories (where available and ethically appropriate), referencing such data using a unique identifier in the references and in the "Availability of Data and Materials" section of your manuscript. | Yes |

*Powered by Editorial Manager® and ProduXion Manager® from Aries Systems Corporation*

| | |
|---|---|
| Have you have met the above requirement as detailed in our Minimum Standards Reporting Checklist? | |
| GigaScience has policies and guidelines in place for the use of generative AI-writing tools such as ChatGPT. If you have used such writing tools to assist with writing the manuscript this must be declared and cited in the text. Authors should not list AI-writing tools and other AI-assisted technologies as an author or co-author and should acknowledge that they are fully responsible for text generated or refined by AI-writing tools.<p><br><br>A summary of use (particularly in the introduction or among methods) needs to be included at the end of the paper, and the outputs should also be included as a supplementary file hosted in GigaDB or other open repositories. Please <a href=https://academic.oup.com/gigascience/pages/editorial_policies_and_reporting_standards target="_new" > read our guidelines for more information. </a> <p><br><br>By submitting to GigaScience, you are aware of the journal's AI-writing tools policy, and if you have declared use of such tools below, you have acknowledged this where appropriate in your manuscript and have made a summary of use and outputs available. </b><p><br><b>AI-assisted writing tools have been used in the preparation of this manuscript? | No |

Click here to access/download;LaTeX - Main Document (TeX file);main.tex

*GigaScience*, 2023, 1–10

doi: xx.xxxx/xxxx
**Manuscript in Preparation**
**Paper**

PAPER

# HYMET: A Hybrid Metagenomic Pipeline for Accurate and Efficient Taxonomic Classification

Inês Martins[1,*], Jorge Miguel Silva[1,*] and João Rafael Almeida[1]

[1]IEETA/DETI, LASI, University of Aveiro, Aveiro, Portugal

[*]inesbrancomartins@ua.pt; jorge.miguel.ferreira.silva@ua.pt

## Abstract

**Background:** Metagenomics has revolutionized the study of microbial communities. Despite many advances, the field still faces many challenges, such as high computational demands, limited accessibility of tools, and the lack of standardized benchmarking protocols. To address some of these issues, we developed HYMET (Hybrid Metagenomic Tool), a lightweight, mutation-tolerant pipeline designed for accurate and efficient taxonomic classification across all biological domains. HYMET integrates $k$-mer screening and alignment-based methods to improve computational efficiency and accuracy, particularly in handling genetic mutations.
**Findings:** HYMET demonstrates superior performance compared to existing tools, achieving high F1 scores across multiple taxonomic groups and taxonomic levels. It maintains robust accuracy even under mutation rates of up to 30%, with F1 scores above 0.8 for most domains. The pipeline processes most datasets in less than an hour, significantly faster than current state-of-the-art tools, while requiring minimal computational resources (2.82 GB for installation). HYMET's dynamic database construction and weighted Lowest Common Ancestor algorithm enhance its ability to handle diverse and fragmented metagenomic data, making it highly adaptable to different biological domains.
**Conclusions:** HYMET represents a significant advance in metagenomic analysis, offering a balance of precision, efficiency, and resistance to mutation. HYMET source code is fully available at https://github.com/ieeta-pt/HYMET.

**Key words**: Metagenomics, taxonomic classification, $k$-mer screening, alignment-based methods, computational efficiency, mutation resistance, hybrid pipeline.

## Introduction

Metagenomics is a rapidly evolving field that studies the collective genetic material of microorganisms within specific environments. It has revolutionized our understanding of microbial diversity, enabling discoveries in biotechnology, environmental monitoring, and human health [1, 2]. However, the analysis of metagenomic data is heavily based on metagenomic tools, whose effectiveness varies due to factors such as data quality, computational resources, and algorithmic design. Consequently, understanding the strengths and limitations of these tools is crucial for addressing the challenges of metagenomics and improving the current state-of-the-art [3, 4].

A primary challenge in metagenomics is the development of accurate methods for the taxonomic classification of organisms within a sample [5, 3]. Despite the creation of numerous general-purpose and specialized metagenomic tools, several significant hurdles persist. Computational demands pose a major constraint, as tools often require substantial memory and processing power, leading to impractical execution times for large datasets [6, 7, 5]. The sheer volume of metagenomic datasets demands highly efficient algorithms that can operate within reasonable requirements of compute power, which is particularly problematic when dealing with millions of sequencing reads [1, 8, 9, 10]. Furthermore, taxonomic assignment remains a critical challenge in metagenomic analysis, especially at lower taxonomic levels [10]. This issue is exacerbated by the limitations of reference databases, which often exhibit significant sampling bias towards well-studied organisms, while underrepresenting species that are difficult to culture in laboratory settings [6, 5]. This discrepancy results in high rates of

**Compiled on:** May 9, 2025.
Draft manuscript prepared by the author.

**Key Points**

- This study presents HYMET, a novel pipeline combining k-mer screening (Mash) and alignment-based methods (Minimap2) for comprehensive taxonomic classification across all biological domains, addressing critical limitations in current metagenomic analysis tools.
- HYMET was evaluated against nine state-of-the-art tools including comprehensive pipelines (SnakeMAGs, SqueezeMeta), general classifiers (BASTA, CAMITAX, TAMA), domain-specific tools (PhaBOX/ViWrap for viruses, PhyloFlash for rRNA), and antimicrobial resistance classifier MegaPath-Nano.
- The study employed a curated dataset of 26 203 genomes (14.76 GB) from NCBI RefSeq and GTDB, spanning all biological domains: Bacteria, Archaea, Eukarya (fungi, plants, vertebrates) and viruses, featuring simulated mutations (0-30%) for robustness testing.
- HYMET achieved superior accuracy (F1 > 0.9 for every domain vs < 0.5 in state-of-the-art tools), faster processing (2h vs BASTA/SqueezeMeta's 20h), and maintained robust performance (F1 > 0.8 at 30% mutations, viruses:  0.5) while requiring minimal resources (2.82 GB installation).
- HYMET overcomes taxonomic biases through dynamic database construction and a hybrid k-mer/alignment approach, outperforming specialized tools while maintaining universal applicability across all biological domains.

unclassified or misclassified reads, especially in complex environmental samples [4, 3, 2]. Compounding these challenges is the lack of standardized benchmarking protocols and datasets, which hinders objective comparisons of tool performance, as researchers frequently test tools on non-uniform datasets with inconsistent evaluation metrics [2, 11, 12]. Addressing these issues is crucial for advancing our understanding of complex microbial communities and developing efficient, user-friendly software solutions to analyze the enormous amounts of data generated by metagenomic research [6, 5]. These collective challenges directly motivate our core research question:

> *How can a next-generation metagenomic classification tool be designed and implemented to accurately identify taxa across all domains while maintaining high performance and efficiency?*

To address this question, in this paper, we present HYMET (Hybrid Metagenomic Tool), a lightweight, mutation-tolerant solution capable of accurate cross-domain classification. Our contribution extends beyond the pipeline itself to include a carefully curated standardized dataset representing all biological domains, specifically designed to enable rigorous benchmarking of metagenomic tools. HYMET combines several innovative features, including dynamic database construction, precise alignment algorithms, and user-friendly outputs, to deliver superior accuracy, speed, and usability compared to existing solutions. This work provides researchers with a powerful platform for comprehensive analysis of the microbial community and advances the field toward more standardized, reproducible metagenomic research by overcoming the current limitations of state-of-the-art tools.

## Background

In recent years, we have witnessed remarkable progress in metagenomics, particularly in the development of computational tools for taxonomic classification and functional analysis [3, 5, 6]. A dominant trend in current methodologies is the integration of established classification techniques into end-to-end pipelines, which streamline the entire analytical workflow, from raw sequencing data to biologically interpretable results [13]. Currently, the state-of-the-art landscape is populated by a rich ecosystem of interconnected tools, each offering unique capabilities and complementary approaches that collectively advance the field's analytical power. Among these, SnakeMAGs [14] stands out for its specialized focus on reconstructing prokaryotic genomes from Illumina sequencing reads, while SqueezeMeta [15] offers a fully automated and comprehensive solution for metagenomic data analysis [16, 13]. The first tool uses the Genome Taxonomy Database (GTDB) toolkit [17] for taxonomic assignment, leveraging conserved marker genes for analysis. On the other hand, SqueezeMeta uses DIAMOND [18] for alignment and the Lowest Common Ancestor (LCA) algorithm for taxonomic assignment [15].

Complementing these general-purpose pipelines, several lightweight tools have emerged to address specific needs in taxonomic assignment. The Basic Sequence Taxonomy Annotator (BASTA) [19] also employs the LCA algorithm for efficient sequence classification, while the Critical Assessment of Metagenome Interpretation Taxonomy (CAMITAX) [7] improves accuracy through the integration of multiple classification strategies for microbial genome assignment, including genome distance-based classification using Mash [20], Centrifuge [5] and Kaiju [21], that determines the interval-union LCA of gene-level assignments and 16S rRNA gene-based classification employing a naive Bayesian classifier method using Dada2 [22]. For more robust taxonomic profiling, the Taxonomy Analysis by Multiple Assignment (TAMA) tool [23] combines consensus classifications from established classifiers, including Kraken [10], CLARK [24], and Centrifuge, leveraging their complementary strengths.

The field has also seen the development of specialized tools that target specific metagenomic applications. Viral genomics is particularly well served by PhaBOX [25, 26] for viral contig characterization and ViWrap [27] for prediction of viral-host relationship, both providing valuable information on viral diversity and ecological interactions [28, 29, 30]. The first tool, PhaBOX, developed by Shang et al., combines gene prediction and alignment (DIAMOND) with taxonomic classification by semi-supervised learning method (PhaGCN [26]), based on sequence similarities and cluster sharing networks, and final assignments using the LCA. ViWrap, on the other hand, uses machine learning and sequence similarity searches to identify viral sequences and BLAST [31] to identify best hits against databases for taxonomic annotation and host prediction. For microbial community analysis, PhyloFlash [32], developed by Gruber-Vodicka et al., offers unique capabilities through its small subunit ribosomal RNA (SSU rRNA)-based approach, enabling both metagenomic profiling and high-resolution phylogenetic studies [33]. In the critical area of antimicrobial resistance surveillance, MegaPath-Nano [34] has emerged as an important tool for the comprehensive detection of resistance genes, which directly supports public health monitoring efforts, which uses Minimap2 [35] for global alignment and hash-based k-mer mapping, prioritizing global matching of large portions of sequences [36, 37].

Despite this technological progress, significant challenges impede the broader implementation of metagenomic tools in clinical and research settings. Implementation barriers represent a primary obstacle, with inadequate documentation and complex installation procedures frequently compromising tool accessibility and user

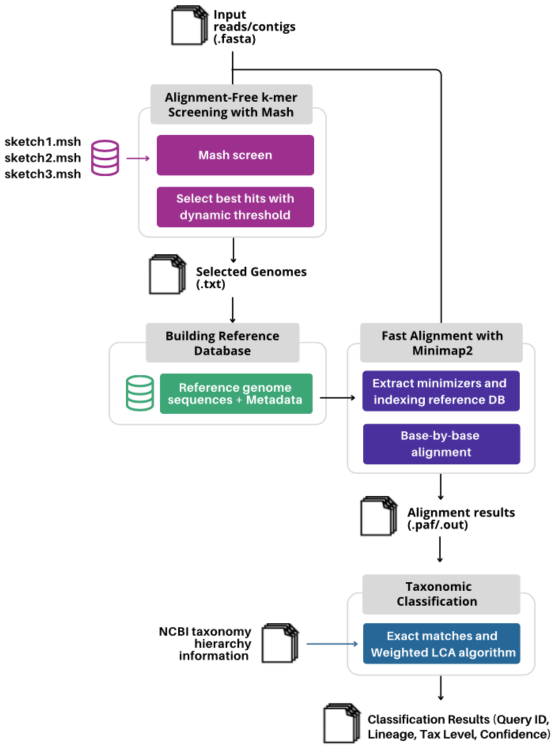


**Figure 1.** Overview of HYMET architecture.

adoption [38]. Computational constraints further limit practical application, as excessive memory and storage requirements hinder scalability. This is exemplified by SqueezeMeta, which demands over 500 GB of storage capacity and exhibits prohibitively long processing times [15], and BASTA, whose dependence on BLAST-based alignments creates computational bottlenecks that render it inefficient for time-sensitive analyses [19, 31]. A fundamental limitation stems from reference database dependencies rather than inherent tool restrictions. For instance, TAMA demonstrates robust classification capabilities in principle, but its default bacterial reference database necessarily limits its taxonomic scope to bacterial identification [23]. Similarly, independent evaluations of MegaPath-Nano confirm its strong performance in the detection of prokaryotic antimicrobial resistance, but note a reduced sensitivity when analyzing higher eukaryotes [34, 36]. This pattern of taxonomic bias is further evidenced in specialized tools such as PhaBOX and ViWrap, which, while excelling in virome analysis, lack versatility for broader metagenomic applications [27, 25]. PhyloFlash's reliance on small subunit rRNA analysis makes it fundamentally unsuitable for viral identification, as viruses lack ribosomal RNA genes [32].

## HYMET´s Workflow

HYMET, illustrated in Figure 1, is implemented using a combination of Perl for workflow orchestration and Python for module execution, with containerization facilitated by Docker to ensure reproducibility and portability. HYMET is also available as a Bioconda package under the name hymet, enabling easy installation and dependency management via the Conda ecosystem. The pipeline integrates two open-source tools: Mash [20] for rapid $k$-mer screening and Minimap2 [35] for precise sequence alignment. The installation and configuration details for HYMET are provided in Supplementary Sections 1 through 4.

## Alignment-Free $k$-mer Screening

The initial phase of HYMET utilizes Mash Screen [20] for rapid $k$-mer-based screening against pre-computed MinHash reference sketch databases [39, 40, 41, 42, 43]. Mash employs containment scores, as defined in Equation 1, to assess the proportion of a reference genome present in a query sequence [44, 45, 46]. The containment index $c_k(a, b)$ is estimated as:

$$c_k(a, b) \approx \frac{|S(A) \cap \pi(B)|}{|S(A)|}, \tag{1}$$

where $S(A)$ is the sketch of the reference genome $A$, and $\pi(B)$ represents the $k$-mers of the query sequence $B$. The containment index ranges from 0.0 to 1.0, with values closer to 1.0 indicating a higher proportion of $k$-mers from the reference genome present in the query. This metric is crucial for tasks such as contamination screening, reference genome selection, and the discovery of novel genomes, as it provides a rapid and unbiased estimate of sequence representation [39, 40, 44, 42, 47]. In this work, the screening process was optimized for computational efficiency by enabling parallel processing and applying a stringent 90% similarity threshold to retain only high-confidence matches, filtering out low-quality alignments. This containment-based approach prioritizes likely taxonomic candidates, reducing the search space and computational load for subsequent alignment-based stages [20, 39, 45]. As previously mentioned, to enable this screening, Mash relies on sketched databases, which are compact representations of genomic sequences. These databases are built using consistent $k$-mer hashing with MurmurHash3 [20, 48, 40, 49], which allows efficient comparison of query sequences against large collections of reference genomes.

### *Sketch and $k$-mer Size*

The construction of these sketch databases involves two key parameters: the $k$-mer size ($k$) and the sketch size ($s$). The choice of $k$-mer size is essential as it balances sensitivity and specificity. Smaller $k$-mers increase sensitivity for divergent genomes but may lead to random collisions, while larger $k$-mers reduce collisions but may miss subtle variations [50, 20, 40, 44, 43]. The optimal $k$-mer size is calculated as:

$$k = \log_{|\Sigma|}\left(\frac{n(1-q)}{q}\right), \tag{2}$$

where $|\Sigma|$ is the alphabet size (4 for nucleotides), $n$ is the genome size, and $q$ is the desired probability of observing a random $k$-mer. For example, smaller genomes (e.g. viruses) and highly variable taxa require smaller $k$-mer sizes (e.g. $k$-mer=15) to ensure specificity, while larger genomes (e.g. vertebrates) benefit from moderate $k$-mer sizes (e.g. $k$-mer=21) to balance sensitivity and computational efficiency [46, 50].The sketch size, which refers to the number of unique min-hashes retained for genomic sequence representation, also plays a critical role in determining the accuracy of distance and containment estimates [50, 20, 43, 45]. The error associated with containment estimation for a given sketch size, $s$, is proportional to:

$$\text{Error} \approx \sqrt{\frac{1}{s}}, \tag{3}$$

indicating that larger sketch sizes improve precision, but at the expense of greater computational resources [50]. For instance, smaller or highly fragmented genomes typically require larger sketch sizes to ensure sufficient genomic information is captured, while larger or less fragmented genomes can achieve accurate con-

**Table 1.** Reference sketched databases.

| Sketch | Content | Sketch Parameters | Sketch Size | Seed Value |
|---|---|---|---|---|
| sketch1.msh | RefSeq nucleotide release 88 | k=21, s=1000 | 1.2 GB | 0 |
| sketch2.msh | GTDB r202 Assembly Set, NCBI Complete Genomes Database + Custom databases (vertebrates, plants, invertebrates) | k=21, s=1000 | 883.25 MB | 42 |
| sketch3.msh | Custom databases (fungi, protozoa, archaea, virus) | k=15, s=5000 | 327.93 MB | 42 |

tainment estimates with smaller sketch sizes. This adaptive approach is supported by empirical evidence, with studies demonstrating that a sketch size of $s = 1000$ is generally adequate for obtaining precise similarity estimates in well-assembled genomes [20]. In fact, B. D. Ondov *et al.* established $s = 1000$ and $k$=21 as the default parameters in Mash, as they provide precise similarity estimates for well-assembled genomes [20]. However, for more divergent genomes, increasing the sketch size (e.g. $s = 5000$) can improve accuracy by capturing a more representative subset of genomic content [50, 43**?** , 46].

### *Reference Sketched Databases*

Following these design principles, we implemented a comprehensive database strategy combining both established public resources and a custom-built collection:

- **RefSeq Nucleotide Release 228:** Contains sketches of 162 138 organisms from RefSeq release 228, compressed using $k = 21$ and $s = 1000$, reducing the original 1.2 TB dataset to a compact representation [50].
- **GTDB r202 Assembly Set and NCBI Complete Genomes Database:** Combines 89 675 genomes from GTDB r202 and NCBI RefSeq (viruses, fungi, and bacteria/archaea), compressed with $k = 21$ and $s = 1000$ [49].
- **Custom Reference Database:** Enhances representation of underrepresented taxa by including 19 505 up-to-date genomes from NCBI RefSeq. For smaller genomes (e.g., archaea, fungi, protozoa, viruses), sketches were generated with $k = 15$ and $s = 5000$, while larger genomes (e.g., vertebrates, plants, invertebrates) used the default parameters ($k = 21$, $s = 1000$) [43].

These databases were grouped on the basis of shared seed values and parameters to optimize the screening efficiency. Table 1 summarizes their characteristics. All databases are publicly available through our project repository. For reproduction, detailed instructions are provided in Supplementary Material Section 2, Subsection "Reproducing Sketched Databases".

## Modular Reference Database Download

### *Candidate selection with dynamic threshold*

After running Mash Screen, the output can be extensive, potentially including a large number of candidate genomes with varying degrees of similarity to the query sequences. Downloading and analyzing this entire list would be computationally inefficient and could introduce noise into subsequent analyses. On the other hand, setting an arbitrarily high static threshold might exclude important reference genomes, leading to incomplete coverage of the query sequences. To address these challenges, HYMET introduces a dynamic thresholding mechanism to identify the most relevant candidate genomes and to create a targeted, input-specific database. A dynamic thresholding algorithm identifies relevant candidate genomes by iteratively adjusting a containment score threshold, starting from a preset value and reducing it by 0.02 until a minimum candidate count (3.25 times the number of input queries) is met or a lower limit of 0.71 is reached.

### *Genome Retrieval*

Following the selection of candidate genomes, their format was analyzed to enable efficient mapping and retrieval. These genomes were identified using RefSeq (GCF) and Genbank Assembly Genomes (GCA) accession numbers, unique identifiers assigned by NCBI. Thus, the NCBI Assembly database [51] was selected as the primary resource for constructing the reference database [2, 52]. To optimize the process, summary files from the NCBI Assembly database were downloaded, providing efficient access to metadata. A custom script was developed to map candidate genomes to these files using accession numbers. This script extracted the base accession number (e.g. "000169215") to ensure compatibility between different assembly versions (e.g.: GCF_000169215.1, GCF_000169215.2), preventing retrieval failures due to version updates [51]. Genomes were downloaded and decompressed from NCBI FTP server and saved locally in FASTA format. To enhance efficiency, the script employed ThreadPoolExecutor for parallel downloads, allowing up to 64 concurrent threads, and incorporated retry logic with exponential backoff to handle transient network errors. The taxonomy IDs (TaxID) of the assembly files were stored alongside the accession numbers and sequence identifiers, creating a comprehensive reference linking each genome to its taxonomic and sequence-level information [51, 52].

## Fast Alignment

In the second processing stage, HYMET employs Minimap2 for efficient and precise sequence alignment. This choice was motivated by Minimap2's adaptive scoring system, and global alignment capabilities enable accurate mapping even with highly divergent sequences or incomplete reads, making the pipeline particularly resilient to common metagenomic challenges such as mutation-rich or fragmented samples [34, 35]. The pipeline uses minimizers to index reference sequences, enabling the rapid identification of alignment regions [53]. This was executed with the `-x asm10` parameter, which is optimized for genome-to-genome alignment and allows up to 10% sequence divergence, which is equivalent to a 90% identity threshold, ensuring robust alignment even in the presence of significant genetic differences [54, 35]. The results are saved in a PAF file, providing essential alignment details such as sequence IDs, lengths, positions, and mapping quality [35, 55].

## Taxonomic Assignment

HYMET uses a hybrid taxonomic assignment strategy, combining the LCA algorithm with a weighted approach based on alignment coverage [23, 10, 24]. For exact matches, the reference's taxonomic lineage is directly assigned with a confidence score of 1.0. On the other hand, for non-exact matches, the weights for each TaxID are calculated according to:

$$\text{Weight} = \text{Coverage} \times \text{Abundance}, \tag{4}$$

where *Coverage* is the proportion of the query aligned with the reference and *Abundance* is the reference's frequency in the dataset. The most weighted TaxID at each taxonomic level is selected, and the confidence score is derived as the product of confidence values

**Table 2.** Composition of the test and validation dataset

| Domain/Group | Number of GCFs | Size (GB) |
|---|---|---|
| Viruses | 1 498 | 0.05 |
| Other Vertebrates | 43 | 2.83 |
| Vertebrate Mammals | 23 | 2.29 |
| Protozoa | 12 | 0.03 |
| Plants | 19 | 1.02 |
| Invertebrates | 43 | 1.14 |
| Fungi | 63 | 0.15 |
| Bacteria | 24 271 | 7.23 |
| Archaea | 231 | 0.05 |
| **Total** | **26 203** | **14.76** |

across ranks:

$$\text{Confidence Score} = \prod_{i=1}^{n} \text{Confidence at Rank}_i, \qquad (5)$$

where $n$ is the number of ranks. This ensures higher consistency across ranks results in higher confidence scores. The final output includes the query identifier, taxonomic lineage (kingdom to strain), most specific rank, and a confidence score (0.0 to 1.0), reflecting classification reliability [24, 56, 57].

## Materials and Methods

All analyses, including tool evaluation, development, and validation, were conducted on a high-performance Linux-based virtual machine with 2 TB storage and 250 GB RAM. HYMET's performance was assessed using precision and F1 score metrics for classifying organisms across the three domains of life, considering taxonomic levels (kingdom to species) and mutation rates (0% to 30%). The analysis also examined the relationship between F1 scores, execution time, and resource usage (CPU and memory), ensuring a comprehensive evaluation of accuracy and efficiency. The same methodology was applied to the other current state-of-the-art tools described in Section to ensure a consistent comparison. Detailed instructions for reproducing the benchmarking of these tools are provided in Supplementary Section 5.

### *Test and Validation Dataset*

The test dataset was derived from the NCBI RefSeq Assembly database (last modified: 13 October 2024), chosen for its curated and validated sequences [58, 2, 59]. Assembly summary files for all biological domains and viruses were downloaded and 10% of the entries were randomly selected based on GCF accession numbers. For each GCF, 10% of its genome sequences were further sampled to ensure proportional representation and mimic the fragmentation of metagenomic data [60]. This approach resulted in a diverse and representative dataset, as detailed in Table 2. For dataset replication, complete instructions and scripts are provided in the Supplementary Material Section 3, Subsection"Replicating the Benchmark Dataset".

## Results

### *Performance Evaluation*

HYMET demonstrates superior classification performance compared to other state-of-the-art tools, as evidenced by consistently high F1 scores across diverse taxonomic groups (Figure 2; Supplementary Tables 1–8). While most state-of-the-art tools struggle with F1 scores below 0.5, HYMET stands out, particularly at higher taxonomic levels (kingdom to class), where it achieves perfect F1 scores (1.0) for vertebrate mammals, plants, and bacteria. Performance decreases slightly to 0.8–0.9 at the species level, but remains robust for archaea and microbial eukaryotes (fungi, protozoa), with F1 scores exceeding 0.95 throughout all levels. For viruses, accuracy remains high at the species and kingdom levels (above 0.9), with a minor decline at intermediate ranks.

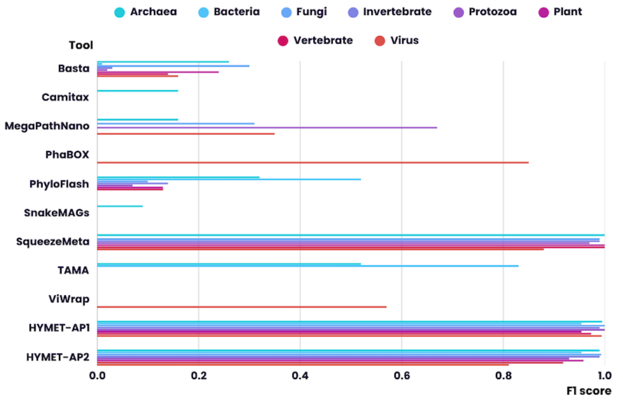


**Figure 2.** F1 scores achieved by various taxonomic classification tools, including both state-of-the-art tools and HYMET, across different taxonomic groups. The x-axis represents the F1 scores, ranging from 0 to 1, while the y-axis lists the evaluated tools. Each bar is color-coded to indicate the corresponding taxonomic group. This data is based on analysis with 0% mutation rate.

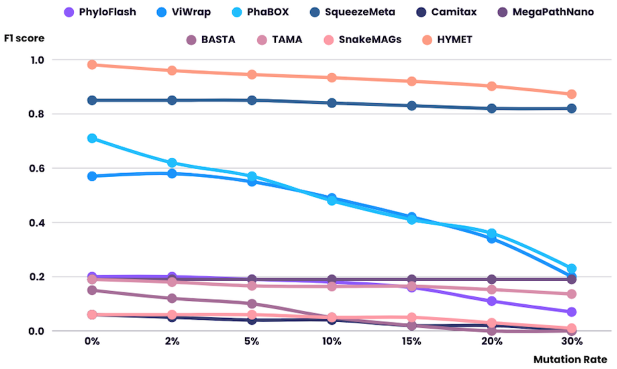


**Figure 3.** Performance of the state-of-the-art tools and HYMET as the mutation rate increases. The x-axis represents the mutation rate (ranging from 0% to 30%), while the y-axis shows the F1 score. Each curve on the graph corresponds to a different tool.

### *Mutation Resilience*

HYMET's performance proves exceptionally stable under varying mutation rates (0–30%), outperforming all benchmarked tools in both accuracy and consistency (Figure 3). Viral classification shows a progressive decline at extreme mutations (F1 scores approximately 0.5 at thirty percent), while archaea, invertebrates and fungi maintain F1 scores over 0.9. Other groups show only minor, non-significant reductions, staying above 0.8 (Figure 4). This contrasts with competing tools, where their scores decline as the mutation rate increases (Supplementary Figures 1-2).

### *Computational Efficiency*

The analysis of Figure 5 and Supplementary Figure 3 reveals significant variations in execution time. Tools like PhyloFlash, PhaBOX, TAMA and ViWrap demonstrate rapid processing with execution times under one hour, while SqueezeMeta offers good performance at the cost of longer computational times. In contrast, BASTA consistently shows the longest execution times, approaching more than 20 hours for every domain, with correspondingly low performance across different taxonomic domains. On the other hand,

**Table 3.** Computational Resources required by each tool.

| Tool | Resources (GB) | CPU Usage (%) | Memory Usage (%) |
|---|---|---|---|
| **HYMET** | **2.82 (+10-50)** | **13.04** | **13.65** |
| BASTA | 40 | 12.72 | 3.05 |
| CAMITAX | 33 | 22.37 | 21.08 |
| MegaPath-Nano | 71 | 28.86 | 15.71 |
| PhaBOX | 2.8 | 69.16 | 3.67 |
| PhyloFlash | 16 | 34.21 | 3.86 |
| SnakeMAGs | 67 | 20.17 | 3.57 |
| Squeezemeta | 403 | 54.92 | 17.03 |
| ViWrap | 2 | 40.65 | 8.76 |
| TAMA | 285 | 51.77 | 32.47 |

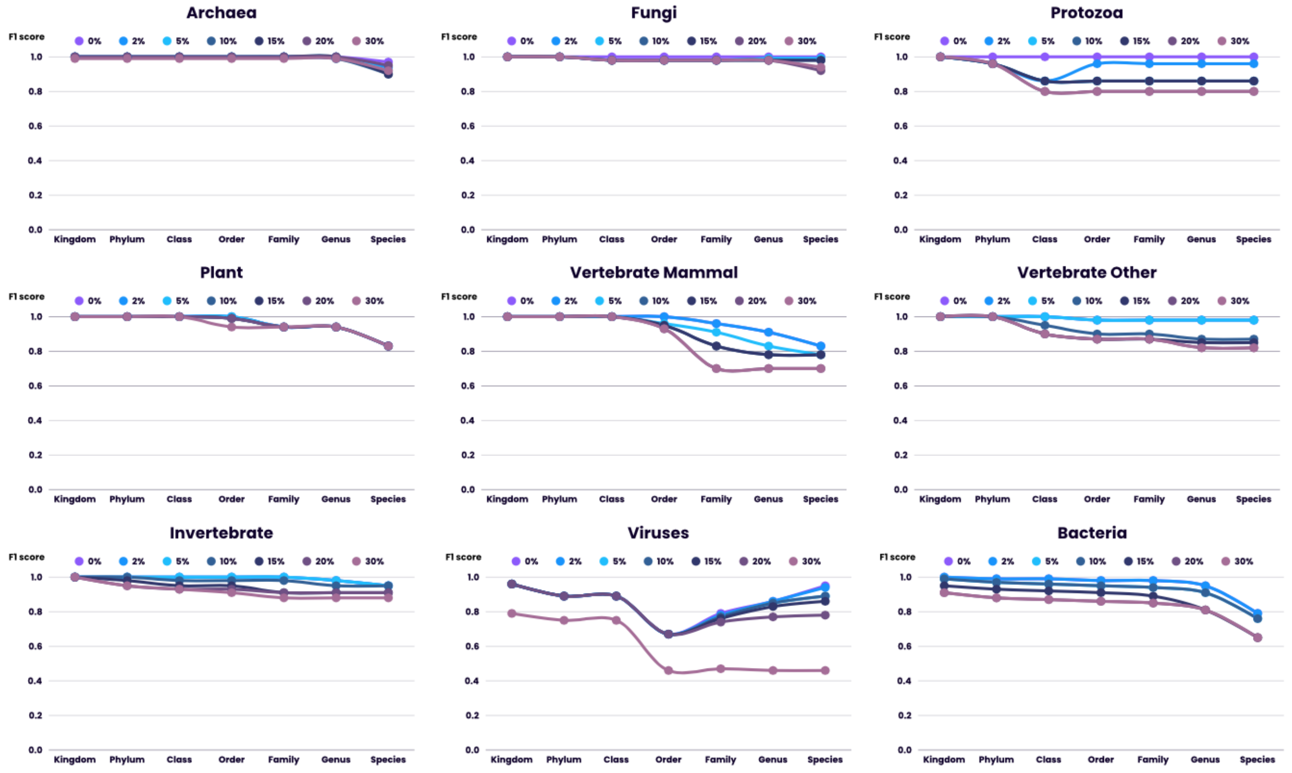


**Figure 4.** Performance of HYMET across taxonomic levels for different taxonomic groups as mutation rates increase (0% to 30%). The x-axis shows taxonomic levels (kingdom to species), and the y-axis represents the F1 score. Each curve corresponds to a specific mutation rate, and each graph focuses on a particular taxonomic group.

HYMET processes datasets in approximately 2 hours, significantly faster than existing tools, while maintaining an overall F1 score of approximately 0.95. Execution times vary by taxonomic group, with larger eukaryotes and bacteria taking more than 150 minutes, while microbial eukaryotes, archaea, and viruses are processed in approximately 10 minutes. In terms of computacional resource requirements, represented in Table 3, HYMET requires only 2.82 GB for installation and configuration, with dynamically downloaded reference databases typically ranging from 10 to 50 GB, depending on metagenomic composition. This balance of speed, accuracy, and resource efficiency positions HYMET as a leading tool for metagenomic analysis.

## Discussion

HYMET's success stems from its innovative design and methodological advances, setting it apart from existing tools and overcoming current limitations. A key innovation is the use of Mash Screen for sequence pre-filtering, which represents a novel contribution to the field, as no existing work has utilized Mash for screening purposes in this context [7, 49, 61], enhancing efficiency and accuracy in candidate selection. Moreover, HYMET employs a dynamic threshold selection methodology that adapts to the specific characteristics of each sample, ensuring optimized performance across diverse datasets. This is a significant improvement over fixed-threshold approaches, such as those used in CAMITAX, which rely on static thresholds for candidate selection, leading to the exclusion of potentially relevant candidate genomes [7, 61]. For taxonomic classification, HYMET implements a weighted LCA algorithm, which improves the widely used LCA method among the state-of-the-art tools [15, 25, 10], with the incorporation of an adaptive scoring methodology. This refinement reduces penalties for single-nucleotide mismatches, which are common in highly mutated sequences, and excludes low-confidence reads prior to taxonomic assignment. This strategy, similar to that used in TAMA, significantly improves classification precision, particularly at lower taxonomic levels, where closely related species can be difficult to distinguish [23, 24, 10].

Another important feature of HYMET is its modular database system, which provides flexibility and adaptability to different biological domains, demonstrating remarkable efficiency in resource utilization. This allows operation on standard computing infras-

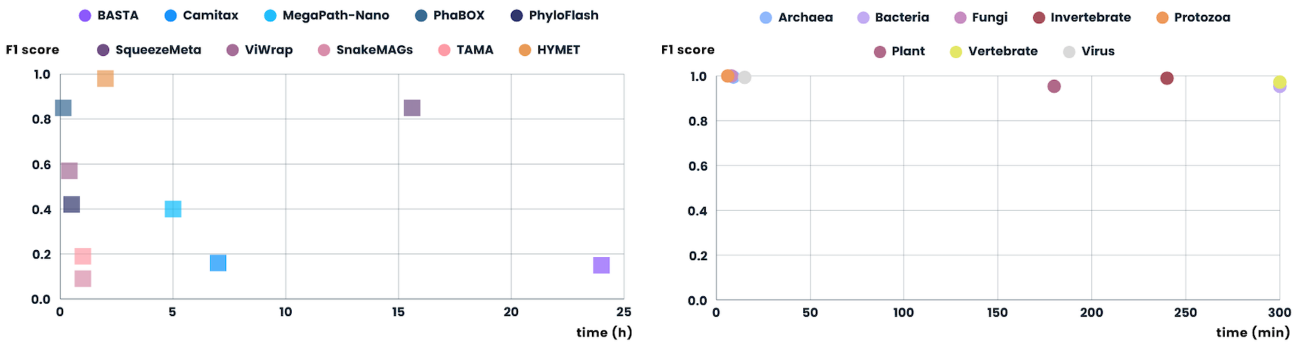


**Figure 5.** Relationship between execution time (in hours) and F1 score (ranging from 0.0 to 1.0) across various domains for HYMET and state-of-the-art tools, as well as HYMET's relationship between execution time (in minutes) and F1 score across biological domains.

tructure, reducing the dependency on high-performance computing resources, distinguishing it from state-of-the-art tools that depend on fixed and extensive reference databases [23, 15, 14]. For instance, Squeezemeta (403 GB) and TAMA (285 GB) require significantly higher memory. This advantage is directly related to the pipeline's scalability, which adapts to the complexity and size of the query sample. The reference database is dynamically generated based on the sample's content, resulting in execution times and resource requirements that scale linearly with genome complexity. For example, processing viral or small genomes is significantly faster due to the reduced size of candidate genomes to be downloaded and aligned. In contrast, while processing complex eukaryotic genomes increases computation time, HYMET remains faster than state-of-the-art tools like BASTA, Squeezemeta, and MegaPath-Nano. Unlike these tools, which rely on predefined reference databases, HYMET's dynamic approach allows for more efficient adaptation to the sample's content, minimizing resource waste, and improving overall performance.

A key strength of HYMET is its two-stage mutation-handling framework. During screening, the custom sketched database, built using optimized parameters ($k$ = 15, $s$ = 5000) improves genomic diversity representation and sensitivity to mutations. These settings increased candidate matches by approximately 30% for sequences with 30% mutations and reduced false negatives by 25% in viral sequences during the screening stage. In the alignment stage, Minimap2's seed-chain-align approach identifies exact matches and extends them, tolerating gaps and mismatches. This ensures robust performance, with F1 scores greater than 0.5 for viral sequences with mutation rates of 20–30% and greater than 0.8 for other domains with high mutation rates [20, 35]. This combination of an optimized database and advanced alignment makes HYMET highly effective in handling mutations.

Overall, HYMET demonstrates robust performance across diverse biological domains, although its accuracy and efficiency vary depending on the complexity of the organisms being analyzed. For bacteria, despite the challenges posed by a large test dataset of ~20 000 genomes, HYMET achieves high accuracy at higher taxonomic levels, though species-level classification is more challenging due to genetic similarity among closely related species. The sheer volume of sequences also increases computational demands, suggesting that a more balanced dataset with fewer representative genomes could improve consistency and reduce execution time. Within Eukarya, microbial eukaryotes such as fungi and protozoa achieve high accuracy across all taxonomic levels, benefiting from distinct genetic signatures and well-documented reference genomes, just like archaeal organisms. In contrast, larger eukaryotic organisms, such as vertebrates and plants, show reduced accuracy at the species level due to their complex genomes, higher intraspecies diversity, and closer evolutionary relationships [62]. Uneven representation in reference databases further complicates classification, as bias towards well-studied species limits the diversity of reference sequences [58, 17]. For viruses, HYMET excels at species-level classification due to the distinct genetic signatures of viral strains, but accuracy decreases at higher taxonomic levels, reflecting the polyphyletic nature of viral evolution and the lack of a unified taxonomic framework [63, 64].

Despite the pipeline's robust performance in metagenomic classification, particularly at higher taxonomic levels, we observed a decrease in accuracy at the genus and species levels, specially as mutation increases. This reduction in precision can be attributed to several factors. Firstly, bias in reference databases is a significant consideration. The uneven representation of species and genera in databases can result in skewed classifications, favoring more extensively documented organisms. Additionally, a significant drawback encountered during testing was the challenge of genome downloads, particularly with the RefSeq sketched database [58]. As an older version, some genomes were no longer available in the assembly files, while others had non-functional links or had been suppressed. Quantitative analysis revealed an overall 6.04% failure rate in genome retrieval across all datasets, which significantly impacted the pipeline's performance. This issue likely contributed to the reduced F1 scores observed at the genus and species levels. This issue leads to the non-representation of certain organisms in the reference database, even when detected as candidates by Mash Screen. Consequently, this may explain the reduced F1 scores observed at the genus and species levels. These limitations underscore the importance of maintaining up-to-date and comprehensive databases, as well as the need to develop strategies for handling missing or inaccessible genomes to improve classification accuracy across all taxonomic levels.

## Conclusion

HYMET represents a significant methodological advancement in metagenomic analysis through its innovative integration of $k$-mer screening with precise alignment-based classification. The pipeline's core strength lies in its modular architecture, which combines dynamic Mash-based filtering, weighted LCA algorithms, and an optimized reference database system to achieve robust taxonomic assignment across diverse biological domains. This integrated approach addresses critical limitations in current tools, particularly in handling highly divergent sequences and uneven reference databases, while maintaining computational efficiency on standard infrastructure. Future development of HYMET should prioritize the implementation of a dynamically updated reference database system incorporating multiple genomic repositories to address current limitations in genome retrieval and taxonomic coverage, and integration of machine learning classifiers, particularly deep neural networks, to improve discrimination at lower taxonomic ranks where sequence similarity challenges conventional methods [29, 30]. In the long term, transforming HYMET into

a comprehensive metagenomic analysis platform, encompassing quality control, assembly, annotation, and interactive visualization, would address critical gaps in current end-to-end solutions [16, 13]. These strategic improvements would position HYMET as both a robust taxonomic classifier and a versatile framework for reproducible large-scale metagenomic studies across basic and translational research applications.

## Availability of source code and requirements

- Project name: HYMET (Hybrid Metagenomic Tool)
- Project home page: https://github.com/ieeta-pt/HYMET
- Operating system(s): Linux
- Programming language: Perl, Python, Bash
- Other requirements: Docker, Conda
- License: No restrictions.

## Data Availability

The Supplementary Material accompanying this article includes comprehensive instructions for reproducibility, encompassing all aspects of data analysis, tool utilization, environment configuration, script execution, and supplementary results.

## Additional Files

***Supplementary Tab. S1 to S8.*** These tables presents the performance results of HYMET and each state-of-the-art tool, including Precision, Recall, and F1 score, for different taxonomic groups categorized by taxonomic levels (Kingdom, Phylum, Class, Order, Family, Genus, Species) with mutation rates of 0%. Each table represents a specific taxonomic group, and each row within the table corresponds to a different tool. The columns of each table are organized by taxonomic level, with the corresponding performance metrics listed for each level.

***Supplementary Tab. S9 to S15.*** These tables presents the performance results of HYMET, including Precision, Recall, and F1 score, across all taxonomic groups for mutation rates ranging from 0% to 30%. Each row corresponds to a specific taxonomic group, while the columns are organized by taxonomic levels (Kingdom, Phylum, Class, Order, Family, Genus, Species). The performance metrics (F1 score, Precision, and Recall) are listed for each taxonomic level, with each table representing a distinct mutation rate.

***Supplementary Fig. S1 and S2.*** This figure illustrates the performance of the state-of-the-art tools as the mutation rate increases, across different taxonomic levels for various taxonomic groups. The x-axis represents the taxonomic levels (Kingdom, Phylum, Class, Order, Family, Genus, Species), while the y-axis shows the F1 score. Each curve on the graph corresponds to a different mutation rate, ranging from 0% to 30%, and each graph represents the performance of one tool within a specific taxonomic group.

***Supplementary Fig. S3.*** This figure illustrates the relationship between execution time (hours) and F1 score (0.0 to 1.0) for the evaluated state-of-the-art tools. Each plot represents a specific taxonomic group, and each point within the plot corresponds to a different tool that was evaluated for that taxonomic group. The x-axis shows the execution time in hours, while the y-axis represents the F1 score. Only the tools that provided results for each taxonomic group were included in this Figure.

## List of abbreviations

BASTA: Basic Sequence Taxonomy Annotator;
CAMITAX: Critical Assessment of Metagenome Interpretation Taxonomy;
GCA: GenBank Assembly Genomes;
GCF: RefSeq Assembly Genomes;
HYMET: Hybrid Metagenomic Tool;
LCA: Lowest Common Ancestor;
TAMA: Taxonomy Analysis by Multiple Assignment;
TaxID: Taxonomy ID.

## Funding

This work has received funding from the FCT (Foundation for Science and Technology) under unit 00127-IEETA and through the project Advanced Genomic Data Processing in Portuguese FEGA Node (ref. 2023.14342.CPCA.A1; DOI: 10.54499/2023.14342.CPCA.A1). J.M.S. has received funding from the European Commission under grant agreement 101081813 (Genomic Data Infrastructure).

## References

1. Kim N, Ma J, Kim W, Kim J, Belenky P, Lee I. Genome-resolved metagenomics: a game changer for microbiome medicine. Experimental & Molecular Medicine 2024;56(7):1501–1512.
2. Martins IB, Miguel Silva J, Almeida JR. A comprehensive study of databases to assess the reliability of metagenomic tools. In: 2024 IEEE Conference on Computational Intelligence in Bioinformatics and Computational Biology (CIBCB); 2024. p. 1–6.
3. Simon HY, Siddle KJ, Park DJ, Sabeti PC. Benchmarking metagenomics tools for taxonomic classification. Cell 2019;178(4):779–794.
4. Lema NK, Gemeda MT, Woldesemayat AA. Recent Advances in Metagenomic Approaches, Applications, and Challenges. Current Microbiology 2023;80(11):347.
5. Kim D, Song L, Breitwieser FP, Salzberg SL. Centrifuge: rapid and sensitive classification of metagenomic sequences. Genome research 2016;26(12):1721–1729.
6. Wood DE, Salzberg SL. Kraken: ultrafast metagenomic sequence classification using exact alignments. Genome biology 2014;15(3):1–12.
7. Bremges A, Fritz A, McHardy AC. CAMITAX: Taxon labels for microbial genomes. GigaScience 2020;9(1):giz154.
8. Mallawaarachchi V, Lin Y. Accurate binning of metagenomic contigs using composition, coverage, and assembly graphs. Journal of Computational Biology 2022;29(12):1357–1376.
9. Ayling M, Clark MD, Leggett RM. New approaches for metagenome assembly with short reads. Briefings in bioinformatics 2020;21(2):584–594.
10. Wood DE, Lu J, Langmead B. Improved metagenomic analysis with Kraken 2. Genome biology 2019;20:1–13.
11. Xu R, Rajeev S, Salvador LC. The selection of software and database for metagenomics sequence analysis impacts the outcome of microbial profiling and pathogen detection. Plos one 2023;18(4):e0284031.
12. Breitwieser FP, Lu J, Salzberg SL. A review of methods and databases for metagenomic classification and assembly. Briefings in bioinformatics 2019;20(4):1125–1136.
13. Kieser S, Brown J, Zdobnov EM, Trajkovski M, McCue LA. ATLAS: a Snakemake workflow for assembly, annotation, and genomic binning of metagenome sequence data. BMC bioinformatics 2020;21:1–8.
14. Tadrent N, Dedeine F, Hervé V. SnakeMAGs: a simple, efficient, flexible and scalable workflow to reconstruct prokaryotic

genomes from metagenomes. F1000Research 2022;11.
15. Tamames J, Puente-Sánchez F. SqueezeMeta, a highly portable, fully automatic metagenomic analysis pipeline. Frontiers in microbiology 2019;9:425882.
16. Clarke EL, Taylor LJ, Zhao C, Connell A, Lee JJ, Fett B, et al. Sunbeam: an extensible pipeline for analyzing metagenomic sequencing experiments. Microbiome 2019;7:1–13.
17. Chaumeil PA, Mussig AJ, Hugenholtz P, Parks DH, GTDB-Tk: a toolkit to classify genomes with the Genome Taxonomy Database. Oxford University Press; 2020.
18. Buchfink B, Xie C, Huson DH. Fast and sensitive protein alignment using DIAMOND. Nature methods 2015;12(1):59–60.
19. Kahlke T, Ralph PJ. BASTA–Taxonomic classification of sequences and sequence bins using last common ancestor estimations. Methods in Ecology and Evolution 2019;10(1):100–103.
20. Ondov BD, Treangen TJ, Melsted P, Mallonee AB, Bergman NH, Koren S, et al. Mash: fast genome and metagenome distance estimation using MinHash. Genome biology 2016;17:1–14.
21. Menzel P, Ng KL, Krogh A. Fast and sensitive taxonomic classification for metagenomics with Kaiju. Nature communications 2016;7(1):11257.
22. Callahan BJ, McMurdie PJ, Rosen MJ, Han AW, Johnson AJA, Holmes SP. DADA2: High-resolution sample inference from Illumina amplicon data. Nature methods 2016;13(7):581–583.
23. Sim M, Lee J, Lee D, Kwon D, Kim J. TAMA: improved metagenomic sequence classification through meta-analysis. BMC bioinformatics 2020;21:1–17.
24. Ounit R, Wanamaker S, Close TJ, Lonardi S. CLARK: fast and accurate classification of metagenomic and genomic sequences using discriminative k-mers. BMC genomics 2015;16(1):1–13.
25. Shang J, Peng C, Liao H, Tang X, Sun Y. PhaBOX: a web server for identifying and characterizing phage contigs in metagenomic data. Bioinformatics Advances 2023;3(1):vbad101.
26. Shang J, Jiang J, Sun Y. Bacteriophage classification for assembled contigs using graph convolutional network. Bioinformatics 2021;37(Supplement_1):i25–i33.
27. Zhou Z, Martin C, Kosmopoulos JC, Anantharaman K. ViWrap: A modular pipeline to identify, bin, classify, and predict viral–host relationships for viruses from metagenomes. Imeta 2023;2(3):e118.
28. Auslander N, Gussow AB, Benler S, Wolf YI, Koonin EV. Seeker: alignment-free identification of bacteriophage genomes by deep learning. Nucleic acids research 2020;48(21):e121–e121.
29. Gałan W, Bąk M, Jakubowska M. Host taxon predictor-a tool for predicting taxon of the host of a newly discovered virus. Scientific reports 2019;9(1):3436.
30. Jiang G, Zhang J, Zhang Y, Yang X, Li T, Wang N, et al. DCiPatho: deep cross-fusion networks for genome scale identification of pathogens. Briefings in Bioinformatics 2023;24(4):bbad194.
31. Altschul SF, Gish W, Miller W, Myers EW, Lipman DJ. Basic local alignment search tool. Journal of molecular biology 1990;215(3):403–410.
32. Gruber-Vodicka HR, Seah BK, Pruesse E. phyloFlash: rapid small-subunit rRNA profiling and targeted assembly from metagenomes. Msystems 2020;5(5):10–1128.
33. Truong DT, Franzosa EA, Tickle TL, Scholz M, Weingart G, Pasolli E, et al. MetaPhlAn2 for enhanced metagenomic taxonomic profiling. Nature methods 2015;12(10):902–903.
34. Lui WW, Leung AW, Leung HC, Xin Y, Teng JL, Woo PC, et al. MegaPath-Nano: Accurate Compositional Analysis and Drug-level Antimicrobial Resistance Detection Software for Oxford Nanopore Long-read Metagenomics. In: 2020 IEEE International Conference on Bioinformatics and Biomedicine (BIBM) IEEE; 2020. p. 329–336.
35. Li H. Minimap2: pairwise alignment for nucleotide sequences. Bioinformatics 2018;34(18):3094–3100.
36. Liang X, Zhang J, Kim Y, Ho J, Liu K, Keenum I, et al. ARGem: a new metagenomics pipeline for antibiotic resistance genes: metadata, analysis, and visualization. Frontiers in Genetics 2023;14:1219297.
37. Prosperi M, Marini S. Karga: Multi-platform toolkit for k-mer-based antibiotic resistance gene analysis of high-throughput sequencing data. In: 2021 IEEE EMBS International Conference on Biomedical and Health Informatics (BHI) IEEE; 2021. p. 1–4.
38. Olawoye IB, Frost SD, Happi CT. The Bacteria Genome Pipeline (BAGEP): an automated, scalable workflow for bacteria genomes with Snakemake. PeerJ 2020;8:e10121.
39. Ondov BD, Starrett GJ, Sappington A, Kostic A, Koren S, Buck CB, et al. Mash Screen: high-throughput sequence containment estimation for genome discovery. Genome biology 2019;20:1–13.
40. Baker DN, Langmead B. Dashing: fast and accurate genomic distances with HyperLogLog. Genome biology 2019;20:1–12.
41. Besta M, Kanakagiri R, Mustafa H, Karasikov M, Rätsch G, Hoefler T, et al. Communication-efficient jaccard similarity for high-performance distributed genome comparisons. In: 2020 IEEE International Parallel and Distributed Processing Symposium (IPDPS) IEEE; 2020. p. 1122–1132.
42. Zhao X. BinDash, software for fast genome distance estimation on a typical personal laptop. Bioinformatics 2019;35(4):671–673.
43. Katz LS, Griswold T, Morrison SS, Caravas JA, Zhang S, den Bakker HC, et al. Mashtree: a rapid comparison of whole genome sequence files. Journal of Open Source Software 2019;4(44):10–21105.
44. Broder AZ. On the resemblance and containment of documents. In: Proceedings. Compression and Complexity of SEQUENCES 1997 (Cat. No. 97TB100171) IEEE; 1997. p. 21–29.
45. Pierce NT, Irber L, Reiter T, Brooks P, Brown CT. Large-scale sequence comparisons with sourmash. F1000Research 2019;8:1006.
46. Hernández-Salmerón JE, Moreno-Hagelsieb G. FastANI, Mash and Dashing equally differentiate between Klebsiella species. PeerJ 2022;10:e13784.
47. Hera MR, Liu S, Wei W, Rodriguez JS, Ma C, Koslicki D. Metagenomic functional profiling: to sketch or not to sketch? Bioinformatics 2024;40(Supplement_2):ii165–ii173.
48. Wu W, Li B, Chen L, Gao J, Zhang C. A review for weighted minhash algorithms. IEEE Transactions on Knowledge and Data Engineering 2020;34(6):2553–2573.
49. Sánchez-Reyes A, Fernández-López M. Sketched reference databases for genome-based taxonomy and comparative genomics. Brazilian Journal of Biology 2022;84:e256673.
50. Team MD, Mash Tutorials; 2023. Accessed: 2025-01-10. https://mash.readthedocs.io/en/latest/tutorials.html.
51. Kitts PA, Church DM, Thibaud-Nissen F, Choi J, Hem V, Sapojnikov V, et al. Assembly: a resource for assembled genomes at NCBI. Nucleic acids research 2016;44(D1):D73–D80.
52. Schoch CL, Ciufo S, Domrachev M, Hotton CL, Kannan S, Khovanskaya R, et al. NCBI Taxonomy: a comprehensive update on curation, resources and tools. Database 2020;2020:baaa062.
53. Li H. Minimap and miniasm: fast mapping and de novo assembly for noisy long sequences. Bioinformatics 2016;32(14):2103–2110.
54. Dong J, Liu X, Sadasivan H, Sitaraman S, Narayanasamy S. mm2-gb: GPU accelerated minimap2 for long read dna mapping. In: Proceedings of the 15th ACM International Conference on Bioinformatics, Computational Biology and Health Informatics; 2024. p. 1–9.
55. Langmead B, Wilks C, Antonescu V, Charles R. Scaling read aligners to hundreds of threads on general-purpose processors. Bioinformatics 2019;35(3):421–432.
56. Rosen G, Garbarine E, Caseiro D, Polikar R, Sokhansanj B. Metagenome Fragment Classification Using N-Mer Frequency Profiles. Advances in bioinformatics 2008;2008(1):205969.

57. Liu B, Gibbons T, Ghodsi M, Treangen T, Pop M. Accurate and fast estimation of taxonomic profiles from metagenomic shotgun sequences. Genome biology 2011;12:1–27.
58. Pruitt KD, Tatusova T, Maglott DR. NCBI reference sequences (RefSeq): a curated non-redundant sequence database of genomes, transcripts and proteins. Nucleic acids research 2007;35(suppl_1):D61–D65.
59. O'Leary NA, Wright MW, Brister JR, Ciufo S, Haddad D, McVeigh R, et al. Reference sequence (RefSeq) database at NCBI: current status, taxonomic expansion, and functional annotation. Nucleic acids research 2016;44(D1):D733–D745.
60. Sayers EW, Beck J, Bolton EE, Bourexis D, Brister JR, Canese K, et al. Database resources of the national center for biotechnology information. Nucleic acids research 2021;49(D1):D10.
61. Jesus TF, Ribeiro-Gonçalves B, Silva DN, Bortolaia V, Ramirez M, Carriço JA. Plasmid ATLAS: plasmid visual analytics and identification in high-throughput sequencing data. Nucleic acids research 2019;47(D1):D188–D194.
62. Burki F, Roger AJ, Brown MW, Simpson AG. The new tree of eukaryotes. Trends in ecology & evolution 2020;35(1):43–55.
63. Simmonds P, Adams MJ, Benkő M, Breitbart M, Brister JR, Carstens EB, et al. Virus taxonomy in the age of metagenomics. Nature Reviews Microbiology 2017;15(3):161–168.
64. Harris HM, Hill C. A place for viruses on the tree of life. Frontiers in Microbiology 2021;11:604048.

Manuscript Click here to access/download;Manuscript;HYMET.pdf

*GigaScience*, 2023, 1–10

doi: xx.xxxx/xxxx
**Manuscript in Preparation**
**Paper**

PAPER

# HYMET: A Hybrid Metagenomic Pipeline for Accurate and Efficient Taxonomic Classification

Inês Martins[1,*], Jorge Miguel Silva[1,*] and João Rafael Almeida[1]

[1]IEETA/DETI, LASI, University of Aveiro, Aveiro, Portugal

[*]inesbrancomartins@ua.pt; jorge.miguel.ferreira.silva@ua.pt

## Abstract

**Background:** Metagenomics has revolutionized the study of microbial communities. Despite many advances, the field still faces many challenges, such as high computational demands, limited accessibility of tools, and the lack of standardized benchmarking protocols. To address some of these issues, we developed HYMET (Hybrid Metagenomic Tool), a lightweight, mutation-tolerant pipeline designed for accurate and efficient taxonomic classification across all biological domains. HYMET integrates *k*-mer screening and alignment-based methods to improve computational efficiency and accuracy, particularly in handling genetic mutations.
**Findings:** HYMET demonstrates superior performance compared to existing tools, achieving high F1 scores across multiple taxonomic groups and taxonomic levels. It maintains robust accuracy even under mutation rates of up to 30%, with F1 scores above 0.8 for most domains. The pipeline processes most datasets in less than an hour, significantly faster than current state-of-the-art tools, while requiring minimal computational resources (2.82 GB for installation). HYMET's dynamic database construction and weighted Lowest Common Ancestor algorithm enhance its ability to handle diverse and fragmented metagenomic data, making it highly adaptable to different biological domains.
**Conclusions:** HYMET represents a significant advance in metagenomic analysis, offering a balance of precision, efficiency, and resistance to mutation. HYMET source code is fully available at https://github.com/ieeta-pt/HYMET.

**Key words**: Metagenomics, taxonomic classification, *k*-mer screening, alignment-based methods, computational efficiency, mutation resistance, hybrid pipeline.

## Introduction

Metagenomics is a rapidly evolving field that studies the collective genetic material of microorganisms within specific environments. It has revolutionized our understanding of microbial diversity, enabling discoveries in biotechnology, environmental monitoring, and human health [1, 2]. However, the analysis of metagenomic data is heavily based on metagenomic tools, whose effectiveness varies due to factors such as data quality, computational resources, and algorithmic design. Consequently, understanding the strengths and limitations of these tools is crucial for addressing the challenges of metagenomics and improving the current state-of-the-art [3, 4].

A primary challenge in metagenomics is the development of accurate methods for the taxonomic classification of organisms within a sample [5, 3]. Despite the creation of numerous general-purpose and specialized metagenomic tools, several significant hurdles persist. Computational demands pose a major constraint, as tools often require substantial memory and processing power, leading to impractical execution times for large datasets [6, 7, 5]. The sheer volume of metagenomic datasets demands highly efficient algorithms that can operate within reasonable requirements of compute power, which is particularly problematic when dealing with millions of sequencing reads [1, 8, 9, 10]. Furthermore, taxonomic assignment remains a critical challenge in metagenomic analysis, especially at lower taxonomic levels [10]. This issue is exacerbated by the limitations of reference databases, which often exhibit significant sampling bias towards well-studied organisms, while underrepresenting species that are difficult to culture in laboratory settings [6, 5]. This discrepancy results in high rates of

**Compiled on:** May 9, 2025.
Draft manuscript prepared by the author.

**Key Points**

- This study presents HYMET, a novel pipeline combining k-mer screening (Mash) and alignment-based methods (Minimap2) for comprehensive taxonomic classification across all biological domains, addressing critical limitations in current metagenomic analysis tools.
- HYMET was evaluated against nine state-of-the-art tools including comprehensive pipelines (SnakeMAGs, SqueezeMeta), general classifiers (BASTA, CAMITAX, TAMA), domain-specific tools (PhaBOX/ViWrap for viruses, PhyloFlash for rRNA), and antimicrobial resistance classifier MegaPath-Nano.
- The study employed a curated dataset of 26 203 genomes (14.76 GB) from NCBI RefSeq and GTDB, spanning all biological domains: Bacteria, Archaea, Eukarya (fungi, plants, vertebrates) and viruses, featuring simulated mutations (0-30%) for robustness testing.
- HYMET achieved superior accuracy (F1 > 0.9 for every domain vs < 0.5 in state-of-the-art tools), faster processing (2h vs BASTA/SqueezeMeta's 20h), and maintained robust performance (F1 > 0.8 at 30% mutations, viruses: 0.5) while requiring minimal resources (2.82 GB installation).
- HYMET overcomes taxonomic biases through dynamic database construction and a hybrid k-mer/alignment approach, outperforming specialized tools while maintaining universal applicability across all biological domains.

unclassified or misclassified reads, especially in complex environmental samples [4, 3, 2]. Compounding these challenges is the lack of standardized benchmarking protocols and datasets, which hinders objective comparisons of tool performance, as researchers frequently test tools on non-uniform datasets with inconsistent evaluation metrics [2, 11, 12]. Addressing these issues is crucial for advancing our understanding of complex microbial communities and developing efficient, user-friendly software solutions to analyze the enormous amounts of data generated by metagenomic research [6, 5]. These collective challenges directly motivate our core research question:

> *How can a next-generation metagenomic classification tool be designed and implemented to accurately identify taxa across all domains while maintaining high performance and efficiency?*

To address this question, in this paper, we present HYMET (Hybrid Metagenomic Tool), a lightweight, mutation-tolerant solution capable of accurate cross-domain classification. Our contribution extends beyond the pipeline itself to include a carefully curated standardized dataset representing all biological domains, specifically designed to enable rigorous benchmarking of metagenomic tools. HYMET combines several innovative features, including dynamic database construction, precise alignment algorithms, and user-friendly outputs, to deliver superior accuracy, speed, and usability compared to existing solutions. This work provides researchers with a powerful platform for comprehensive analysis of the microbial community and advances the field toward more standardized, reproducible metagenomic research by overcoming the current limitations of state-of-the-art tools.

## Background

In recent years, we have witnessed remarkable progress in metagenomics, particularly in the development of computational tools for taxonomic classification and functional analysis [3, 5, 6]. A dominant trend in current methodologies is the integration of established classification techniques into end-to-end pipelines, which streamline the entire analytical workflow, from raw sequencing data to biologically interpretable results [13]. Currently, the state-of-the-art landscape is populated by a rich ecosystem of interconnected tools, each offering unique capabilities and complementary approaches that collectively advance the field's analytical power. Among these, SnakeMAGs [14] stands out for its specialized focus on reconstructing prokaryotic genomes from Illumina sequencing reads, while SqueezeMeta [15] offers a fully automated and comprehensive solution for metagenomic data analysis [16, 13]. The first tool uses the Genome Taxonomy Database (GTDB) toolkit [17] for taxonomic assignment, leveraging conserved marker genes for analysis. On the other hand, SqueezeMeta uses DIAMOND [18] for alignment and the Lowest Common Ancestor (LCA) algorithm for taxonomic assignment [15].

Complementing these general-purpose pipelines, several lightweight tools have emerged to address specific needs in taxonomic assignment. The Basic Sequence Taxonomy Annotator (BASTA) [19] also employs the LCA algorithm for efficient sequence classification, while the Critical Assessment of Metagenome Interpretation Taxonomy (CAMITAX) [7] improves accuracy through the integration of multiple classification strategies for microbial genome assignment, including genome distance-based classification using Mash [20], Centrifuge [5] and Kaiju [21], that determines the interval-union LCA of gene-level assignments and 16S rRNA gene-based classification employing a naive Bayesian classifier method using Dada2 [22]. For more robust taxonomic profiling, the Taxonomy Analysis by Multiple Assignment (TAMA) tool [23] combines consensus classifications from established classifiers, including Kraken [10], CLARK [24], and Centrifuge, leveraging their complementary strengths.

The field has also seen the development of specialized tools that target specific metagenomic applications. Viral genomics is particularly well served by PhaBOX [25, 26] for viral contig characterization and ViWrap [27] for prediction of viral-host relationship, both providing valuable information on viral diversity and ecological interactions [28, 29, 30]. The first tool, PhaBOX, developed by Shang et al., combines gene prediction and alignment (DIAMOND) with taxonomic classification by semi-supervised learning method (PhaGCN [26]), based on sequence similarities and cluster sharing networks, and final assignments using the LCA. ViWrap, on the other hand, uses machine learning and sequence similarity searches to identify viral sequences and BLAST [31] to identify best hits against databases for taxonomic annotation and host prediction. For microbial community analysis, PhyloFlash [32], developed by Gruber-Vodicka et al., offers unique capabilities through its small subunit ribosomal RNA (SSU rRNA)-based approach, enabling both metagenomic profiling and high-resolution phylogenetic studies [33]. In the critical area of antimicrobial resistance surveillance, MegaPath-Nano [34] has emerged as an important tool for the comprehensive detection of resistance genes, which directly supports public health monitoring efforts, which uses Minimap2 [35] for global alignment and hash-based k-mer mapping, prioritizing global matching of large portions of sequences [36, 37].

Despite this technological progress, significant challenges impede the broader implementation of metagenomic tools in clinical and research settings. Implementation barriers represent a primary obstacle, with inadequate documentation and complex installation procedures frequently compromising tool accessibility and user

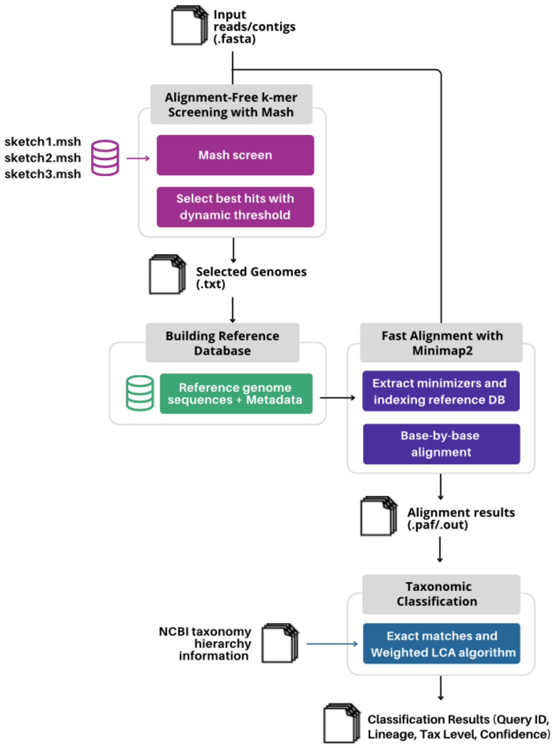


**Figure 1.** Overview of HYMET architecture.

adoption [38]. Computational constraints further limit practical application, as excessive memory and storage requirements hinder scalability. This is exemplified by SqueezeMeta, which demands over 500 GB of storage capacity and exhibits prohibitively long processing times [15], and BASTA, whose dependence on BLAST-based alignments creates computational bottlenecks that render it inefficient for time-sensitive analyses [19, 31]. A fundamental limitation stems from reference database dependencies rather than inherent tool restrictions. For instance, TAMA demonstrates robust classification capabilities in principle, but its default bacterial reference database necessarily limits its taxonomic scope to bacterial identification [23]. Similarly, independent evaluations of MegaPath-Nano confirm its strong performance in the detection of prokaryotic antimicrobial resistance, but note a reduced sensitivity when analyzing higher eukaryotes [34, 36]. This pattern of taxonomic bias is further evidenced in specialized tools such as PhaBOX and ViWrap, which, while excelling in virome analysis, lack versatility for broader metagenomic applications [27, 25]. PhyloFlash's reliance on small subunit rRNA analysis makes it fundamentally unsuitable for viral identification, as viruses lack ribosomal RNA genes [32].

## HYMET´s Workflow

HYMET, illustrated in Figure 1, is implemented using a combination of Perl for workflow orchestration and Python for module execution, with containerization facilitated by Docker to ensure reproducibility and portability. HYMET is also available as a Bioconda package under the name hymet, enabling easy installation and dependency management via the Conda ecosystem. The pipeline integrates two open-source tools: Mash [20] for rapid $k$-mer screening and Minimap2 [35] for precise sequence alignment. The installation and configuration details for HYMET are provided in Supplementary Sections 1 through 4.

## Alignment-Free $k$-mer Screening

The initial phase of HYMET utilizes Mash Screen [20] for rapid $k$-mer-based screening against pre-computed MinHash reference sketch databases [39, 40, 41, 42, 43]. Mash employs containment scores, as defined in Equation 1, to assess the proportion of a reference genome present in a query sequence [44, 45, 46]. The containment index $c_k(a, b)$ is estimated as:

$$c_k(a, b) \approx \frac{|S(A) \cap \pi(B)|}{|S(A)|}, \quad (1)$$

where $S(A)$ is the sketch of the reference genome $A$, and $\pi(B)$ represents the $k$-mers of the query sequence $B$. The containment index ranges from 0.0 to 1.0, with values closer to 1.0 indicating a higher proportion of $k$-mers from the reference genome present in the query. This metric is crucial for tasks such as contamination screening, reference genome selection, and the discovery of novel genomes, as it provides a rapid and unbiased estimate of sequence representation [39, 40, 44, 42, 47]. In this work, the screening process was optimized for computational efficiency by enabling parallel processing and applying a stringent 90% similarity threshold to retain only high-confidence matches, filtering out low-quality alignments. This containment-based approach prioritizes likely taxonomic candidates, reducing the search space and computational load for subsequent alignment-based stages [20, 39, 45]. As previously mentioned, to enable this screening, Mash relies on sketched databases, which are compact representations of genomic sequences. These databases are built using consistent $k$-mer hashing with MurmurHash3 [20, 48, 40, 49], which allows efficient comparison of query sequences against large collections of reference genomes.

### *Sketch and $k$-mer Size*

The construction of these sketch databases involves two key parameters: the $k$-mer size ($k$) and the sketch size ($s$). The choice of $k$-mer size is essential as it balances sensitivity and specificity. Smaller $k$-mers increase sensitivity for divergent genomes but may lead to random collisions, while larger $k$-mers reduce collisions but may miss subtle variations [50, 20, 40, 44, 43]. The optimal $k$-mer size is calculated as:

$$k = \log_{|\Sigma|}\left(\frac{n(1-q)}{q}\right), \quad (2)$$

where $|\Sigma|$ is the alphabet size (4 for nucleotides), $n$ is the genome size, and $q$ is the desired probability of observing a random $k$-mer. For example, smaller genomes (e.g. viruses) and highly variable taxa require smaller $k$-mer sizes (e.g. $k$-mer=15) to ensure specificity, while larger genomes (e.g. vertebrates) benefit from moderate $k$-mer sizes (e.g. $k$-mer=21) to balance sensitivity and computational efficiency [46, 50].The sketch size, which refers to the number of unique min-hashes retained for genomic sequence representation, also plays a critical role in determining the accuracy of distance and containment estimates [50, 20, 43, 45]. The error associated with containment estimation for a given sketch size, $s$, is proportional to:

$$\text{Error} \approx \sqrt{\frac{1}{s}}, \quad (3)$$

indicating that larger sketch sizes improve precision, but at the expense of greater computational resources [50]. For instance, smaller or highly fragmented genomes typically require larger sketch sizes to ensure sufficient genomic information is captured, while larger or less fragmented genomes can achieve accurate con-

**Table 1.** Reference sketched databases.

| Sketch | Content | Sketch Parameters | Sketch Size | Seed Value |
|---|---|---|---|---|
| sketch1.msh | RefSeq nucleotide release 88 | k=21, s=1000 | 1.2 GB | 0 |
| sketch2.msh | GTDB r202 Assembly Set, NCBI Complete Genomes Database + Custom databases (vertebrates, plants, invertebrates) | k=21, s=1000 | 883.25 MB | 42 |
| sketch3.msh | Custom databases (fungi, protozoa, archaea, virus) | k=15, s=5000 | 327.93 MB | 42 |

tainment estimates with smaller sketch sizes. This adaptive approach is supported by empirical evidence, with studies demonstrating that a sketch size of $s = 1000$ is generally adequate for obtaining precise similarity estimates in well-assembled genomes [20]. In fact, B. D. Ondov *et al.* established $s = 1000$ and $k$=21 as the default parameters in Mash, as they provide precise similarity estimates for well-assembled genomes [20]. However, for more divergent genomes, increasing the sketch size (e.g. $s = 5000$) can improve accuracy by capturing a more representative subset of genomic content [50, 43**?** , 46].

### *Reference Sketched Databases*

Following these design principles, we implemented a comprehensive database strategy combining both established public resources and a custom-built collection:

- **RefSeq Nucleotide Release 228:** Contains sketches of 162 138 organisms from RefSeq release 228, compressed using $k = 21$ and $s = 1000$, reducing the original 1.2 TB dataset to a compact representation [50].
- **GTDB r202 Assembly Set and NCBI Complete Genomes Database:** Combines 89 675 genomes from GTDB r202 and NCBI RefSeq (viruses, fungi, and bacteria/archaea), compressed with $k = 21$ and $s = 1000$ [49].
- **Custom Reference Database:** Enhances representation of underrepresented taxa by including 19 505 up-to-date genomes from NCBI RefSeq. For smaller genomes (e.g., archaea, fungi, protozoa, viruses), sketches were generated with $k = 15$ and $s = 5000$, while larger genomes (e.g., vertebrates, plants, invertebrates) used the default parameters ($k = 21$, $s = 1000$) [43].

These databases were grouped on the basis of shared seed values and parameters to optimize the screening efficiency. Table 1 summarizes their characteristics. All databases are publicly available through our project repository. For reproduction, detailed instructions are provided in Supplementary Material Section 2, Subsection "Reproducing Sketched Databases".

## Modular Reference Database Download

### *Candidate selection with dynamic threshold*

After running Mash Screen, the output can be extensive, potentially including a large number of candidate genomes with varying degrees of similarity to the query sequences. Downloading and analyzing this entire list would be computationally inefficient and could introduce noise into subsequent analyses. On the other hand, setting an arbitrarily high static threshold might exclude important reference genomes, leading to incomplete coverage of the query sequences. To address these challenges, HYMET introduces a dynamic thresholding mechanism to identify the most relevant candidate genomes and to create a targeted, input-specific database. A dynamic thresholding algorithm identifies relevant candidate genomes by iteratively adjusting a containment score threshold, starting from a preset value and reducing it by 0.02 until a minimum candidate count (3.25 times the number of input queries) is met or a lower limit of 0.71 is reached.

### *Genome Retrieval*

Following the selection of candidate genomes, their format was analyzed to enable efficient mapping and retrieval. These genomes were identified using RefSeq (GCF) and Genbank Assembly Genomes (GCA) accession numbers, unique identifiers assigned by NCBI. Thus, the NCBI Assembly database [51] was selected as the primary resource for constructing the reference database [2, 52]. To optimize the process, summary files from the NCBI Assembly database were downloaded, providing efficient access to metadata. A custom script was developed to map candidate genomes to these files using accession numbers. This script extracted the base accession number (e.g. "000169215") to ensure compatibility between different assembly versions (e.g.: GCF_000169215.1, GCF_000169215.2), preventing retrieval failures due to version updates [51]. Genomes were downloaded and decompressed from NCBI FTP server and saved locally in FASTA format. To enhance efficiency, the script employed ThreadPoolExecutor for parallel downloads, allowing up to 64 concurrent threads, and incorporated retry logic with exponential backoff to handle transient network errors. The taxonomy IDs (TaxID) of the assembly files were stored alongside the accession numbers and sequence identifiers, creating a comprehensive reference linking each genome to its taxonomic and sequence-level information [51, 52].

## Fast Alignment

In the second processing stage, HYMET employs Minimap2 for efficient and precise sequence alignment. This choice was motivated by Minimap2's adaptive scoring system, and global alignment capabilities enable accurate mapping even with highly divergent sequences or incomplete reads, making the pipeline particularly resilient to common metagenomic challenges such as mutation-rich or fragmented samples [34, 35]. The pipeline uses minimizers to index reference sequences, enabling the rapid identification of alignment regions [53]. This was executed with the `-x asm10` parameter, which is optimized for genome-to-genome alignment and allows up to 10% sequence divergence, which is equivalent to a 90% identity threshold, ensuring robust alignment even in the presence of significant genetic differences [54, 35]. The results are saved in a PAF file, providing essential alignment details such as sequence IDs, lengths, positions, and mapping quality [35, 55].

## Taxonomic Assignment

HYMET uses a hybrid taxonomic assignment strategy, combining the LCA algorithm with a weighted approach based on alignment coverage [23, 10, 24]. For exact matches, the reference's taxonomic lineage is directly assigned with a confidence score of 1.0. On the other hand, for non-exact matches, the weights for each TaxID are calculated according to:

$$\text{Weight} = \text{Coverage} \times \text{Abundance}, \tag{4}$$

where *Coverage* is the proportion of the query aligned with the reference and *Abundance* is the reference's frequency in the dataset. The most weighted TaxID at each taxonomic level is selected, and the confidence score is derived as the product of confidence values

**Table 2.** Composition of the test and validation dataset

| Domain/Group | Number of GCFs | Size (GB) |
|---|---|---|
| Viruses | 1 498 | 0.05 |
| Other Vertebrates | 43 | 2.83 |
| Vertebrate Mammals | 23 | 2.29 |
| Protozoa | 12 | 0.03 |
| Plants | 19 | 1.02 |
| Invertebrates | 43 | 1.14 |
| Fungi | 63 | 0.15 |
| Bacteria | 24 271 | 7.23 |
| Archaea | 231 | 0.05 |
| **Total** | **26 203** | **14.76** |

across ranks:

$$\text{Confidence Score} = \prod_{i=1}^{n} \text{Confidence at Rank}_i, \quad (5)$$

where $n$ is the number of ranks. This ensures higher consistency across ranks results in higher confidence scores. The final output includes the query identifier, taxonomic lineage (kingdom to strain), most specific rank, and a confidence score (0.0 to 1.0), reflecting classification reliability [24, 56, 57].

## Materials and Methods

All analyses, including tool evaluation, development, and validation, were conducted on a high-performance Linux-based virtual machine with 2 TB storage and 250 GB RAM. HYMET's performance was assessed using precision and F1 score metrics for classifying organisms across the three domains of life, considering taxonomic levels (kingdom to species) and mutation rates (0% to 30%). The analysis also examined the relationship between F1 scores, execution time, and resource usage (CPU and memory), ensuring a comprehensive evaluation of accuracy and efficiency. The same methodology was applied to the other current state-of-the-art tools described in Section to ensure a consistent comparison. Detailed instructions for reproducing the benchmarking of these tools are provided in Supplementary Section 5.

### *Test and Validation Dataset*

The test dataset was derived from the NCBI RefSeq Assembly database (last modified: 13 October 2024), chosen for its curated and validated sequences [58, 2, 59]. Assembly summary files for all biological domains and viruses were downloaded and 10% of the entries were randomly selected based on GCF accession numbers. For each GCF, 10% of its genome sequences were further sampled to ensure proportional representation and mimic the fragmentation of metagenomic data [60]. This approach resulted in a diverse and representative dataset, as detailed in Table 2. For dataset replication, complete instructions and scripts are provided in the Supplementary Material Section 3, Subsection"Replicating the Benchmark Dataset".

## Results

### *Performance Evaluation*

HYMET demonstrates superior classification performance compared to other state-of-the-art tools, as evidenced by consistently high F1 scores across diverse taxonomic groups (Figure 2; Supplementary Tables 1–8). While most state-of-the-art tools struggle with F1 scores below 0.5, HYMET stands out, particularly at higher taxonomic levels (kingdom to class), where it achieves perfect F1

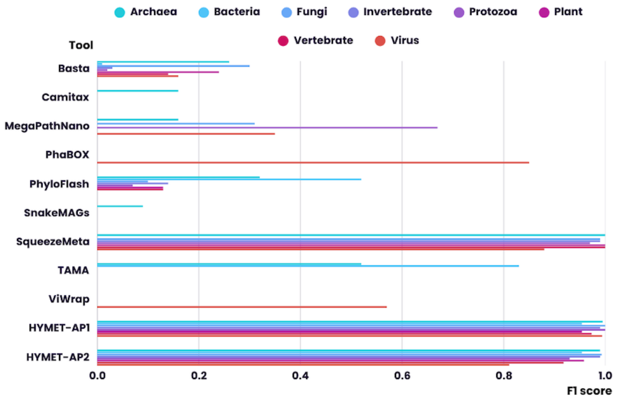


**Figure 2.** F1 scores achieved by various taxonomic classification tools, including both state-of-the-art tools and HYMET, across different taxonomic groups. The x-axis represents the F1 scores, ranging from 0 to 1, while the y-axis lists the evaluated tools. Each bar is color-coded to indicate the corresponding taxonomic group. This data is based on analysis with 0% mutation rate.

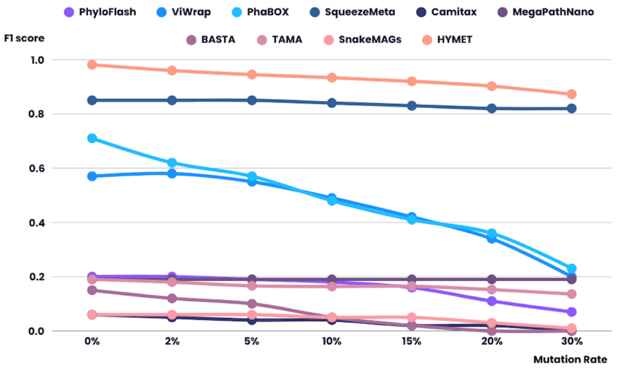


**Figure 3.** Performance of the state-of-the-art tools and HYMET as the mutation rate increases. The x-axis represents the mutation rate (ranging from 0% to 30%), while the y-axis shows the F1 score. Each curve on the graph corresponds to a different tool.

scores (1.0) for vertebrate mammals, plants, and bacteria. Performance decreases slightly to 0.8–0.9 at the species level, but remains robust for archaea and microbial eukaryotes (fungi, protozoa), with F1 scores exceeding 0.95 throughout all levels. For viruses, accuracy remains high at the species and kingdom levels (above 0.9), with a minor decline at intermediate ranks.

### *Mutation Resilience*

HYMET's performance proves exceptionally stable under varying mutation rates (0–30%), outperforming all benchmarked tools in both accuracy and consistency (Figure 3). Viral classification shows a progressive decline at extreme mutations (F1 scores approximately 0.5 at thirty percent), while archaea, invertebrates and fungi maintain F1 scores over 0.9. Other groups show only minor, non-significant reductions, staying above 0.8 (Figure 4). This contrasts with competing tools, where their scores decline as the mutation rate increases (Supplementary Figures 1-2).

### *Computational Efficiency*

The analysis of Figure 5 and Supplementary Figure 3 reveals significant variations in execution time. Tools like PhyloFlash, PhaBOX, TAMA and ViWrap demonstrate rapid processing with execution times under one hour, while SqueezeMeta offers good performance at the cost of longer computational times. In contrast, BASTA consistently shows the longest execution times, approaching more than 20 hours for every domain, with correspondingly low performance across different taxonomic domains. On the other hand,

**Table 3.** Computational Resources required by each tool.

| Tool | Resources (GB) | CPU Usage (%) | Memory Usage (%) |
|---|---|---|---|
| **HYMET** | **2.82 (+10-50)** | **13.04** | **13.65** |
| BASTA | 40 | 12.72 | 3.05 |
| CAMITAX | 33 | 22.37 | 21.08 |
| MegaPath-Nano | 71 | 28.86 | 15.71 |
| PhaBOX | 2.8 | 69.16 | 3.67 |
| PhyloFlash | 16 | 34.21 | 3.86 |
| SnakeMAGs | 67 | 20.17 | 3.57 |
| Squeezemeta | 403 | 54.92 | 17.03 |
| ViWrap | 2 | 40.65 | 8.76 |
| TAMA | 285 | 51.77 | 32.47 |

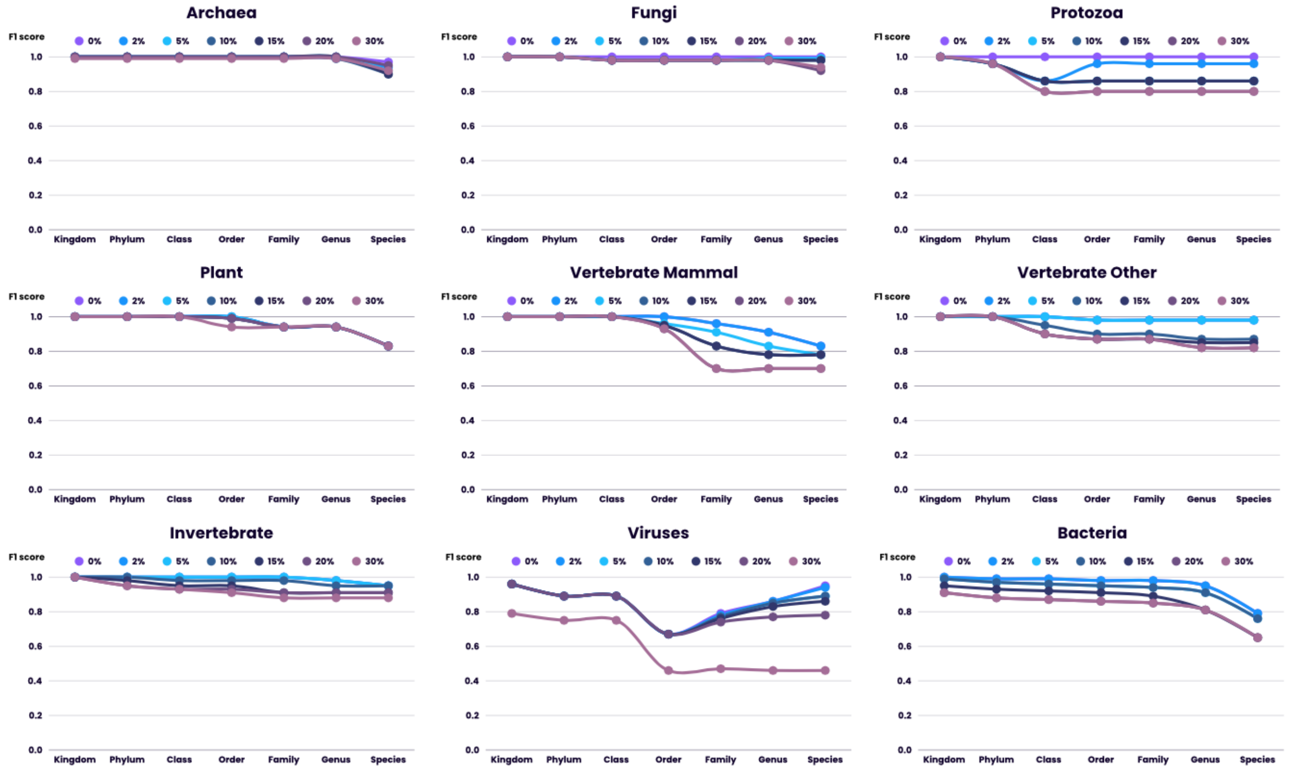


**Figure 4.** Performance of HYMET across taxonomic levels for different taxonomic groups as mutation rates increase (0% to 30%). The x-axis shows taxonomic levels (kingdom to species), and the y-axis represents the F1 score. Each curve corresponds to a specific mutation rate, and each graph focuses on a particular taxonomic group.

HYMET processes datasets in approximately 2 hours, significantly faster than existing tools, while maintaining an overall F1 score of approximately 0.95. Execution times vary by taxonomic group, with larger eukaryotes and bacteria taking more than 150 minutes, while microbial eukaryotes, archaea, and viruses are processed in approximately 10 minutes. In terms of computacional resource requirements, represented in Table 3, HYMET requires only 2.82 GB for installation and configuration, with dynamically downloaded reference databases typically ranging from 10 to 50 GB, depending on metagenomic composition. This balance of speed, accuracy, and resource efficiency positions HYMET as a leading tool for metagenomic analysis.

## Discussion

HYMET's success stems from its innovative design and methodological advances, setting it apart from existing tools and overcoming current limitations. A key innovation is the use of Mash Screen for sequence pre-filtering, which represents a novel contribution to the field, as no existing work has utilized Mash for screening purposes in this context [7, 49, 61], enhancing efficiency and accuracy in candidate selection. Moreover, HYMET employs a dynamic threshold selection methodology that adapts to the specific characteristics of each sample, ensuring optimized performance across diverse datasets. This is a significant improvement over fixed-threshold approaches, such as those used in CAMITAX, which rely on static thresholds for candidate selection, leading to the exclusion of potentially relevant candidate genomes [7, 61]. For taxonomic classification, HYMET implements a weighted LCA algorithm, which improves the widely used LCA method among the state-of-the-art tools [15, 25, 10], with the incorporation of an adaptive scoring methodology. This refinement reduces penalties for single-nucleotide mismatches, which are common in highly mutated sequences, and excludes low-confidence reads prior to taxonomic assignment. This strategy, similar to that used in TAMA, significantly improves classification precision, particularly at lower taxonomic levels, where closely related species can be difficult to distinguish [23, 24, 10].

Another important feature of HYMET is its modular database system, which provides flexibility and adaptability to different biological domains, demonstrating remarkable efficiency in resource utilization. This allows operation on standard computing infras-

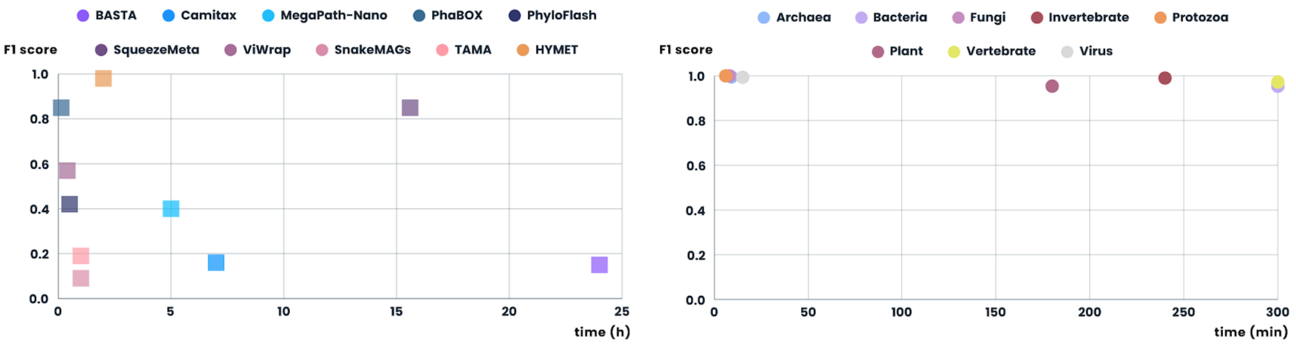


**Figure 5.** Relationship between execution time (in hours) and F1 score (ranging from 0.0 to 1.0) across various domains for HYMET and state-of-the-art tools, as well as HYMET's relationship between execution time (in minutes) and F1 score across biological domains.

tructure, reducing the dependency on high-performance computing resources, distinguishing it from state-of-the-art tools that depend on fixed and extensive reference databases [23, 15, 14]. For instance, Squeezemeta (403 GB) and TAMA (285 GB) require significantly higher memory. This advantage is directly related to the pipeline's scalability, which adapts to the complexity and size of the query sample. The reference database is dynamically generated based on the sample's content, resulting in execution times and resource requirements that scale linearly with genome complexity. For example, processing viral or small genomes is significantly faster due to the reduced size of candidate genomes to be downloaded and aligned. In contrast, while processing complex eukaryotic genomes increases computation time, HYMET remains faster than state-of-the-art tools like BASTA, Squeezemeta, and MegaPath-Nano. Unlike these tools, which rely on predefined reference databases, HYMET's dynamic approach allows for more efficient adaptation to the sample's content, minimizing resource waste, and improving overall performance.

A key strength of HYMET is its two-stage mutation-handling framework. During screening, the custom sketched database, built using optimized parameters ($k = 15$, $s = 5000$) improves genomic diversity representation and sensitivity to mutations. These settings increased candidate matches by approximately 30% for sequences with 30% mutations and reduced false negatives by 25% in viral sequences during the screening stage. In the alignment stage, Minimap2's seed-chain-align approach identifies exact matches and extends them, tolerating gaps and mismatches. This ensures robust performance, with F1 scores greater than 0.5 for viral sequences with mutation rates of 20–30% and greater than 0.8 for other domains with high mutation rates [20, 35]. This combination of an optimized database and advanced alignment makes HYMET highly effective in handling mutations.

Overall, HYMET demonstrates robust performance across diverse biological domains, although its accuracy and efficiency vary depending on the complexity of the organisms being analyzed. For bacteria, despite the challenges posed by a large test dataset of ~20 000 genomes, HYMET achieves high accuracy at higher taxonomic levels, though species-level classification is more challenging due to genetic similarity among closely related species. The sheer volume of sequences also increases computational demands, suggesting that a more balanced dataset with fewer representative genomes could improve consistency and reduce execution time. Within Eukarya, microbial eukaryotes such as fungi and protozoa achieve high accuracy across all taxonomic levels, benefiting from distinct genetic signatures and well-documented reference genomes, just like archaeal organisms. In contrast, larger eukaryotic organisms, such as vertebrates and plants, show reduced accuracy at the species level due to their complex genomes, higher intraspecies diversity, and closer evolutionary relationships [62]. Uneven representation in reference databases further complicates classification, as bias towards well-studied species limits the diversity of reference sequences [58, 17]. For viruses, HYMET excels at species-level classification due to the distinct genetic signatures of viral strains, but accuracy decreases at higher taxonomic levels, reflecting the polyphyletic nature of viral evolution and the lack of a unified taxonomic framework [63, 64].

Despite the pipeline's robust performance in metagenomic classification, particularly at higher taxonomic levels, we observed a decrease in accuracy at the genus and species levels, specially as mutation increases. This reduction in precision can be attributed to several factors. Firstly, bias in reference databases is a significant consideration. The uneven representation of species and genera in databases can result in skewed classifications, favoring more extensively documented organisms. Additionally, a significant drawback encountered during testing was the challenge of genome downloads, particularly with the RefSeq sketched database [58]. As an older version, some genomes were no longer available in the assembly files, while others had non-functional links or had been suppressed. Quantitative analysis revealed an overall 6.04% failure rate in genome retrieval across all datasets, which significantly impacted the pipeline's performance. This issue likely contributed to the reduced F1 scores observed at the genus and species levels. This issue leads to the non-representation of certain organisms in the reference database, even when detected as candidates by Mash Screen. Consequently, this may explain the reduced F1 scores observed at the genus and species levels. These limitations underscore the importance of maintaining up-to-date and comprehensive databases, as well as the need to develop strategies for handling missing or inaccessible genomes to improve classification accuracy across all taxonomic levels.

## Conclusion

HYMET represents a significant methodological advancement in metagenomic analysis through its innovative integration of $k$-mer screening with precise alignment-based classification. The pipeline's core strength lies in its modular architecture, which combines dynamic Mash-based filtering, weighted LCA algorithms, and an optimized reference database system to achieve robust taxonomic assignment across diverse biological domains. This integrated approach addresses critical limitations in current tools, particularly in handling highly divergent sequences and uneven reference databases, while maintaining computational efficiency on standard infrastructure. Future development of HYMET should prioritize the implementation of a dynamically updated reference database system incorporating multiple genomic repositories to address current limitations in genome retrieval and taxonomic coverage, and integration of machine learning classifiers, particularly deep neural networks, to improve discrimination at lower taxonomic ranks where sequence similarity challenges conventional methods [29, 30]. In the long term, transforming HYMET into

a comprehensive metagenomic analysis platform, encompassing quality control, assembly, annotation, and interactive visualization, would address critical gaps in current end-to-end solutions [16, 13]. These strategic improvements would position HYMET as both a robust taxonomic classifier and a versatile framework for reproducible large-scale metagenomic studies across basic and translational research applications.

## Availability of source code and requirements

- Project name: HYMET (Hybrid Metagenomic Tool)
- Project home page: https://github.com/ieeta-pt/HYMET
- Operating system(s): Linux
- Programming language: Perl, Python, Bash
- Other requirements: Docker, Conda
- License: No restrictions.

## Data Availability

The Supplementary Material accompanying this article includes comprehensive instructions for reproducibility, encompassing all aspects of data analysis, tool utilization, environment configuration, script execution, and supplementary results.

## Additional Files

***Supplementary Tab. S1 to S8.*** These tables presents the performance results of HYMET and each state-of-the-art tool, including Precision, Recall, and F1 score, for different taxonomic groups categorized by taxonomic levels (Kingdom, Phylum, Class, Order, Family, Genus, Species) with mutation rates of 0%. Each table represents a specific taxonomic group, and each row within the table corresponds to a different tool. The columns of each table are organized by taxonomic level, with the corresponding performance metrics listed for each level.

***Supplementary Tab. S9 to S15.*** These tables presents the performance results of HYMET, including Precision, Recall, and F1 score, across all taxonomic groups for mutation rates ranging from 0% to 30%. Each row corresponds to a specific taxonomic group, while the columns are organized by taxonomic levels (Kingdom, Phylum, Class, Order, Family, Genus, Species). The performance metrics (F1 score, Precision, and Recall) are listed for each taxonomic level, with each table representing a distinct mutation rate.

***Supplementary Fig. S1 and S2.*** This figure illustrates the performance of the state-of-the-art tools as the mutation rate increases, across different taxonomic levels for various taxonomic groups. The x-axis represents the taxonomic levels (Kingdom, Phylum, Class, Order, Family, Genus, Species), while the y-axis shows the F1 score. Each curve on the graph corresponds to a different mutation rate, ranging from 0% to 30%, and each graph represents the performance of one tool within a specific taxonomic group.

***Supplementary Fig. S3.*** This figure illustrates the relationship between execution time (hours) and F1 score (0.0 to 1.0) for the evaluated state-of-the-art tools. Each plot represents a specific taxonomic group, and each point within the plot corresponds to a different tool that was evaluated for that taxonomic group. The x-axis shows the execution time in hours, while the y-axis represents the F1 score. Only the tools that provided results for each taxonomic group were included in this Figure.

## List of abbreviations

BASTA: Basic Sequence Taxonomy Annotator;
CAMITAX: Critical Assessment of Metagenome Interpretation Taxonomy;
GCA: GenBank Assembly Genomes;
GCF: RefSeq Assembly Genomes;
HYMET: Hybrid Metagenomic Tool;
LCA: Lowest Common Ancestor;
TAMA: Taxonomy Analysis by Multiple Assignment;
TaxID: Taxonomy ID.

## Funding

This work has received funding from the FCT (Foundation for Science and Technology) under unit 00127-IEETA and through the project Advanced Genomic Data Processing in Portuguese FEGA Node (ref. 2023.14342.CPCA.A1; DOI: 10.54499/2023.14342.CPCA.A1). J.M.S. has received funding from the European Commission under grant agreement 101081813 (Genomic Data Infrastructure).

## References

1. Kim N, Ma J, Kim W, Kim J, Belenky P, Lee I. Genome-resolved metagenomics: a game changer for microbiome medicine. Experimental & Molecular Medicine 2024;56(7):1501–1512.
2. Martins IB, Miguel Silva J, Almeida JR. A comprehensive study of databases to assess the reliability of metagenomic tools. In: 2024 IEEE Conference on Computational Intelligence in Bioinformatics and Computational Biology (CIBCB); 2024. p. 1–6.
3. Simon HY, Siddle KJ, Park DJ, Sabeti PC. Benchmarking metagenomics tools for taxonomic classification. Cell 2019;178(4):779–794.
4. Lema NK, Gemeda MT, Woldesemayat AA. Recent Advances in Metagenomic Approaches, Applications, and Challenges. Current Microbiology 2023;80(11):347.
5. Kim D, Song L, Breitwieser FP, Salzberg SL. Centrifuge: rapid and sensitive classification of metagenomic sequences. Genome research 2016;26(12):1721–1729.
6. Wood DE, Salzberg SL. Kraken: ultrafast metagenomic sequence classification using exact alignments. Genome biology 2014;15(3):1–12.
7. Bremges A, Fritz A, McHardy AC. CAMITAX: Taxon labels for microbial genomes. GigaScience 2020;9(1):giz154.
8. Mallawaarachchi V, Lin Y. Accurate binning of metagenomic contigs using composition, coverage, and assembly graphs. Journal of Computational Biology 2022;29(12):1357–1376.
9. Ayling M, Clark MD, Leggett RM. New approaches for metagenome assembly with short reads. Briefings in bioinformatics 2020;21(2):584–594.
10. Wood DE, Lu J, Langmead B. Improved metagenomic analysis with Kraken 2. Genome biology 2019;20:1–13.
11. Xu R, Rajeev S, Salvador LC. The selection of software and database for metagenomics sequence analysis impacts the outcome of microbial profiling and pathogen detection. Plos one 2023;18(4):e0284031.
12. Breitwieser FP, Lu J, Salzberg SL. A review of methods and databases for metagenomic classification and assembly. Briefings in bioinformatics 2019;20(4):1125–1136.
13. Kieser S, Brown J, Zdobnov EM, Trajkovski M, McCue LA. ATLAS: a Snakemake workflow for assembly, annotation, and genomic binning of metagenome sequence data. BMC bioinformatics 2020;21:1–8.
14. Tadrent N, Dedeine F, Hervé V. SnakeMAGs: a simple, efficient, flexible and scalable workflow to reconstruct prokaryotic

genomes from metagenomes. F1000Research 2022;11.
15. Tamames J, Puente-Sánchez F. SqueezeMeta, a highly portable, fully automatic metagenomic analysis pipeline. Frontiers in microbiology 2019;9:425882.
16. Clarke EL, Taylor LJ, Zhao C, Connell A, Lee JJ, Fett B, et al. Sunbeam: an extensible pipeline for analyzing metagenomic sequencing experiments. Microbiome 2019;7:1–13.
17. Chaumeil PA, Mussig AJ, Hugenholtz P, Parks DH, GTDB-Tk: a toolkit to classify genomes with the Genome Taxonomy Database. Oxford University Press; 2020.
18. Buchfink B, Xie C, Huson DH. Fast and sensitive protein alignment using DIAMOND. Nature methods 2015;12(1):59–60.
19. Kahlke T, Ralph PJ. BASTA–Taxonomic classification of sequences and sequence bins using last common ancestor estimations. Methods in Ecology and Evolution 2019;10(1):100–103.
20. Ondov BD, Treangen TJ, Melsted P, Mallonee AB, Bergman NH, Koren S, et al. Mash: fast genome and metagenome distance estimation using MinHash. Genome biology 2016;17:1–14.
21. Menzel P, Ng KL, Krogh A. Fast and sensitive taxonomic classification for metagenomics with Kaiju. Nature communications 2016;7(1):11257.
22. Callahan BJ, McMurdie PJ, Rosen MJ, Han AW, Johnson AJA, Holmes SP. DADA2: High-resolution sample inference from Illumina amplicon data. Nature methods 2016;13(7):581–583.
23. Sim M, Lee J, Lee D, Kwon D, Kim J. TAMA: improved metagenomic sequence classification through meta-analysis. BMC bioinformatics 2020;21:1–17.
24. Ounit R, Wanamaker S, Close TJ, Lonardi S. CLARK: fast and accurate classification of metagenomic and genomic sequences using discriminative k-mers. BMC genomics 2015;16(1):1–13.
25. Shang J, Peng C, Liao H, Tang X, Sun Y. PhaBOX: a web server for identifying and characterizing phage contigs in metagenomic data. Bioinformatics Advances 2023;3(1):vbad101.
26. Shang J, Jiang J, Sun Y. Bacteriophage classification for assembled contigs using graph convolutional network. Bioinformatics 2021;37(Supplement_1):i25–i33.
27. Zhou Z, Martin C, Kosmopoulos JC, Anantharaman K. ViWrap: A modular pipeline to identify, bin, classify, and predict viral–host relationships for viruses from metagenomes. Imeta 2023;2(3):e118.
28. Auslander N, Gussow AB, Benler S, Wolf YI, Koonin EV. Seeker: alignment-free identification of bacteriophage genomes by deep learning. Nucleic acids research 2020;48(21):e121–e121.
29. Gałan W, Bąk M, Jakubowska M. Host taxon predictor-a tool for predicting taxon of the host of a newly discovered virus. Scientific reports 2019;9(1):3436.
30. Jiang G, Zhang J, Zhang Y, Yang X, Li T, Wang N, et al. DCiPatho: deep cross-fusion networks for genome scale identification of pathogens. Briefings in Bioinformatics 2023;24(4):bbad194.
31. Altschul SF, Gish W, Miller W, Myers EW, Lipman DJ. Basic local alignment search tool. Journal of molecular biology 1990;215(3):403–410.
32. Gruber-Vodicka HR, Seah BK, Pruesse E. phyloFlash: rapid small-subunit rRNA profiling and targeted assembly from metagenomes. Msystems 2020;5(5):10–1128.
33. Truong DT, Franzosa EA, Tickle TL, Scholz M, Weingart G, Pasolli E, et al. MetaPhlAn2 for enhanced metagenomic taxonomic profiling. Nature methods 2015;12(10):902–903.
34. Lui WW, Leung AW, Leung HC, Xin Y, Teng JL, Woo PC, et al. MegaPath-Nano: Accurate Compositional Analysis and Drug-level Antimicrobial Resistance Detection Software for Oxford Nanopore Long-read Metagenomics. In: 2020 IEEE International Conference on Bioinformatics and Biomedicine (BIBM) IEEE; 2020. p. 329–336.
35. Li H. Minimap2: pairwise alignment for nucleotide sequences. Bioinformatics 2018;34(18):3094–3100.
36. Liang X, Zhang J, Kim Y, Ho J, Liu K, Keenum I, et al. ARGem: a new metagenomics pipeline for antibiotic resistance genes: metadata, analysis, and visualization. Frontiers in Genetics 2023;14:1219297.
37. Prosperi M, Marini S. Karga: Multi-platform toolkit for k-mer-based antibiotic resistance gene analysis of high-throughput sequencing data. In: 2021 IEEE EMBS International Conference on Biomedical and Health Informatics (BHI) IEEE; 2021. p. 1–4.
38. Olawoye IB, Frost SD, Happi CT. The Bacteria Genome Pipeline (BAGEP): an automated, scalable workflow for bacteria genomes with Snakemake. PeerJ 2020;8:e10121.
39. Ondov BD, Starrett GJ, Sappington A, Kostic A, Koren S, Buck CB, et al. Mash Screen: high-throughput sequence containment estimation for genome discovery. Genome biology 2019;20:1–13.
40. Baker DN, Langmead B. Dashing: fast and accurate genomic distances with HyperLogLog. Genome biology 2019;20:1–12.
41. Besta M, Kanakagiri R, Mustafa H, Karasikov M, Rätsch G, Hoefler T, et al. Communication-efficient jaccard similarity for high-performance distributed genome comparisons. In: 2020 IEEE International Parallel and Distributed Processing Symposium (IPDPS) IEEE; 2020. p. 1122–1132.
42. Zhao X. BinDash, software for fast genome distance estimation on a typical personal laptop. Bioinformatics 2019;35(4):671–673.
43. Katz LS, Griswold T, Morrison SS, Caravas JA, Zhang S, den Bakker HC, et al. Mashtree: a rapid comparison of whole genome sequence files. Journal of Open Source Software 2019;4(44):10–21105.
44. Broder AZ. On the resemblance and containment of documents. In: Proceedings. Compression and Complexity of SEQUENCES 1997 (Cat. No. 97TB100171) IEEE; 1997. p. 21–29.
45. Pierce NT, Irber L, Reiter T, Brooks P, Brown CT. Large-scale sequence comparisons with sourmash. F1000Research 2019;8:1006.
46. Hernández-Salmerón JE, Moreno-Hagelsieb G. FastANI, Mash and Dashing equally differentiate between Klebsiella species. PeerJ 2022;10:e13784.
47. Hera MR, Liu S, Wei W, Rodriguez JS, Ma C, Koslicki D. Metagenomic functional profiling: to sketch or not to sketch? Bioinformatics 2024;40(Supplement_2):ii165–ii173.
48. Wu W, Li B, Chen L, Gao J, Zhang C. A review for weighted minhash algorithms. IEEE Transactions on Knowledge and Data Engineering 2020;34(6):2553–2573.
49. Sánchez-Reyes A, Fernández-López M. Sketched reference databases for genome-based taxonomy and comparative genomics. Brazilian Journal of Biology 2022;84:e256673.
50. Team MD, Mash Tutorials; 2023. Accessed: 2025-01-10. https://mash.readthedocs.io/en/latest/tutorials.html.
51. Kitts PA, Church DM, Thibaud-Nissen F, Choi J, Hem V, Sapojnikov V, et al. Assembly: a resource for assembled genomes at NCBI. Nucleic acids research 2016;44(D1):D73–D80.
52. Schoch CL, Ciufo S, Domrachev M, Hotton CL, Kannan S, Khovanskaya R, et al. NCBI Taxonomy: a comprehensive update on curation, resources and tools. Database 2020;2020:baaa062.
53. Li H. Minimap and miniasm: fast mapping and de novo assembly for noisy long sequences. Bioinformatics 2016;32(14):2103–2110.
54. Dong J, Liu X, Sadasivan H, Sitaraman S, Narayanasamy S. mm2-gb: GPU accelerated minimap2 for long read dna mapping. In: Proceedings of the 15th ACM International Conference on Bioinformatics, Computational Biology and Health Informatics; 2024. p. 1–9.
55. Langmead B, Wilks C, Antonescu V, Charles R. Scaling read aligners to hundreds of threads on general-purpose processors. Bioinformatics 2019;35(3):421–432.
56. Rosen G, Garbarine E, Caseiro D, Polikar R, Sokhansanj B. Metagenome Fragment Classification Using N-Mer Frequency Profiles. Advances in bioinformatics 2008;2008(1):205969.

57. Liu B, Gibbons T, Ghodsi M, Treangen T, Pop M. Accurate and fast estimation of taxonomic profiles from metagenomic shotgun sequences. Genome biology 2011;12:1–27.
58. Pruitt KD, Tatusova T, Maglott DR. NCBI reference sequences (RefSeq): a curated non-redundant sequence database of genomes, transcripts and proteins. Nucleic acids research 2007;35(suppl_1):D61–D65.
59. O'Leary NA, Wright MW, Brister JR, Ciufo S, Haddad D, McVeigh R, et al. Reference sequence (RefSeq) database at NCBI: current status, taxonomic expansion, and functional annotation. Nucleic acids research 2016;44(D1):D733–D745.
60. Sayers EW, Beck J, Bolton EE, Bourexis D, Brister JR, Canese K, et al. Database resources of the national center for biotechnology information. Nucleic acids research 2021;49(D1):D10.
61. Jesus TF, Ribeiro-Gonçalves B, Silva DN, Bortolaia V, Ramirez M, Carriço JA. Plasmid ATLAS: plasmid visual analytics and identification in high-throughput sequencing data. Nucleic acids research 2019;47(D1):D188–D194.
62. Burki F, Roger AJ, Brown MW, Simpson AG. The new tree of eukaryotes. Trends in ecology & evolution 2020;35(1):43–55.
63. Simmonds P, Adams MJ, Benkő M, Breitbart M, Brister JR, Carstens EB, et al. Virus taxonomy in the age of metagenomics. Nature Reviews Microbiology 2017;15(3):161–168.
64. Harris HM, Hill C. A place for viruses on the tree of life. Frontiers in Microbiology 2021;11:604048.

Manuscript

Click here to access/download
**Supplementary Material**
HYMET___supplementary_material.pdf

Manuscript Click here to access/download;Personal Cover;coverletter.pdf

**Jorge Miguel Ferreira da Silva**
IEETA/DETI, University of Aveiro
Aveiro, Portugal
jorge.miguel.ferreira.silva@ua.pt
+351 234 370 500

09/05/2025

Editor-in-Chief
*GigaScience*

Dear Editor,

I am pleased to submit our manuscript entitled **"HYMET: A Hybrid Metagenomic Pipeline for Accurate and Efficient Taxonomic Classification"** for consideration in *GigaScience*. Authored by Inês Martins, João Rafael Almeida, and myself, the work introduces HYMET, a lightweight pipeline that combines rapid $k$-mer screening with precise alignment to achieve robust taxonomic classification across all biological domains.

Metagenomic studies are often limited by the computational burden of large reference databases, the bias inherent in those resources, and the diminished accuracy of existing tools when confronted with highly mutated or fragmented sequences. HYMET overcomes these barriers through a two-stage workflow: first, *Mash Screen* rapidly filters candidate genomes, and then *Minimap2* refines the search with high-fidelity alignments. A dynamic thresholding algorithm tailors the reference set to each sample, and a weighted lowest-common-ancestor scheme delivers reliable lineage calls down to the species level. In benchmarks encompassing 26 203 genomes (14.76 GB) from Bacteria, Archaea, Eukarya, and viruses, HYMET achieved mean F1 scores above 0.9 and retained performance greater than 0.8 even at 30 % simulated mutation. Typical analyses complete in under two hours on standard workstations, with installation requiring only 2.82 GB and a transient reference cache of 10–50 GB—an efficiency advantage of one to two orders of magnitude over current state-of-the-art pipelines. All source code, sketched reference databases, test data, and analysis scripts are openly available under permissive licences, ensuring full reproducibility.

These contributions align directly with *GigaScience*'s commitment to disseminating reproducible, data-intensive research. The manuscript is original, not under review elsewhere, and all authors have approved its submission. No ethical approvals are required, as the study is entirely computational, and there are no competing interests to declare.

Thank you for considering our work. We believe this paper will be of significant interest to the journal's readership, providing both a methodological advance and a freely accessible resource for the metagenomics community. We look forward to your response and would be happy to supply any additional information you may require.

Sincerely,

Jorge Miguel Ferreira da Silva

Manuscript
Click here to access/download;Graphical Abstract;abstract.pdf

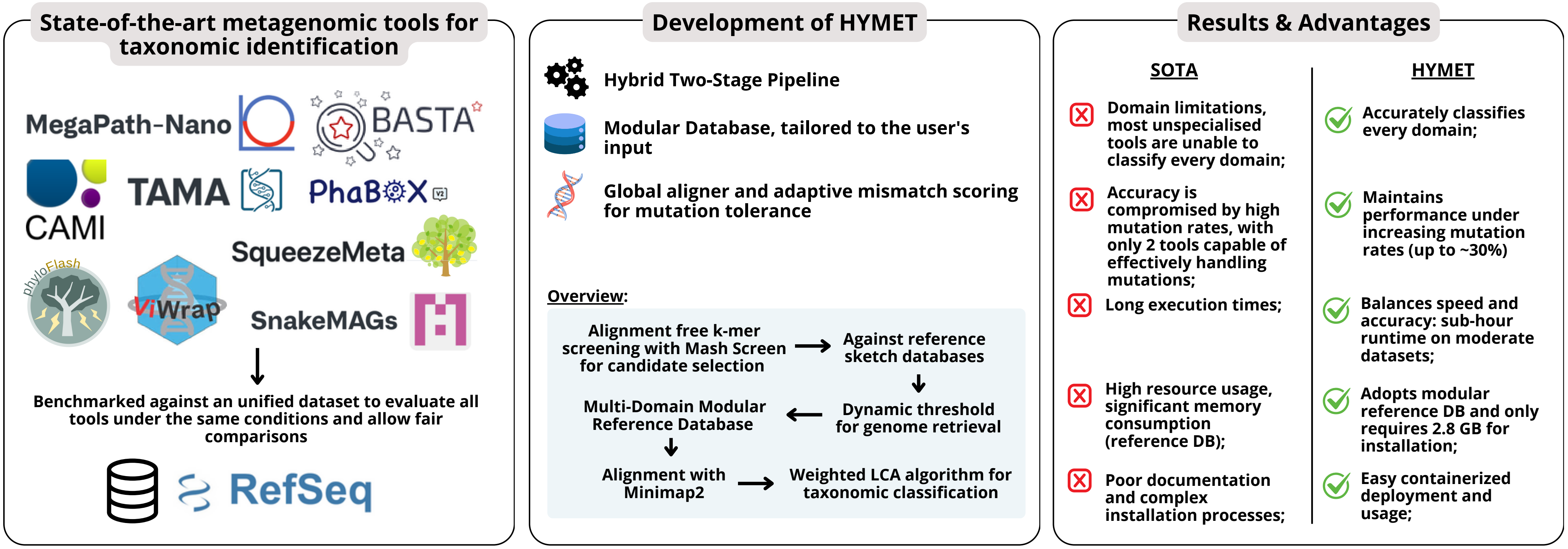

Supplement: giag024_GIGA-D-25-00184_original_submission [file giag024_giga-d-25-00184_original_submission.pdf]
