# Supplementary material for: HYMET: a hybrid metagenomic pipeline for accurate and efficient taxonomic classification
Source: Gigascience. 2026 Mar 2;15:giag024. doi: 10.1093/gigascience/giag024 (PMC13042306; doi:10.1093/gigascience/giag024)

GigaScience

HYMET: A Hybrid Metagenomic Pipeline for Accurate and Efficient Taxonomic Classification

--Manuscript Draft--

**Manuscript Number:**	GIGA-D-25-00184R1
**Full Title:**	HYMET: A Hybrid Metagenomic Pipeline for Accurate and Efficient Taxonomic Classification
**Article Type:**	Technical Note
**Funding Information:**	FCT Fundação para a Ciência e a Tecnologia (00127-IEETA) — Not applicable<br>European Commission (101081813) — Not applicable<br>FCCN Fundação para a Computação Científica Nacional (2023.14342.CPCA.A1) — Not applicable
**Abstract:**	Background: Reliable taxonomic classification of metagenomic sequences remains constrained by high mutation rates, fragmented assemblies, and large heterogeneous reference databases. HYMET (Hybrid Metagenomic Tool) was developed to overcome these challenges through a two-stage hybrid design combining adaptive Mash-based screening with Minimap2 alignment and a coverage-weighted Lowest Common Ancestor (LCA) classifier. Its sample-adaptive thresholds and on-the-fly reference construction enable efficient, domain-agnostic classification while maintaining accuracy across divergent genomes.<br><br>Results: Across seven CAMI assemblies in contig mode, HYMET achieved a mean F1 of 83.89%, with genus-level F1 of 76.75% and species-level F1 of 60.18%, while averaging 115.93 s runtime and 6.24 GB of peak memory. Performance remained stable under mutation rates up to 30% for most domains (F1≥0.8), with viral sequences showing the expected decline (F1≈0.5 at 30%). Read and contig inputs produced nearly identical results when sharing reference caches, and real-world datasets confirmed robustness with the human gut metagenome reproduced typical anaerobic profiles, while in the ZymoBIOMICS mock community HYMET recovered all bacterial members.<br><br>Conclusions: HYMET achieves a practical balance of accuracy, efficiency, and scalability for metagenomic classification. Its adaptive candidate selection, alignment-anchored taxonomy, and reproducible reference caching collectively enhance performance across domains. HYMET source code is fully available at https://github.com/ieeta-pt/HYMET.
**Corresponding Author:**	Jorge Miguel Ferreira da Silva<br>Universidade de Aveiro Instituto de Engenharia Eletrónica e Informática de Aveiro<br>Esmoriz, Seleccione um PORTUGAL
**Corresponding Author Secondary Information:**	
**Corresponding Author's Institution:**	Universidade de Aveiro Instituto de Engenharia Eletrónica e Informática de Aveiro
**Corresponding Author's Secondary Institution:**	
**First Author:**	Jorge Miguel Ferreira da Silva
**First Author Secondary Information:**	

**Order of Authors:**	Jorge Miguel Ferreira da Silva
	Inês Martins, M.D.
	João Rafael Almeida
**Order of Authors Secondary Information:**	
**Response to Reviewers:**	HYMET (GIGA-D-25-00184R1) – Response to Reviewers<br>We appreciate the comments, which have contributed to improving the clarity, completeness, and robustness of our work. In the revised version, we have addressed the points raised, providing detailed responses, implementing manuscript edits, and, where applicable, enhancing the HYMET codebase to ensure consistency between the description and the tool's actual capabilities. We believe these revisions have substantially strengthened the manuscript, ensuring it meets the standards of GigaScience. We hope it is now suitable for publication.<br><br>Reviewer #1<br>The authors present a metagenomic classification workflow -- HYMET, which integrates k-mer screening (Mash) and alignment-based method (Minimap2) to reduce computational resources. Although it's not the first tool to employ this strategy in this field, HYMET offers some innovations, including a dynamic threshold method for candidate genome selection and a hybrid taxonomic assignment strategy.<br><br>Response:<br><br>We thank the reviewer for their constructive comments and careful evaluation of our work. We have carefully addressed the points raised in the review and revised the manuscript accordingly. We believe that these changes have strengthened the clarity and impact of the paper, and we hope that it is now suitable for publication.<br><br>Major:<br><br>Comment:<br>Metalign (10.1186/s13059-020-02159-0) adopts a highly similar strategy of pre-filtering with CMash and alignment with Minimap2. The manuscript does not mention this.<br><br>Response:<br><br>We agree. Metalign uses CMash pre-filtering followed by Minimap2. We now cite and discuss it. We also clarify what is new in HYMET, being the use of Mash Screen with a dynamic, sample-adaptive threshold, on-the-fly database construction across all domains, and weighted-LCA taxonomic assignment.<br>Comment:<br>- The chosen competitors (most are workflows) do not cover the widely used and state-of-the-art tools, such as KrakenUniq, Ganon, Sourmash gather, Taxor, Centrifuger, etc.<br><br>Response:<br>Agreed. We now cite KrakenUniq, Ganon/ganon2, Centrifuge and the recent Centrifuger, Taxor, and sourmash gather, and we state why they were not benchmarked directly. Our comparisons target end-to-end, cross-domain workflows; component-level short-read classifiers are represented via pipelines already in our set (e.g., CAMITAX uses Centrifuge/Kaiju; TAMA combines Kraken/CLARK/Centrifuge). Furthermore, we now compare many of these tools in a direct comparisson in CAMI section with HYMET, and added a brief scope note and references.<br>Comment:<br>- Is there a specific reason to use Mash for pre-filtering? Does it perform better than Sourmash, CMash, etc?<br><br>Response:<br><br>We agree that clarifying the choice of Mash is important. We chose Mash Screen primarily because it directly estimates containment between unassembled read sets and reference genomes, uses compact fixed-size sketches, and provides built-in statistical significance testing. These features integrate into HYMET's adaptive containment threshold approach, simplifying our strategy of selecting candidates per

Powered by Editorial Manager® and ProduXion Manager® from Aries Systems Corporation

query. Other similar tools such as sourmash typically employ scaled MinHash sketches and iterative set-cover methods, making them better suited for compositional decomposition tasks rather than directly prioritizing references by containment scores for downstream alignment. CMash (as used by Metalign) provides multi-resolution containment estimates across multiple k-values simultaneously. However, this capability was not required in HYMET, as we already handle separate sketch sets for different k-values and adjust containment thresholds dynamically per sample.

Comment:
- There's only one test dataset (test and validation dataset seems to be the same one), no real sequencing data. Moreover, the test dataset is simulated by introducing only point mutations, no structure variations. Widely used test datasets can be more convincing, such as those from the CAMI challenge.

Response:
We have expanded the evaluation to include the CAMI challenge subsets (seven samples across six tools) with full metrics. We also report real world case studies (MGnify gut contigs and the Zymo mock community) with runtime and taxonomic.
Comment:
- The program is not easy to use.
--- Though it's installable with conda, users still need to clone the code and run it within the directory.

Response:
We agree and have refactored HYMET to facilitate this issue. We unified Python CLI (bin/hymet); and it now allows directory-independent execution (e.g. hymet run/bench/case) and wraps batch processing, so cloning into a specific path is no longer required.
Comment:
--- The main entry is a Perl script named 'main.pl', and users need to manually type in the input directory, which prevents batch processing.

Response:

The maintained entry point is now the Python CLI (bin/hymet), which accepts explicit --contigs, --out, and --threads parameters and delegates to the same workflow wrappers used in the manuscript's experiments. main.pl is kept only for legacy compatibility and is flagged as such in the documentation.

Comment:
--- Though the docs say it accepts both read and contig as input, the tutorial says the input directory needs to contain .fna files.

Response:
Thank you for highlighting this ambiguity. The maintained entry point is the Python CLI, which supports both contig and read inputs. Contig runs accept a single multi FASTA (e.g., .fna/.fa/.fasta), while reads runs are supported via the harness and single sample CLI using Minimap2's sr preset. We updated the Supplementary to include an explicit reads example and to clarify the preset and other reproducibility knobs.

Minor:
Comment:
- Table 1: Why use different seed values for different databases? Did it affect the accuracy?

Response:
We thank the reviewer for pointing this out. The use of different seed values is due to how the reference sketches were obtained: the RefSeq88 sketch was downloaded directly from the Mash project, which provides its sketches built with seed 0, while we generated the GTDB and custom databases locally using Mash's default seed 42. Importantly, Mash Screen always hashes queries according to the seed embedded in each sketch, and each sketch file we use is internally consistent. Given the large sketch sizes (1000–5000 hashes), minor differences in seed selection have negligible impact on accuracy, and any minor stochastic variation is resolved during downstream

Powered by Editorial Manager® and ProduXion Manager® from Aries Systems Corporation

alignment. Thus, the choice of different seeds across databases does not meaningfully affect HYMET's performance.
To clarify this, we have added a brief note to the manuscript indicating the reason behind the seed differences.

Comment:
- Table 3: CPU usage is less informative, and exact memory occupation should be used, rather than percentage.

Response: The CAMI benchmark and both case studies (gut and Zymo) now report wall clock time and peak RSS (GB), providing clearer and more comparable measures of resource usage.
Comment:
- Downloading reference genomes might be slow and even problematic in some regions around the world, due to the connection speed and connectivity.

Response:
We agree. HYMET now minimizes network transfers and supports constrained connectivity. First, it screens locally against compact Mash sketches, so only a small set of candidate genomes is fetched. Second, every downloaded reference bundle is cached under a stable key and reused across runs, including the concatenated FASTA and the Minimap2 index. Third, the workflow supports mirrors and preloading: users can point candidate deduplication at a local mirror of the NCBI assembly summaries, and pre-seed a cache entry with a curated FASTA and a sequence-to-TaxID map using the provided preload helper, after which indices rebuild locally. The downloader itself uses parallel fetches with retries and exponential backoff and logs any failures. Together, these measures reduce transfer when a network is available and enable reproducible, fully offline runs once caches are populated.

Comment:
- The output should output TaxIDs for further analysis, which is more stable than lineages.

Response:
We agree, HYMET now emits TaxID. We highlighted this in the documentation and ensured the downstream converters keep the TaxID column alongside the lineage strings.

Reviewer #2

Martins et al. present HYMET, a novel method for fast and accurate taxonomic classification across all domains of life. Taxonomic classification remains a central challenge in metagenomics, and HYMET has the potential to contribute meaningfully. However, the current benchmarking is not sufficiently robust to support claims of superiority, and several critical issues should be addressed prior to publication.

Response:
We thank the reviewer for the constructive feedback. We have addressed the issues raised and revised the manuscript to improve clarity and robustness. We believe the paper has been strengthened and is now suitable for publication.
Comment:
* The rationale for sampling 10% of genome sequences to simulate fragmentation should be explained. Performance should be evaluated at varying genome fractions (e.g., 10%, 20%, 30%), reflecting more realistic diversity in metagenomic datasets. The authors should also consider benchmarking on simulated data derived from public metagenomic contigs to better emulate real-world conditions.

Response: We thank the reviewer for this suggestion. Our goal with the 10% choice was to emulate contig-level fragmentation and heterogeneity while keeping the build and evaluation pipeline reproducible and tractable. To that end, we subsample at two stages: (i) we select 10% of assemblies, then (ii) we select 10% of each assembly's sequences. We have documented the exact steps and scripts used (including the filtering stage) so the sampling can be reproduced precisely. Evaluations at additional fractions (e.g., 20% and 30%) are planned. In this revision we focus on the

Powered by Editorial Manager® and ProduXion Manager® from Aries Systems Corporation

consolidated CAMI suite and two real-data case studies (MGnify gut contigs and the Zymo mock community), which together provide a stronger view of real-world performance than simulated data alone. We note that the case harness already accepts public metagenomic contigs directly via a manifest, so future experiments that derive simulated sets from public contigs can be integrated without changes to the pipeline.
Comment:
* The manuscript does not clarify how mutation rates (0% to 30%) were introduced. If mutations were randomly generated, this should be stated explicitly. Simulations should ideally reflect evolutionary processes to better mirror biological variation.

Response:
We appreciate the point and now state this explicitly. For the mutation sweep, we used a deterministic simulator that applies independent per-base substitutions at the target rate (0–30%), with optional short (1–3 bp) insertions/deletions, all under a fixed random seed for reproducibility. This produces controlled divergence for robustness testing and is not intended to emulate evolutionary processes or tree-based substitution models. Our primary evidence relies on CAMI ground-truth datasets and real case studies, the mutation sweep complements these by stress-testing tolerance to divergence.
Comment:
* Commonly used methods like MetaPhlAn and Kraken should be included in comparative evaluations to contextualize HYMET's performance within the current landscape of tools.

Response:
We now addressed via the expanded CAMI benchmarking suite, which now includes MetaPhlAn4, kraken2, ganon2, centrifuge, sourmash gather, and HYMET with publicly available metrics.
Comment:
* For a fair comparison with other tools, a standardized database should be used wherever possible. For methods that support user-defined databases, such as Kraken, the authors should construct and apply the same reference database. A supplementary table summarizing the database composition for each benchmarked tool (e.g., number of genomes, species, domains) should be included to ensure transparency and facilitate reproducibility.

Response:
We now include a concise supplementary table summarizing each tool's database provenance, build date, and size, for tools that require their own official databases, we note deviations. HYMET's sketch checksums and Zenodo record (doi: 10.5281/zenodo.17428354) are also documented.

Comment:
* The manuscript references a single representative dataset (Table 2) for benchmarking. To demonstrate robustness, the authors should evaluate HYMET across multiple representative datasets with varied genome content.

Response: We now adress this issue by the expanded CAMI runs and the real-data case studies described in the revised Results section.

Comment:
* Benchmarking is currently based solely on the authors' own simulated data. This is insufficient to validate performance claims. Standard datasets such as those from the CAMI challenge should be incorporated.

Response: Thank you for the comment, we have adress this through the CAMI challenge evaluations, whose outputs and figures are now part of the results package.
Comment:
* A case study applying HYMET to a real-world metagenomic dataset would significantly strengthen the manuscript and demonstrate practical utility.

Response: Thanks you for the comment. We have now Added the MGnify gut and Zymo mock community case studies with visual summaries and runtime stats.
Comment:

* The impact of database incompleteness on HYMET's performance should be evaluated, as real-world databases rarely contain all possible genomes.

Response: Thank you for your comment. To adress this issue we have conducted curated-reference ablation experiments that quantify classification retention when key taxa are removed.

Comment:
* The manuscript's organization needs improvement. The Results section is too brief and lacks detail about the benchmarking experiments. The number of samples and replicates per parameter combination should be clearly described within the Results, not just in the Methods or Supplementary Materials. The "HYMET Workflow" section should be moved under Materials and Methods.

Response:
Thank you for the comment. We've reorganized and clarified as requested. The HYMET Workflow is now under Materials and Methods. The Results now include a concise Benchmark Scope and Replicates subsection that states: CAMI (7 assemblies), one run per sample per tool (no technical replicates), HYMET contigs vs reads on the same 7 assemblies, one run per mode, mutation sweep (0–30%) one run per rate per group, two case studies (MGnify gut, Zymo), one run each. We also reference the versioned manifest and harness in the Results for traceability.

Minor Comments

Comment:
* The abstract's opening sentence ("Metagenomics has revolutionized...") is generic and does not frame the specific problem of taxonomic classification. It should be revised or removed.

Response:
We revised the abstract's opening to frame the specific problem of metagenomic taxonomic classification and HYMET's contribution, removing generic phrasing.

Comment:
* The Introduction should include a brief rationale for why HYMET could outperform existing methods and summarize its core innovations. A note on the inspiration behind HYMET's design would also add clarity.

Response:
We tightened the Introduction to motivate HYMET's design and summarize key innovations (adaptive Mash Screen, on the fly reference construction, weighted LCA resolver).
Comment:
* The manuscript is overly wordy. For example, the opening sentence of the Introduction does not add meaningful context and can be removed. The text should be streamlined to improve readability.

Response:
We streamlined the Introduction by removing generic lead ins and tightening wording.
Comment:
* The potential for HYMET to classify 16S rRNA sequencing data should be discussed.

Response:
HYMET targets assembled contigs and whole genomes; while 16S segments within contigs can be classified, targeted 16S amplicon profiling is better served by 16S specific tools (e.g., phyloFlash). We added a brief scope note to the manuscript explaining this.
Comment:
* As Figure 1 indicates that HYMET can accept both contigs and reads, the tool's performance should be evaluated separately for each input type.

Response:
We agree that input types should be assessed separately. The canonical benchmark in

Powered by Editorial Manager® and ProduXion Manager® from Aries Systems Corporation

this revision is contig-based (CAMI panels and case studies), and we state this explicitly in the Results. In addition, we performed an internal HYMET-only comparison of the two input paths, contigs vs reads, using the harness read-mode (FASTQ staging, Mash screening, Minimap2 short-read alignment, weighted-LCA). Using synthetic reads derived from the CAMI assemblies to preserve ground truth, we observed near-identical contig-level accuracies but differences in profile-level F1 at species, consistent with abundance estimation from read counts. This supports keeping cross-tool comparisons on contigs while providing a HYMET-vs-HYMET(reads) side-by-side view for transparency.

Reviewer #3
I would like to thank the authors for developing a well-structured and thoughtfully implemented tool, and for ensuring the work is fully reproducible through the inclusion of accessible datasets and code. While the manuscript is already strong and suitable for publication, I would like to offer a few suggestions that, in my opinion, could further enhance the clarity, robustness, and impact of the work.

Response:
We thank the reviewer for the positive assessment of our work and for recognizing the reproducibility and structure of the manuscript. We have carefully considered the suggestions provided and incorporated revisions to improve clarity. We believe the manuscript has benefited from these adjustments and hope is now suitable for publication.
Comment:
1. The validation dataset was chosen from curated sequences. The F1 scores for these are quite high for most of the tested groups. Could you provide an environmental or human microbiome sample to showcase real-world applicability with a broader application?

Response:
Addressed via the MGnify gut metagenome case study (plus the Zymo mock community), whose outputs and figures accompany the revised manuscript.
Comment:
2. Could you provide or propose strategies to improve classification at genus/species level, especially for high-mutation or underrepresented taxa?

Response:
At lower ranks, we recommend a practical set of steps to recover genus/species calls in difficult settings. First, soften the pre-filter so that more divergent candidates proceed to alignment; this helps when sequences carry many mutations. Second, augment the reference set with a small curated panel for the taxa of interest so that close representatives are available during alignment. Third, adjust the reporting thresholds of the weighted-LCA so that low-abundance but internally consistent evidence is retained, while still defaulting to genus-level reporting whenever species-level support is insufficient. Finally, use the ablation analysis to identify concrete gaps and focus curation where it measurably improves recall. Together, these measures improve sensitivity for high-mutation and underrepresented clades while maintaining precision through alignment confirmation and conservative calling.

Comment:
3. Genome retrieval failure is mentioned for some of the genomes. Could you propose an alternative strategy or a backup plan for genome downloads when RefSeq access fails, either automated retry policies or alternative sources.

Response:
Yes, we now adopted a more resilient retrieval policy. HYMET consults both RefSeq and GenBank catalogues and retrieves assemblies over HTTPS; transient errors are handled with bounded retries and exponential backoff, with failures recorded in the run logs. For constrained or offline environments, users can point to mirrored assembly summaries and pre-seed the reference cache with small curated panels so the pipeline proceeds without network access; indices are rebuilt on the next run. Persistent failures no longer block classification when sufficient references remain available. Information was added to the Supplementary Material.

Powered by Editorial Manager® and ProduXion Manager® from Aries Systems Corporation

	Comment:<br>4. Is it possible to add a Singularity-Aptainer installation procedure? That would extend the use to HPC where the admin privileges are not necessary as in Docker.<br><br>Response:<br>Yes. We added a Singularity/Apptainer workflow for HPC use, the repository now ships a definition file (Singularity.def) mirroring the Docker image, and we document a simple build/exec procedure so HYMET can run without root privileges on clusters. The container exposes the same hymet CLI as the Bioconda and Docker installs, so inputs/outputs and caching behave identically.
**Additional Information:**	
**Question**	**Response**
Are you submitting this manuscript to a special series or article collection?	No
**Experimental design and statistics**<br><br>Full details of the experimental design and statistical methods used should be given in the Methods section, as detailed in our Minimum Standards Reporting Checklist. Information essential to interpreting the data presented should be made available in the figure legends.<br><br>Have you included all the information requested in your manuscript?	Yes
**Resources**<br><br>A description of all resources used, including antibodies, cell lines, animals and software tools, with enough information to allow them to be uniquely identified, should be included in the Methods section. Authors are strongly encouraged to cite Research Resource Identifiers (RRIDs) for antibodies, model organisms and tools, where possible.<br><br>Have you included the information requested as detailed in our Minimum Standards Reporting Checklist?	Yes
**Availability of data and materials**<br><br>All datasets and code on which the	Yes

conclusions of the paper rely must be either included in your submission or deposited in publicly available repositories (where available and ethically appropriate), referencing such data using a unique identifier in the references and in the "Availability of Data and Materials" section of your manuscript.<br><br>Have you have met the above requirement as detailed in our Minimum Standards Reporting Checklist?	
GigaScience has policies and guidelines in place for the use of generative AI-writing tools such as ChatGPT. If you have used such writing tools to assist with writing the manuscript this must be declared and cited in the text. Authors should not list AI-writing tools and other AI-assisted technologies as an author or co-author and should acknowledge that they are fully responsible for text generated or refined by AI-writing tools.<p><br><br>A summary of use (particularly in the introduction or among methods) needs to be included at the end of the paper, and the outputs should also be included as a supplementary file hosted in GigaDB or other open repositories. Please read our guidelines for more information. <p><br><br>By submitting to GigaScience, you are aware of the journal's AI-writing tools policy, and if you have declared use of such tools below, you have acknowledged this where appropriate in your manuscript and have made a summary of use and outputs available. <p><br><b>AI-assisted writing tools have been used in the preparation of this manuscript?	No

LaTeX - Main Document (TeX file)

Click here to access/download;LaTeX - Main Document (TeX file);main.tex

GigaScience, 2023, 1–16

doi: xx.xxxx/xxxx
Manuscript in Preparation
Paper

PAPER

HYMET: A Hybrid Metagenomic Pipeline for Accurate and Efficient Taxonomic Classification

Inês Martins[1,*], Jorge Miguel Silva[1,*] and João Rafael Almeida[1]

[1]IEETA/DETI, LASI, University of Aveiro, Aveiro, Portugal

[*]inesbrancomartins@ua.pt; jorge.miguel.ferreira.silva@ua.pt

Abstract

Background: Reliable taxonomic classification of metagenomic sequences remains constrained by high mutation rates, fragmented assemblies, and large heterogeneous reference databases. HYMET (Hybrid Metagenomic Tool) was developed to overcome these challenges through a two-stage hybrid design combining adaptive Mash-based screening with Minimap2 alignment and a coverage-weighted Lowest Common Ancestor (LCA) classifier. Its sample-adaptive thresholds and on-the-fly reference construction enable efficient, domain-agnostic classification while maintaining accuracy across divergent genomes.
Results: Across seven CAMI assemblies in contig mode, HYMET achieved a mean F1 of 83.89%, with genus-level F1 of 76.75% and species-level F1 of 60.18%, while averaging 115.93 s runtime and 6.24 GB of peak memory. Performance remained stable under mutation rates up to 30% for most domains (F1 $\geq$ 0.8), with viral sequences showing the expected decline (F1 $\approx$ 0.5 at 30%). Read and contig inputs produced nearly identical results when sharing reference caches, and real-world datasets confirmed robustness with the human gut metagenome reproduced typical anaerobic profiles, while in the ZymoBIOMICS mock community HYMET recovered all bacterial members.
Conclusions: HYMET achieves a practical balance of accuracy, efficiency, and scalability for metagenomic classification. Its adaptive candidate selection, alignment-anchored taxonomy, and reproducible reference caching collectively enhance performance across domains. HYMET source code is fully available at `https://github.com/ieeta-pt/HYMET`.

Key words: Metagenomics, taxonomic classification, *k*-mer screening, alignment-based methods, computational efficiency, mutation resistance, hybrid pipeline.

Compiled on: October 31, 2025.
Draft manuscript prepared by the author.

Key Points

HYMET integrates Mash-based adaptive screening and Minimap2 alignment with a weighted Lowest Common Ancestor (LCA) classification, dynamically tailoring reference databases to each metagenomic dataset for improved efficiency and accuracy.

Evaluated across diverse CAMI benchmarks, HYMET achieves robust F1-scores (mean across ranks: 83.89%, genus: 76.75%, species: 60.18%) while maintaining rapid runtimes (average 116 s) and moderate memory usage (peak 6.2 GB).

HYMET demonstrates strong resilience to genetic mutations (up to 30%), maintaining high accuracy for most biological domains (F1 ≥ 0.8), though viral classification accuracy decreases at higher mutation rates (F1 ≈ 0.5 at 30%).

Real-world evaluations confirm HYMET's practical applicability: gut microbiome profiles closely matched expected anaerobic bacterial taxa, while ZymoBIOMICS mock-community results accurately identified bacterial constituents, despite missing yeast species under current parameters.

Resource-efficient design enables reproducible and scalable runs, the tool requires only 2.82 GB for installation, dynamically manages reference cache footprints (10–50 GB typical), and offers full reproducibility via Bioconda, container images, and versioned databases deposited on Zenodo.

1 Introduction

A significant challenge in metagenomics is the development of accurate methods for the taxonomic classification of organisms within a sample [1, 2]. Despite the creation of numerous general-purpose and specialized metagenomic tools, several significant issues persist. Particularly, computational demands pose a major constraint, as tools often require substantial memory and processing power, leading to impractical execution times for large datasets [3, 4, 1]. The sheer volume of metagenomic datasets demands highly efficient algorithms that can operate within reasonable requirements of compute power, which is particularly problematic when dealing with millions of sequencing reads [5, 6, 7, 8]. Furthermore, taxonomic assignment remains a critical challenge in metagenomic analysis, especially at lower taxonomic levels [8]. This issue is exacerbated by the limitations of reference databases, which often exhibit significant sampling bias towards well-studied organisms, while underrepresenting species that are difficult to culture in laboratory settings [3, 1]. This discrepancy results in high rates of unclassified or misclassified reads, especially in complex environmental samples [9, 2, 10]. Furthermore, the lack of standardized benchmarking protocols and datasets, hinder objective comparisons of tool performance, as researchers frequently test tools on non-uniform datasets with inconsistent evaluation metrics [10, 11, 12]. Addressing these issues is crucial for improving our understanding of complex microbial communities and developing efficient, user-friendly software solutions to analyze the enormous amounts of data generated by metagenomic research [3, 1]. These collective challenges directly motivate our core research question:

> *How can a next-generation metagenomic classification tool be designed and implemented to accurately identify taxa across all domains while maintaining high performance and efficiency?*

HYMET (Hybrid Metagenomic Tool) was conceived in response to the recurrent bottlenecks observed when processing large and diverse metagenomic datasets, where existing profilers either failed to capture divergent taxa or consumed excessive resources. Its design draws inspiration from practical experience with real microbial communities, where rapid screening and selective reference construction often proved more effective than relying on static, monolithic databases. HYMET follows this principle by integrating adaptive reference selection with precise alignment and a weighted taxonomic resolver. This hybrid, sample-aware approach enables the tool to dynamically tailor its search space to each dataset, thereby reducing memory usage and execution time while preserving classification accuracy across domains.

Specifically, HYMET introduces three main innovations. First, an adaptive Mash Screen step selects candidate references on-the-fly, ensuring that downstream analysis focuses only on the most relevant genomes under a fixed resource budget. Second, the selected references are combined into a temporary, sample-specific database that supports accurate alignment even in the presence of mutations or incomplete references. Third, a coverage-weighted lowest common ancestor (LCA) algorithm integrates the breadth and depth of alignment evidence to improve taxonomic consistency, particularly at lower ranks. Together, these components allow HYMET to outperform static index-based approaches in both speed and precision, especially for complex or previously unseen samples.

2 Background

In recent years, we have witnessed remarkable progress in metagenomics, particularly in the development of computational tools for taxonomic classification and functional analysis [2, 1, 3]. A dominant trend in current methodologies is the integration of established classification techniques into end-to-end pipelines, which streamline the entire analytical workflow, from raw sequencing data to biologically interpretable results [13]. Currently, the state-of-the-art landscape is populated by a rich ecosystem of interconnected tools, each offering unique capabilities and complementary approaches that collectively advance the field's analytical power. Among these, SnakeMAGs [14] stands out for its specialized focus on reconstructing prokaryotic genomes from Illumina sequencing reads, while SqueezeMeta [15] offers a fully automated and comprehensive solution for metagenomic data analysis [16, 13]. The first tool uses the Genome Taxonomy Database (GTDB) toolkit [17] for taxonomic assignment, leveraging conserved marker genes for analysis. On the other hand, SqueezeMeta uses DIAMOND [18] for alignment and the Lowest Common Ancestor (LCA) algorithm for taxonomic assignment [15].

Complementing these general-purpose pipelines, several lightweight tools have emerged to address specific needs in taxonomic assignment. The Basic Sequence Taxonomy Annotator (BASTA) [19] also employs the LCA algorithm for efficient sequence classification, while the Critical Assessment of Metagenome Interpretation Taxonomy (CAMITAX) [4] improves accuracy through the integration of multiple classification strategies for microbial genome assignment, including genome distance-based classification using Mash [20], Centrifuge [1] and Kaiju [21], that determines the interval-union LCA of gene-level assignments and 16S rRNA gene-based classification employing a naive Bayesian classifier method using Dada2 [22]. For more robust taxonomic profiling, the Taxonomy Analysis by Multiple Assignment (TAMA) tool [23] combines consensus classifications from established classifiers, including Kraken [8], CLARK [24], and Centrifuge, leveraging their complementary strengths. In addition to these workflows, widely used read-level classifiers include KrakenUniq, Ganon (and ganon2), Centrifuge/Centrifuger, Taxor, and compositional MinHash methods such as sourmash gather; these are often embedded within workflows (for example, CAMITAX integrates Centrifuge/Kaiju and TAMA combines Kraken, CLARK, and Centrifuge), but we do not benchmark them directly here [25, 26, 27, 28, 29, 30].

The field has also seen the development of specialized tools that target specific metagenomic applications. Viral genomics is particularly well served by PhaBOX [31, 32] for viral contig characterization and ViWrap [33] for prediction of viral-host relationship, both providing valuable information on viral diversity and ecological interactions [34, 35, 36]. The first tool, PhaBOX, developed by Shang et al., combines gene prediction and alignment (DIAMOND) with taxonomic classification by semi-supervised learning method (PhaGCN [32]), based on sequence similarities and cluster sharing networks, and final assignments using the LCA. ViWrap, on the other hand, uses machine learning and sequence similarity searches to identify viral sequences and BLAST [37] to identify best hits against databases for taxonomic annotation and host prediction. For the analysis of the microbial community, PhyloFlash [38], developed by Gruber-Vodicka et al., offers unique capabilities through its small subunit ribosomal RNA (SSU rRNA)-based approach, enabling both metagenomic profiling and high-resolution phylogenetic studies [39]. In the critical area of antimicrobial resistance surveillance, MegaPath-Nano [40] has emerged as an important tool for the comprehensive detection of resistance genes, directly supporting public health monitoring efforts; it couples hash-based *k*-mer mapping with Minimap2's seed-chain-extend *local* alignment model [41], thereby covering broad and potentially divergent sequence segments [42, 43]. Related alignment-based profilers adopt a two-stage design. For example, Metalign [44] first applies CMash to pre-filter the reference by containment and then aligns reads with Minimap2 to produce the profile.

Despite this technological progress, significant challenges impede the broader implementation of metagenomic tools in clinical and research settings. Implementation barriers represent a primary obstacle, with inadequate documentation and complex installation procedures frequently compromising tool accessibility and user

adoption [45]. Computational constraints further limit practical application, as excessive memory and storage requirements hinder scalability. This is exemplified by SqueezeMeta, which stages more than 500 GB of on-disk reference data and exhibits prohibitively long processing times [15], and BASTA, whose dependence on BLAST-based alignments creates computational bottlenecks that render it inefficient for time-sensitive analyses [19, 37]. A fundamental limitation stems from reference database dependencies rather than inherent tool restrictions. For instance, TAMA demonstrates robust classification capabilities in principle, but its default bacterial reference database necessarily limits its taxonomic scope to bacterial identification while requiring hundreds of gigabytes of disk space for its bundled indices [23]. Similarly, independent evaluations of MegaPath-Nano confirm its strong performance in the detection of prokaryotic antimicrobial resistance, but note a reduced sensitivity when analyzing higher eukaryotes [40, 42]. This pattern of taxonomic bias is further evidenced in specialized tools such as PhaBOX and ViWrap, which, while excelling in virome analysis, lack versatility for broader metagenomic applications [33, 31]. PhyloFlash's reliance on small subunit rRNA analysis makes it fundamentally unsuitable for viral identification, as viruses lack ribosomal RNA genes [38].

3 Materials and Methods

3.1 HYMET workflow overview

HYMET, illustrated in Figure 1, is driven by a unified Python CLI (`hymet` from Bioconda or `bin/hymet` in a source checkout) that orchestrates Mash (k-mer screening), Minimap2 (alignment), and the weighted-LCA classifier. For reproducible installs we provide container images (Docker and Apptainer/Singularity) and a Bioconda package (Conda/Mamba). Legacy Perl components are bundled for reproducibility but are not the primary entry point. HYMET is designed for shotgun metagenomes in contig or genome form. It can classify 16S/SSU rRNA sequences when they occur in assembled contigs or shotgun reads, but targeted 16S rRNA amplicon libraries are out of scope and are better analyzed with rRNA-centric profilers (e.g., phyloFlash [38]). Installation and configuration details are provided in Supplementary Sections 1–4.

Alignment-Free k-mer Screening

The initial phase of HYMET utilizes Mash Screen [20] for rapid k-mer-based screening against pre-computed MinHash reference sketch databases [46, 47, 48, 49, 50]. Mash employs containment scores, as defined in Equation 1, to assess the proportion of a reference genome present in a query sequence [51, 52, 53]. The containment index $c_k(a, b)$ is estimated as:

$$c_k(a, b) \approx \frac{|S(A) \cap \pi(B)|}{|S(A)|}, \quad (1)$$

where $S(A)$ is the sketch of the reference genome A, and $\pi(B)$ represents the k-mers of the query sequence B. The containment index ranges from 0.0 to 1.0, with values closer to 1.0 indicating a higher proportion of k-mers from the reference genome present in the query. This metric is crucial for tasks such as contamination screening, reference genome selection, and the discovery of novel genomes, as it provides a rapid and unbiased estimate of sequence representation [46, 47, 51, 49, 54]. In this work, the screening process was optimized for computational efficiency by enabling parallel processing and applying a stringent 90% similarity threshold to retain only high-confidence matches, filtering out low-quality alignments. This containment-based approach prioritizes likely taxonomic candidates, reducing the search space and computational load for subsequent alignment-based stages [20, 46, 52]. As

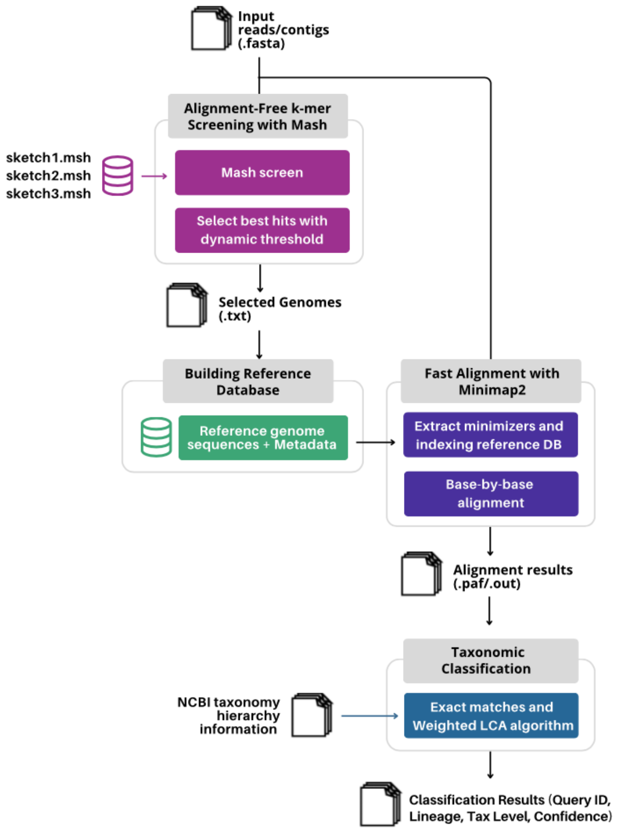

Figure 1. Overview of HYMET architecture.

previously mentioned, to enable this screening, Mash relies on sketched databases, which are compact representations of genomic sequences. These databases are built using consistent k-mer hashing with MurmurHash3 [20, 55, 47, 56], which allows efficient comparison of query sequences against large collections of reference genomes.

HYMET requires fast per-reference containment ranking on unassembled reads under a fixed memory budget. Mash Screen provides fixed-size MinHash sketches, direct read-set → reference containment estimates, and per-hit p-values, which integrate cleanly with our adaptive cut-off and candidate budgeting. In contrast, sourmash uses scaled FracMinHash with an iterative gather (set-cover) procedure aimed at mixture decomposition rather than per-reference ranking, and CMash (as in Metalign [44]) focuses on multi-k containment estimation, which we do not require here. Consequently, HYMET applies a dynamic, sample-adaptive containment threshold that by default targets about 3.25 candidates per input sequence (rounded; minimum of five) and enforces a floor threshold of 0.70, reducing the search space while preserving recall. The pre-filter is modular and could be replaced by sourmash or CMash without altering downstream alignment; we chose Mash for its simplicity, stable memory, and native significance testing [30, 57, 44].

Sketch and k-mer Size. The construction of these sketch databases involves two key parameters: the k-mer size (k) and the sketch size (s). The choice of k-mer size is essential as it balances sensitivity and specificity. Smaller k-mers increase sensitivity for divergent genomes but may lead to random collisions, while larger k-mers reduce collisions but may miss subtle variations [58, 20, 47, 51, 50]. The optimal k-mer size is calculated as:

$$k = \log_{|\Sigma|}\left(\frac{n(1-q)}{q}\right), \quad (2)$$

where $|\Sigma|$ is the alphabet size (4 for nucleotides), n is the genome size, and q is the desired probability of observing a random k-mer. For example, smaller genomes (e.g. viruses) and highly variable taxa require smaller k-mer sizes (e.g. k-mer=15) to ensure specificity, while larger genomes (e.g. vertebrates) benefit from moderate k-mer sizes (e.g. k-mer=21) to balance sensitivity and computational efficiency [53, 58].The sketch size, which refers to the number of unique min-hashes retained for genomic sequence representation, also plays a critical role in determining the accuracy of distance and containment estimates [58, 20, 50, 52]. The error associated with containment estimation for a given sketch size, s, is proportional to:

$$\text{Error} \approx \sqrt{\frac{1}{s}}, \tag{3}$$

indicating that larger sketch sizes improve precision, but at the expense of greater computational resources [58]. For instance, smaller or highly fragmented genomes typically require larger sketch sizes to ensure sufficient genomic information is captured, while larger or less fragmented genomes can achieve accurate containment estimates with smaller sketch sizes. This adaptive approach is supported by empirical evidence, with studies demonstrating that a sketch size of $s = 1000$ is generally adequate for obtaining precise similarity estimates in well-assembled genomes [20]. In fact, B. D. Ondov *et al.* established $s = 1000$ and k=21 as the default parameters in Mash, as they provide precise similarity estimates for well-assembled genomes [20]. However, for more divergent genomes, increasing the sketch size (e.g. $s = 5000$) can improve accuracy by capturing a more representative subset of genomic content [58, 50, 59, 53].

Reference Sketched Databases. Following these design principles, we implemented a comprehensive database strategy combining both established public resources and a custom-built collection:

- **RefSeq Nucleotide Release 228:** Contains sketches of 162 138 organisms from RefSeq release 228, compressed using $k = 21$ and $s = 1000$, reducing the original 1.2 TB dataset to a compact representation [58].
- **GTDB r202 Assembly Set and NCBI Complete Genomes Database:** Combines 89 675 genomes from GTDB r202 and NCBI RefSeq (viruses, fungi, and bacteria/archaea), compressed with $k = 21$ and $s = 1000$ [56].
- **Custom Reference Database:** Enhances representation of underrepresented taxa by including 19 505 up-to-date genomes from NCBI RefSeq. For smaller genomes (e.g., archaea, fungi, protozoa, viruses), sketches were generated with $k = 15$ and $s = 5000$, while larger genomes (e.g., vertebrates, plants, invertebrates) used the default parameters ($k = 21$, $s = 1000$) [50].

These databases were grouped on the basis of shared seed values and parameters to optimize the screening efficiency. Table 1 summarizes their characteristics. All databases are publicly available through our project repository. For reproduction, detailed instructions are provided in Supplementary Material Section 2, Subsection “Reproducing Sketched Databases”.

Table 1. Reference sketched databases. Different seed values reflect the provenance of each sketch set. RefSeq88 sketch from Mash with seed 0, GTDB and custom sketches generated locally using default seed 42.

Sketch	Content	Sketch Parameters	Sketch Size	Seed Value
sketch1.msh	RefSeq nucleotide release 88	k=21, s=1000	1.2 GB	0
sketch2.msh	GTDB r202 Assembly Set, NCBI Complete Genomes Database + Custom databases (vertebrates, plants, invertebrates)	k=21, s=1000	883.25 MB	42
sketch3.msh	Custom databases (fungi, protozoa, archaea, virus)	k=15, s=5000	327.93 MB	42

Modular Reference Database Download

Candidate selection with dynamic threshold. After running Mash Screen, the output can be extensive, potentially including a large number of candidate genomes with varying degrees of similarity to the query sequences. Downloading and analyzing this entire list would be computationally inefficient and could introduce noise into subsequent analyses. On the other hand, setting an arbitrarily high static threshold might exclude important reference genomes, leading to incomplete coverage of the query sequences. To address these challenges, HYMET introduces a dynamic Mash-Screen thresholding mechanism to identify the most relevant candidate genomes and to create a targeted, input-specific database. The algorithm iteratively lowers the containment threshold in 0.02 steps until a minimum candidate count is reached (about 3.25 per input sequence, rounded; minimum five), or until a floor of 0.70 is reached.

Genome Retrieval. Following the selection of candidate genomes, their format was analyzed to enable efficient mapping and retrieval. These genomes were identified using RefSeq (GCF) and Genbank Assembly Genomes (GCA) accession numbers, unique identifiers assigned by NCBI. Thus, the NCBI Assembly database [60] was selected as the primary resource for constructing the reference database [10, 61]. To optimize the process, summary files from the NCBI Assembly database were downloaded, providing efficient access to metadata. A custom script was developed to map candidate genomes to these files using accession numbers. This script extracted the base accession number (e.g. “000169215”) to ensure compatibility between different assembly versions (e.g.: GCF_000169215.1, GCF_000169215.2), preventing retrieval failures due to version updates [60].

During the initial mutation study (conducted with the systematic-review harness), genomes were retrieved from the NCBI FTP service and decompressed locally. In all subsequent benchmarks and case studies in this manuscript, genomes were retrieved over HTTPS from NCBI and decompressed locally. The downloader uses bounded retries with exponential backoff and records failures in the run logs. To enhance efficiency, the script employed ThreadPoolExecutor for parallel downloads, allowing up to 64 concurrent threads. The taxonomy IDs (TaxID) of the assembly files were stored alongside the accession numbers and sequence identifiers, creating a comprehensive reference linking each genome to its taxonomic and sequence-level information [60, 61].

HYMET is designed to operate efficiently in network-constrained or offline environments. Users can supply a local directory containing mirrored NCBI assembly summaries to enable species-level candidate deduplication without requiring internet connectivity. Reference data are cached locally, and users have the option to preload these caches with curated FASTA files along with their corresponding sequence-to-TaxID mappings. Indices are automatically updated during subsequent analyses, ensuring reproducibility and consistency even in offline scenarios. Additional details can be found in “Reference retrieval policy and fallbacks” of the Supplementary Material.

Fast Alignment

In the second processing stage, HYMET employs Minimap2 for efficient and precise sequence alignment. This choice was motivated by Minimap2’s adaptive scoring system and its seed-chain-

extend *local* alignment strategy, which enables accurate mapping even with highly divergent sequences or incomplete reads, making the pipeline particularly resilient to common metagenomic challenges such as mutation-rich or fragmented samples [40, 41]. The pipeline uses minimizers to index reference sequences, enabling the rapid identification of alignment regions [62]. For contig/genome inputs we use the `-x asm10` preset, optimized for genome-to-genome alignment (approximately 10% divergence, or 90% identity) [63, 41]; for read inputs we use Minimap2's short-read preset `-x sr`. The results are saved in a PAF file, providing essential alignment details such as sequence IDs, lengths, positions, and mapping quality [41, 64].

Taxonomic Assignment

HYMET uses a hybrid taxonomic assignment strategy, combining the LCA algorithm with a weighted approach based on alignment coverage [23, 8, 24]. For exact matches, the reference's taxonomic lineage is directly assigned with a confidence score of 1.0. On the other hand, for non-exact matches, the weights for each TaxID are calculated according to:

$$\text{Weight} = \text{Coverage} \times \text{Abundance}, \tag{4}$$

where *Coverage* is the proportion of the query aligned with the reference and *Abundance* is the reference's frequency in the dataset. HYMET then computes a weighted consensus lineage across ranks and reports a single representative TaxID (the taxon with the highest cumulative weight across the supporting alignments). The confidence score is derived as the product of the per-rank consensus fractions.

$$\text{Confidence Score} = \prod_{i=1}^{n} \text{Confidence at Rank}_i, \tag{5}$$

where n is the number of ranks. This ensures higher consistency across ranks results in higher confidence scores. The final output includes the query identifier, a taxonomic lineage (kingdom to strain), the most specific rank, the representative NCBI TaxID, and a confidence score (0.0 to 1.0), reflecting the reliability of the classification [24, 65, 66]. The tab-delimited file `classified_sequences.tsv` contains the columns: Query, Lineage, Taxonomic Level, TaxID, Confidence.

All analyses, including tool evaluation, development, and validation, were conducted on a high-performance Linux-based virtual machine with 2 TB storage and 250 GB RAM. HYMET's performance was assessed using precision and F1 score metrics for classifying organisms across the three domains of life, considering taxonomic levels (kingdom to species) and mutation rates (0% to 30%). The analysis also examined the relationship between F1 scores, execution time, and resource usage (CPU and memory), ensuring a complete evaluation of precision and efficiency. The same methodology was applied to the other current state-of-the-art tools described in Section 1 to ensure a consistent comparison. Benchmarking was performed by comparing HYMET with general-purpose cross-domain workflows that produce per-read taxonomic assignments. Component-level short-read classifiers (e.g., KrakenUniq, Ganon, Centrifuge/Centrifuger) and compositisonal search methods (e.g., sourmash gather) were out of scope for head-to-head benchmarking; their behavior is represented through the workflows that include them. Detailed instructions for reproducing the benchmarking of these tools are provided in Supplementary Section 5.

Table 2. Composition of the test and validation dataset

Domain/Group	Number of GCFs	Size (GB)
Viruses	1 498	0.05
Other Vertebrates	43	2.83
Vertebrate Mammals	23	2.29
Protozoa	12	0.03
Plants	19	1.02
Invertebrates	43	1.14
Fungi	63	0.15
Bacteria	24 271	7.23
Archaea	231	0.05
**Total**	**26 203**	**14.76**

3.2 Test and Validation Dataset

The test dataset was derived from the NCBI RefSeq Assembly database (last modified: 13 October 2024), chosen for its curated and validated sequences [67, 10, 68]. Assembly summary files for all biological domains and viruses were downloaded and 10% of the entries were randomly selected based on GCF accession numbers. For each GCF, 10% of its genome sequences were further sampled to ensure proportional representation and mimic the fragmentation of metagenomic data [69]. This approach resulted in a diverse and representative dataset, as detailed in Table 2. For dataset replication, complete instructions and scripts are provided in the Supplementary Material Section 3, Subsection "Replicating the Benchmark Dataset".

3.3 CAMI Benchmarking Design

To assess the performance of HYMET alongside other baseline metagenomic classification tools, we performed standardized benchmarking using the CAMI (Critical Assessment of Metagenome Interpretation) datasets. Specifically, we selected seven publicly available contig assemblies representing various levels of complexity and ecological contexts. These include the Low, Medium, and High Complexity communities from CAMI I, the Mouse Gut, Marine, and Strain Madness panels from CAMI II, and the CAMI reference sample_0 (details are provided in Table 3).

We executed all tools strictly in contig mode to maintain consistent conditions across evaluations. To ensure fair comparisons, our benchmarking pipeline standardized input staging, fixed the number of computational threads, and maintained consistent input/output management. Additionally, it captured computational metrics such as wall-clock and CPU time, as well as peak memory usage.

The evaluation methodology adhered closely to established CAMI conventions. We reported metrics across taxonomic ranks from superkingdom down to species level, including both profile-based distances (L1 total variation and Bray–Curtis distance) and precision, recall, and F1-score for presence/absence classification (with a minimum abundance threshold of 0.1%). Contig-level accuracy was determined by directly comparing predicted taxonomic IDs (TaxIDs) with the CAMI-provided ground truth. Results were summarized individually for each sample and aggregated by taxonomic rank across all seven assemblies.

Tools included in the benchmarking process were HYMET, Kraken 2, Centrifuge, Ganon 2, TAMA, SqueezeMeta, ViWrap, MegaPath-Nano, BASTA, CAMITAX, MetaPhlAn 4, sourmash gather, phyloFlash, SnakeMAGs, and PhaBOX. Each tool was configured using its recommended database or default settings suitable for contig-based inputs. The specific software versions and database sources utilized are fully documented in the supplementary materials.

Table 3. CAMI benchmark samples and inputs used in the benchmark. All evaluations are contig based and scored against CAMI truth profiles and contig maps.

Sample ID	CAMI panel	Complexity/context	Input	Truth assets
cami_i_lc	CAMI I	Low complexity community	contigs	profile + contig truth
cami_i_mc	CAMI I	Medium complexity community	contigs	profile + contig truth
cami_i_hc	CAMI I	High complexity community	contigs	profile + contig truth
cami_ii_mousegut	CAMI II	Mouse gut metagenome	contigs	profile + contig truth
cami_ii_marine	CAMI II	Marine metagenome	contigs	profile + contig truth
cami_ii_strainmadness	CAMI II	Strain Madness (strain variation)	contigs	profile + contig truth
cami_sample_0	CAMI reference	Reference assembly	contigs	profile + contig truth

3.4 HYMET Contig vs Read Evaluation

To assess how input modality affects HYMET's performance, each CAMI assembly listed in Table 3 was analyzed under two conditions: first, using assembled contigs, and second, using synthetic reads generated from these same assemblies. Both modes followed the same general workflow—initial sketch-based screening for candidate references, construction or reuse of reference caches, sequence alignment, and weighted lowest-common-ancestor taxonomic assignment. Conditions such as computational threading, file handling, and benchmarking instrumentation remained consistent between modes. The primary distinction between the two analyses involved the alignment parameters and input handling. For the contig mode, HYMET employed alignment settings optimized specifically for longer, genome-to-genome comparisons. In contrast, the read mode processed single-end reads with alignment parameters tailored for shorter, fragmented sequences. Importantly, steps like candidate selection, reference caching, and taxonomic classification remained unchanged, ensuring comparability across modalities. For each assembly and input modality, the benchmarking framework calculated CAMI performance metrics at all taxonomic ranks, including L1 total variation, Bray–Curtis dissimilarity, precision, recall, and F1 scores. Additionally, wall-clock time and peak memory usage were recorded. Results were first summarized individually per sample, then aggregated as averages across all seven assemblies to enable a balanced comparison of performance between contig and read inputs.

3.5 Case-study Design: Gut and Zymo

To complement the CAMI benchmarks with real samples, we conducted two case studies that reflect common metagenomic contexts: a human gut metagenome assembly from MGnify and the ZymoBIOMICS mock community assembly curated by the Loman Lab. Both were processed in contig mode using the same workflow in Section 3.1, with a fixed number of threads, shared taxonomy inputs, and identical reference caching policy. This ensured that any differences in outcomes arise from sample biology rather than methodological drift.

The Zymo mock community provides a laboratory-defined composition and a canonical set of reference genomes. Ground truth was established at two levels: (i) contig-level labels by mapping assembled contigs to the curated Zymo reference panel and assigning each contig to a species TaxID; and (ii) a CAMI-style abundance profile used to compute rank-wise precision, recall, F1, and abundance distances (L1 total variation and Bray–Curtis). The human gut assembly does not have a strict gold standard; evaluation therefore emphasized plausibility of dominant taxa and concordance with public annotations, together with resource measurements.

For both samples, we recorded wall-clock time and peak resident memory for the complete pipeline. Table 4 summarizes the inputs and available truth assets.

In addition to the main Zymo case study, we conducted a targeted reference-ablation protocol to probe robustness to incomplete databases—an increasingly common scenario as public catalogues lag behind newly observed strains. Using the same reference panel employed in the case analysis, we progressively withheld the indexed sequences for each of the ten constituent organisms (TaxIDs 562, 28901, 1423, 1639, 1351, 1280, 1613, 287, 4932, 5207) at 0%, 25%, 50%, 75%, and 100% removal levels. After each removal step we rebuilt the reference index and repeated the full HYMET classification pipeline without changing any parameters. For every level we captured the distribution of contig assignments by taxonomic rank (species or strain, genus, family, and higher) alongside the standard rank-wise profile metrics (precision, recall, F1, L1 total variation, Bray–Curtis) and runtime measurements. This procedure isolates the impact of reference incompleteness while keeping the sample and analytical settings fixed, thereby reflecting realistic deployments in which key genomes are missing or outdated.

Table 4. Case-study samples and inputs. The Zymo mock community is evaluated against curated contig-level labels and a CAMI-style profile; the gut assembly is assessed descriptively in the absence of a ground-truth profile.

Sample ID	Source	Context	Input	Truth assets
zymo_mc	ZymoBIOMICS mock community (Loman Lab assembly)	Even bacterial/fungal mix	contigs	contig labels + CAMI profile
gut_case	MGnify MGYS00006849; SRS9791096; SRR15489027; ERZ24911249; MGYA00794604	Human stool metagenome	contigs	none (top-taxa comparison)

4 Results

Benchmark Scope and Replicates

We report results over seven CAMI assemblies (Table 3): CAMI I low/medium/high complexity; CAMI II mouse gut, marine, and strain madness; and the CAMI reference sample_0. Each assembly was processed once per tool under fixed threads, yielding one run per sample per tool; no technical replicates were used. Rank-wise summaries reflect means across the seven assemblies. The manifest that enumerates these samples is versioned in the repository.

For HYMET's modality comparison, the same seven assemblies were analysed twice: (i) contigs as provided; and (ii) deterministic synthetic reads created by windowing contigs into 250 bp slices with a 125 bp minimum tail. Aside from input handling and Minimap2 presets, all pipeline stages (candidate selection, cache construction, alignment, weighted-LCA) and resource controls were identical; again, one run per sample per mode (no replicates).

The mutation-sweep experiment spans nine higher-level groups (Viruses, Archaea, Bacteria, Fungi, Plants, Protozoa, Invertebrates, Vertebrate Mammals, Other Vertebrates). For each group, we generated one mutated contig set per rate between 0% and 30% using reproducible seeds (substitutions with short indels), then computed precision/recall/F1 by rank from contig-level truth. No technical replicates were used in this sweep.

Real-data evaluations comprise two case studies (MGnify gut assembly and ZymoBIOMICS mock community), each run once in contig mode using the same workflow as above (Section 3.1).

Performance Evaluation

All quantitative evaluations in this revision are contig based. HYMET also accepts read inputs via the unified CLI (Minimap2 `sr` preset), but a dedicated raw-read benchmark is deferred to a follow-up to avoid mixing modalities. Against contemporary baselines, HYMET maintains high F1 across ranks and taxonomic groups; many competitors lose precision and recall at lower ranks, whereas HYMET preserves balanced performance into genus and species, particularly for microbes and small eukaryotes. Viruses remain the most sensitive to divergence, with modest drops at intermediate ranks, but species-level accuracy remains competitive.

Mutation Resilience

HYMET's performance proves exceptionally stable under varying mutation rates (0–30%), outperforming all benchmarked tools in both accuracy and consistency (Figure 3). Viral classification shows a progressive decline at extreme mutations (F1 scores approximately 0.5 at thirty percent), while archaea, invertebrates and fungi maintain F1 scores over 0.9. Other groups show only minor, non-significant reductions, staying above 0.8 (Figure 4). This contrasts with competing tools, where their scores decline as the mutation rate increases (Supplementary Figures 1-2).

Computational Efficiency

We report resource usage for two CAMI suites that share the same assemblies but differ in the candidate-reference budget. In the canonical multi-tool suite (tight candidate cap with species deduplication), HYMET builds smaller caches and averages approximately 116 seconds wall-clock and 6.2 GB peak resident memory across the seven assemblies (Figure 7; Table 6). Baselines in this suite illustrate distinct speed–memory–accuracy trade-offs (e.g., MetaPhlAn 4: 147 s, 18.8 GB; Kraken 2: 40 s, 11.0 GB; MegaPath-Nano: 24 s, 10.2 GB; TAMA: 56 s, 16.9 GB).

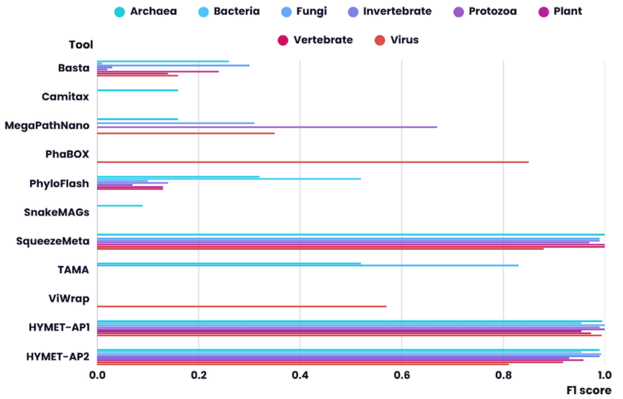

Figure 2. F1 scores achieved by various taxonomic classification tools, including both state-of-the-art tools and HYMET, across different taxonomic groups. The x-axis represents the F1 scores, ranging from 0 to 1, while the y-axis lists the evaluated tools. Each bar is color-coded to indicate the corresponding taxonomic group. This data is based on analysis with 0% mutation rate.

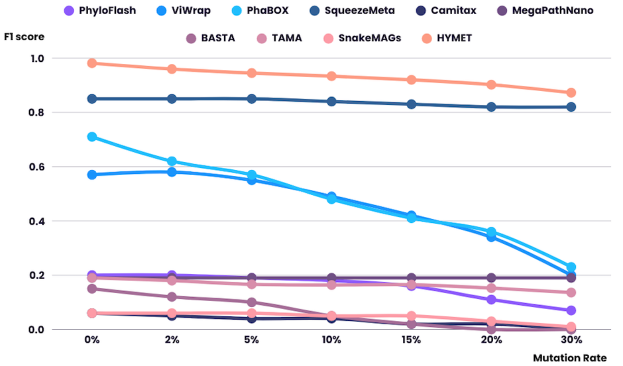

Figure 3. Performance of the state-of-the-art tools and HYMET as the mutation rate increases. The x-axis represents the mutation rate (ranging from 0% to 30%), while the y-axis shows the F1 score. Each curve on the graph corresponds to a different tool.

To isolate input-modality effects, the HYMET-only reads-vs-contigs suite deliberately expands the candidate budget so both modes reuse the same, larger cache. Under this regime, HYMET's contig runs average 361 seconds and 17.37 GB peak memory, while the synthetic-read runs average 334 seconds and 17.36 GB (Figure 9; Table 7). The near-identical memory stems from the shared cache; the modest runtime delta arises primarily from Minimap2 presets (`asm10` for contigs vs `sr` for reads), not from differences in the search space.

The case studies align with the expanded-budget envelope. The MGnify gut assembly and the ZymoBIOMICS mock community each completed in 4–5 minutes with 17.4 GB peak memory on the reference machine (Table 9), consistent with the reads-vs-contigs suite and illustrating predictable behavior on real assemblies. In practice, users can choose between these profiles by selecting a tighter candidate cap (minutes-scale, lower memory) or an expanded budget (longer runs with larger caches that can improve

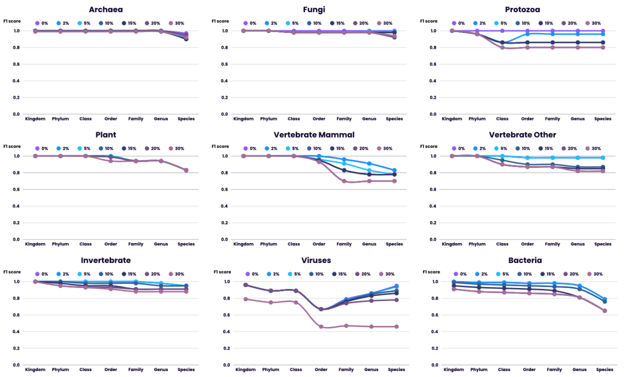

Figure 4. Performance of HYMET across taxonomic levels for different taxonomic groups as mutation rates increase (0% to 30%). The x-axis shows taxonomic levels (kingdom to species), and the y-axis represents the F1 score. Each curve corresponds to a specific mutation rate, and each graph focuses on a particular taxonomic group.

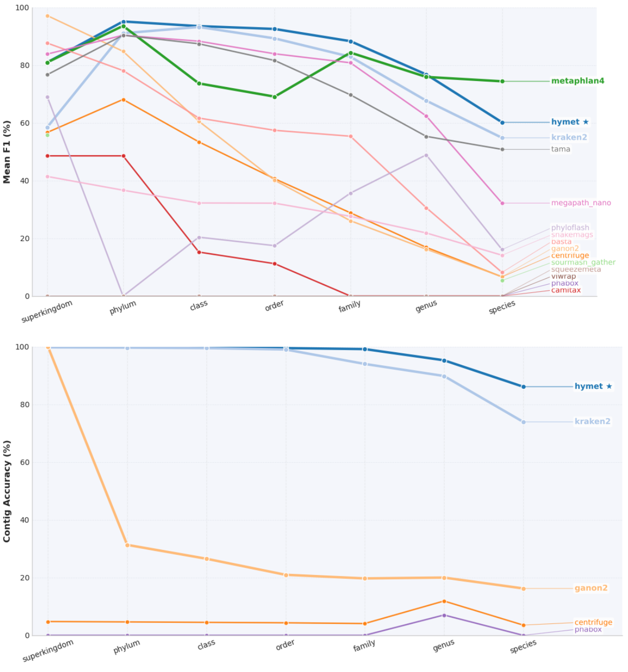

Figure 5. Top: mean F1 by rank (superkingdom to species) across seven assemblies. Bottom: mean contig-level accuracy by rank.

sensitivity). Install footprint remains 2.82 GB; dynamically downloaded references typically add 10–50 GB (disk-footprint details are provided in the Supplementary Material).

CAMI Benchmark

We benchmarked HYMET and several contig-mode classification tools using diverse CAMI assemblies. Figures 5–7 summarize the classification performance at different taxonomic ranks and resource usage across these tools.

Figure 5 (top) shows that HYMET consistently outperforms most other tools at various taxonomic ranks, particularly from family up to class. While MetaPhlAn 4 achieves high species-level accuracy, it underperforms at intermediate ranks. Index-based classifiers like Kraken 2 and Centrifuge demonstrate reduced accuracy at lower taxonomic ranks due to lower recall rates. Figure 5 (bottom) highlights the stability and consistency of HYMET's contig-level accuracy across ranks, contrasting sharply with tools relying solely on k-mer indices, which exhibit more variability at lower ranks. Only tools that emit per-contig classifications (HYMET, Kraken 2, Centrifuge, Ganon 2, etc.) appear in the contig-accuracy panel because marker-based profilers and read-focused workflows do not produce contig-level outputs.

Profile-distance metrics reinforce HYMET's advantage. As

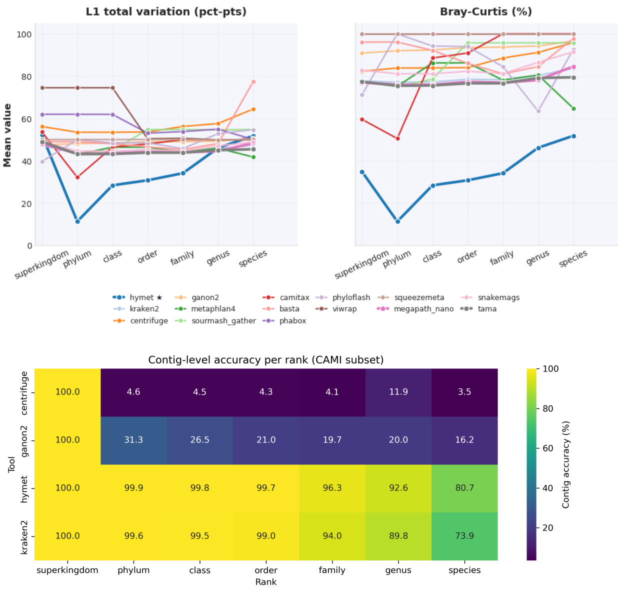

Figure 6. Top: mean L1 total variation and Bray–Curtis dissimilarity by rank across the seven CAMI assemblies. Bottom: per-rank CAMI accuracy heatmap for the profilers benchmarked.

Table 5. CAMI profile-distance summary (mean across taxonomic ranks and assemblies). Lower values indicate improved alignment with CAMI truth profiles.

Tool	Mean L1 (pct pts)	Mean Bray–Curtis (%)	Mean F1 (%)
HYMET	36.35	33.88	83.89
MetaPhlAn 4	45.21	78.34	78.83
Kraken 2	45.21	78.96	76.76
TAMA	44.76	77.17	73.11
MegaPath-Nano	45.10	77.95	74.53

shown in Figure 6 (top), HYMET attains the lowest Bray–Curtis dissimilarity at all ranks and the lowest or near-lowest L1 at most ranks (with genus, species, and superkingdom showing narrow leads by MegaPath-Nano, MetaPhlAn 4, and phyloFlash, respectively). This indicates tighter abundance estimates than competing profilers overall. The heatmap in Figure 6 (bottom) illustrates how this translates into rank-wise accuracy breadth, particularly below the family level where index-only tools lose recall. The heatmap is restricted to tools that emit per-contig assignments (HYMET and the index-only classifiers Kraken 2, Centrifuge, and Ganon 2) because marker-based profilers (e.g., MetaPhlAn 4) and pipeline workflows (e.g., TAMA, SqueezeMeta) report profiles only and do not provide per-contig labels.

Table 5 complements these plots: HYMET lowers mean L1 deviation by roughly nine percentage points relative to Kraken 2 and MetaPhlAn 4 while retaining the strongest average F1. Tools optimized for speed, such as MegaPath-Nano, exhibit higher profile distances despite competitive F1 values, highlighting the trade-off between coarse abundance estimates and precise community reconstruction.

Resource utilization data (Figure 7) shows HYMET's balanced performance, averaging approximately 116 seconds of wall-clock time and 6.2 GB of peak memory usage across the seven assemblies. MetaPhlAn 4 required greater resources (about 147 seconds and 18.8 GB), while Kraken 2 and MegaPath-Nano operated faster (approximately 40 and 24 seconds, respectively) but sacrificed accuracy at finer taxonomic resolutions. TAMA provided balanced precision but demanded higher memory usage (around 16.9 GB).

Table 6 summarizes these performance trends. Overall, HYMET delivered the highest genus-level F1 score (76.75%), a competitive species-level F1 score (60.18%), and the strongest overall average across ranks (83.89%) driven by balanced precision and recall (62.59% / 62.00%). MetaPhlAn 4 excelled at species-level accuracy

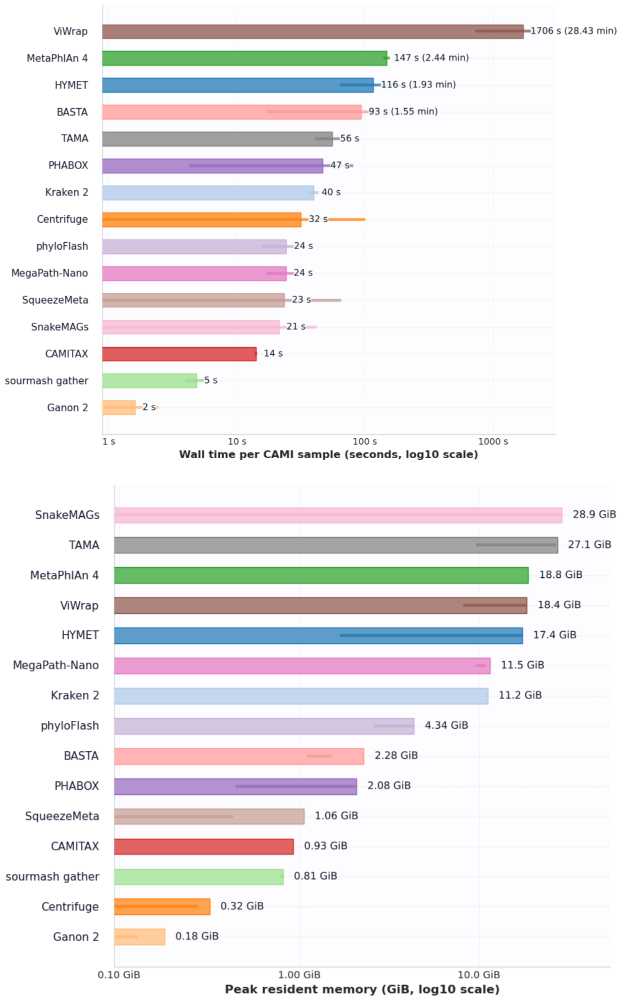

Figure 7. CAMI resource envelopes. Aggregated wall-clock time (top) and peak resident memory (bottom) per tool across the seven assemblies (canonical multi-tool suite; tight candidate cap).

Table 6. CAMI summary: mean F1 (%) at genus, species, and across all ranks (Avg F1), mean precision/recall at species, mean wall time (s) and mean peak memory (GB) across seven assemblies (canonical multi-tool suite; tight candidate cap).

Tool	Genus F1	Species F1	Avg F1	Species Precision	Species Recall	Wall time	Peak GB
HYMET	76.75	60.18	83.89	62.59	62.00	115.93	6.24
MetaPhlAn 4	75.90	74.38	78.83	75.46	77.92	146.54	18.76
Kraken 2	67.68	54.81	76.76	69.40	47.19	39.86	10.95
TAMA	55.31	50.81	73.11	79.52	40.14	55.61	16.88
MegaPath-Nano	62.38	32.19	74.53	45.63	25.54	24.11	10.20

(74.38%, precision 75.46%, recall 77.92%) but lagged at intermediate ranks, yielding a lower overall F1 average (78.83%). Kraken 2 and MegaPath-Nano completed faster but showed reduced sensitivity at lower ranks (species F1: 54.81% and 32.19%; averages 76.76% and 74.53%) with skewed precision/recall. TAMA balanced precision (79.52%) against higher memory usage (16.88 GB) and obtained an overall average of 73.11%. Together with Figure 5, these results show that HYMET's hybrid design sustains recall into lower ranks without sacrificing precision or inflating resource costs.

HYMET Read vs Contig Modes

Figures 8 and 9 provide a detailed comparison between HYMET's contig-based workflow and its synthetic-read workflow across CAMI assemblies, highlighting differences in rank-wise performance and computational resource usage. Aggregated metrics that support these visual comparisons are summarized comprehensively in Table 7.

As illustrated in Figure 8, both workflows exhibit closely matched F1 scores across taxonomic ranks. However, the synthetic-read approach demonstrates modestly improved abundance distance metrics at intermediate and lower ranks, as evidenced by lower L1 total variation and Bray–Curtis dissimilarity scores.

The resource usage depicted in Figure 9 confirms negligible differences between the two workflows in terms of wall-clock time and memory consumption. This similarity arises because both modes employ identical downstream reference database construction, alignment strategies, and classification methodologies.

Table 7 quantifies these observations, showing that while the synthetic-read workflow achieves comparable recall and overall F1 scores relative to the contig-based workflow (F1: 83.37 vs. 84.41), it provides slightly improved abundance distances (L1: 28.63 vs. 34.55; Bray–Curtis: 26.49 vs. 32.09). Furthermore, both workflows demonstrate similar resource efficiency in terms of execution time

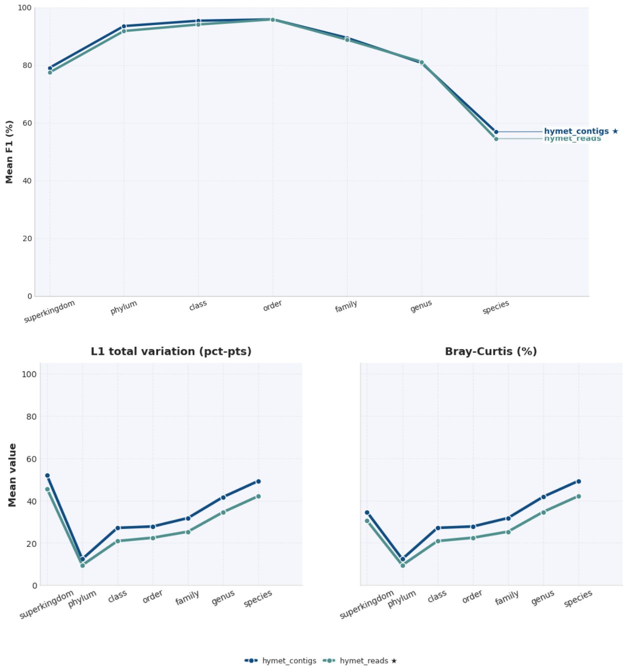

Figure 8. Comparison of HYMET's contig and synthetic-read workflows across CAMI assemblies. Top panel: Mean F1 scores across taxonomic ranks (superkingdom to species). Bottom panel: Mean abundance distances, with L1 total variation represented by solid lines and Bray–Curtis dissimilarity represented by dashed lines.

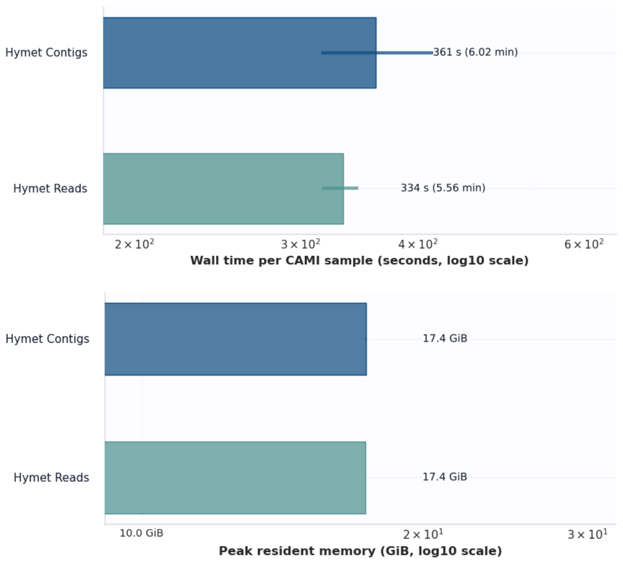

Figure 9. Resource usage comparison between HYMET contig and read workflows, measured across seven CAMI assemblies (HYMET-only suite; expanded candidate budget). Top panel: Wall-clock execution time (seconds). Bottom panel: Peak memory usage (GB).

Table 7. HYMET contig vs read comparison across seven CAMI assemblies: mean precision/recall/F1, mean L1 total variation (percentage points), Bray–Curtis (%), wall-clock time (s), and peak resident memory (GB) (HYMET-only suite; expanded candidate budget).

Mode	Precision	Recall	F1	L1	Bray–Curtis	Wall time	Peak GB
HYMET (contigs)	82.15	89.90	84.41	34.55	32.09	361.19	17.37
HYMET (reads)	80.05	90.57	83.37	28.63	26.49	333.61	17.36

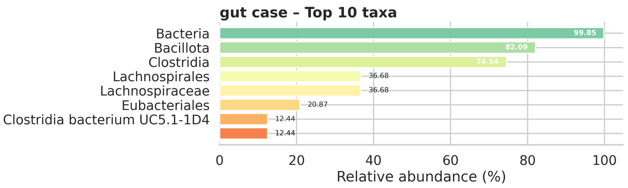

Figure 10. Top taxa panel for the human gut assembly. Panels display the most abundant ranks from superkingdom through species for this sample.

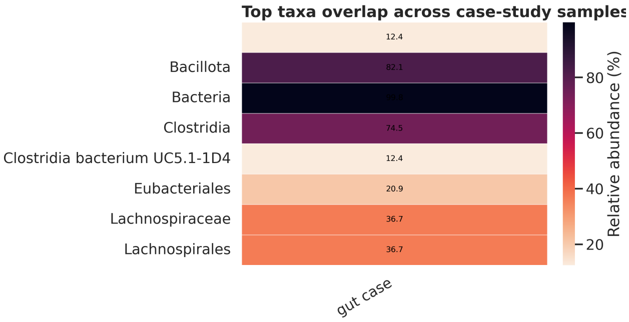

Figure 11. Abundance heatmap for the gut assembly. Color intensity encodes relative abundance across taxonomic ranks; darker cells indicate higher contributions.

(333.61 vs. 361.19 seconds) and memory usage (17.36 vs. 17.37 GB), ilustrating the robustness of HYMET's pipeline across different input modalities.

Gut and Zymo Case-study Results

To extend the evaluation beyond CAMI benchmarks, we analyzed two real-world metagenomic datasets: the human gut assembly from MGnify and the ZymoBIOMICS mock community from the Loman Lab. Figures 10 and 12 illustrate the primary taxonomic profiles for these samples, while Figures 11 and 13 provide detailed abundance heatmaps. Computational resource usage for both analyses is summarized in Table 9.

At the contig level, HYMET assigned species/strain labels to 82.82% of gut contigs; in the Zymo mock, HYMET produced species-level labels for 75.00% of predicted contigs (54/72), and among truth-matched contigs (n=61) exactly matched the curated species for 44.26% (27/61; 56.25% of species-assigned), consistent with genus-level substitutions.

In the human gut sample, HYMET identified a microbiome predominantly composed of Bacillota (Firmicutes; 82.09%), followed by significant contributions from Pseudomonadota (9.59%) and Actinomycetota (8.17%) at the phylum level. At the class level, Clostridia strongly dominated (74.54%), particularly represented by the orders Lachnospirales (36.68%) and Eubacteriales (20.87%), with Lachnospiraceae notably prevalent at the family level. These findings reflect typical adult gut microbiomes characterized by obligate anaerobic bacteria. Among the identified species, the most abundant were *Clostridia bacterium UC5.1-1D4* (12.44%), *[Clostridium] scindens* (9.49%), *Coprococcus phoceensis* (6.85%), *Longicatena caecimuris* (6.53%), *Roseburia intestinalis* (6.21%), and *Ruthenibacterium lactatiformans* (5.61%). Additionally, lower-level signals such as *Escherichia sp. KTE172* (5.47%) were also detected (Figure 10). The gut community heatmap highlighted dense abundance clusters within the Lachnospiraceae and Oscillospiraceae families, reinforcing these results (Figure 11).

The Zymo mock community comprises ten known organisms,

Table 8. Zymo bacterial species: truth (renormalized within Bacteria) versus HYMET profile. Notes indicate genus-level matches where the exact species differs.

Species	Within-Bacteria (%)		Note
	Truth	HYMET	
Escherichia coli	18.15	22.22	exact
Salmonella enterica	21.20	12.50	exact
Bacillus subtilis	16.33	15.28	genus match (B. spizizenii)
Listeria monocytogenes	12.12	13.89	exact
Enterococcus faecalis	11.54	8.33	exact
Staphylococcus aureus	11.23	9.72	exact
Limosilactobacillus fermentum	8.23	6.94	exact
Pseudomonas aeruginosa	1.20	4.17	genus match (Pseudomonas sp.)

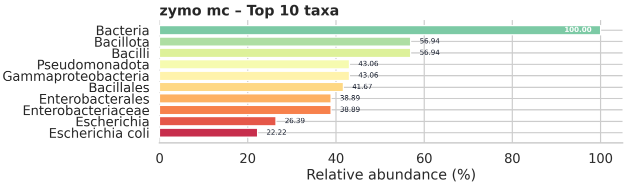

Figure 12. Top taxa panel for the Zymo mock community. Panels display the most abundant ranks across superkingdom through species for this sample.

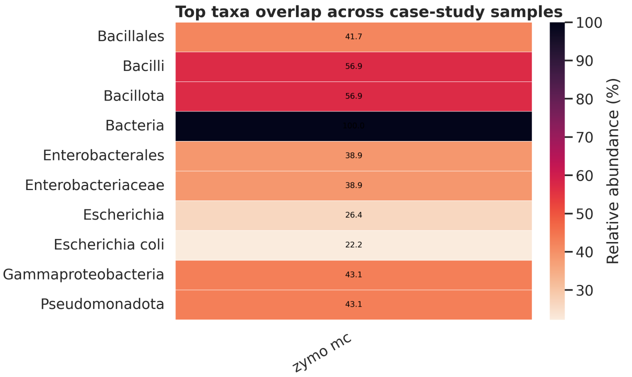

Figure 13. Abundance heatmap for the Zymo mock community. Color intensity encodes relative abundance across taxonomic ranks; darker cells indicate higher contributions.

including eight bacterial species and two yeasts (*Saccharomyces cerevisiae* and *Cryptococcus neoformans*). Although yeasts represent 46.80% of the truth profile, in this run HYMET did not recover the eukaryotic component, and the reported profile is restricted to bacterial taxa (53.20%). Within bacteria, HYMET accurately identified major species such as *Escherichia coli* (22.22%), *Listeria monocytogenes* (13.89%), and *Salmonella enterica* (12.50%). Minor genus-level substitutions occurred: *Bacillus* spizizenii in place of *B. subtilis*, and *Pseudomonas* sp. in place of *P. aeruginosa*. Table 8 compares the bacterial abundances (renormalized within bacteria) to HYMET's predictions, showing six exact species matches and two genus-level substitutions. The Zymo abundance patterns concentrate within Enterobacterales and Bacillales, consistent with Figure 12 and Figure 13.

Computationally, both datasets exhibited similar performance metrics. HYMET completed the analyses efficiently in approximately 4–5 minutes each, with peak memory usage consistently around 17.4 GB, showcasing the robustness and scalability of the method across diverse metagenomic contexts (Table 9).

Table 9. Case-study runtime and memory summary on the reference machine.

Sample	Wall time (s)	Peak RSS (GB)
gut_case	258.32	17.41
zymo_mc	255.73	17.42

5 Zymo Ablation Results

To assess robustness to incomplete references, we ran an ablation study on the Zymo dataset using the canonical suite, progressively removing species/strain-level entries and re-indexing at each level. Across ablation levels, runtime and memory remained stable (~4.2–4.5 minutes, ~17.4 GB). Classification quality degrades as expected at finer ranks: the share of species/strain assignments falls from 75.00% to 64.52%, while higher/unknown rises from 25.00% to 35.48%; genus F1 drops from 88.89% to 58.82% and species F1 from 57.14% to 0.00% at full ablation.

Table 10. Zymo ablation summary. Levels indicate the ablation proportion; totals are number of contigs classified.

Level (%)	Total classified	Species/strain (%)	Higher/unknown (%)	Genus F1 (%)	Species F1 (%)
0	72	75.00	25.00	88.89	57.14
25	69	72.46	27.54	88.89	57.14
50	71	73.24	26.76	88.89	43.48
75	66	66.67	33.33	73.68	13.79
100	62	64.52	35.48	58.82	0.00

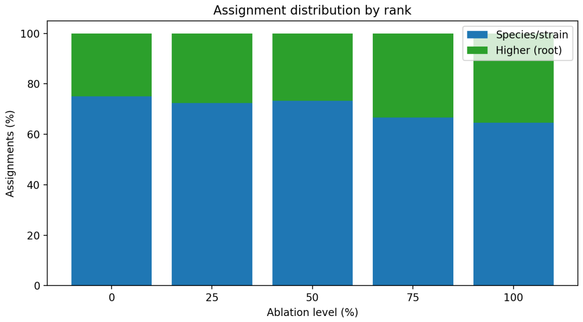

Figure 14. Ablation o distribution of assignments by rank group across ablation levels on Zymo.

Figure 14 shows a gradual reweighting from species/strain toward higher ranks as references are withheld: species/strain drops from 75.00% to 64.52%, while higher/unknown rises from 25.00% to 35.48%; the number of classified contigs declines modestly (72 → 62). Genus and family contributions remain stable through 50% ablation and only drift at ≥75%, indicating HYMET backs off to appropriate higher ranks rather than emitting incorrect fine-grained labels when exact references are missing.

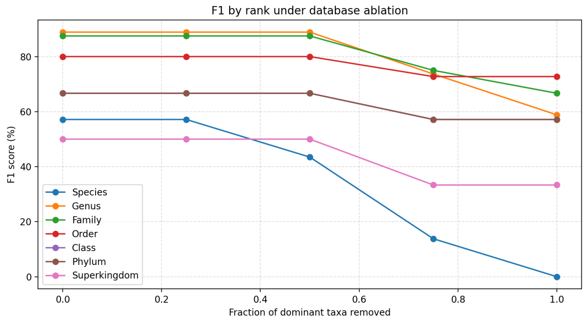

Figure 15. Ablation on Zymo (canonical): F1 by rank under increasing ablation.

Rank-wise accuracy (Figure 15) remains flat at coarse levels, while genus F1 stays high through 50% (88.89%) and then declines at 75% (73.68%) and 100% (58.82%). Species F1 degrades from 57.14% to 0.00% under full ablation, reflecting the intended removal of discriminative references. Throughout, runtime and memory are effectively unchanged (~4.2–4.5 minutes; ~17.4 GB), isolating reference incompleteness as the driver of accuracy loss rather than compute differences.

6 Discussion

HYMET effectively addresses significant challenges in metagenomic analysis by integrating adaptive MinHash-based prefiltering with precise alignment and a coverage-weighted Lowest Common Ancestor (LCA) classification. This hybrid design builds upon previous approaches such as Metalign's combination of CMash and Minimap2, yet it uniquely introduces an adaptive candidate selection method, dynamically generated cross-domain reference databases, and an evidence-weighted LCA step. Compared to fixed-threshold methods, such as CAMITAX, HYMET's adaptive strategy enhances sensitivity to divergent organisms, avoiding the exclusion of potentially relevant candidates and ensuring broader domain applicability [4, 70].

Our evaluations across diverse CAMI benchmarks demonstrate HYMET's robust accuracy, computational efficiency, and stable performance across taxonomic ranks. Specifically, HYMET achieved a mean F1 of 83.89% across ranks, a strong genus-level F1 of 76.75%, and a competitive species-level F1 of 60.18%, while maintaining low computational resource demands (average runtime of 116 seconds and peak memory usage of approximately 6.2 GB). Unlike marker-based tools such as MetaPhlAn 4, which excel at species-level assignments but sacrifice intermediate-rank accuracy and per-contig labeling capabilities, HYMET balances precision and recall consistently across multiple ranks, which is crucial for downstream metagenomic workflows such as genome binning [71, 15, 23].

HYMET's adaptive cache management strategy allows predictable and flexible resource usage. Both contig and synthetic-read analyses converged in memory usage when sharing reference caches, differing slightly only in runtime due to alignment parameter presets. Practically, users can thus optimize for rapid exploratory analyses or deeper comparative studies across related samples by adjusting candidate cache parameters without compromising accuracy.

Importantly, HYMET demonstrates resilience against genetic mutations, maintaining robust performance ($F1 \geq 0.8$) for diverse taxonomic groups even at mutation rates up to 30%. While viral sequences experienced a notable decrease in accuracy ($F1 \approx 0.5$ at 30%), this likely reflects genuine biological and database limitations rather than methodological shortcomings. The stability in performance arises primarily from the optimized choice of screening parameters—smaller k-mers and larger sketch sizes for divergent genomes—and the robust seed-chain-extend alignment method employed by Minimap2 [20, 41].

The ZymoBIOMICS case study provided additional insight into HYMET's limitations. While bacterial taxa were accurately classified (six exact matches, two genus-level substitutions), the method failed to recover yeast species, which represented a significant proportion of the mock community. The systematic ablation experiment further clarified this behavior, showing HYMET appropriately adjusts assignments to higher taxonomic ranks when species-level references are incomplete or missing. This indicates that future versions could benefit significantly from domain-specific adjustments to candidate thresholds, particularly enhancing sensitivity towards underrepresented groups like yeasts.

A notable operational challenge identified was related to reference database completeness and retrieval reliability. The hybrid reference databases, combining older public sketches with newly generated local sketches, exhibited a non-negligible retrieval failure rate (6.04%), significantly impacting classification accuracy, particularly at lower taxonomic levels. This emphasizes the importance of maintaining continuously updated, comprehensive databases, systematically refreshing manifests, and implementing robust checksum and fallback protocols to mitigate data retrieval issues [67, 17].

HYMET demonstrated robust accuracy across bacteria, archaea, fungi, and small eukaryotes, emphasizing its suitability across diverse microbial communities. However, challenges remain in classifying more complex eukaryotic organisms due to inherent genome complexity, substantial intra-species diversity, and biases towards well-studied taxa in reference databases [72]. Similarly, the polyphyletic and rapidly evolving nature of viral genomes underscores the importance of regularly updated, comprehensive databases to maintain accurate viral taxonomic classification [73, 74].

Future improvements should focus on directly addressing identified limitations: enhancing domain-specific candidate thresholds (particularly for viruses and eukaryotes), implementing scheduled updates of sketch databases enriched with underrepresented taxa, refining the weighted LCA with explicit rules for resolving multi-mapped alignments and establishing minimum evidence thresholds. Integrating uncertainty quantification methods, such as bootstrapping or replicate analyses, will further enhance the reliability and practical applicability of HYMET.

7 Conclusion

HYMET advances metagenomic classification by integrating adaptive MinHash screening, precise Minimap2 alignment, and a coverage-weighted Lowest Common Ancestor algorithm. Evaluations across diverse CAMI benchmarks demonstrate that HYMET achieves robust accuracy, consistently outperforming or matching existing methods at multiple taxonomic ranks, notably with an average F1 score of 83.89%, including 76.75% at the genus level and 60.18% at the species level. This high performance is maintained even under substantial genetic divergence, indicating strong mutation resilience.

Real-world validations further confirm HYMET's practical value, successfully identifying expected bacterial communities from human gut and ZymoBIOMICS mock samples. Computationally efficient and lightweight, HYMET's adaptive and dynamic caching strategy ensures reproducible and resource-predictable analyses suitable for diverse deployment contexts.

While current results illustrate excellent overall accuracy and scalability, future enhancements could include domain-specific reference optimizations, explicit handling of multi-mapping ambiguities, and integration of machine learning classifiers to further improve lower-rank discrimination. Such developments would position HYMET as a versatile platform, supporting reliable, efficient, and scalable metagenomic investigations across various biological contexts.

8 Availability of source code and requirements

- Project name: HYMET (Hybrid Metagenomic Tool)
- Project home page: `https://github.com/ieeta-pt/HYMET`
- Operating system(s): Linux
- Programming language: Python (primary), Perl (legacy), Bash
- Other requirements: Docker or Apptainer/Singularity; Conda/Mamba
- License: MIT.

9 Data Availability

The supplementary material describes the full reproducibility workflow (environment setup, scripts, execution logs, and additional figures). Digital resources used in the manuscript are listed below.

- HYMET source code, CAMI/reads-versus-contigs/case manifests, runtime logs, and aggregated TSV/figure outputs are versioned in the project repository (`https://github.com/ieeta-pt/HYMET`; see `results/`, `bench/`, and `case/` subdirectories).
- The Syst_Review repository retains the systematic benchmark harness for third-party tools, including installation recipes and evaluation scripts (`https://github.com/inesbmartins02/Syst_Review`).
- The Mash sketch databases used for candidate selection (`sketch1.msh`, `sketch2.msh`, `sketch3.msh`) are deposited at Zenodo (doi:10.5281/zenodo.17428354); checksums are mirrored in the repository (`sketch\_sha256.txt`).
- CAMI contig assemblies used in the benchmark are enumerated in `bench/cami_manifest.tsv`; running `bench/fetch_cami.sh` downloads the official CAMI I sample_0 bundle from the Publisso mirror (`https://frl.publisso.de/data/frl:6421672/dataset/2017.12.29_11.37.26_sample_0_contigs.tar`), extracts the required assets into `/data/cami/`, caches the archive, and regenerates the lightweight subsets (`cami_i_*`, `cami_ii_*`) via `tools/generate_cami_subsets.py` so every benchmark run starts from the same inputs.
- The test and validation dataset (26,203 genomes, 14.76 GB) was derived from the NCBI RefSeq Assembly database (`https://ftp.ncbi.nlm.nih.gov/genomes/refseq/`); assembly summary files and scripts for replication are provided in Supplementary Material Section 3.
- Reference genomes for dynamic database construction are retrieved from the NCBI Assembly database (`https://www.ncbi.nlm.nih.gov/assembly/`) using GCF and GCA accession numbers identified through Mash screening. GTDB r202 genomes included in the sketch databases are available from the Genome Taxonomy Database (`https://gtdb.ecogenomic.org/`).
- Case-study inputs can be fetched with `case/fetch_case_data.sh`, which retrieves the Zymo mock assembly (Nanopore S3: `http://nanopore.s3.climb.ac.uk/mockcommunity/v3/7cd60d3b-eafb-48d1-9aab-c8701232f2f8.ctg.cns.fa`) and the MGnify gut assembly (EBI API: `https://www.ebi.ac.uk/metagenomics/api/v1/analyses/MGYA00794604/file/ERZ24911249_FASTA.fasta.gz`) before staging them under `/data/case/`. The manifest `case/manifest.tsv` records the same paths for reference.
- Zymo ablation outputs (candidate lists, cached references, evaluation tables, figures) are versioned under `results/ablation/canonical/run_20251031T191804Z/`; the workflow can be rerun with `case/run_ablation.sh` as documented in Supplementary Section 6.

10 Additional Files

Supplementary Tab. S1. On-disk resource footprint (install + references) for every benchmarked tool in the canonical CAMI environ-

ment.

Supplementary Tab. S2. Provenance and composition of the reference databases used by each tool, including shared corpora and official releases.

Supplementary Tab. S3 to S10. Per-domain benchmarking tables (viruses, archaea, bacteria, fungi, protozoa, plants, invertebrates, vertebrates) reporting precision, recall, and F1 across taxonomic ranks at 0% mutation.

Supplementary Tab. S11 to S17. HYMET-only benchmarking tables summarizing precision, recall, and F1 across taxonomic ranks under mutation rates from 0% to 30%.

Supplementary Fig. S1 and S2. Line plots showing how tool performance varies with mutation rate across taxonomic levels for each biological domain.

Supplementary Fig. S3. Scatter plots relating execution time (hours) to F1 score (0.0–1.0) for all evaluated tools, presented per domain.

10.1 List of abbreviations

BASTA: BAsic Sequence Taxonomy Annotation;
BLAST: Basic Local Alignment Search Tool;
CAMI: Critical Assessment of Metagenome Interpretation;
CAMITAX: CAMI TAXonomy (tool for taxon labels);
CMash: Containment MinHash;
CPU: Central Processing Unit;
CSV: Comma-Separated Values;
DOI: Digital Object Identifier;
F1: Harmonic mean of precision and recall;
FCT: Fundação para a Ciência e a Tecnologia;
GCA: GenBank assembly accession;
GCF: RefSeq assembly accession;
GTDB: Genome Taxonomy Database;
HYMET: Hybrid Metagenomic Tool;
LCA: Lowest Common Ancestor;
NCBI: National Center for Biotechnology Information;
PAF: Pairwise mApping Format;
RefSeq: NCBI Reference Sequence database;
RSS: Resident Set Size;
SSU rRNA: Small Subunit ribosomal RNA;
TAMA: Taxonomy Analysis pipeline for metagenome using Meta-Analysis;
TaxID: Taxonomy Identifier;
TSV: Tab-Separated Values.

Funding

This work has received funding from the FCT (Foundation for Science and Technology) under unit 00127-IEETA and through the project Advanced Genomic Data Processing in Portuguese FEGA Node (ref. 2023.14342.CPCA.A1; DOI: 10.54499/2023.14342.CPCA.A1). J.M.S. has received funding from the European Commission under grant agreement 101081813 (Genomic Data Infrastructure).

References

1. Kim D, Song L, Breitwieser FP, Salzberg SL. Centrifuge: rapid and sensitive classification of metagenomic sequences. Genome research 2016;26(12):1721–1729.
2. Simon HY, Siddle KJ, Park DJ, Sabeti PC. Benchmarking metagenomics tools for taxonomic classification. Cell 2019;178(4):779–794.
3. Wood DE, Salzberg SL. Kraken: ultrafast metagenomic sequence classification using exact alignments. Genome biology 2014;15(3):1–12.
4. Bremges A, Fritz A, McHardy AC. CAMITAX: Taxon labels for microbial genomes. GigaScience 2020;9(1):giz154.
5. Kim N, Ma J, Kim W, Kim J, Belenky P, Lee I. Genome-resolved metagenomics: a game changer for microbiome medicine. Experimental & Molecular Medicine 2024;56(7):1501–1512.
6. Mallawaarachchi V, Lin Y. Accurate binning of metagenomic contigs using composition, coverage, and assembly graphs. Journal of Computational Biology 2022;29(12):1357–1376.
7. Ayling M, Clark MD, Leggett RM. New approaches for metagenome assembly with short reads. Briefings in bioinformatics 2020;21(2):584–594.
8. Wood DE, Lu J, Langmead B. Improved metagenomic analysis with Kraken 2. Genome biology 2019;20:1–13.
9. Lema NK, Gemeda MT, Woldesemayat AA. Recent Advances in Metagenomic Approaches, Applications, and Challenges. Current Microbiology 2023;80(11):347.
10. Martins IB, Miguel Silva J, Almeida JR. A comprehensive study of databases to assess the reliability of metagenomic tools. In: 2024 IEEE Conference on Computational Intelligence in Bioinformatics and Computational Biology (CIBCB); 2024. p. 1–6.
11. Xu R, Rajeev S, Salvador LC. The selection of software and database for metagenomics sequence analysis impacts the outcome of microbial profiling and pathogen detection. Plos one 2023;18(4):e0284031.
12. Breitwieser FP, Lu J, Salzberg SL. A review of methods and databases for metagenomic classification and assembly. Briefings in bioinformatics 2019;20(4):1125–1136.
13. Kieser S, Brown J, Zdobnov EM, Trajkovski M, McCue LA. ATLAS: a Snakemake workflow for assembly, annotation, and genomic binning of metagenome sequence data. BMC bioinformatics 2020;21:1–8.
14. Tadrent N, Dedeine F, Hervé V. SnakeMAGs: a simple, efficient, flexible and scalable workflow to reconstruct prokaryotic genomes from metagenomes. F1000Research 2022;11.
15. Tamames J, Puente-Sánchez F. SqueezeMeta, a highly portable, fully automatic metagenomic analysis pipeline. Frontiers in microbiology 2019;9:425882.
16. Clarke EL, Taylor LJ, Zhao C, Connell A, Lee JJ, Fett B, et al. Sunbeam: an extensible pipeline for analyzing metagenomic sequencing experiments. Microbiome 2019;7:1–13.
17. Chaumeil PA, Mussig AJ, Hugenholtz P, Parks DH, GTDB-Tk: a toolkit to classify genomes with the Genome Taxonomy Database. Oxford University Press; 2020.
18. Buchfink B, Xie C, Huson DH. Fast and sensitive protein alignment using DIAMOND. Nature methods 2015;12(1):59–60.
19. Kahlke T, Ralph PJ. BASTA–Taxonomic classification of sequences and sequence bins using last common ancestor estimations. Methods in Ecology and Evolution 2019;10(1):100–103.
20. Ondov BD, Treangen TJ, Melsted P, Mallonee AB, Bergman NH, Koren S, et al. Mash: fast genome and metagenome distance estimation using MinHash. Genome biology 2016;17:1–14.
21. Menzel P, Ng KL, Krogh A. Fast and sensitive taxonomic classification for metagenomics with Kaiju. Nature communications 2016;7(1):11257.
22. Callahan BJ, McMurdie PJ, Rosen MJ, Han AW, Johnson AJA, Holmes SP. DADA2: High-resolution sample inference from Illumina amplicon data. Nature methods 2016;13(7):581–583.
23. Sim M, Lee J, Lee D, Kwon D, Kim J. TAMA: improved metagenomic sequence classification through meta-analysis. BMC bioinformatics 2020;21:1–17.
24. Ounit R, Wanamaker S, Close TJ, Lonardi S. CLARK: fast and accurate classification of metagenomic and genomic sequences

using discriminative k-mers. BMC genomics 2015;16(1):1–13.
25. Breitwieser FP, Baker DN, Salzberg SL. KrakenUniq: confident and fast metagenomics classification using unique k-mer counts. Genome Biology 2018;19(1):198. https://doi.org/10.1186/s13059-018-1568-0.
26. Piro VC, Dadi TH, Seiler E, Reinert K, Renard BY. ganon: precise metagenomics classification against large and up-to-date sets of reference sequences. Bioinformatics 2020 Jul;36(Suppl_1):i12–i20.
27. Piro VC, Reinert K. ganon2: up-to-date and scalable metagenomics analysis. NAR Genomics and Bioinformatics 2025 07;7(3):lqaf094. https://doi.org/10.1093/nargab/lqaf094.
28. Song L, Langmead B. Centrifuger: lossless compression of microbial genomes for efficient and accurate metagenomic sequence classification. Genome Biology 2024;25(1):106. https://doi.org/10.1186/s13059-024-03244-4.
29. Ulrich JU, Renard BY. Taxor: Fast and space-efficient taxonomic classification of long reads with hierarchical interleaved XOR filters. bioRxiv 2023;https://www.biorxiv.org/content/early/2023/07/22/2023.07.20.549822.
30. Brown CT, Irber L. sourmash: a library for MinHash sketching of DNA. Journal of Open Source Software 2016;1(5):27. https://doi.org/10.21105/joss.00027.
31. Shang J, Peng C, Liao H, Tang X, Sun Y. PhaBOX: a web server for identifying and characterizing phage contigs in metagenomic data. Bioinformatics Advances 2023;3(1):vbad101.
32. Shang J, Jiang J, Sun Y. Bacteriophage classification for assembled contigs using graph convolutional network. Bioinformatics 2021;37(Supplement_1):i25–i33.
33. Zhou Z, Martin C, Kosmopoulos JC, Anantharaman K. ViWrap: A modular pipeline to identify, bin, classify, and predict viral–host relationships for viruses from metagenomes. Imeta 2023;2(3):e118.
34. Auslander N, Gussow AB, Benler S, Wolf YI, Koonin EV. Seeker: alignment-free identification of bacteriophage genomes by deep learning. Nucleic acids research 2020;48(21):e121–e121.
35. Gałan W, Bąk M, Jakubowska M. Host taxon predictor-a tool for predicting taxon of the host of a newly discovered virus. Scientific reports 2019;9(1):3436.
36. Jiang G, Zhang J, Zhang Y, Yang X, Li T, Wang N, et al. DCiPatho: deep cross-fusion networks for genome scale identification of pathogens. Briefings in Bioinformatics 2023;24(4):bbad194.
37. Altschul SF, Gish W, Miller W, Myers EW, Lipman DJ. Basic local alignment search tool. Journal of molecular biology 1990;215(3):403–410.
38. Gruber-Vodicka HR, Seah BK, Pruesse E. phyloFlash: rapid small-subunit rRNA profiling and targeted assembly from metagenomes. Msystems 2020;5(5):10–1128.
39. Truong DT, Franzosa EA, Tickle TL, Scholz M, Weingart G, Pasolli E, et al. MetaPhlAn2 for enhanced metagenomic taxonomic profiling. Nature methods 2015;12(10):902–903.
40. Lui WW, Leung AW, Leung HC, Xin Y, Teng JL, Woo PC, et al. MegaPath-Nano: Accurate Compositional Analysis and Drug-level Antimicrobial Resistance Detection Software for Oxford Nanopore Long-read Metagenomics. In: 2020 IEEE International Conference on Bioinformatics and Biomedicine (BIBM) IEEE; 2020. p. 329–336.
41. Li H. Minimap2: pairwise alignment for nucleotide sequences. Bioinformatics 2018;34(18):3094–3100.
42. Liang X, Zhang J, Kim Y, Ho J, Liu K, Keenum I, et al. ARGem: a new metagenomics pipeline for antibiotic resistance genes: metadata, analysis, and visualization. Frontiers in Genetics 2023;14:1219297.
43. Prosperi M, Marini S. Karga: Multi-platform toolkit for k-mer-based antibiotic resistance gene analysis of high-throughput sequencing data. In: 2021 IEEE EMBS International Conference on Biomedical and Health Informatics (BHI) IEEE; 2021. p. 1–4.
44. LaPierre N, Alser M, Eskin E, Koslicki D, Mangul S. Metalign: efficient alignment-based metagenomic profiling via containment min hash. Genome Biology 2020;21(1):242. https://doi.org/10.1186/s13059-020-02159-0.
45. Olawoye IB, Frost SD, Happi CT. The Bacteria Genome Pipeline (BAGEP): an automated, scalable workflow for bacteria genomes with Snakemake. PeerJ 2020;8:e10121.
46. Ondov BD, Starrett GJ, Sappington A, Kostic A, Koren S, Buck CB, et al. Mash Screen: high-throughput sequence containment estimation for genome discovery. Genome biology 2019;20:1–13.
47. Baker DN, Langmead B. Dashing: fast and accurate genomic distances with HyperLogLog. Genome biology 2019;20:1–12.
48. Besta M, Kanakagiri R, Mustafa H, Karasikov M, Rätsch G, Hoefler T, et al. Communication-efficient jaccard similarity for high-performance distributed genome comparisons. In: 2020 IEEE International Parallel and Distributed Processing Symposium (IPDPS) IEEE; 2020. p. 1122–1132.
49. Zhao X. BinDash, software for fast genome distance estimation on a typical personal laptop. Bioinformatics 2019;35(4):671–673.
50. Katz LS, Griswold T, Morrison SS, Caravas JA, Zhang S, den Bakker HC, et al. Mashtree: a rapid comparison of whole genome sequence files. Journal of Open Source Software 2019;4(44):10–21105.
51. Broder AZ. On the resemblance and containment of documents. In: Proceedings. Compression and Complexity of SEQUENCES 1997 (Cat. No. 97TB100171) IEEE; 1997. p. 21–29.
52. Pierce NT, Irber L, Reiter T, Brooks P, Brown CT. Large-scale sequence comparisons with sourmash. F1000Research 2019;8:1006.
53. Hernández-Salmerón JE, Moreno-Hagelsieb G. FastANI, Mash and Dashing equally differentiate between Klebsiella species. PeerJ 2022;10:e13784.
54. Hera MR, Liu S, Wei W, Rodriguez JS, Ma C, Koslicki D. Metagenomic functional profiling: to sketch or not to sketch? Bioinformatics 2024;40(Supplement_2):ii165–ii173.
55. Wu W, Li B, Chen L, Gao J, Zhang C. A review for weighted minhash algorithms. IEEE Transactions on Knowledge and Data Engineering 2020;34(6):2553–2573.
56. Sánchez-Reyes A, Fernández-López M. Sketched reference databases for genome-based taxonomy and comparative genomics. Brazilian Journal of Biology 2022;84:e256673.
57. Liu S, Koslicki D. CMash: fast, multi-resolution estimation of k-mer-based Jaccard and containment indices. Bioinformatics 2022 06;38:i28–i35. https://doi.org/10.1093/bioinformatics/btac237.
58. Team MD, Mash Tutorials; 2023. Accessed: 2025-01-10. https://mash.readthedocs.io/en/latest/tutorials.html.
59. Irber L, Brown CT. Lightweight compositional analysis of metagenomes with sourmash gather. Manubot Available at: https://dib-lab github io/2020-paper-sourmash-gather/(Accessed: 16 December 2020) 2020;.
60. Kitts PA, Church DM, Thibaud-Nissen F, Choi J, Hem V, Sapojnikov V, et al. Assembly: a resource for assembled genomes at NCBI. Nucleic acids research 2016;44(D1):D73–D80.
61. Schoch CL, Ciufo S, Domrachev M, Hotton CL, Kannan S, Khovanskaya R, et al. NCBI Taxonomy: a comprehensive update on curation, resources and tools. Database 2020;2020:baaa062.
62. Li H. Minimap and miniasm: fast mapping and de novo assembly for noisy long sequences. Bioinformatics 2016;32(14):2103–2110.
63. Dong J, Liu X, Sadasivan H, Sitaraman S, Narayanasamy S. mm2-gb: GPU accelerated minimap2 for long read dna mapping. In: Proceedings of the 15th ACM International Conference on Bioinformatics, Computational Biology and Health Informatics; 2024. p. 1–9.
64. Langmead B, Wilks C, Antonescu V, Charles R. Scaling read aligners to hundreds of threads on general-purpose processors.

Bioinformatics 2019;35(3):421–432.
65. Rosen G, Garbarine E, Caseiro D, Polikar R, Sokhansanj B. Metagenome Fragment Classification Using N-Mer Frequency Profiles. Advances in bioinformatics 2008;2008(1):205969.
66. Liu B, Gibbons T, Ghodsi M, Treangen T, Pop M. Accurate and fast estimation of taxonomic profiles from metagenomic shotgun sequences. Genome biology 2011;12:1–27.
67. Pruitt KD, Tatusova T, Maglott DR. NCBI reference sequences (RefSeq): a curated non-redundant sequence database of genomes, transcripts and proteins. Nucleic acids research 2007;35(suppl_1):D61–D65.
68. O'Leary NA, Wright MW, Brister JR, Ciufo S, Haddad D, McVeigh R, et al. Reference sequence (RefSeq) database at NCBI: current status, taxonomic expansion, and functional annotation. Nucleic acids research 2016;44(D1):D733–D745.
69. Sayers EW, Beck J, Bolton EE, Bourexis D, Brister JR, Canese K, et al. Database resources of the national center for biotechnology information. Nucleic acids research 2021;49(D1):D10.
70. Jesus TF, Ribeiro-Gonçalves B, Silva DN, Bortolaia V, Ramirez M, Carriço JA. Plasmid ATLAS: plasmid visual analytics and identification in high-throughput sequencing data. Nucleic acids research 2019;47(D1):D188–D194.
71. Blanco-Míguez A, Beghini F, Cumbo F, McIver LJ, Thompson KN, Zolfo M, et al. Extending and improving metagenomic taxonomic profiling with uncharacterized species using MetaPhlAn 4. Nature Biotechnology 2023;41(11):1633–1644. https://doi.org/10.1038/s41587-023-01688-w.
72. Burki F, Roger AJ, Brown MW, Simpson AG. The new tree of eukaryotes. Trends in ecology & evolution 2020;35(1):43–55.
73. Simmonds P, Adams MJ, Benkő M, Breitbart M, Brister JR, Carstens EB, et al. Virus taxonomy in the age of metagenomics. Nature Reviews Microbiology 2017;15(3):161–168.
74. Harris HM, Hill C. A place for viruses on the tree of life. Frontiers in Microbiology 2021;11:604048.

Manuscript Click here to access/download;Manuscript;main.pdf

GigaScience, 2023, 1–16

doi: xx.xxxx/xxxx
Manuscript in Preparation
Paper

PAPER

HYMET: A Hybrid Metagenomic Pipeline for Accurate and Efficient Taxonomic Classification

Inês Martins[1,*], Jorge Miguel Silva[1,*] and João Rafael Almeida[1]

[1]IEETA/DETI, LASI, University of Aveiro, Aveiro, Portugal

[*]inesbrancomartins@ua.pt; jorge.miguel.ferreira.silva@ua.pt

Abstract

Background: Reliable taxonomic classification of metagenomic sequences remains constrained by high mutation rates, fragmented assemblies, and large heterogeneous reference databases. HYMET (Hybrid Metagenomic Tool) was developed to overcome these challenges through a two-stage hybrid design combining adaptive Mash-based screening with Minimap2 alignment and a coverage-weighted Lowest Common Ancestor (LCA) classifier. Its sample-adaptive thresholds and on-the-fly reference construction enable efficient, domain-agnostic classification while maintaining accuracy across divergent genomes.
Results: Across seven CAMI assemblies in contig mode, HYMET achieved a mean F1 of 83.89%, with genus-level F1 of 76.75% and species-level F1 of 60.18%, while averaging 115.93 s runtime and 6.24 GB of peak memory. Performance remained stable under mutation rates up to 30% for most domains ($F1 \geq 0.8$), with viral sequences showing the expected decline ($F1 \approx 0.5$ at 30%). Read and contig inputs produced nearly identical results when sharing reference caches, and real-world datasets confirmed robustness with the human gut metagenome reproduced typical anaerobic profiles, while in the ZymoBIOMICS mock community HYMET recovered all bacterial members.
Conclusions: HYMET achieves a practical balance of accuracy, efficiency, and scalability for metagenomic classification. Its adaptive candidate selection, alignment-anchored taxonomy, and reproducible reference caching collectively enhance performance across domains. HYMET source code is fully available at https://github.com/ieeta-pt/HYMET.

Key words: Metagenomics, taxonomic classification, *k*-mer screening, alignment-based methods, computational efficiency, mutation resistance, hybrid pipeline.

Compiled on: November 1, 2025.
Draft manuscript prepared by the author.

Key Points

HYMET integrates Mash-based adaptive screening and Minimap2 alignment with a weighted Lowest Common Ancestor (LCA) classification, dynamically tailoring reference databases to each metagenomic dataset for improved efficiency and accuracy.

Evaluated across diverse CAMI benchmarks, HYMET achieves robust F1-scores (mean across ranks: 83.89%, genus: 76.75%, species: 60.18%) while maintaining rapid runtimes (average 116 s) and moderate memory usage (peak 6.2 GB).

HYMET demonstrates strong resilience to genetic mutations (up to 30%), maintaining high accuracy for most biological domains ($F1 \geq 0.8$), though viral classification accuracy decreases at higher mutation rates ($F1 \approx 0.5$ at 30%).

Real-world evaluations confirm HYMET's practical applicability: gut microbiome profiles closely matched expected anaerobic bacterial taxa, while ZymoBIOMICS mock-community results accurately identified bacterial constituents, despite missing yeast species under current parameters.

Resource-efficient design enables reproducible and scalable runs, the tool requires only 2.82 GB for installation, dynamically manages reference cache footprints (10–50 GB typical), and offers full reproducibility via Bioconda, container images, and versioned databases deposited on Zenodo.

1 Introduction

A significant challenge in metagenomics is the development of accurate methods for the taxonomic classification of organisms within a sample [1, 2]. Despite the creation of numerous general-purpose and specialized metagenomic tools, several significant issues persist. Particularly, computational demands pose a major constraint, as tools often require substantial memory and processing power, leading to impractical execution times for large datasets [3, 4, 1]. The sheer volume of metagenomic datasets demands highly efficient algorithms that can operate within reasonable requirements of compute power, which is particularly problematic when dealing with millions of sequencing reads [5, 6, 7, 8]. Furthermore, taxonomic assignment remains a critical challenge in metagenomic analysis, especially at lower taxonomic levels [8]. This issue is exacerbated by the limitations of reference databases, which often exhibit significant sampling bias towards well-studied organisms, while underrepresenting species that are difficult to culture in laboratory settings [3, 1]. This discrepancy results in high rates of unclassified or misclassified reads, especially in complex environmental samples [9, 2, 10]. Furthermore, the lack of standardized benchmarking protocols and datasets, hinder objective comparisons of tool performance, as researchers frequently test tools on non-uniform datasets with inconsistent evaluation metrics [10, 11, 12]. Addressing these issues is crucial for improving our understanding of complex microbial communities and developing efficient, user-friendly software solutions to analyze the enormous amounts of data generated by metagenomic research [3, 1]. These collective challenges directly motivate our core research question:

> *How can a next-generation metagenomic classification tool be designed and implemented to accurately identify taxa across all domains while maintaining high performance and efficiency?*

HYMET (Hybrid Metagenomic Tool) was conceived in response to the recurrent bottlenecks observed when processing large and diverse metagenomic datasets, where existing profilers either failed to capture divergent taxa or consumed excessive resources. Its design draws inspiration from practical experience with real microbial communities, where rapid screening and selective reference construction often proved more effective than relying on static, monolithic databases. HYMET follows this principle by integrating adaptive reference selection with precise alignment and a weighted taxonomic resolver. This hybrid, sample-aware approach enables the tool to dynamically tailor its search space to each dataset, thereby reducing memory usage and execution time while preserving classification accuracy across domains.

Specifically, HYMET introduces three main innovations. First, an adaptive Mash Screen step selects candidate references on-the-fly, ensuring that downstream analysis focuses only on the most relevant genomes under a fixed resource budget. Second, the selected references are combined into a temporary, sample-specific database that supports accurate alignment even in the presence of mutations or incomplete references. Third, a coverage-weighted lowest common ancestor (LCA) algorithm integrates the breadth and depth of alignment evidence to improve taxonomic consistency, particularly at lower ranks. Together, these components allow HYMET to outperform static index-based approaches in both speed and precision, especially for complex or previously unseen samples.

2 Background

In recent years, we have witnessed remarkable progress in metagenomics, particularly in the development of computational tools for taxonomic classification and functional analysis [2, 1, 3]. A dominant trend in current methodologies is the integration of established classification techniques into end-to-end pipelines, which streamline the entire analytical workflow, from raw sequencing data to biologically interpretable results [13]. Currently, the state-of-the-art landscape is populated by a rich ecosystem of interconnected tools, each offering unique capabilities and complementary approaches that collectively advance the field's analytical power. Among these, SnakeMAGs [14] stands out for its specialized focus on reconstructing prokaryotic genomes from Illumina sequencing reads, while SqueezeMeta [15] offers a fully automated and comprehensive solution for metagenomic data analysis [16, 13]. The first tool uses the Genome Taxonomy Database (GTDB) toolkit [17] for taxonomic assignment, leveraging conserved marker genes for analysis. On the other hand, SqueezeMeta uses DIAMOND [18] for alignment and the Lowest Common Ancestor (LCA) algorithm for taxonomic assignment [15].

Complementing these general-purpose pipelines, several lightweight tools have emerged to address specific needs in taxonomic assignment. The Basic Sequence Taxonomy Annotator (BASTA) [19] also employs the LCA algorithm for efficient sequence classification, while the Critical Assessment of Metagenome Interpretation Taxonomy (CAMITAX) [4] improves accuracy through the integration of multiple classification strategies for microbial genome assignment, including genome distance-based classification using Mash [20], Centrifuge [1] and Kaiju [21], that determines the interval-union LCA of gene-level assignments and 16S rRNA gene-based classification employing a naive Bayesian classifier method using Dada2 [22]. For more robust taxonomic profiling, the Taxonomy Analysis by Multiple Assignment (TAMA) tool [23] combines consensus classifications from established classifiers, including Kraken [8], CLARK [24], and Centrifuge, leveraging their complementary strengths. In addition to these workflows, widely used read-level classifiers include KrakenUniq, Ganon (and ganon2), Centrifuge/Centrifuger, Taxor, and compositional MinHash methods such as sourmash gather; these are often embedded within workflows (for example, CAMITAX integrates Centrifuge/Kaiju and TAMA combines Kraken, CLARK, and Centrifuge), but we do not benchmark them directly here [25, 26, 27, 28, 29, 30].

The field has also seen the development of specialized tools that target specific metagenomic applications. Viral genomics is particularly well served by PhaBOX [31, 32] for viral contig characterization and ViWrap [33] for prediction of viral-host relationship, both providing valuable information on viral diversity and ecological interactions [34, 35, 36]. The first tool, PhaBOX, developed by Shang et al., combines gene prediction and alignment (DIAMOND) with taxonomic classification by semi-supervised learning method (PhaGCN [32]), based on sequence similarities and cluster sharing networks, and final assignments using the LCA. ViWrap, on the other hand, uses machine learning and sequence similarity searches to identify viral sequences and BLAST [37] to identify best hits against databases for taxonomic annotation and host prediction. For the analysis of the microbial community, PhyloFlash [38], developed by Gruber-Vodicka et al., offers unique capabilities through its small subunit ribosomal RNA (SSU rRNA)-based approach, enabling both metagenomic profiling and high-resolution phylogenetic studies [39]. In the critical area of antimicrobial resistance surveillance, MegaPath-Nano [40] has emerged as an important tool for the comprehensive detection of resistance genes, directly supporting public health monitoring efforts; it couples hash-based k-mer mapping with Minimap2's seed-chain-extend *local* alignment model [41], thereby covering broad and potentially divergent sequence segments [42, 43]. Related alignment-based profilers adopt a two-stage design. For example, Metalign [44] first applies CMash to pre-filter the reference by containment and then aligns reads with Minimap2 to produce the profile.

Despite this technological progress, significant challenges impede the broader implementation of metagenomic tools in clinical and research settings. Implementation barriers represent a primary obstacle, with inadequate documentation and complex installation procedures frequently compromising tool accessibility and user

adoption [45]. Computational constraints further limit practical application, as excessive memory and storage requirements hinder scalability. This is exemplified by SqueezeMeta, which stages more than 500 GB of on-disk reference data and exhibits prohibitively long processing times [15], and BASTA, whose dependence on BLAST-based alignments creates computational bottlenecks that render it inefficient for time-sensitive analyses [19, 37]. A fundamental limitation stems from reference database dependencies rather than inherent tool restrictions. For instance, TAMA demonstrates robust classification capabilities in principle, but its default bacterial reference database necessarily limits its taxonomic scope to bacterial identification while requiring hundreds of gigabytes of disk space for its bundled indices [23]. Similarly, independent evaluations of MegaPath-Nano confirm its strong performance in the detection of prokaryotic antimicrobial resistance, but note a reduced sensitivity when analyzing higher eukaryotes [40, 42]. This pattern of taxonomic bias is further evidenced in specialized tools such as PhaBOX and ViWrap, which, while excelling in virome analysis, lack versatility for broader metagenomic applications [33, 31]. PhyloFlash's reliance on small subunit rRNA analysis makes it fundamentally unsuitable for viral identification, as viruses lack ribosomal RNA genes [38].

3 Materials and Methods

3.1 HYMET workflow overview

HYMET, illustrated in Figure 1, is driven by a unified Python CLI (`hymet` from Bioconda or `bin/hymet` in a source checkout) that orchestrates Mash (k-mer screening), Minimap2 (alignment), and the weighted-LCA classifier. For reproducible installs we provide container images (Docker and Apptainer/Singularity) and a Bioconda package (Conda/Mamba). Legacy Perl components are bundled for reproducibility but are not the primary entry point. HYMET is designed for shotgun metagenomes in contig or genome form. It can classify 16S/SSU rRNA sequences when they occur in assembled contigs or shotgun reads, but targeted 16S rRNA amplicon libraries are out of scope and are better analyzed with rRNA-centric profilers (e.g., phyloFlash [38]). Installation and configuration details are provided in Supplementary Sections 1–4.

Alignment-Free k-mer Screening

The initial phase of HYMET utilizes Mash Screen [20] for rapid k-mer-based screening against pre-computed MinHash reference sketch databases [46, 47, 48, 49, 50]. Mash employs containment scores, as defined in Equation 1, to assess the proportion of a reference genome present in a query sequence [51, 52, 53]. The containment index $c_k(a, b)$ is estimated as:

$$c_k(a, b) \approx \frac{|S(A) \cap \pi(B)|}{|S(A)|}, \quad (1)$$

where $S(A)$ is the sketch of the reference genome A, and $\pi(B)$ represents the k-mers of the query sequence B. The containment index ranges from 0.0 to 1.0, with values closer to 1.0 indicating a higher proportion of k-mers from the reference genome present in the query. This metric is crucial for tasks such as contamination screening, reference genome selection, and the discovery of novel genomes, as it provides a rapid and unbiased estimate of sequence representation [46, 47, 51, 49, 54]. In this work, the screening process was optimized for computational efficiency by enabling parallel processing and applying a stringent 90% similarity threshold to retain only high-confidence matches, filtering out low-quality alignments. This containment-based approach prioritizes likely taxonomic candidates, reducing the search space and computational load for subsequent alignment-based stages [20, 46, 52]. As

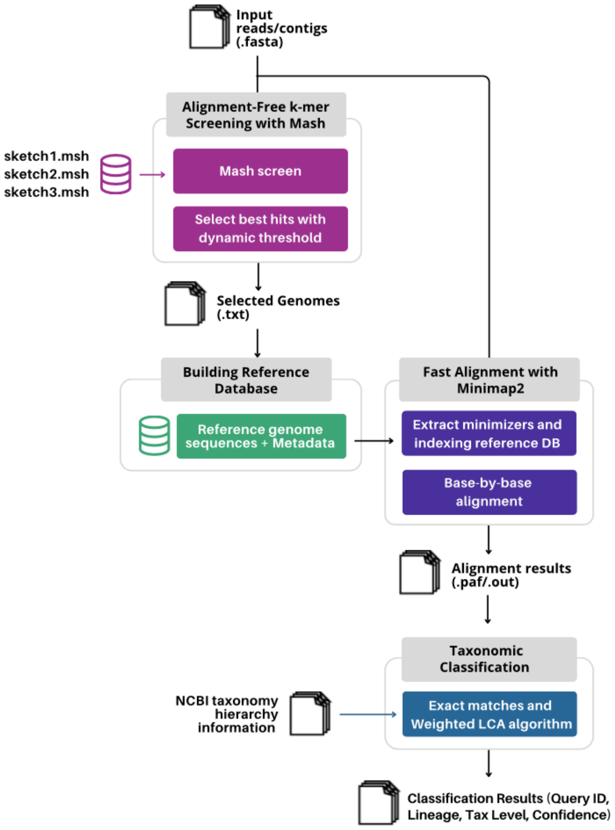

Figure 1. Overview of HYMET architecture.

previously mentioned, to enable this screening, Mash relies on sketched databases, which are compact representations of genomic sequences. These databases are built using consistent k-mer hashing with MurmurHash3 [20, 55, 47, 56], which allows efficient comparison of query sequences against large collections of reference genomes.

HYMET requires fast per-reference containment ranking on unassembled reads under a fixed memory budget. Mash Screen provides fixed-size MinHash sketches, direct read-set → reference containment estimates, and per-hit p-values, which integrate cleanly with our adaptive cut-off and candidate budgeting. In contrast, sourmash uses scaled FracMinHash with an iterative gather (set-cover) procedure aimed at mixture decomposition rather than per-reference ranking, and CMash (as in Metalign [44]) focuses on multi-k containment estimation, which we do not require here. Consequently, HYMET applies a dynamic, sample-adaptive containment threshold that by default targets about 3.25 candidates per input sequence (rounded; minimum of five) and enforces a floor threshold of 0.70, reducing the search space while preserving recall. The pre-filter is modular and could be replaced by sourmash or CMash without altering downstream alignment; we chose Mash for its simplicity, stable memory, and native significance testing [30, 57, 44].

Sketch and k-mer Size. The construction of these sketch databases involves two key parameters: the k-mer size (k) and the sketch size (s). The choice of k-mer size is essential as it balances sensitivity and specificity. Smaller k-mers increase sensitivity for divergent genomes but may lead to random collisions, while larger k-mers reduce collisions but may miss subtle variations [58, 20, 47, 51, 50]. The optimal k-mer size is calculated as:

$$k = \log_{|\Sigma|}\left(\frac{n(1-q)}{q}\right), \quad (2)$$

where $|\Sigma|$ is the alphabet size (4 for nucleotides), n is the genome size, and q is the desired probability of observing a random k-mer. For example, smaller genomes (e.g. viruses) and highly variable taxa require smaller k-mer sizes (e.g. k-mer=15) to ensure specificity, while larger genomes (e.g. vertebrates) benefit from moderate k-mer sizes (e.g. k-mer=21) to balance sensitivity and computational efficiency [53, 58].The sketch size, which refers to the number of unique min-hashes retained for genomic sequence representation, also plays a critical role in determining the accuracy of distance and containment estimates [58, 20, 50, 52]. The error associated with containment estimation for a given sketch size, s, is proportional to:

$$\text{Error} \approx \sqrt{\frac{1}{s}}, \tag{3}$$

indicating that larger sketch sizes improve precision, but at the expense of greater computational resources [58]. For instance, smaller or highly fragmented genomes typically require larger sketch sizes to ensure sufficient genomic information is captured, while larger or less fragmented genomes can achieve accurate containment estimates with smaller sketch sizes. This adaptive approach is supported by empirical evidence, with studies demonstrating that a sketch size of $s = 1000$ is generally adequate for obtaining precise similarity estimates in well-assembled genomes [20]. In fact, B. D. Ondov *et al.* established $s = 1000$ and k=21 as the default parameters in Mash, as they provide precise similarity estimates for well-assembled genomes [20]. However, for more divergent genomes, increasing the sketch size (e.g. $s = 5000$) can improve accuracy by capturing a more representative subset of genomic content [58, 50, 59, 53].

Reference Sketched Databases. Following these design principles, we implemented a comprehensive database strategy combining both established public resources and a custom-built collection:

- **RefSeq Nucleotide Release 228:** Contains sketches of 162 138 organisms from RefSeq release 228, compressed using $k = 21$ and $s = 1000$, reducing the original 1.2 TB dataset to a compact representation [58].
- **GTDB r202 Assembly Set and NCBI Complete Genomes Database:** Combines 89 675 genomes from GTDB r202 and NCBI RefSeq (viruses, fungi, and bacteria/archaea), compressed with $k = 21$ and $s = 1000$ [56].
- **Custom Reference Database:** Enhances representation of underrepresented taxa by including 19 505 up-to-date genomes from NCBI RefSeq. For smaller genomes (e.g., archaea, fungi, protozoa, viruses), sketches were generated with $k = 15$ and $s = 5000$, while larger genomes (e.g., vertebrates, plants, invertebrates) used the default parameters ($k = 21$, $s = 1000$) [50].

These databases were grouped on the basis of shared seed values and parameters to optimize the screening efficiency. Table 1 summarizes their characteristics. All databases are publicly available through our project repository. For reproduction, detailed instructions are provided in Supplementary Material Section 2, Subsection "Reproducing Sketched Databases".

Modular Reference Database Download

Candidate selection with dynamic threshold. After running Mash Screen, the output can be extensive, potentially including a large number of candidate genomes with varying degrees of similarity to the query sequences. Downloading and analyzing this entire list would be computationally inefficient and could introduce noise into subsequent analyses. On the other hand, setting an arbitrarily high static threshold might exclude important reference genomes, leading to incomplete coverage of the query sequences. To address these challenges, HYMET introduces a dynamic Mash-Screen thresholding mechanism to identify the most relevant candidate genomes and to create a targeted, input-specific database. The algorithm iteratively lowers the containment threshold in 0.02 steps until a minimum candidate count is reached (about 3.25 per input sequence, rounded; minimum five), or until a floor of 0.70 is reached.

Table 1. Reference sketched databases. Different seed values reflect the provenance of each sketch set. RefSeq88 sketch from Mash with seed 0, GTDB and custom sketches generated locally using default seed 42.

Sketch	Content	Sketch Parameters	Sketch Size	Seed Value
sketch1.msh	RefSeq nucleotide release 88	k=21, s=1000	1.2 GB	0
sketch2.msh	GTDB r202 Assembly Set, NCBI Complete Genomes Database + Custom databases (vertebrates, plants, invertebrates)	k=21, s=1000	883.25 MB	42
sketch3.msh	Custom databases (fungi, protozoa, archaea, virus)	k=15, s=5000	327.93 MB	42

Genome Retrieval. Following the selection of candidate genomes, their format was analyzed to enable efficient mapping and retrieval. These genomes were identified using RefSeq (GCF) and Genbank Assembly Genomes (GCA) accession numbers, unique identifiers assigned by NCBI. Thus, the NCBI Assembly database [60] was selected as the primary resource for constructing the reference database [10, 61]. To optimize the process, summary files from the NCBI Assembly database were downloaded, providing efficient access to metadata. A custom script was developed to map candidate genomes to these files using accession numbers. This script extracted the base accession number (e.g. "000169215") to ensure compatibility between different assembly versions (e.g.: GCF_000169215.1, GCF_000169215.2), preventing retrieval failures due to version updates [60].

During the initial mutation study (conducted with the systematic-review harness), genomes were retrieved from the NCBI FTP service and decompressed locally. In all subsequent benchmarks and case studies in this manuscript, genomes were retrieved over HTTPS from NCBI and decompressed locally. The downloader uses bounded retries with exponential backoff and records failures in the run logs. To enhance efficiency, the script employed ThreadPoolExecutor for parallel downloads, allowing up to 64 concurrent threads. The taxonomy IDs (TaxID) of the assembly files were stored alongside the accession numbers and sequence identifiers, creating a comprehensive reference linking each genome to its taxonomic and sequence-level information [60, 61].

HYMET is designed to operate efficiently in network-constrained or offline environments. Users can supply a local directory containing mirrored NCBI assembly summaries to enable species-level candidate deduplication without requiring internet connectivity. Reference data are cached locally, and users have the option to preload these caches with curated FASTA files along with their corresponding sequence-to-TaxID mappings. Indices are automatically updated during subsequent analyses, ensuring reproducibility and consistency even in offline scenarios. Additional details can be found in "Reference retrieval policy and fallbacks" of the Supplementary Material.

Fast Alignment

In the second processing stage, HYMET employs Minimap2 for efficient and precise sequence alignment. This choice was motivated by Minimap2's adaptive scoring system and its seed-chain-

extend *local* alignment strategy, which enables accurate mapping even with highly divergent sequences or incomplete reads, making the pipeline particularly resilient to common metagenomic challenges such as mutation-rich or fragmented samples [40, 41]. The pipeline uses minimizers to index reference sequences, enabling the rapid identification of alignment regions [62]. For contig/genome inputs we use the `-x asm10` preset, optimized for genome-to-genome alignment (approximately 10% divergence, or 90% identity) [63, 41]; for read inputs we use Minimap2's short-read preset `-x sr`. The results are saved in a PAF file, providing essential alignment details such as sequence IDs, lengths, positions, and mapping quality [41, 64].

Taxonomic Assignment

HYMET uses a hybrid taxonomic assignment strategy, combining the LCA algorithm with a weighted approach based on alignment coverage [23, 8, 24]. For exact matches, the reference's taxonomic lineage is directly assigned with a confidence score of 1.0. On the other hand, for non-exact matches, the weights for each TaxID are calculated according to:

$$\text{Weight} = \text{Coverage} \times \text{Abundance}, \tag{4}$$

where *Coverage* is the proportion of the query aligned with the reference and *Abundance* is the reference's frequency in the dataset. HYMET then computes a weighted consensus lineage across ranks and reports a single representative TaxID (the taxon with the highest cumulative weight across the supporting alignments). The confidence score is derived as the product of the per-rank consensus fractions.

$$\text{Confidence Score} = \prod_{i=1}^{n} \text{Confidence at Rank}_i, \tag{5}$$

where n is the number of ranks. This ensures higher consistency across ranks results in higher confidence scores. The final output includes the query identifier, a taxonomic lineage (kingdom to strain), the most specific rank, the representative NCBI TaxID, and a confidence score (0.0 to 1.0), reflecting the reliability of the classification [24, 65, 66]. The tab-delimited file `classified_sequences.tsv` contains the columns: Query, Lineage, Taxonomic Level, TaxID, Confidence.

All analyses, including tool evaluation, development, and validation, were conducted on a high-performance Linux-based virtual machine with 2 TB storage and 250 GB RAM. HYMET's performance was assessed using precision and F1 score metrics for classifying organisms across the three domains of life, considering taxonomic levels (kingdom to species) and mutation rates (0% to 30%). The analysis also examined the relationship between F1 scores, execution time, and resource usage (CPU and memory), ensuring a complete evaluation of precision and efficiency. The same methodology was applied to the other current state-of-the-art tools described in Section 1 to ensure a consistent comparison. Benchmarking was performed by comparing HYMET with general-purpose cross-domain workflows that produce per-read taxonomic assignments. Component-level short-read classifiers (e.g., KrakenUniq, Ganon, Centrifuge/Centrifuger) and compositisonal search methods (e.g., sourmash gather) were out of scope for head-to-head benchmarking; their behavior is represented through the workflows that include them. Detailed instructions for reproducing the benchmarking of these tools are provided in Supplementary Section 5.

Table 2. Composition of the test and validation dataset

Domain/Group	Number of GCFs	Size (GB)
Viruses	1 498	0.05
Other Vertebrates	43	2.83
Vertebrate Mammals	23	2.29
Protozoa	12	0.03
Plants	19	1.02
Invertebrates	43	1.14
Fungi	63	0.15
Bacteria	24 271	7.23
Archaea	231	0.05
**Total**	**26 203**	**14.76**

3.2 Test and Validation Dataset

The test dataset was derived from the NCBI RefSeq Assembly database (last modified: 13 October 2024), chosen for its curated and validated sequences [67, 10, 68]. Assembly summary files for all biological domains and viruses were downloaded and 10% of the entries were randomly selected based on GCF accession numbers. For each GCF, 10% of its genome sequences were further sampled to ensure proportional representation and mimic the fragmentation of metagenomic data [69]. This approach resulted in a diverse and representative dataset, as detailed in Table 2. For dataset replication, complete instructions and scripts are provided in the Supplementary Material Section 3, Subsection "Replicating the Benchmark Dataset".

3.3 CAMI Benchmarking Design

To assess the performance of HYMET alongside other baseline metagenomic classification tools, we performed standardized benchmarking using the CAMI (Critical Assessment of Metagenome Interpretation) datasets. Specifically, we selected seven publicly available contig assemblies representing various levels of complexity and ecological contexts. These include the Low, Medium, and High Complexity communities from CAMI I, the Mouse Gut, Marine, and Strain Madness panels from CAMI II, and the CAMI reference sample_0 (details are provided in Table 3).

We executed all tools strictly in contig mode to maintain consistent conditions across evaluations. To ensure fair comparisons, our benchmarking pipeline standardized input staging, fixed the number of computational threads, and maintained consistent input/output management. Additionally, it captured computational metrics such as wall-clock and CPU time, as well as peak memory usage.

The evaluation methodology adhered closely to established CAMI conventions. We reported metrics across taxonomic ranks from superkingdom down to species level, including both profile-based distances (L1 total variation and Bray–Curtis distance) and precision, recall, and F1-score for presence/absence classification (with a minimum abundance threshold of 0.1%). Contig-level accuracy was determined by directly comparing predicted taxonomic IDs (TaxIDs) with the CAMI-provided ground truth. Results were summarized individually for each sample and aggregated by taxonomic rank across all seven assemblies.

Tools included in the benchmarking process were HYMET, Kraken 2, Centrifuge, Ganon 2, TAMA, SqueezeMeta, ViWrap, MegaPath-Nano, BASTA, CAMITAX, MetaPhlAn 4, sourmash gather, phyloFlash, SnakeMAGs, and PhaBOX. Each tool was configured using its recommended database or default settings suitable for contig-based inputs. The specific software versions and database sources utilized are fully documented in the supplementary materials.

Table 3. CAMI benchmark samples and inputs used in the benchmark. All evaluations are contig based and scored against CAMI truth profiles and contig maps.

Sample ID	CAMI panel	Complexity/context	Input	Truth assets
cami_i_lc	CAMI I	Low complexity community	contigs	profile + contig truth
cami_i_mc	CAMI I	Medium complexity community	contigs	profile + contig truth
cami_i_hc	CAMI I	High complexity community	contigs	profile + contig truth
cami_ii_mousegut	CAMI II	Mouse gut metagenome	contigs	profile + contig truth
cami_ii_marine	CAMI II	Marine metagenome	contigs	profile + contig truth
cami_ii_strainmadness	CAMI II	Strain Madness (strain variation)	contigs	profile + contig truth
cami_sample_0	CAMI reference	Reference assembly	contigs	profile + contig truth

3.4 HYMET Contig vs Read Evaluation

To assess how input modality affects HYMET's performance, each CAMI assembly listed in Table 3 was analyzed under two conditions: first, using assembled contigs, and second, using synthetic reads generated from these same assemblies. Both modes followed the same general workflow—initial sketch-based screening for candidate references, construction or reuse of reference caches, sequence alignment, and weighted lowest-common-ancestor taxonomic assignment. Conditions such as computational threading, file handling, and benchmarking instrumentation remained consistent between modes. The primary distinction between the two analyses involved the alignment parameters and input handling. For the contig mode, HYMET employed alignment settings optimized specifically for longer, genome-to-genome comparisons. In contrast, the read mode processed single-end reads with alignment parameters tailored for shorter, fragmented sequences. Importantly, steps like candidate selection, reference caching, and taxonomic classification remained unchanged, ensuring comparability across modalities. For each assembly and input modality, the benchmarking framework calculated CAMI performance metrics at all taxonomic ranks, including L1 total variation, Bray–Curtis dissimilarity, precision, recall, and F1 scores. Additionally, wall-clock time and peak memory usage were recorded. Results were first summarized individually per sample, then aggregated as averages across all seven assemblies to enable a balanced comparison of performance between contig and read inputs.

3.5 Case-study Design: Gut and Zymo

To complement the CAMI benchmarks with real samples, we conducted two case studies that reflect common metagenomic contexts: a human gut metagenome assembly from MGnify and the ZymoBIOMICS mock community assembly curated by the Loman Lab. Both were processed in contig mode using the same workflow in Section 3.1, with a fixed number of threads, shared taxonomy inputs, and identical reference caching policy. This ensured that any differences in outcomes arise from sample biology rather than methodological drift.

The Zymo mock community provides a laboratory-defined composition and a canonical set of reference genomes. Ground truth was established at two levels: (i) contig-level labels by mapping assembled contigs to the curated Zymo reference panel and assigning each contig to a species TaxID; and (ii) a CAMI-style abundance profile used to compute rank-wise precision, recall, F1, and abundance distances (L1 total variation and Bray–Curtis). The human gut assembly does not have a strict gold standard; evaluation therefore emphasized plausibility of dominant taxa and concordance with public annotations, together with resource measurements.

For both samples, we recorded wall-clock time and peak resident memory for the complete pipeline. Table 4 summarizes the inputs and available truth assets.

In addition to the main Zymo case study, we conducted a targeted reference-ablation protocol to probe robustness to incomplete databases—an increasingly common scenario as public catalogues lag behind newly observed strains. Using the same reference panel employed in the case analysis, we progressively withheld the indexed sequences for each of the ten constituent organisms (TaxIDs 562, 28901, 1423, 1639, 1351, 1280, 1613, 287, 4932, 5207) at 0%, 25%, 50%, 75%, and 100% removal levels. After each removal step we rebuilt the reference index and repeated the full HYMET classification pipeline without changing any parameters. For every level we captured the distribution of contig assignments by taxonomic rank (species or strain, genus, family, and higher) alongside the standard rank-wise profile metrics (precision, recall, F1, L1 total variation, Bray–Curtis) and runtime measurements. This procedure isolates the impact of reference incompleteness while keeping the sample and analytical settings fixed, thereby reflecting realistic deployments in which key genomes are missing or outdated.

Table 4. Case-study samples and inputs. The Zymo mock community is evaluated against curated contig-level labels and a CAMI-style profile; the gut assembly is assessed descriptively in the absence of a ground-truth profile.

Sample ID	Source	Context	Input	Truth assets
zymo_mc	ZymoBIOMICS mock community (Loman Lab assembly)	Even bacterial/fungal mix	contigs	contig labels + CAMI profile
gut_case	MGnify MGYS00006849; SRS9791096; SRR15489027; ERZ24911249; MGYA00794604	Human stool metagenome	contigs	none (top-taxa comparison)

4 Results

Benchmark Scope and Replicates

We report results over seven CAMI assemblies (Table 3): CAMI I low/medium/high complexity; CAMI II mouse gut, marine, and strain madness; and the CAMI reference sample_0. Each assembly was processed once per tool under fixed threads, yielding one run per sample per tool; no technical replicates were used. Rank-wise summaries reflect means across the seven assemblies. The manifest that enumerates these samples is versioned in the repository.

For HYMET's modality comparison, the same seven assemblies were analysed twice: (i) contigs as provided; and (ii) deterministic synthetic reads created by windowing contigs into 250 bp slices with a 125 bp minimum tail. Aside from input handling and Minimap2 presets, all pipeline stages (candidate selection, cache construction, alignment, weighted-LCA) and resource controls were identical; again, one run per sample per mode (no replicates).

The mutation-sweep experiment spans nine higher-level groups (Viruses, Archaea, Bacteria, Fungi, Plants, Protozoa, Invertebrates, Vertebrate Mammals, Other Vertebrates). For each group, we generated one mutated contig set per rate between 0% and 30% using reproducible seeds (substitutions with short indels), then computed precision/recall/F1 by rank from contig-level truth. No technical replicates were used in this sweep.

Real-data evaluations comprise two case studies (MGnify gut assembly and ZymoBIOMICS mock community), each run once in contig mode using the same workflow as above (Section 3.1).

Performance Evaluation

All quantitative evaluations in this revision are contig based. HYMET also accepts read inputs via the unified CLI (Minimap2 `sr` preset), but a dedicated raw-read benchmark is deferred to a follow-up to avoid mixing modalities. Against contemporary baselines, HYMET maintains high F1 across ranks and taxonomic groups; many competitors lose precision and recall at lower ranks, whereas HYMET preserves balanced performance into genus and species, particularly for microbes and small eukaryotes. Viruses remain the most sensitive to divergence, with modest drops at intermediate ranks, but species-level accuracy remains competitive.

Mutation Resilience

HYMET's performance proves exceptionally stable under varying mutation rates (0–30%), outperforming all benchmarked tools in both accuracy and consistency (Figure 3). Viral classification shows a progressive decline at extreme mutations (F1 scores approximately 0.5 at thirty percent), while archaea, invertebrates and fungi maintain F1 scores over 0.9. Other groups show only minor, non-significant reductions, staying above 0.8 (Figure 4). This contrasts with competing tools, where their scores decline as the mutation rate increases (Supplementary Figures 1-2).

Computational Efficiency

We report resource usage for two CAMI suites that share the same assemblies but differ in the candidate-reference budget. In the canonical multi-tool suite (tight candidate cap with species deduplication), HYMET builds smaller caches and averages approximately 116 seconds wall-clock and 6.2 GB peak resident memory across the seven assemblies (Figure 7; Table 6). Baselines in this suite illustrate distinct speed–memory–accuracy trade-offs (e.g., MetaPhlAn 4: 147 s, 18.8 GB; Kraken 2: 40 s, 11.0 GB; MegaPath-Nano: 24 s, 10.2 GB; TAMA: 56 s, 16.9 GB).

To isolate input-modality effects, the HYMET-only reads-vs-contigs suite deliberately expands the candidate budget so both modes reuse the same, larger cache. Under this regime, HYMET's contig runs average 361 seconds and 17.37 GB peak memory, while the synthetic-read runs average 334 seconds and 17.36 GB (Figure 9; Table 7). The near-identical memory stems from the shared cache; the modest runtime delta arises primarily from Minimap2 presets (`asm10` for contigs vs `sr` for reads), not from differences in the search space.

The case studies align with the expanded-budget envelope. The MGnify gut assembly and the ZymoBIOMICS mock community each completed in 4–5 minutes with 17.4 GB peak memory on the reference machine (Table 9), consistent with the reads-vs-contigs suite and illustrating predictable behavior on real assemblies. In practice, users can choose between these profiles by selecting a tighter candidate cap (minutes-scale, lower memory) or an expanded budget (longer runs with larger caches that can improve

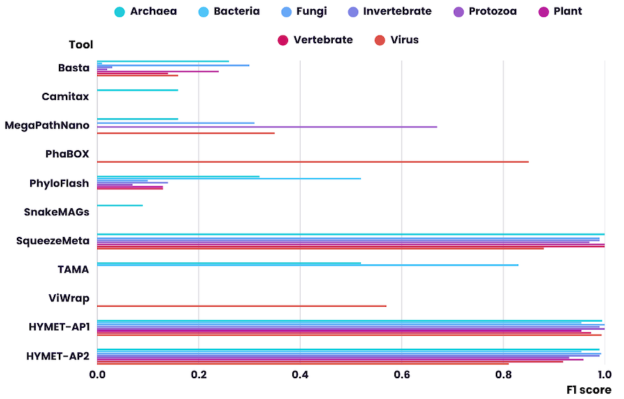

Figure 2. F1 scores achieved by various taxonomic classification tools, including both state-of-the-art tools and HYMET, across different taxonomic groups. The x-axis represents the F1 scores, ranging from 0 to 1, while the y-axis lists the evaluated tools. Each bar is color-coded to indicate the corresponding taxonomic group. This data is based on analysis with 0% mutation rate.

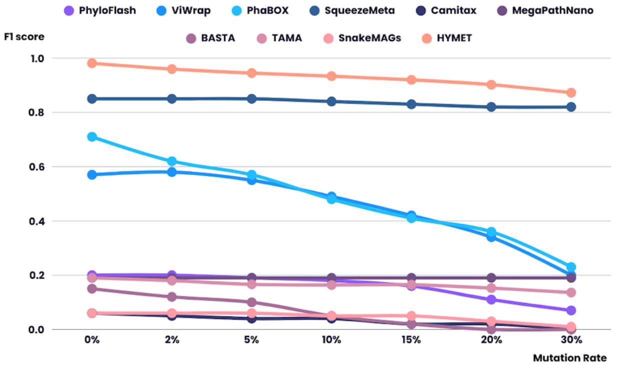

Figure 3. Performance of the state-of-the-art tools and HYMET as the mutation rate increases. The x-axis represents the mutation rate (ranging from 0% to 30%), while the y-axis shows the F1 score. Each curve on the graph corresponds to a different tool.

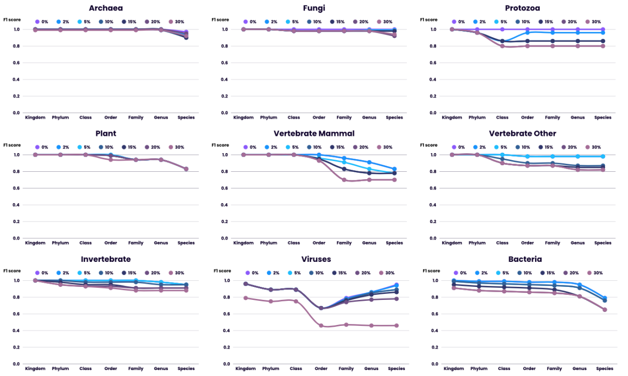

Figure 4. Performance of HYMET across taxonomic levels for different taxonomic groups as mutation rates increase (0% to 30%). The x-axis shows taxonomic levels (kingdom to species), and the y-axis represents the F1 score. Each curve corresponds to a specific mutation rate, and each graph focuses on a particular taxonomic group.

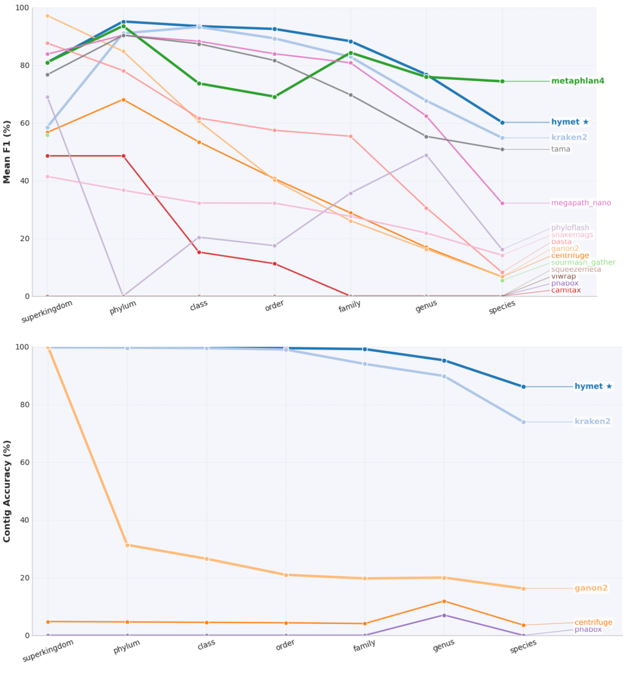

Figure 5. Top: mean F1 by rank (superkingdom to species) across seven assemblies. Bottom: mean contig-level accuracy by rank.

sensitivity). Install footprint remains 2.82 GB; dynamically downloaded references typically add 10–50 GB (disk-footprint details are provided in the Supplementary Material).

CAMI Benchmark

We benchmarked HYMET and several contig-mode classification tools using diverse CAMI assemblies. Figures 5–7 summarize the classification performance at different taxonomic ranks and resource usage across these tools.

Figure 5 (top) shows that HYMET consistently outperforms most other tools at various taxonomic ranks, particularly from family up to class. While MetaPhlAn 4 achieves high species-level accuracy, it underperforms at intermediate ranks. Index-based classifiers like Kraken 2 and Centrifuge demonstrate reduced accuracy at lower taxonomic ranks due to lower recall rates. Figure 5 (bottom) highlights the stability and consistency of HYMET's contig-level accuracy across ranks, contrasting sharply with tools relying solely on k-mer indices, which exhibit more variability at lower ranks. Only tools that emit per-contig classifications (HYMET, Kraken 2, Centrifuge, Ganon 2, etc.) appear in the contig-accuracy panel because marker-based profilers and read-focused workflows do not produce contig-level outputs.

Profile-distance metrics reinforce HYMET's advantage. As

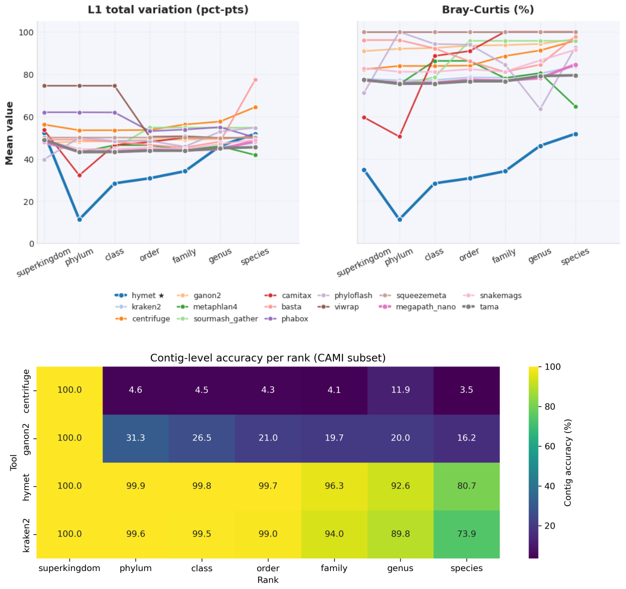

Figure 6. Top: mean L1 total variation and Bray–Curtis dissimilarity by rank across the seven CAMI assemblies. Bottom: per-rank CAMI accuracy heatmap for the profilers benchmarked.

Table 5. CAMI profile-distance summary (mean across taxonomic ranks and assemblies). Lower values indicate improved alignment with CAMI truth profiles.

Tool	Mean L1 (pct pts)	Mean Bray–Curtis (%)	Mean F1 (%)
HYMET	36.35	33.88	83.89
MetaPhlAn 4	45.21	78.34	78.83
Kraken 2	45.21	78.96	76.76
TAMA	44.76	77.17	73.11
MegaPath-Nano	45.10	77.95	74.53

shown in Figure 6 (top), HYMET attains the lowest Bray–Curtis dissimilarity at all ranks and the lowest or near-lowest L1 at most ranks (with genus, species, and superkingdom showing narrow leads by MegaPath-Nano, MetaPhlAn 4, and phyloFlash, respectively). This indicates tighter abundance estimates than competing profilers overall. The heatmap in Figure 6 (bottom) illustrates how this translates into rank-wise accuracy breadth, particularly below the family level where index-only tools lose recall. The heatmap is restricted to tools that emit per-contig assignments (HYMET and the index-only classifiers Kraken 2, Centrifuge, and Ganon 2) because marker-based profilers (e.g., MetaPhlAn 4) and pipeline workflows (e.g., TAMA, SqueezeMeta) report profiles only and do not provide per-contig labels.

Table 5 complements these plots: HYMET lowers mean L1 deviation by roughly nine percentage points relative to Kraken 2 and MetaPhlAn 4 while retaining the strongest average F1. Tools optimized for speed, such as MegaPath-Nano, exhibit higher profile distances despite competitive F1 values, highlighting the trade-off between coarse abundance estimates and precise community reconstruction.

Resource utilization data (Figure 7) shows HYMET's balanced performance, averaging approximately 116 seconds of wall-clock time and 6.2 GB of peak memory usage across the seven assemblies. MetaPhlAn 4 required greater resources (about 147 seconds and 18.8 GB), while Kraken 2 and MegaPath-Nano operated faster (approximately 40 and 24 seconds, respectively) but sacrificed accuracy at finer taxonomic resolutions. TAMA provided balanced precision but demanded higher memory usage (around 16.9 GB).

Table 6 summarizes these performance trends. Overall, HYMET delivered the highest genus-level F1 score (76.75%), a competitive species-level F1 score (60.18%), and the strongest overall average across ranks (83.89%) driven by balanced precision and recall (62.59% / 62.00%). MetaPhlAn 4 excelled at species-level accuracy

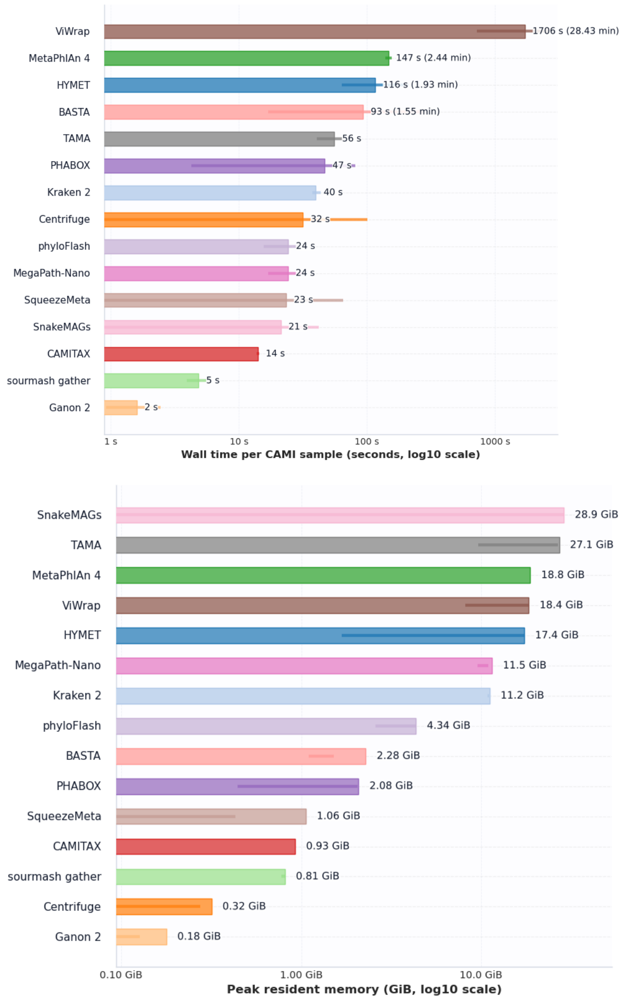

Figure 7. CAMI resource envelopes. Aggregated wall-clock time (top) and peak resident memory (bottom) per tool across the seven assemblies (canonical multi-tool suite; tight candidate cap).

Table 6. CAMI summary: mean F1 (%) at genus, species, and across all ranks (Avg F1), mean precision/recall at species, mean wall time (s) and mean peak memory (GB) across seven assemblies (canonical multi-tool suite; tight candidate cap).

Tool	Genus F1	Species F1	Avg F1	Species Precision	Species Recall	Wall time	Peak GB
HYMET	76.75	60.18	83.89	62.59	62.00	115.93	6.24
MetaPhlAn 4	75.90	74.38	78.83	75.46	77.92	146.54	18.76
Kraken 2	67.68	54.81	76.76	69.40	47.19	39.86	10.95
TAMA	55.31	50.81	73.11	79.52	40.14	55.61	16.88
MegaPath-Nano	62.38	32.19	74.53	45.63	25.54	24.11	10.20

(74.38%, precision 75.46%, recall 77.92%) but lagged at intermediate ranks, yielding a lower overall F1 average (78.83%). Kraken 2 and MegaPath-Nano completed faster but showed reduced sensitivity at lower ranks (species F1: 54.81% and 32.19%; averages 76.76% and 74.53%) with skewed precision/recall. TAMA balanced precision (79.52%) against higher memory usage (16.88 GB) and obtained an overall average of 73.11%. Together with Figure 5, these results show that HYMET's hybrid design sustains recall into lower ranks without sacrificing precision or inflating resource costs.

HYMET Read vs Contig Modes

Figures 8 and 9 provide a detailed comparison between HYMET's contig-based workflow and its synthetic-read workflow across CAMI assemblies, highlighting differences in rank-wise performance and computational resource usage. Aggregated metrics that support these visual comparisons are summarized comprehensively in Table 7.

As illustrated in Figure 8, both workflows exhibit closely matched F1 scores across taxonomic ranks. However, the synthetic-read approach demonstrates modestly improved abundance distance metrics at intermediate and lower ranks, as evidenced by lower L1 total variation and Bray–Curtis dissimilarity scores.

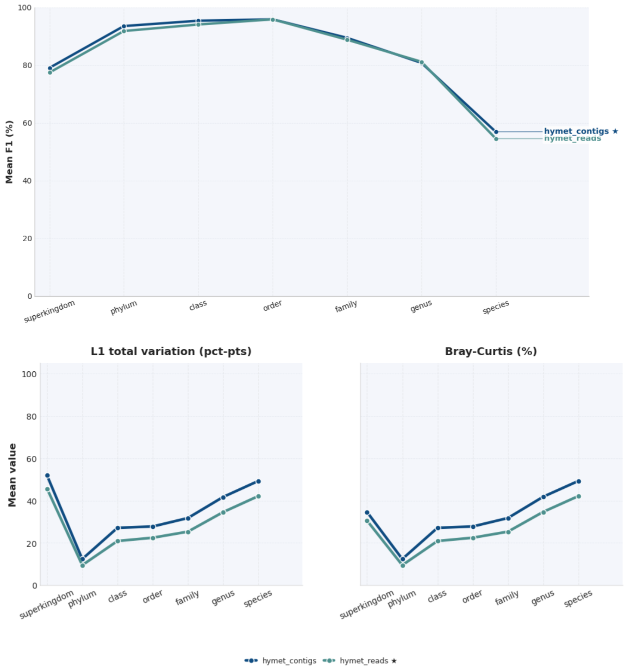

Figure 8. Comparison of HYMET's contig and synthetic-read workflows across CAMI assemblies. Top panel: Mean F1 scores across taxonomic ranks (superkingdom to species). Bottom panel: Mean abundance distances, with L1 total variation represented by solid lines and Bray–Curtis dissimilarity represented by dashed lines.

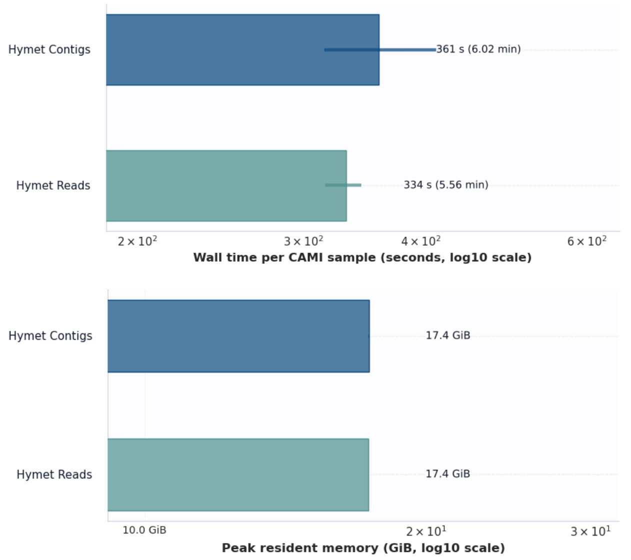

Figure 9. Resource usage comparison between HYMET contig and read workflows, measured across seven CAMI assemblies (HYMET-only suite; expanded candidate budget). Top panel: Wall-clock execution time (seconds). Bottom panel: Peak memory usage (GB).

The resource usage depicted in Figure 9 confirms negligible differences between the two workflows in terms of wall-clock time and memory consumption. This similarity arises because both modes employ identical downstream reference database construction, alignment strategies, and classification methodologies.

Table 7 quantifies these observations, showing that while the synthetic-read workflow achieves comparable recall and overall F1 scores relative to the contig-based workflow (F1: 83.37 vs. 84.41), it provides slightly improved abundance distances (L1: 28.63 vs. 34.55; Bray–Curtis: 26.49 vs. 32.09). Furthermore, both workflows demonstrate similar resource efficiency in terms of execution time

Table 7. HYMET contig vs read comparison across seven CAMI assemblies: mean precision/recall/F1, mean L1 total variation (percentage points), Bray–Curtis (%), wall-clock time (s), and peak resident memory (GB) (HYMET-only suite; expanded candidate budget).

Mode	Precision	Recall	F1	L1	Bray–Curtis	Wall time	Peak GB
HYMET (contigs)	82.15	89.90	84.41	34.55	32.09	361.19	17.37
HYMET (reads)	80.05	90.57	83.37	28.63	26.49	333.61	17.36

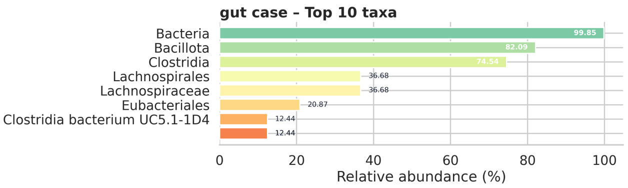

Figure 10. Top taxa panel for the human gut assembly. Panels display the most abundant ranks from superkingdom through species for this sample.

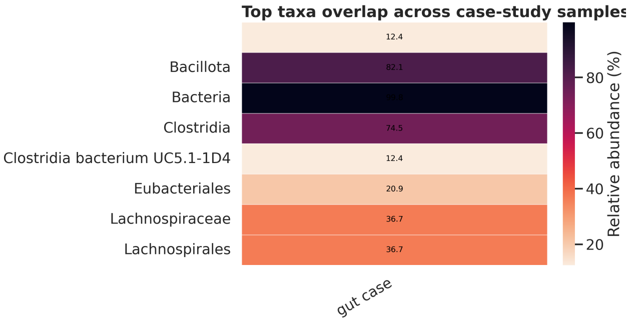

Figure 11. Abundance heatmap for the gut assembly. Color intensity encodes relative abundance across taxonomic ranks; darker cells indicate higher contributions.

(333.61 vs. 361.19 seconds) and memory usage (17.36 vs. 17.37 GB), ilustrating the robustness of HYMET's pipeline across different input modalities.

Gut and Zymo Case-study Results

To extend the evaluation beyond CAMI benchmarks, we analyzed two real-world metagenomic datasets: the human gut assembly from MGnify and the ZymoBIOMICS mock community from the Loman Lab. Figures 10 and 12 illustrate the primary taxonomic profiles for these samples, while Figures 11 and 13 provide detailed abundance heatmaps. Computational resource usage for both analyses is summarized in Table 9.

At the contig level, HYMET assigned species/strain labels to 82.82% of gut contigs; in the Zymo mock, HYMET produced species-level labels for 75.00% of predicted contigs (54/72), and among truth-matched contigs (n=61) exactly matched the curated species for 44.26% (27/61; 56.25% of species-assigned), consistent with genus-level substitutions.

In the human gut sample, HYMET identified a microbiome predominantly composed of Bacillota (Firmicutes; 82.09%), followed by significant contributions from Pseudomonadota (9.59%) and Actinomycetota (8.17%) at the phylum level. At the class level, Clostridia strongly dominated (74.54%), particularly represented by the orders Lachnospirales (36.68%) and Eubacteriales (20.87%), with Lachnospiraceae notably prevalent at the family level. These findings reflect typical adult gut microbiomes characterized by obligate anaerobic bacteria. Among the identified species, the most abundant were *Clostridia bacterium UC5.1-1D4* (12.44%), *[Clostridium] scindens* (9.49%), *Coprococcus phoceensis* (6.85%), *Longicatena caecimuris* (6.53%), *Roseburia intestinalis* (6.21%), and *Ruthenibacterium lactatiformans* (5.61%). Additionally, lower-level signals such as *Escherichia sp. KTE172* (5.47%) were also detected (Figure 10). The gut community heatmap highlighted dense abundance clusters within the Lachnospiraceae and Oscillospiraceae families, reinforcing these results (Figure 11).

The Zymo mock community comprises ten known organisms,

Table 8. Zymo bacterial species: truth (renormalized within Bacteria) versus HYMET profile. Notes indicate genus-level matches where the exact species differs.

Species	Within-Bacteria (%)		Note
	Truth	HYMET	
Escherichia coli	18.15	22.22	exact
Salmonella enterica	21.20	12.50	exact
Bacillus subtilis	16.33	15.28	genus match (B. spizizenii)
Listeria monocytogenes	12.12	13.89	exact
Enterococcus faecalis	11.54	8.33	exact
Staphylococcus aureus	11.23	9.72	exact
Limosilactobacillus fermentum	8.23	6.94	exact
Pseudomonas aeruginosa	1.20	4.17	genus match (Pseudomonas sp.)

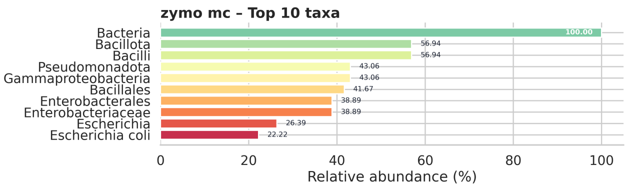

Figure 12. Top taxa panel for the Zymo mock community. Panels display the most abundant ranks across superkingdom through species for this sample.

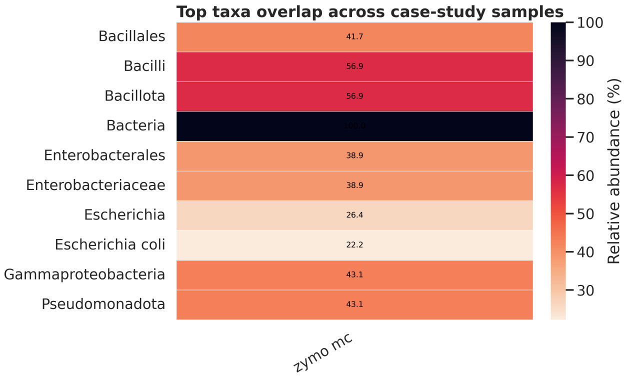

Figure 13. Abundance heatmap for the Zymo mock community. Color intensity encodes relative abundance across taxonomic ranks; darker cells indicate higher contributions.

including eight bacterial species and two yeasts (*Saccharomyces cerevisiae* and *Cryptococcus neoformans*). Although yeasts represent 46.80% of the truth profile, in this run HYMET did not recover the eukaryotic component, and the reported profile is restricted to bacterial taxa (53.20%). Within bacteria, HYMET accurately identified major species such as *Escherichia coli* (22.22%), *Listeria monocytogenes* (13.89%), and *Salmonella enterica* (12.50%). Minor genus-level substitutions occurred: *Bacillus* spizizenii in place of *B. subtilis*, and *Pseudomonas* sp. in place of *P. aeruginosa*. Table 8 compares the bacterial abundances (renormalized within bacteria) to HYMET's predictions, showing six exact species matches and two genus-level substitutions. The Zymo abundance patterns concentrate within Enterobacterales and Bacillales, consistent with Figure 12 and Figure 13.

Computationally, both datasets exhibited similar performance metrics. HYMET completed the analyses efficiently in approximately 4–5 minutes each, with peak memory usage consistently around 17.4 GB, showcasing the robustness and scalability of the method across diverse metagenomic contexts (Table 9).

Table 9. Case-study runtime and memory summary on the reference machine.

Sample	Wall time (s)	Peak RSS (GB)
gut_case	258.32	17.41
zymo_mc	255.73	17.42

5 Zymo Ablation Results

To assess robustness to incomplete references, we ran an ablation study on the Zymo dataset using the canonical suite, progressively removing species/strain-level entries and re-indexing at each level. Across ablation levels, runtime and memory remained stable (~4.2–4.5 minutes, ~17.4 GB). Classification quality degrades as expected at finer ranks: the share of species/strain assignments falls from 75.00% to 64.52%, while higher/unknown rises from 25.00% to 35.48%; genus F1 drops from 88.89% to 58.82% and species F1 from 57.14% to 0.00% at full ablation.

Table 10. Zymo ablation summary. Levels indicate the ablation proportion; totals are number of contigs classified.

Level (%)	Total classified	Species/strain (%)	Higher/unknown (%)	Genus F1 (%)	Species F1 (%)
0	72	75.00	25.00	88.89	57.14
25	69	72.46	27.54	88.89	57.14
50	71	73.24	26.76	88.89	43.48
75	66	66.67	33.33	73.68	13.79
100	62	64.52	35.48	58.82	0.00

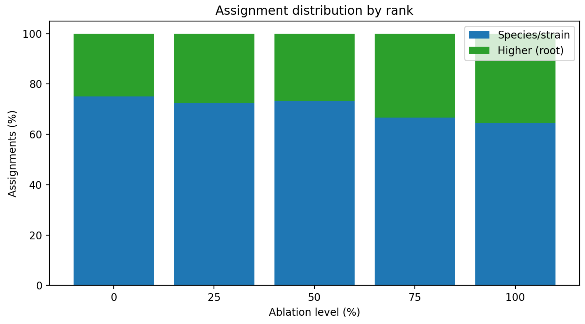

Figure 14. Ablation o distribution of assignments by rank group across ablation levels on Zymo.

Figure 14 shows a gradual reweighting from species/strain toward higher ranks as references are withheld: species/strain drops from 75.00% to 64.52%, while higher/unknown rises from 25.00% to 35.48%; the number of classified contigs declines modestly (72 → 62). Genus and family contributions remain stable through 50% ablation and only drift at ≥75%, indicating HYMET backs off to appropriate higher ranks rather than emitting incorrect fine-grained labels when exact references are missing.

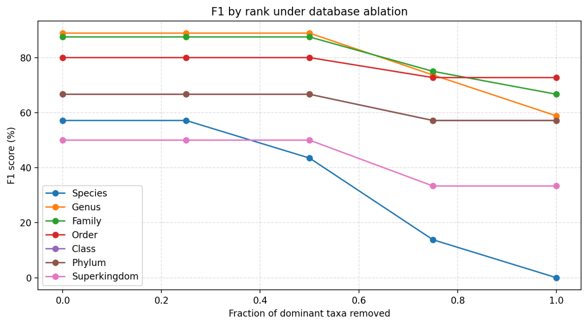

Figure 15. Ablation on Zymo (canonical): F1 by rank under increasing ablation.

Rank-wise accuracy (Figure 15) remains flat at coarse levels, while genus F1 stays high through 50% (88.89%) and then declines at 75% (73.68%) and 100% (58.82%). Species F1 degrades from 57.14% to 0.00% under full ablation, reflecting the intended removal of discriminative references. Throughout, runtime and memory are effectively unchanged (~4.2–4.5 minutes; ~17.4 GB), isolating reference incompleteness as the driver of accuracy loss rather than compute differences.

6 Discussion

HYMET effectively addresses significant challenges in metagenomic analysis by integrating adaptive MinHash-based prefiltering with precise alignment and a coverage-weighted Lowest Common Ancestor (LCA) classification. This hybrid design builds upon previous approaches such as Metalign's combination of CMash and Minimap2, yet it uniquely introduces an adaptive candidate selection method, dynamically generated cross-domain reference databases, and an evidence-weighted LCA step. Compared to fixed-threshold methods, such as CAMITAX, HYMET's adaptive strategy enhances sensitivity to divergent organisms, avoiding the exclusion of potentially relevant candidates and ensuring broader domain applicability [4, 70].

Our evaluations across diverse CAMI benchmarks demonstrate HYMET's robust accuracy, computational efficiency, and stable performance across taxonomic ranks. Specifically, HYMET achieved a mean F1 of 83.89% across ranks, a strong genus-level F1 of 76.75%, and a competitive species-level F1 of 60.18%, while maintaining low computational resource demands (average runtime of 116 seconds and peak memory usage of approximately 6.2 GB). Unlike marker-based tools such as MetaPhlAn 4, which excel at species-level assignments but sacrifice intermediate-rank accuracy and per-contig labeling capabilities, HYMET balances precision and recall consistently across multiple ranks, which is crucial for downstream metagenomic workflows such as genome binning [71, 15, 23].

HYMET's adaptive cache management strategy allows predictable and flexible resource usage. Both contig and synthetic-read analyses converged in memory usage when sharing reference caches, differing slightly only in runtime due to alignment parameter presets. Practically, users can thus optimize for rapid exploratory analyses or deeper comparative studies across related samples by adjusting candidate cache parameters without compromising accuracy.

Importantly, HYMET demonstrates resilience against genetic mutations, maintaining robust performance (F1 ≥ 0.8) for diverse taxonomic groups even at mutation rates up to 30%. While viral sequences experienced a notable decrease in accuracy (F1 ≈ 0.5 at 30%), this likely reflects genuine biological and database limitations rather than methodological shortcomings. The stability in performance arises primarily from the optimized choice of screening parameters—smaller k-mers and larger sketch sizes for divergent genomes—and the robust seed-chain-extend alignment method employed by Minimap2 [20, 41].

The ZymoBIOMICS case study provided additional insight into HYMET's limitations. While bacterial taxa were accurately classified (six exact matches, two genus-level substitutions), the method failed to recover yeast species, which represented a significant proportion of the mock community. The systematic ablation experiment further clarified this behavior, showing HYMET appropriately adjusts assignments to higher taxonomic ranks when species-level references are incomplete or missing. This indicates that future versions could benefit significantly from domain-specific adjustments to candidate thresholds, particularly enhancing sensitivity towards underrepresented groups like yeasts.

A notable operational challenge identified was related to reference database completeness and retrieval reliability. The hybrid reference databases, combining older public sketches with newly generated local sketches, exhibited a non-negligible retrieval failure rate (6.04%), significantly impacting classification accuracy, particularly at lower taxonomic levels. This emphasizes the importance of maintaining continuously updated, comprehensive databases, systematically refreshing manifests, and implementing robust checksum and fallback protocols to mitigate data retrieval issues [67, 17].

HYMET demonstrated robust accuracy across bacteria, archaea, fungi, and small eukaryotes, emphasizing its suitability across diverse microbial communities. However, challenges remain in classifying more complex eukaryotic organisms due to inherent genome complexity, substantial intra-species diversity, and biases towards well-studied taxa in reference databases [72]. Similarly, the polyphyletic and rapidly evolving nature of viral genomes underscores the importance of regularly updated, comprehensive databases to maintain accurate viral taxonomic classification [73, 74].

Future improvements should focus on directly addressing identified limitations: enhancing domain-specific candidate thresholds (particularly for viruses and eukaryotes), implementing scheduled updates of sketch databases enriched with underrepresented taxa, refining the weighted LCA with explicit rules for resolving multi-mapped alignments and establishing minimum evidence thresholds. Integrating uncertainty quantification methods, such as bootstrapping or replicate analyses, will further enhance the reliability and practical applicability of HYMET.

7 Conclusion

HYMET advances metagenomic classification by integrating adaptive MinHash screening, precise Minimap2 alignment, and a coverage-weighted Lowest Common Ancestor algorithm. Evaluations across diverse CAMI benchmarks demonstrate that HYMET achieves robust accuracy, consistently outperforming or matching existing methods at multiple taxonomic ranks, notably with an average F1 score of 83.89%, including 76.75% at the genus level and 60.18% at the species level. This high performance is maintained even under substantial genetic divergence, indicating strong mutation resilience.

Real-world validations further confirm HYMET's practical value, successfully identifying expected bacterial communities from human gut and ZymoBIOMICS mock samples. Computationally efficient and lightweight, HYMET's adaptive and dynamic caching strategy ensures reproducible and resource-predictable analyses suitable for diverse deployment contexts.

While current results illustrate excellent overall accuracy and scalability, future enhancements could include domain-specific reference optimizations, explicit handling of multi-mapping ambiguities, and integration of machine learning classifiers to further improve lower-rank discrimination. Such developments would position HYMET as a versatile platform, supporting reliable, efficient, and scalable metagenomic investigations across various biological contexts.

8 Availability of source code and requirements

- Project name: HYMET (Hybrid Metagenomic Tool)
- Project home page: `https://github.com/ieeta-pt/HYMET`
- Operating system(s): Linux
- Programming language: Python (primary), Perl (legacy), Bash
- Other requirements: Docker or Apptainer/Singularity; Conda/Mamba
- License: MIT.

9 Data Availability

The supplementary material describes the full reproducibility workflow (environment setup, scripts, execution logs, and additional figures). Digital resources used in the manuscript are listed below.

- HYMET source code, CAMI/reads-versus-contigs/case manifests, runtime logs, and aggregated TSV/figure outputs are versioned in the project repository (`https://github.com/ieeta-pt/HYMET`; see `results/`, `bench/`, and `case/` subdirectories).
- The Syst_Review repository retains the systematic benchmark harness for third-party tools, including installation recipes and evaluation scripts (`https://github.com/inesbmartins02/Syst_Review`).
- The Mash sketch databases used for candidate selection (`sketch1.msh`, `sketch2.msh`, `sketch3.msh`) are deposited at Zenodo (doi:10.5281/zenodo.17428354); checksums are mirrored in the repository (`sketch\_sha256.txt`).
- CAMI contig assemblies used in the benchmark are enumerated in `bench/cami_manifest.tsv`; running `bench/fetch_cami.sh` downloads the official CAMI I sample_0 bundle from the Publisso mirror (`https://frl.publisso.de/data/frl:6421672/dataset/2017.12.29_11.37.26_sample_0_contigs.tar`), extracts the required assets into `/data/cami/`, caches the archive, and regenerates the lightweight subsets (`cami_i_*`, `cami_ii_*`) via `tools/generate_cami_subsets.py` so every benchmark run starts from the same inputs.
- The test and validation dataset (26,203 genomes, 14.76 GB) was derived from the NCBI RefSeq Assembly database (`https://ftp.ncbi.nlm.nih.gov/genomes/refseq/`); assembly summary files and scripts for replication are provided in Supplementary Material Section 3.
- Reference genomes for dynamic database construction are retrieved from the NCBI Assembly database (`https://www.ncbi.nlm.nih.gov/assembly/`) using GCF and GCA accession numbers identified through Mash screening. GTDB r202 genomes included in the sketch databases are available from the Genome Taxonomy Database (`https://gtdb.ecogenomic.org/`).
- Case-study inputs can be fetched with `case/fetch_case_data.sh`, which retrieves the Zymo mock assembly (Nanopore S3: `http://nanopore.s3.climb.ac.uk/mockcommunity/v3/7cd60d3b-eafb-48d1-9aab-c8701232f2f8.ctg.cns.fa`) and the MGnify gut assembly (EBI API: `https://www.ebi.ac.uk/metagenomics/api/v1/analyses/MGYA00794604/file/ERZ24911249_FASTA.fasta.gz`) before staging them under `/data/case/`. The manifest `case/manifest.tsv` records the same paths for reference.
- Zymo ablation outputs (candidate lists, cached references, evaluation tables, figures) are versioned under `results/ablation/canonical/run_20251031T191804Z/`; the workflow can be rerun with `case/run_ablation.sh` as documented in Supplementary Section 6.

10 Additional Files

Supplementary Tab. S1. On-disk resource footprint (install + references) for every benchmarked tool in the canonical CAMI environ-

ment.

Supplementary Tab. S2. Provenance and composition of the reference databases used by each tool, including shared corpora and official releases.

Supplementary Tab. S3 to S10. Per-domain benchmarking tables (viruses, archaea, bacteria, fungi, protozoa, plants, invertebrates, vertebrates) reporting precision, recall, and F1 across taxonomic ranks at 0% mutation.

Supplementary Tab. S11 to S17. HYMET-only benchmarking tables summarizing precision, recall, and F1 across taxonomic ranks under mutation rates from 0% to 30%.

Supplementary Fig. S1 and S2. Line plots showing how tool performance varies with mutation rate across taxonomic levels for each biological domain.

Supplementary Fig. S3. Scatter plots relating execution time (hours) to F1 score (0.0–1.0) for all evaluated tools, presented per domain.

10.1 List of abbreviations

BASTA: BAsic Sequence Taxonomy Annotation;
BLAST: Basic Local Alignment Search Tool;
CAMI: Critical Assessment of Metagenome Interpretation;
CAMITAX: CAMI TAXonomy (tool for taxon labels);
CMash: Containment MinHash;
CPU: Central Processing Unit;
CSV: Comma-Separated Values;
DOI: Digital Object Identifier;
F1: Harmonic mean of precision and recall;
FCT: Fundação para a Ciência e a Tecnologia;
GCA: GenBank assembly accession;
GCF: RefSeq assembly accession;
GTDB: Genome Taxonomy Database;
HYMET: Hybrid Metagenomic Tool;
LCA: Lowest Common Ancestor;
NCBI: National Center for Biotechnology Information;
PAF: Pairwise mApping Format;
RefSeq: NCBI Reference Sequence database;
RSS: Resident Set Size;
SSU rRNA: Small Subunit ribosomal RNA;
TAMA: Taxonomy Analysis pipeline for metagenome using Meta-Analysis;
TaxID: Taxonomy Identifier;
TSV: Tab-Separated Values.

Funding

This work has received funding from the FCT (Foundation for Science and Technology) under unit 00127-IEETA and through the project Advanced Genomic Data Processing in Portuguese FEGA Node (ref. 2023.14342.CPCA.A1; DOI: 10.54499/2023.14342.CPCA.A1). J.M.S. has received funding from the European Commission under grant agreement 101081813 (Genomic Data Infrastructure).

References

1. Kim D, Song L, Breitwieser FP, Salzberg SL. Centrifuge: rapid and sensitive classification of metagenomic sequences. Genome research 2016;26(12):1721–1729.
2. Simon HY, Siddle KJ, Park DJ, Sabeti PC. Benchmarking metagenomics tools for taxonomic classification. Cell 2019;178(4):779–794.
3. Wood DE, Salzberg SL. Kraken: ultrafast metagenomic sequence classification using exact alignments. Genome biology 2014;15(3):1–12.
4. Bremges A, Fritz A, McHardy AC. CAMITAX: Taxon labels for microbial genomes. GigaScience 2020;9(1):giz154.
5. Kim N, Ma J, Kim W, Kim J, Belenky P, Lee I. Genome-resolved metagenomics: a game changer for microbiome medicine. Experimental & Molecular Medicine 2024;56(7):1501–1512.
6. Mallawaarachchi V, Lin Y. Accurate binning of metagenomic contigs using composition, coverage, and assembly graphs. Journal of Computational Biology 2022;29(12):1357–1376.
7. Ayling M, Clark MD, Leggett RM. New approaches for metagenome assembly with short reads. Briefings in bioinformatics 2020;21(2):584–594.
8. Wood DE, Lu J, Langmead B. Improved metagenomic analysis with Kraken 2. Genome biology 2019;20:1–13.
9. Lema NK, Gemeda MT, Woldesemayat AA. Recent Advances in Metagenomic Approaches, Applications, and Challenges. Current Microbiology 2023;80(11):347.
10. Martins IB, Miguel Silva J, Almeida JR. A comprehensive study of databases to assess the reliability of metagenomic tools. In: 2024 IEEE Conference on Computational Intelligence in Bioinformatics and Computational Biology (CIBCB); 2024. p. 1–6.
11. Xu R, Rajeev S, Salvador LC. The selection of software and database for metagenomics sequence analysis impacts the outcome of microbial profiling and pathogen detection. Plos one 2023;18(4):e0284031.
12. Breitwieser FP, Lu J, Salzberg SL. A review of methods and databases for metagenomic classification and assembly. Briefings in bioinformatics 2019;20(4):1125–1136.
13. Kieser S, Brown J, Zdobnov EM, Trajkovski M, McCue LA. ATLAS: a Snakemake workflow for assembly, annotation, and genomic binning of metagenome sequence data. BMC bioinformatics 2020;21:1–8.
14. Tadrent N, Dedeine F, Hervé V. SnakeMAGs: a simple, efficient, flexible and scalable workflow to reconstruct prokaryotic genomes from metagenomes. F1000Research 2022;11.
15. Tamames J, Puente-Sánchez F. SqueezeMeta, a highly portable, fully automatic metagenomic analysis pipeline. Frontiers in microbiology 2019;9:425882.
16. Clarke EL, Taylor LJ, Zhao C, Connell A, Lee JJ, Fett B, et al. Sunbeam: an extensible pipeline for analyzing metagenomic sequencing experiments. Microbiome 2019;7:1–13.
17. Chaumeil PA, Mussig AJ, Hugenholtz P, Parks DH, GTDB-Tk: a toolkit to classify genomes with the Genome Taxonomy Database. Oxford University Press; 2020.
18. Buchfink B, Xie C, Huson DH. Fast and sensitive protein alignment using DIAMOND. Nature methods 2015;12(1):59–60.
19. Kahlke T, Ralph PJ. BASTA–Taxonomic classification of sequences and sequence bins using last common ancestor estimations. Methods in Ecology and Evolution 2019;10(1):100–103.
20. Ondov BD, Treangen TJ, Melsted P, Mallonee AB, Bergman NH, Koren S, et al. Mash: fast genome and metagenome distance estimation using MinHash. Genome biology 2016;17:1–14.
21. Menzel P, Ng KL, Krogh A. Fast and sensitive taxonomic classification for metagenomics with Kaiju. Nature communications 2016;7(1):11257.
22. Callahan BJ, McMurdie PJ, Rosen MJ, Han AW, Johnson AJA, Holmes SP. DADA2: High-resolution sample inference from Illumina amplicon data. Nature methods 2016;13(7):581–583.
23. Sim M, Lee J, Lee D, Kwon D, Kim J. TAMA: improved metagenomic sequence classification through meta-analysis. BMC bioinformatics 2020;21:1–17.
24. Ounit R, Wanamaker S, Close TJ, Lonardi S. CLARK: fast and accurate classification of metagenomic and genomic sequences

using discriminative k-mers. BMC genomics 2015;16(1):1–13.
25. Breitwieser FP, Baker DN, Salzberg SL. KrakenUniq: confident and fast metagenomics classification using unique k-mer counts. Genome Biology 2018;19(1):198. https://doi.org/10.1186/s13059-018-1568-0.
26. Piro VC, Dadi TH, Seiler E, Reinert K, Renard BY. ganon: precise metagenomics classification against large and up-to-date sets of reference sequences. Bioinformatics 2020 Jul;36(Suppl_1):i12–i20.
27. Piro VC, Reinert K. ganon2: up-to-date and scalable metagenomics analysis. NAR Genomics and Bioinformatics 2025 07;7(3):lqaf094. https://doi.org/10.1093/nargab/lqaf094.
28. Song L, Langmead B. Centrifuger: lossless compression of microbial genomes for efficient and accurate metagenomic sequence classification. Genome Biology 2024;25(1):106. https://doi.org/10.1186/s13059-024-03244-4.
29. Ulrich JU, Renard BY. Taxor: Fast and space-efficient taxonomic classification of long reads with hierarchical interleaved XOR filters. bioRxiv 2023;https://www.biorxiv.org/content/early/2023/07/22/2023.07.20.549822.
30. Brown CT, Irber L. sourmash: a library for MinHash sketching of DNA. Journal of Open Source Software 2016;1(5):27. https://doi.org/10.21105/joss.00027.
31. Shang J, Peng C, Liao H, Tang X, Sun Y. PhaBOX: a web server for identifying and characterizing phage contigs in metagenomic data. Bioinformatics Advances 2023;3(1):vbad101.
32. Shang J, Jiang J, Sun Y. Bacteriophage classification for assembled contigs using graph convolutional network. Bioinformatics 2021;37(Supplement_1):i25–i33.
33. Zhou Z, Martin C, Kosmopoulos JC, Anantharaman K. ViWrap: A modular pipeline to identify, bin, classify, and predict viral–host relationships for viruses from metagenomes. Imeta 2023;2(3):e118.
34. Auslander N, Gussow AB, Benler S, Wolf YI, Koonin EV. Seeker: alignment-free identification of bacteriophage genomes by deep learning. Nucleic acids research 2020;48(21):e121–e121.
35. Gałan W, Bąk M, Jakubowska M. Host taxon predictor-a tool for predicting taxon of the host of a newly discovered virus. Scientific reports 2019;9(1):3436.
36. Jiang G, Zhang J, Zhang Y, Yang X, Li T, Wang N, et al. DCiPatho: deep cross-fusion networks for genome scale identification of pathogens. Briefings in Bioinformatics 2023;24(4):bbad194.
37. Altschul SF, Gish W, Miller W, Myers EW, Lipman DJ. Basic local alignment search tool. Journal of molecular biology 1990;215(3):403–410.
38. Gruber-Vodicka HR, Seah BK, Pruesse E. phyloFlash: rapid small-subunit rRNA profiling and targeted assembly from metagenomes. Msystems 2020;5(5):10–1128.
39. Truong DT, Franzosa EA, Tickle TL, Scholz M, Weingart G, Pasolli E, et al. MetaPhlAn2 for enhanced metagenomic taxonomic profiling. Nature methods 2015;12(10):902–903.
40. Lui WW, Leung AW, Leung HC, Xin Y, Teng JL, Woo PC, et al. MegaPath-Nano: Accurate Compositional Analysis and Drug-level Antimicrobial Resistance Detection Software for Oxford Nanopore Long-read Metagenomics. In: 2020 IEEE International Conference on Bioinformatics and Biomedicine (BIBM) IEEE; 2020. p. 329–336.
41. Li H. Minimap2: pairwise alignment for nucleotide sequences. Bioinformatics 2018;34(18):3094–3100.
42. Liang X, Zhang J, Kim Y, Ho J, Liu K, Keenum I, et al. ARGem: a new metagenomics pipeline for antibiotic resistance genes: metadata, analysis, and visualization. Frontiers in Genetics 2023;14:1219297.
43. Prosperi M, Marini S. Karga: Multi-platform toolkit for k-mer-based antibiotic resistance gene analysis of high-throughput sequencing data. In: 2021 IEEE EMBS International Conference on Biomedical and Health Informatics (BHI) IEEE; 2021. p. 1–4.
44. LaPierre N, Alser M, Eskin E, Koslicki D, Mangul S. Metalign: efficient alignment-based metagenomic profiling via containment min hash. Genome Biology 2020;21(1):242. https://doi.org/10.1186/s13059-020-02159-0.
45. Olawoye IB, Frost SD, Happi CT. The Bacteria Genome Pipeline (BAGEP): an automated, scalable workflow for bacteria genomes with Snakemake. PeerJ 2020;8:e10121.
46. Ondov BD, Starrett GJ, Sappington A, Kostic A, Koren S, Buck CB, et al. Mash Screen: high-throughput sequence containment estimation for genome discovery. Genome biology 2019;20:1–13.
47. Baker DN, Langmead B. Dashing: fast and accurate genomic distances with HyperLogLog. Genome biology 2019;20:1–12.
48. Besta M, Kanakagiri R, Mustafa H, Karasikov M, Rätsch G, Hoefler T, et al. Communication-efficient jaccard similarity for high-performance distributed genome comparisons. In: 2020 IEEE International Parallel and Distributed Processing Symposium (IPDPS) IEEE; 2020. p. 1122–1132.
49. Zhao X. BinDash, software for fast genome distance estimation on a typical personal laptop. Bioinformatics 2019;35(4):671–673.
50. Katz LS, Griswold T, Morrison SS, Caravas JA, Zhang S, den Bakker HC, et al. Mashtree: a rapid comparison of whole genome sequence files. Journal of Open Source Software 2019;4(44):10–21105.
51. Broder AZ. On the resemblance and containment of documents. In: Proceedings. Compression and Complexity of SEQUENCES 1997 (Cat. No. 97TB100171) IEEE; 1997. p. 21–29.
52. Pierce NT, Irber L, Reiter T, Brooks P, Brown CT. Large-scale sequence comparisons with sourmash. F1000Research 2019;8:1006.
53. Hernández-Salmerón JE, Moreno-Hagelsieb G. FastANI, Mash and Dashing equally differentiate between Klebsiella species. PeerJ 2022;10:e13784.
54. Hera MR, Liu S, Wei W, Rodriguez JS, Ma C, Koslicki D. Metagenomic functional profiling: to sketch or not to sketch? Bioinformatics 2024;40(Supplement_2):ii165–ii173.
55. Wu W, Li B, Chen L, Gao J, Zhang C. A review for weighted minhash algorithms. IEEE Transactions on Knowledge and Data Engineering 2020;34(6):2553–2573.
56. Sánchez-Reyes A, Fernández-López M. Sketched reference databases for genome-based taxonomy and comparative genomics. Brazilian Journal of Biology 2022;84:e256673.
57. Liu S, Koslicki D. CMash: fast, multi-resolution estimation of k-mer-based Jaccard and containment indices. Bioinformatics 2022 06;38:i28–i35. https://doi.org/10.1093/bioinformatics/btac237.
58. Team MD, Mash Tutorials; 2023. Accessed: 2025-01-10. https://mash.readthedocs.io/en/latest/tutorials.html.
59. Irber L, Brown CT. Lightweight compositional analysis of metagenomes with sourmash gather. Manubot Available at: https://dib-lab github io/2020-paper-sourmash-gather/(Accessed: 16 December 2020) 2020;.
60. Kitts PA, Church DM, Thibaud-Nissen F, Choi J, Hem V, Sapojnikov V, et al. Assembly: a resource for assembled genomes at NCBI. Nucleic acids research 2016;44(D1):D73–D80.
61. Schoch CL, Ciufo S, Domrachev M, Hotton CL, Kannan S, Khovanskaya R, et al. NCBI Taxonomy: a comprehensive update on curation, resources and tools. Database 2020;2020:baaa062.
62. Li H. Minimap and miniasm: fast mapping and de novo assembly for noisy long sequences. Bioinformatics 2016;32(14):2103–2110.
63. Dong J, Liu X, Sadasivan H, Sitaraman S, Narayanasamy S. mm2-gb: GPU accelerated minimap2 for long read dna mapping. In: Proceedings of the 15th ACM International Conference on Bioinformatics, Computational Biology and Health Informatics; 2024. p. 1–9.
64. Langmead B, Wilks C, Antonescu V, Charles R. Scaling read aligners to hundreds of threads on general-purpose processors.

Bioinformatics 2019;35(3):421–432.
65. Rosen G, Garbarine E, Caseiro D, Polikar R, Sokhansanj B. Metagenome Fragment Classification Using N-Mer Frequency Profiles. Advances in bioinformatics 2008;2008(1):205969.
66. Liu B, Gibbons T, Ghodsi M, Treangen T, Pop M. Accurate and fast estimation of taxonomic profiles from metagenomic shotgun sequences. Genome biology 2011;12:1–27.
67. Pruitt KD, Tatusova T, Maglott DR. NCBI reference sequences (RefSeq): a curated non-redundant sequence database of genomes, transcripts and proteins. Nucleic acids research 2007;35(suppl_1):D61–D65.
68. O'Leary NA, Wright MW, Brister JR, Ciufo S, Haddad D, McVeigh R, et al. Reference sequence (RefSeq) database at NCBI: current status, taxonomic expansion, and functional annotation. Nucleic acids research 2016;44(D1):D733–D745.
69. Sayers EW, Beck J, Bolton EE, Bourexis D, Brister JR, Canese K, et al. Database resources of the national center for biotechnology information. Nucleic acids research 2021;49(D1):D10.
70. Jesus TF, Ribeiro-Gonçalves B, Silva DN, Bortolaia V, Ramirez M, Carriço JA. Plasmid ATLAS: plasmid visual analytics and identification in high-throughput sequencing data. Nucleic acids research 2019;47(D1):D188–D194.
71. Blanco-Míguez A, Beghini F, Cumbo F, McIver LJ, Thompson KN, Zolfo M, et al. Extending and improving metagenomic taxonomic profiling with uncharacterized species using MetaPhlAn 4. Nature Biotechnology 2023;41(11):1633–1644. https://doi.org/10.1038/s41587-023-01688-w.
72. Burki F, Roger AJ, Brown MW, Simpson AG. The new tree of eukaryotes. Trends in ecology & evolution 2020;35(1):43–55.
73. Simmonds P, Adams MJ, Benkő M, Breitbart M, Brister JR, Carstens EB, et al. Virus taxonomy in the age of metagenomics. Nature Reviews Microbiology 2017;15(3):161–168.
74. Harris HM, Hill C. A place for viruses on the tree of life. Frontiers in Microbiology 2021;11:604048.

Click here to access/download;Figure;abstract.png

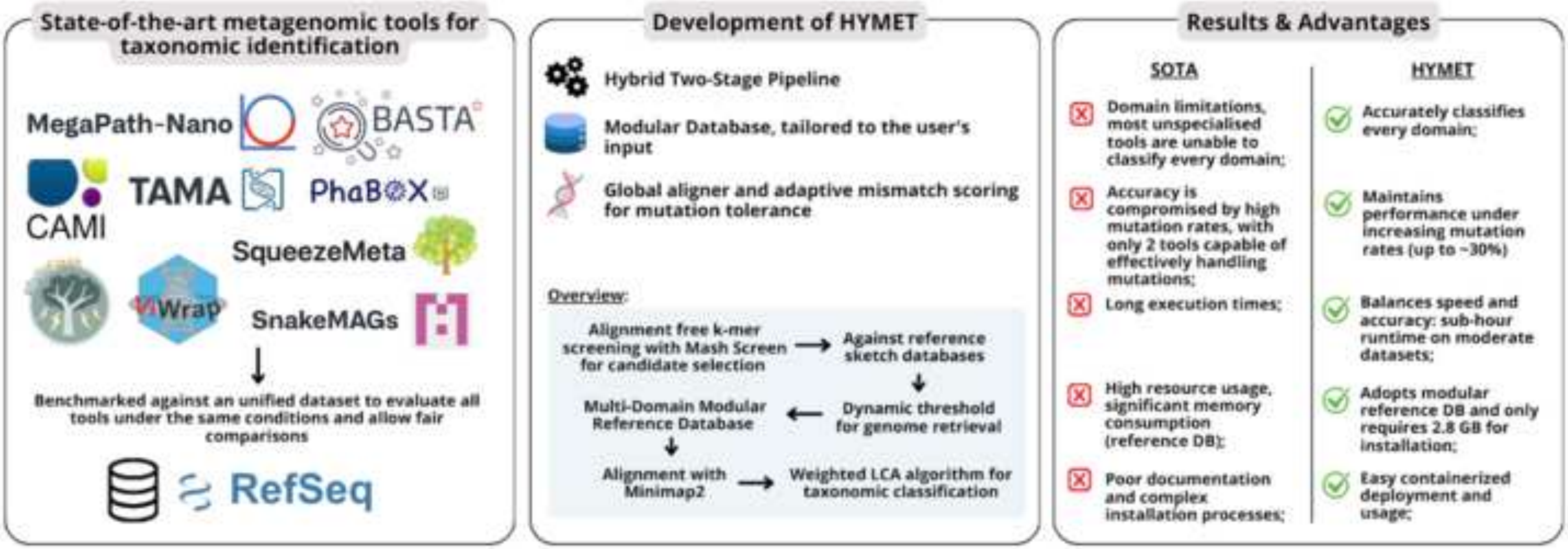

Click here to access/download
Supplementary Material
HYMET___supplementary_material__Copy_ (1).pdf

Click here to access/download
Supplementary Material
main.aux

Click here to access/download
Supplementary Material
main.blg

Click here to access/download
Supplementary Material
main.fdb_latexmk

Click here to access/download
Supplementary Material
main.fls

Click here to access/download
Supplementary Material
main.log

Click here to access/download
Supplementary Material
main.out

Manuscript Click here to access/download;Personal Cover;coverletter.pdf

Jorge Miguel Ferreira da Silva
IEETA/DETI, University of Aveiro
Aveiro, Portugal
jorge.miguel.ferreira.silva@ua.pt
+351 234 370 500

09/05/2025

Editor-in-Chief
GigaScience

Dear Editor,

I am pleased to submit our manuscript entitled **"HYMET: A Hybrid Metagenomic Pipeline for Accurate and Efficient Taxonomic Classification"** for consideration in *GigaScience*. Authored by Inês Martins, João Rafael Almeida, and myself, the work introduces HYMET, a lightweight pipeline that combines rapid k-mer screening with precise alignment to achieve robust taxonomic classification across all biological domains.

Metagenomic studies are often limited by the computational burden of large reference databases, the bias inherent in those resources, and the diminished accuracy of existing tools when confronted with highly mutated or fragmented sequences. HYMET overcomes these barriers through a two-stage workflow: first, *Mash Screen* rapidly filters candidate genomes, and then *Minimap2* refines the search with high-fidelity alignments. A dynamic thresholding algorithm tailors the reference set to each sample, and a weighted lowest-common-ancestor scheme delivers reliable lineage calls down to the species level. In benchmarks encompassing 26 203 genomes (14.76 GB) from Bacteria, Archaea, Eukarya, and viruses, HYMET achieved mean F1 scores above 0.9 and retained performance greater than 0.8 even at 30 % simulated mutation. Typical analyses complete in under two hours on standard workstations, with installation requiring only 2.82 GB and a transient reference cache of 10–50 GB—an efficiency advantage of one to two orders of magnitude over current state-of-the-art pipelines. All source code, sketched reference databases, test data, and analysis scripts are openly available under permissive licences, ensuring full reproducibility.

These contributions align directly with *GigaScience*'s commitment to disseminating reproducible, data-intensive research. The manuscript is original, not under review elsewhere, and all authors have approved its submission. No ethical approvals are required, as the study is entirely computational, and there are no competing interests to declare.

Thank you for considering our work. We believe this paper will be of significant interest to the journal's readership, providing both a methodological advance and a freely accessible resource for the metagenomics community. We look forward to your response and would be happy to supply any additional information you may require.

Sincerely,

Jorge Miguel Ferreira da Silva

Manuscript

Click here to access/download;Graphical Abstract;abstract.pdf

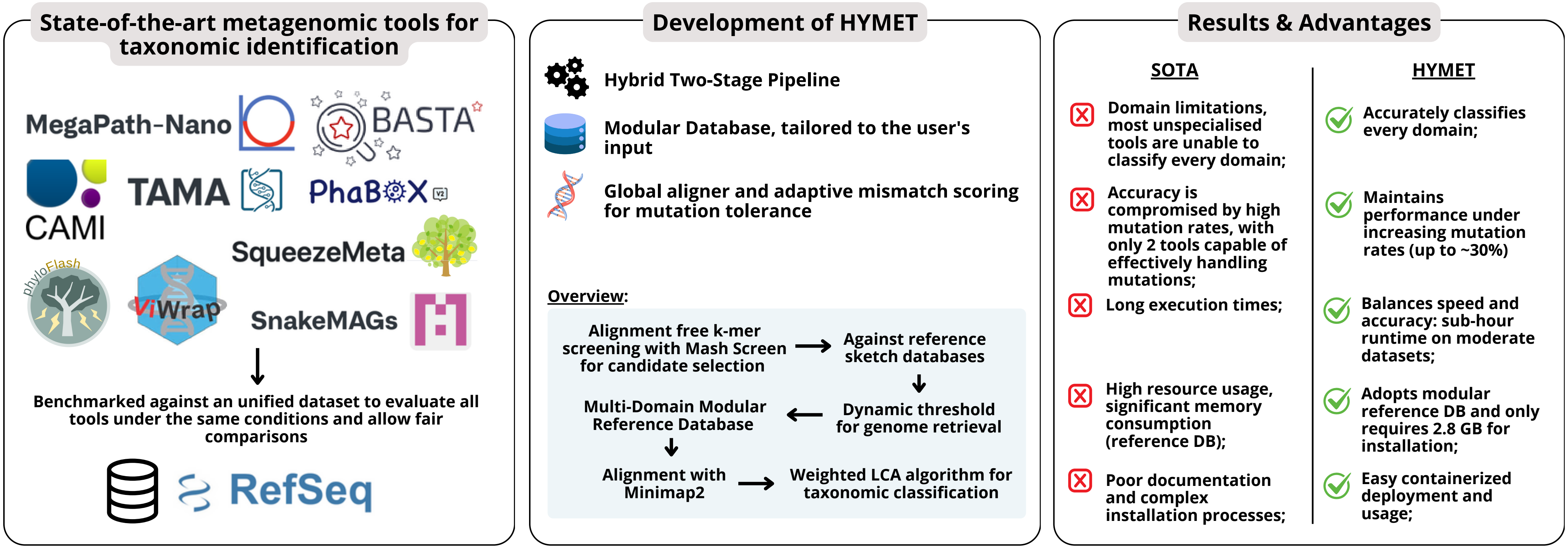

Supplement: giag024_GIGA-D-25-00184_Revision_1 [file giag024_giga-d-25-00184_revision_1.pdf]
