# Supplementary material for: HYMET: a hybrid metagenomic pipeline for accurate and efficient taxonomic classification
Source: Gigascience. 2026 Mar 2;15:giag024. doi: 10.1093/gigascience/giag024 (PMC13042306; doi:10.1093/gigascience/giag024)

GigaScience

HYMET: A Hybrid Metagenomic Pipeline for Accurate and Efficient Taxonomic Classification

--Manuscript Draft--

**Manuscript Number:**	GIGA-D-25-00184R2
**Full Title:**	HYMET: A Hybrid Metagenomic Pipeline for Accurate and Efficient Taxonomic Classification
**Article Type:**	Technical Note
**Funding Information:**	FCT Fundação para a Ciência e a Tecnologia (00127-IEETA) — Not applicable<br>European Commission (101081813) — Not applicable<br>FCCN Fundação para a Computação Científica Nacional (2023.14342.CPCA.A1) — Not applicable
**Abstract:**	Background: Reliable taxonomic classification of metagenomic sequences remains constrained by high mutation rates, fragmented assemblies, and large heterogeneous reference databases. HYMET (Hybrid Metagenomic Tool) was developed to overcome these challenges through a two-stage hybrid design combining adaptive Mash-based screening with Minimap2 alignment and a coverage-weighted Lowest Common Ancestor (LCA) classifier. Its sample-adaptive thresholds and on-the-fly reference database construction enable efficient, domain-agnostic classification while maintaining accuracy across divergent genomes.<br>Results: Across seven CAMI assembly datasets in contig mode, HYMET achieved a mean F1 of 83.89%, with genus-level F1 of 76.75% and species-level F1 of 60.18%, while averaging 115.93 s runtime and a mean peak memory of 6.24 GB. Performance remained stable under mutation rates up to 30% for most domains (F1 ≥ 0.8), with viral sequences showing the expected decline (F1 ≈ 0.5 at 30%). Read and contig inputs produced nearly identical results when sharing reference caches, and real-world datasets confirmed robustness with the human gut metagenome reproduced typical anaerobic profiles, while in the ZymoBIOMICS mock community HYMET recovered all bacterial members; a further ground-truth evaluation on the ZymoBIOMICS Gut Microbiome Standard (D6331) yielded near-perfect genus-level concordance (Pearson r = 0.998, Bray–Curtis = 0.04) across bacteria, fungi, and archaea.<br>Conclusions: HYMET achieves a practical balance of accuracy, efficiency, and scalability for metagenomic classification. Its adaptive candidate selection, alignment-anchored taxonomy, and reproducible reference caching collectively enhance performance across domains. HYMET source code is fully available at https://github.com/ieeta-pt/HYMET.
**Corresponding Author:**	Jorge Miguel Ferreira da Silva<br>Universidade de Aveiro Instituto de Engenharia Eletrónica e Informática de Aveiro<br>Esmoriz, Seleccione um PORTUGAL
**Corresponding Author Secondary Information:**	
**Corresponding Author's Institution:**	Universidade de Aveiro Instituto de Engenharia Eletrónica e Informática de Aveiro
**Corresponding Author's Secondary Institution:**	
**First Author:**	Jorge Miguel Ferreira da Silva
**First Author Secondary Information:**	
**Order of Authors:**	Jorge Miguel Ferreira da Silva<br>Inês Martins, M.D.<br>João Rafael Almeida

Powered by Editorial Manager® and ProduXion Manager® from Aries Systems Corporation

**Order of Authors Secondary Information:**	
**Response to Reviewers:**	HYMET (GIGA-D-25-00184R1) – Response to Reviewers 2nd Review<br>We appreciate the comments, which improved the clarity, completeness, and robustness of our work. We addressed all points through detailed responses, manuscript edits, and enhancements to the HYMET codebase, aligning the description with the tool's capabilities. We believe the manuscript is now ready for publishing.<br><br>Reviewer #1<br>I appreciate the effort the authors had taken on the software, results and manuscript. However, some issues remain.<br><br><br>Response:<br>We addressed all remaining concerns through manuscript revisions and software enhancements, as detailed below.<br><br>Major:<br>Comment:<br>- In the Background, the reason for not benchmarking Taxor and Ganon2 is not convincing, as they are not embedded within CAMITAX nor TAMA, and they were published recently (2024 and 2025). Taxor is designed for long-read classification that is similar to contig classification in HYMET. Ganon2 works mainly for short-read classification. While in the Result section, Ganon2 is compared.<br><br>Response:<br>We agree the previous wording was confusing. We revised the Background and Methods to distinguish benchmarked classifiers from those only discussed. Ganon2 is now included as a stand-alone baseline in the CAMI contig benchmark. Taxor is designed for read-level long-read classification and falls outside the contig-based evaluation scope, so we cite it but do not benchmark it directly.<br>Comment:<br>- The accuracy evaluation in the human gut assembly lacks the ground-truth. "gut microbiome profiles closely matched expected anaerobic bacterial taxa" is too arbitrary.<br>The sequencing data of ZymoBIOMICS Gut Microbiome Standard can be an alternative (https://github.com/Kirk3gaard/MicroBench?tab=readme-ov-file#zymogut).<br><br>Response:<br>We added the ZymoBIOMICS Gut Microbiome Standard (D6331) as a third case study with known ground truth. HYMET achieved near-perfect genus-level classification (Pearson r = 0.998, Bray-Curtis = 0.04) across 534 contigs spanning bacteria, fungi, and archaea. 11 of 14 expected genera were detected; the three missed genera each had ≤1 contig in the assembly. Species-level confusion between sister species (C. albicans/dubliniensis, S. cerevisiae/paradoxus) is a known limitation for organisms with >95% ANI. A new main-text figure and genus-level comparison table are in the revised manuscript.<br><br>Comment:<br>- It's still not easy to run a simple job with HYMET. After the installation with Conda following the docs (readme.md and reproducibility.md), it took me about **1 hour** to make a contig classification job run, with multiple trials even after checking docs and source code. It's highly recommended to ask a colleague to install it from scratch and do some tests, to make sure that the tool could work as expected.<br><br>\`\`\`<br>$ hymet run --contig t.fasta --out . --threads 16<br>[hymet] (/home/xxx/app/miniconda3/envs/hymet/share/hymet) $ /home/xxx/app/miniconda3/envs/hymet/share/hymet/run_hymet_cami.sh<br>ERROR: missing data/detailed_taxonomy.tsv<br><br># \`detailed_taxonomy.tsv\` seems to be generated by make_refset_taxonomy.py.<br><br># resutls_summary.md provides a command to generate it<br>python bench/tools/make_refset_taxonomy.py \\

```
  --fasta bench/refsets/combined_subset.fasta \
  --taxonkit-db taxonomy_files \
  --output data/detailed_taxonomy.tsv

# But later I realised the file would be overwritten, and a file with only the header row would stop the error. So it's a bug.
```

Response:
The missing detailed_taxonomy.tsv was a bug: the pipeline expected the file but did not create it during initialisation. This is now fixed. A new hymet init subcommand handles first-run setup (NCBI taxonomy dumps, hierarchy file, required stubs) so that hymet run works without manual steps. The CLI also now prints clear error messages when prerequisites are missing. We updated the README and Supplementary Material to reflect the new workflow, and had colleagues unfamiliar with the tool test a fresh install end-to-end.

Minor:

Comment:
- Abstract and other places, "reference construction" should be "reference database construction" or "index building".

Response:
Fixed. We now use "reference database construction" instead of "reference construction" throughout.

Comment:
- Abstract and Result: "seven CAMI assemblies" are ambiguous (seven genomes?), it could be "CAMI assembly datasets".

Response:
Fixed. We now use “CAMI assembly datasets” throughout to avoid confusion with individual genome assemblies.
Comment:
- In Table 1 and the response to reviewers, it said RefSeq 88 is used in sketch1.msh (directly downloaded from the Mash project). While in the section "Reference Sketched Database", it's RefSeq Nucleotide Release 228.

Response:
Good catch. The “Release 228” mention referred to the pool for dynamic alignment updates, not the static screening baseline. We revised the Methods to make this explicit: HYMET screens against RefSeq Release 88 sketches, supplemented by custom databases (sketch3.msh) for modern diversity. The text now reads “RefSeq Release 88 (Base Screen) + Custom Augmentation”.
Comment:
- In the context of sketching databases, the term "seed" can be rephrased as "hash seed" to avoid confusion with the "seed sequence" used in alignment tools such as Blast and Minimap2.

Response:
We agree. We replaced "seed" with "hash seed" in the manuscript text, Table 1 caption, and column header.

Comment:
- There are too many figures and tables in the main text, and most of the text is too small to read.

Response:
We agree. We relocated six figures to the Supplementary Material (now Figs S4–S9) and merged related panels into composites (Figs 6 and 7), bringing the main-text count

from 15 to 8. The extra figure (Fig. 8) covers the ZymoGut D6331 ground-truth evaluation. All remaining figures use full-width layouts.

Comment:
- Supplementary Figure 2 and 3: The texts in the figures are hard to read.

Response:
We increased the resolution and display width of Supplementary Figures S1–S3 to full page width. Captions now list the tools and domains shown in each panel.
Comment:
- Each run creates a new cache directory, such as data/downloaded_genomes/cache/51e459af820785d66646fcaab7c6da69688975da, and assembly summary files of GenBank (1.4 GB) and refseq (200 MB) will be repeatedly downloaded to download_cache/. It's unnecessary and would cost a lot of time for a slow internet connection. Even after setting `--cache-root`, it still creates a new cache directory with a new cache key and fails to use the existing cache.

Response:
The root cause was that the cache key included transient path components, so a new cache directory was created every run even with the same candidate set. HYMET now computes a stable cache key from the sorted accession list, so repeated runs reuse the existing cache. The --cache-root flag is properly propagated to all stages. Assembly summary files are also cached locally and only refreshed with --force-download, avoiding the repeated GenBank (1.4 GB) and RefSeq (200 MB) downloads. Documented in the Supplementary Material.
Comment:

- In the abstract and discussion, it is said in the benchmark, HYMET's peak memory usage is ~6.2GB. However, in the benchmark, there are only 922 reference genomes are used. In my test with a contig (with the 3 default skeches databases), the prefiltering step with Mash would consume ~13 GB.

Response:
The ~6.2 GB figure is the mean across the seven CAMI samples. Peak memory is sample-dependent: five low-complexity panels run under 2.3 GB, while CAMI II marine and strain madness reach ~17.4 GB due to larger candidate sets. The reviewer’s ~13 GB observation with all three default sketches is consistent with this. We revised the abstract, key points, and discussion to report 6.2 GB explicitly as a mean and note the sample-dependent range (under 2 GB to ~17 GB).

Reviewer #2

Most of the concerns have been addressed. However, a few minor issues remain:

Response:
We addressed the remaining points as detailed below.
Comment:
There are too many figures (15 in total). It would be better to merge related figures into composite panels to improve readability.

Response:
We agree. We merged related figures into composite panels and reduced the main-text count from 15 to 8 (the additional figure addresses the ZymoGut ground-truth evaluation).
Comment:
The evaluation metrics for each independent benchmark dataset should be clearly presented.

Response:
We now provide per-dataset metrics in Supplementary Table S18, reporting Avg F1, Genus F1, and Species F1 for each of the seven CAMI samples individually. The full per-sample, per-rank breakdown is also versioned in the project repository

	(results/cami/canonical/RUN_0/tables/).<br>Comment:<br>The Results section is still somewhat confusing, despite the good performance. Consider reorganizing it so that each subsection clearly conveys a specific conclusion.<br><br>Response:<br>We reorganized the Results. The CAMI benchmark now leads with classification accuracy and profile quality, followed by Computational Efficiency, Mutation Resilience, input-modality comparison, and real-data case study subsections.<br>Comment:<br>Figure 2 is not referenced in the text and should be properly cited.<br><br>Response:<br>Thank you for catching this. Figure 2 (F1 scores across taxonomic groups at 0% mutation) is now explicitly cited in the CAMI Benchmark subsection of the Results.
**Additional Information:**	
**Question**	**Response**
Are you submitting this manuscript to a special series or article collection?	No
**Experimental design and statistics**<br><br>Full details of the experimental design and statistical methods used should be given in the Methods section, as detailed in our Minimum Standards Reporting Checklist. Information essential to interpreting the data presented should be made available in the figure legends.<br><br>Have you included all the information requested in your manuscript?	Yes
**Resources**<br><br>A description of all resources used, including antibodies, cell lines, animals and software tools, with enough information to allow them to be uniquely identified, should be included in the Methods section. Authors are strongly encouraged to cite Research Resource Identifiers (RRIDs) for antibodies, model organisms and tools, where possible.<br><br>Have you included the information requested as detailed in our Minimum Standards Reporting Checklist?	Yes

Powered by Editorial Manager® and ProduXion Manager® from Aries Systems Corporation

**Availability of data and materials**<br><br>All datasets and code on which the conclusions of the paper rely must be either included in your submission or deposited in publicly available repositories (where available and ethically appropriate), referencing such data using a unique identifier in the references and in the "Availability of Data and Materials" section of your manuscript.<br><br>Have you have met the above requirement as detailed in our Minimum Standards Reporting Checklist?	Yes
GigaScience has policies and guidelines in place for the use of generative AI-writing tools such as ChatGPT. If you have used such writing tools to assist with writing the manuscript this must be declared and cited in the text. Authors should not list AI-writing tools and other AI-assisted technologies as an author or co-author and should acknowledge that they are fully responsible for text generated or refined by AI-writing tools.<p><br><br>A summary of use (particularly in the introduction or among methods) needs to be included at the end of the paper, and the outputs should also be included as a supplementary file hosted in GigaDB or other open repositories. Please read our guidelines for more information. <p><br><br>By submitting to GigaScience, you are aware of the journal's AI-writing tools policy, and if you have declared use of such tools below, you have acknowledged this where appropriate in your manuscript and have made a summary of use and outputs available. <p><br><b>AI-assisted writing tools have been used in the preparation of this manuscript?	No

LaTeX - Main Document (TeX file) Click here to access/download;LaTeX - Main Document (TeX file);main.tex

```
This is pdfTeX, Version 3.141592653-2.6-1.40.26 (TeX Live 2024)
(preloaded format=pdflatex 2024.8.2)  28 FEB 2026 01:39
entering extended mode
 restricted \write18 enabled.
 %&-line parsing enabled.
**main.tex
(./main.tex
LaTeX2e <2024-06-01> patch level 2
L3 programming layer <2024-05-27>
(./oup-contemporary.cls
Document Class: oup-contemporary 2023/06/12, v1.2
(c:/texlive/2024/texmf-dist/tex/latex/base/article.cls
Document Class: article 2024/02/08 v1.4n Standard LaTeX document class
(c:/texlive/2024/texmf-dist/tex/latex/base/size10.clo
File: size10.clo 2024/02/08 v1.4n Standard LaTeX file (size option)
)
\c@part=\count194
\c@section=\count195
\c@subsection=\count196
\c@subsubsection=\count197
\c@paragraph=\count198
\c@subparagraph=\count199
\c@figure=\count266
\c@table=\count267
\abovecaptionskip=\skip49
\belowcaptionskip=\skip50
\bibindent=\dimen141
) (c:/texlive/2024/texmf-dist/tex/latex/base/inputenc.sty
Package: inputenc 2024/02/08 v1.3d Input encoding file
\inpenc@prehook=\toks17
\inpenc@posthook=\toks18
) (c:/texlive/2024/texmf-dist/tex/latex/base/fontenc.sty
Package: fontenc 2021/04/29 v2.0v Standard LaTeX package
) (c:/texlive/2024/texmf-dist/tex/generic/iftex/ifpdf.sty
Package: ifpdf 2019/10/25 v3.4 ifpdf legacy package. Use iftex instead.
(c:/texlive/2024/texmf-dist/tex/generic/iftex/iftex.sty
Package: iftex 2022/02/03 v1.0f TeX engine tests
)) (c:/texlive/2024/texmf-dist/tex/latex/microtype/microtype.sty
Package: microtype 2024/03/29 v3.1b Micro-typographical refinements (RS)
(c:/texlive/2024/texmf-dist/tex/latex/graphics/keyval.sty
Package: keyval 2022/05/29 v1.15 key=value parser (DPC)
\KV@toks@=\toks19
) (c:/texlive/2024/texmf-dist/tex/latex/etoolbox/etoolbox.sty
Package: etoolbox 2020/10/05 v2.5k e-TeX tools for LaTeX (JAW)
\etb@tempcnta=\count268
)
\MT@toks=\toks20
\MT@tempbox=\box52
\MT@count=\count269
LaTeX Info: Redefining \noprotrusionifhmode on input line 1061.
LaTeX Info: Redefining \leftprotrusion on input line 1062.
\MT@prot@toks=\toks21
LaTeX Info: Redefining \rightprotrusion on input line 1081.
LaTeX Info: Redefining \textls on input line 1392.
```

```
\MT@outer@kern=\dimen142
LaTeX Info: Redefining \textmicrotypecontext on input line 2013.
\MT@listname@count=\count270
(c:/texlive/2024/texmf-dist/tex/latex/microtype/microtype-pdftex.def
File: microtype-pdftex.def 2024/03/29 v3.1b Definitions specific to
pdftex (RS)

LaTeX Info: Redefining \lsstyle on input line 902.
LaTeX Info: Redefining \lslig on input line 902.
\MT@outer@space=\skip51
)
Package microtype Info: Loading configuration file microtype.cfg.
(c:/texlive/2024/texmf-dist/tex/latex/microtype/microtype.cfg
File: microtype.cfg 2024/03/29 v3.1b microtype main configuration file
(RS)
)) (c:/texlive/2024/texmf-dist/tex/latex/euler/euler.sty
Package: euler 1995/03/05 v2.5
Package: `euler' v2.5 <1995/03/05> (FJ and FMi)
LaTeX Font Info:    Redeclaring symbol font `letters' on input line 35.
LaTeX Font Info:    Encoding `OML' has changed to `U' for symbol font
(Font)              `letters' in the math version `normal' on input line
35.
LaTeX Font Info:    Overwriting symbol font `letters' in version `normal'
(Font)                  OML/cmm/m/it --> U/eur/m/n on input line 35.
LaTeX Font Info:    Encoding `OML' has changed to `U' for symbol font
(Font)              `letters' in the math version `bold' on input line
35.
LaTeX Font Info:    Overwriting symbol font `letters' in version `bold'
(Font)                  OML/cmm/b/it --> U/eur/m/n on input line 35.
LaTeX Font Info:    Overwriting symbol font `letters' in version `bold'
(Font)                  U/eur/m/n --> U/eur/b/n on input line 36.
LaTeX Font Info:    Redeclaring math symbol \Gamma on input line 47.
LaTeX Font Info:    Redeclaring math symbol \Delta on input line 48.
LaTeX Font Info:    Redeclaring math symbol \Theta on input line 49.
LaTeX Font Info:    Redeclaring math symbol \Lambda on input line 50.
LaTeX Font Info:    Redeclaring math symbol \Xi on input line 51.
LaTeX Font Info:    Redeclaring math symbol \Pi on input line 52.
LaTeX Font Info:    Redeclaring math symbol \Sigma on input line 53.
LaTeX Font Info:    Redeclaring math symbol \Upsilon on input line 54.
LaTeX Font Info:    Redeclaring math symbol \Phi on input line 55.
LaTeX Font Info:    Redeclaring math symbol \Psi on input line 56.
LaTeX Font Info:    Redeclaring math symbol \Omega on input line 57.
\symEulerFraktur=\mathgroup4
LaTeX Font Info:    Overwriting symbol font `EulerFraktur' in version
`bold'
(Font)                  U/euf/m/n --> U/euf/b/n on input line 63.
LaTeX Info: Redefining \oldstylenums on input line 85.
\symEulerScript=\mathgroup5
LaTeX Font Info:    Overwriting symbol font `EulerScript' in version
`bold'
(Font)                  U/eus/m/n --> U/eus/b/n on input line 93.
LaTeX Font Info:    Redeclaring math symbol \aleph on input line 97.
LaTeX Font Info:    Redeclaring math symbol \Re on input line 98.
LaTeX Font Info:    Redeclaring math symbol \Im on input line 99.
```

```
LaTeX Font Info:    Redeclaring math delimiter \vert on input line 101.
LaTeX Font Info:    Redeclaring math delimiter \backslash on input line
103.
LaTeX Font Info:    Redeclaring math symbol \neg on input line 106.
LaTeX Font Info:    Redeclaring math symbol \wedge on input line 108.
LaTeX Font Info:    Redeclaring math symbol \vee on input line 110.
LaTeX Font Info:    Redeclaring math symbol \setminus on input line 112.
LaTeX Font Info:    Redeclaring math symbol \sim on input line 113.
LaTeX Font Info:    Redeclaring math symbol \mid on input line 114.
LaTeX Font Info:    Redeclaring math delimiter \arrowvert on input line
116.
LaTeX Font Info:    Redeclaring math symbol \mathsection on input line
117.
\symEulerExtension=\mathgroup6
LaTeX Font Info:    Redeclaring math symbol \coprod on input line 125.
LaTeX Font Info:    Redeclaring math symbol \prod on input line 125.
LaTeX Font Info:    Redeclaring math symbol \sum on input line 125.
LaTeX Font Info:    Redeclaring math symbol \intop on input line 130.
LaTeX Font Info:    Redeclaring math symbol \ointop on input line 131.
LaTeX Font Info:    Redeclaring math symbol \braceld on input line 132.
LaTeX Font Info:    Redeclaring math symbol \bracerd on input line 133.
LaTeX Font Info:    Redeclaring math symbol \bracelu on input line 134.
LaTeX Font Info:    Redeclaring math symbol \braceru on input line 135.
LaTeX Font Info:    Redeclaring math symbol \infty on input line 136.
LaTeX Font Info:    Redeclaring math symbol \nearrow on input line 153.
LaTeX Font Info:    Redeclaring math symbol \searrow on input line 154.
LaTeX Font Info:    Redeclaring math symbol \nwarrow on input line 155.
LaTeX Font Info:    Redeclaring math symbol \swarrow on input line 156.
LaTeX Font Info:    Redeclaring math symbol \Leftrightarrow on input line
157.
LaTeX Font Info:    Redeclaring math symbol \Leftarrow on input line 158.
LaTeX Font Info:    Redeclaring math symbol \Rightarrow on input line
159.
LaTeX Font Info:    Redeclaring math symbol \leftrightarrow on input line
160.
LaTeX Font Info:    Redeclaring math symbol \leftarrow on input line 161.
LaTeX Font Info:    Redeclaring math symbol \rightarrow on input line
163.
LaTeX Font Info:    Redeclaring math delimiter \uparrow on input line
166.
LaTeX Font Info:    Redeclaring math delimiter \downarrow on input line
168.
LaTeX Font Info:    Redeclaring math delimiter \updownarrow on input line
170.
LaTeX Font Info:    Redeclaring math delimiter \Uparrow on input line
172.
LaTeX Font Info:    Redeclaring math delimiter \Downarrow on input line
174.
LaTeX Font Info:    Redeclaring math delimiter \Updownarrow on input line
176.
LaTeX Font Info:    Redeclaring math symbol \leftharpoonup on input line
177.
LaTeX Font Info:    Redeclaring math symbol \leftharpoondown on input
line 178.
```

```
LaTeX Font Info:    Redeclaring math symbol \rightharpoonup on input line
179.
LaTeX Font Info:    Redeclaring math symbol \rightharpoondown on input
line 180
.
LaTeX Font Info:    Redeclaring math delimiter \lbrace on input line 182.
LaTeX Font Info:    Redeclaring math delimiter \rbrace on input line 184.
\symcmmigroup=\mathgroup7
LaTeX Font Info:    Overwriting symbol font `cmmigroup' in version `bold'
(Font)                  OML/cmm/m/it --> OML/cmm/b/it on input line 200.
LaTeX Font Info:    Redeclaring math accent \vec on input line 201.
LaTeX Font Info:    Redeclaring math symbol \triangleleft on input line
202.
LaTeX Font Info:    Redeclaring math symbol \triangleright on input line
203.
LaTeX Font Info:    Redeclaring math symbol \star on input line 204.
LaTeX Font Info:    Redeclaring math symbol \lhook on input line 205.
LaTeX Font Info:    Redeclaring math symbol \rhook on input line 206.
LaTeX Font Info:    Redeclaring math symbol \flat on input line 207.
LaTeX Font Info:    Redeclaring math symbol \natural on input line 208.
LaTeX Font Info:    Redeclaring math symbol \sharp on input line 209.
LaTeX Font Info:    Redeclaring math symbol \smile on input line 210.
LaTeX Font Info:    Redeclaring math symbol \frown on input line 211.
LaTeX Font Info:    Redeclaring math accent \grave on input line 245.
LaTeX Font Info:    Redeclaring math accent \acute on input line 246.
LaTeX Font Info:    Redeclaring math accent \tilde on input line 247.
LaTeX Font Info:    Redeclaring math accent \ddot on input line 248.
LaTeX Font Info:    Redeclaring math accent \check on input line 249.
LaTeX Font Info:    Redeclaring math accent \breve on input line 250.
LaTeX Font Info:    Redeclaring math accent \bar on input line 251.
LaTeX Font Info:    Redeclaring math accent \dot on input line 252.
LaTeX Font Info:    Redeclaring math accent \hat on input line 254.
) (./merriweather.sty
Package: merriweather 2024/01/01 Stub for compilation
) (c:/texlive/2024/texmf-dist/tex/latex/lm/lmodern.sty
Package: lmodern 2015/05/01 v1.6.1 Latin Modern Fonts
LaTeX Font Info:    Overwriting symbol font `operators' in version
`normal'
(Font)                  OT1/cmr/m/n --> OT1/lmr/m/n on input line 22.
LaTeX Font Info:    Encoding `U' has changed to `OML' for symbol font
(Font)              `letters' in the math version `normal' on input line
23.
LaTeX Font Info:    Overwriting symbol font `letters' in version `normal'
(Font)                  U/eur/m/n --> OML/lmm/m/it on input line 23.
LaTeX Font Info:    Overwriting symbol font `symbols' in version `normal'
(Font)                  OMS/cmsy/m/n --> OMS/lmsy/m/n on input line 24.
LaTeX Font Info:    Overwriting symbol font `largesymbols' in version
`normal'
(Font)                  OMX/cmex/m/n --> OMX/lmex/m/n on input line 25.
LaTeX Font Info:    Overwriting symbol font `operators' in version `bold'
(Font)                  OT1/cmr/bx/n --> OT1/lmr/bx/n on input line 26.
LaTeX Font Info:    Encoding `U' has changed to `OML' for symbol font
```

```
(Font)              `letters' in the math version `bold' on input line
27.
LaTeX Font Info:    Overwriting symbol font `letters' in version `bold'
(Font)                  U/eur/b/n --> OML/lmm/b/it on input line 27.
LaTeX Font Info:    Overwriting symbol font `symbols' in version `bold'
(Font)                  OMS/cmsy/b/n --> OMS/lmsy/b/n on input line 28.
LaTeX Font Info:    Overwriting symbol font `largesymbols' in version
`bold'
(Font)                  OMX/cmex/m/n --> OMX/lmex/m/n on input line 29.
LaTeX Font Info:    Overwriting math alphabet `\mathbf' in version
`normal'
(Font)                  OT1/cmr/bx/n --> OT1/lmr/bx/n on input line 31.
LaTeX Font Info:    Overwriting math alphabet `\mathsf' in version
`normal'
(Font)                  OT1/cmss/m/n --> OT1/lmss/m/n on input line 32.
LaTeX Font Info:    Overwriting math alphabet `\mathit' in version
`normal'
(Font)                  OT1/cmr/m/it --> OT1/lmr/m/it on input line 33.
LaTeX Font Info:    Overwriting math alphabet `\mathtt' in version
`normal'
(Font)                  OT1/cmtt/m/n --> OT1/lmtt/m/n on input line 34.
LaTeX Font Info:    Overwriting math alphabet `\mathbf' in version `bold'
(Font)                  OT1/cmr/bx/n --> OT1/lmr/bx/n on input line 35.
LaTeX Font Info:    Overwriting math alphabet `\mathsf' in version `bold'
(Font)                  OT1/cmss/bx/n --> OT1/lmss/bx/n on input line 36.
LaTeX Font Info:    Overwriting math alphabet `\mathit' in version `bold'
(Font)                  OT1/cmr/bx/it --> OT1/lmr/bx/it on input line 37.
LaTeX Font Info:    Overwriting math alphabet `\mathtt' in version `bold'
(Font)                  OT1/cmtt/m/n --> OT1/lmtt/m/n on input line 38.
) (c:/texlive/2024/texmf-dist/tex/latex/mathastext/mathastext.sty
Package: mathastext 2024/07/27 v1.4b Use the text font in math mode (JFB)

Package mathastext Info: Starting the math mode configuration.
\mst@exists@muskip=\muskip17
\mst@forall@muskip=\muskip18
\mst@prime@muskip=\muskip19
\mst@do@nonletters=\toks22
\mst@undo@nonletters=\toks23
\mst@do@easynonletters=\toks24
\mst@undo@easynonletters=\toks25
\symmtoperatorfont=\mathgroup8
\symmtletterfont=\mathgroup9
(   mathastext:   ) ! and ?
(   mathastext:   ) punctuation: , . : ; and \colon
LaTeX Info: Redefining \relbar on input line 1201.
LaTeX Info: Redefining \rightarrowfill on input line 1202.
LaTeX Info: Redefining \leftarrowfill on input line 1205.
(   mathastext:   ) + and =
LaTeX Info: Redefining \Relbar on input line 1298.
(   mathastext:   ) adding = ; and + to \nfss@catcodes
(   mathastext:   ) parentheses ( ) [ ] and slash /
(   mathastext:   ) alldelims: < > \backslash \setminus | \vert \mid \{
\}
LaTeX Font Info:    Redeclaring math symbol \setminus on input line 1364.
```

```
LaTeX Info: Redefining \models on input line 1383.
(   mathastext:   ) \# \mathdollar \% \&
(   mathastext:   ) \imath and \jmath
LaTeX Font Info:    Overwriting math alphabet `\Mathnormalbold' in
version `nor
mal'
(Font)                  T1/lmr/b/it --> T1/lmr/b/it on input line 2863.
LaTeX Font Info:    Overwriting math alphabet `\Mathnormalbold' in
version `bol
d'
(Font)                  T1/lmr/b/it --> T1/lmr/b/it on input line 2863.
LaTeX Font Info:    Overwriting symbol font `mtletterfont' in version
`normal'
(Font)                  T1/lmr/m/it --> T1/lmr/m/it on input line 2863.
LaTeX Font Info:    Overwriting symbol font `mtletterfont' in version
`bold'
(Font)                  T1/lmr/m/it --> T1/lmr/b/it on input line 2863.
LaTeX Font Info:    Overwriting symbol font `mtoperatorfont' in version
`normal
'
(Font)                  T1/lmr/m/n --> T1/lmr/m/n on input line 2863.
LaTeX Font Info:    Overwriting symbol font `mtoperatorfont' in version
`bold'
(Font)                  T1/lmr/m/n --> T1/lmr/b/n on input line 2863.
LaTeX Font Info:    Overwriting math alphabet `\Mathbf' in version
`normal'
(Font)                  T1/lmr/b/n --> T1/lmr/b/n on input line 2863.
LaTeX Font Info:    Overwriting math alphabet `\Mathbf' in version `bold'
(Font)                  T1/lmr/b/n --> T1/lmr/b/n on input line 2863.
LaTeX Font Info:    Overwriting math alphabet `\Mathit' in version
`normal'
(Font)                  T1/lmr/m/it --> T1/lmr/m/it on input line 2863.
LaTeX Font Info:    Overwriting math alphabet `\Mathit' in version `bold'
(Font)                  T1/lmr/m/it --> T1/lmr/b/it on input line 2863.
LaTeX Font Info:    Overwriting math alphabet `\Mathsf' in version
`normal'
(Font)                  T1/lmss/m/n --> T1/lmss/m/n on input line 2863.
LaTeX Font Info:    Overwriting math alphabet `\Mathsf' in version `bold'
(Font)                  T1/lmss/m/n --> T1/lmss/b/n on input line 2863.
LaTeX Font Info:    Overwriting math alphabet `\Mathtt' in version
`normal'
(Font)                  T1/lmtt/m/n --> T1/lmtt/m/n on input line 2863.
LaTeX Font Info:    Overwriting math alphabet `\Mathtt' in version `bold'
(Font)                  T1/lmtt/m/n --> T1/lmtt/b/n on input line 2863.
(   mathastext:   ) Latin letters in the `normal', resp. `bold',
(   mathastext:   ) math versions are now set up to use the fonts
(   mathastext:   ) T1/lmr/m/it, resp. T1/lmr/b/it.
(   mathastext:   ) Other characters (digits, ...) and \log-like names
will be
(   mathastext:   ) typeset with the n shape.
(   mathastext:   ) \hbar
(   mathastext:   ) minus as endash
(   mathastext:   ) The italic option is in effect.
(   mathastext:   ) \HUGE has been (re)-defined.
```

```
(   mathastext:   ) mathastext has declared larger sizes for subscripts.
(   mathastext:   ) To keep LaTeX defaults, use option
`defaultmathsizes'.

Package mathastext Info: Loading is complete.  You can now use
\Mathastext to
(mathastext)             modify the normal and bold math versions.  Use
it
(mathastext)             with optional argument or use \MTDeclareVersion
to
(mathastext)             declare additional math versions.
) (c:/texlive/2024/texmf-dist/tex/latex/relsize/relsize.sty
Package: relsize 2013/03/29 ver 4.1
) (c:/texlive/2024/texmf-dist/tex/latex/ragged2e/ragged2e.sty
Package: ragged2e 2023/06/22 v3.6 ragged2e Package
\CenteringLeftskip=\skip52
\RaggedLeftLeftskip=\skip53
\RaggedRightLeftskip=\skip54
\CenteringRightskip=\skip55
\RaggedLeftRightskip=\skip56
\RaggedRightRightskip=\skip57
\CenteringParfillskip=\skip58
\RaggedLeftParfillskip=\skip59
\RaggedRightParfillskip=\skip60
\JustifyingParfillskip=\skip61
\CenteringParindent=\skip62
\RaggedLeftParindent=\skip63
\RaggedRightParindent=\skip64
\JustifyingParindent=\skip65
) (c:/texlive/2024/texmf-dist/tex/latex/xcolor/xcolor.sty
Package: xcolor 2023/11/15 v3.01 LaTeX color extensions (UK)
(c:/texlive/2024/texmf-dist/tex/latex/graphics-cfg/color.cfg
File: color.cfg 2016/01/02 v1.6 sample color configuration
)
Package xcolor Info: Driver file: pdftex.def on input line 274.
(c:/texlive/2024/texmf-dist/tex/latex/graphics-def/pdftex.def
File: pdftex.def 2024/04/13 v1.2c Graphics/color driver for pdftex
) (c:/texlive/2024/texmf-dist/tex/latex/graphics/mathcolor.ltx)
Package xcolor Info: Model `cmy' substituted by `cmy0' on input line
1350.
Package xcolor Info: Model `hsb' substituted by `rgb' on input line 1354.
Package xcolor Info: Model `RGB' extended on input line 1366.
Package xcolor Info: Model `HTML' substituted by `rgb' on input line
1368.
Package xcolor Info: Model `Hsb' substituted by `hsb' on input line 1369.
Package xcolor Info: Model `tHsb' substituted by `hsb' on input line
1370.
Package xcolor Info: Model `HSB' substituted by `hsb' on input line 1371.
Package xcolor Info: Model `Gray' substituted by `gray' on input line
1372.
Package xcolor Info: Model `wave' substituted by `hsb' on input line
1373.
) (c:/texlive/2024/texmf-dist/tex/latex/colortbl/colortbl.sty
Package: colortbl 2024/07/06 v1.0i Color table columns (DPC)
```

```
(c:/texlive/2024/texmf-dist/tex/latex/tools/array.sty
Package: array 2024/06/14 v2.6d Tabular extension package (FMi)
\col@sep=\dimen143
\ar@mcellbox=\box53
\extrarowheight=\dimen144
\NC@list=\toks26
\extratabsurround=\skip66
\backup@length=\skip67
\ar@cellbox=\box54
)
\everycr=\toks27
\minrowclearance=\skip68
\rownum=\count271
) (c:/texlive/2024/texmf-dist/tex/latex/graphics/graphicx.sty
Package: graphicx 2021/09/16 v1.2d Enhanced LaTeX Graphics (DPC,SPQR)
(c:/texlive/2024/texmf-dist/tex/latex/graphics/graphics.sty
Package: graphics 2024/05/23 v1.4g Standard LaTeX Graphics (DPC,SPQR)
(c:/texlive/2024/texmf-dist/tex/latex/graphics/trig.sty
Package: trig 2023/12/02 v1.11 sin cos tan (DPC)
) (c:/texlive/2024/texmf-dist/tex/latex/graphics-cfg/graphics.cfg
File: graphics.cfg 2016/06/04 v1.11 sample graphics configuration
)
Package graphics Info: Driver file: pdftex.def on input line 106.
)
\Gin@req@height=\dimen145
\Gin@req@width=\dimen146
) (c:/texlive/2024/texmf-dist/tex/latex/xpatch/xpatch.sty
(c:/texlive/2024/texm
f-dist/tex/latex/l3kernel/expl3.sty
Package: expl3 2024-05-27 L3 programming layer (loader)
(c:/texlive/2024/texmf-dist/tex/latex/l3backend/l3backend-pdftex.def
File: l3backend-pdftex.def 2024-05-08 L3 backend support: PDF output
(pdfTeX)
\l__color_backend_stack_int=\count272
\l__pdf_internal_box=\box55
))
Package: xpatch 2020/03/25 v0.3a Extending etoolbox patching commands
(c:/texlive/2024/texmf-dist/tex/latex/l3packages/xparse/xparse.sty
Package: xparse 2024-05-08 L3 Experimental document command parser
)) (c:/texlive/2024/texmf-dist/tex/latex/environ/environ.sty
Package: environ 2014/05/04 v0.3 A new way to define environments
(c:/texlive/2024/texmf-dist/tex/latex/trimspaces/trimspaces.sty
Package: trimspaces 2009/09/17 v1.1 Trim spaces around a token list
)
\@envbody=\toks28
) (c:/texlive/2024/texmf-dist/tex/latex/lastpage/lastpage.sty
Package: lastpage 2024/07/07 v2.1c lastpage: 2.09 or 2e? (HMM)
(c:/texlive/2024/texmf-dist/tex/latex/lastpage/lastpage2e.sty
Package: lastpage2e 2024/07/07 v2.1c Decide which 2e lastpage version to
use (H
MM)
(c:/texlive/2024/texmf-dist/tex/latex/lastpage/lastpagemodern.sty
Package: lastpagemodern 2024-07-07 v2.1c Refers to last page's name (HMM;
JPG)
```

```
\c@lastpagecount=\count273
)
)) (c:/texlive/2024/texmf-dist/tex/latex/graphics/rotating.sty
Package: rotating 2016/08/11 v2.16d rotated objects in LaTeX
(c:/texlive/2024/texmf-dist/tex/latex/base/ifthen.sty
Package: ifthen 2024/03/16 v1.1e Standard LaTeX ifthen package (DPC)
)
\c@r@tfl@t=\count274
\rotFPtop=\skip69
\rotFPbot=\skip70
\rot@float@box=\box56
\rot@mess@toks=\toks29
) (c:/texlive/2024/texmf-dist/tex/latex/graphics/lscape.sty
Package: lscape 2020/05/28 v3.02 Landscape Pages (DPC)
) (c:/texlive/2024/texmf-dist/tex/latex/tools/afterpage.sty
Package: afterpage 2023/07/04 v1.08 After-Page Package (DPC)
\AP@output=\toks30
\AP@partial=\box57
\AP@footins=\box58
) (c:/texlive/2024/texmf-dist/tex/latex/textpos/textpos.sty
Package: textpos 2022/07/23 v1.10.1
Package textpos Info: choosing support for LaTeX3 on input line 60.
\TP@textbox=\box59
\TP@holdbox=\box60
\TPHorizModule=\dimen147
\TPVertModule=\dimen148
\TP@margin=\dimen149
\TP@absmargin=\dimen150
Grid set 16 x 16 = 37.34424pt x 52.81541pt
\TPboxrulesize=\dimen151
\TP@ox=\dimen152
\TP@oy=\dimen153
\TP@tbargs=\toks31
TextBlockOrigin set to 0pt x 0pt
) (c:/texlive/2024/texmf-dist/tex/latex/url/url.sty
\Urlmuskip=\muskip20
Package: url 2013/09/16  ver 3.4  Verb mode for urls, etc.
) (c:/texlive/2024/texmf-dist/tex/latex/newfloat/newfloat.sty
Package: newfloat 2023/10/01 v1.2 Defining new floating environments (AR)
Package newfloat Info: `rotating' package detected.
) (c:/texlive/2024/texmf-dist/tex/latex/mdframed/mdframed.sty
Package: mdframed 2013/07/01 1.9b: mdframed
(c:/texlive/2024/texmf-dist/tex/latex/kvoptions/kvoptions.sty
Package: kvoptions 2022-06-15 v3.15 Key value format for package options
(HO)
(c:/texlive/2024/texmf-dist/tex/generic/ltxcmds/ltxcmds.sty
Package: ltxcmds 2023-12-04 v1.26 LaTeX kernel commands for general use
(HO)
) (c:/texlive/2024/texmf-dist/tex/latex/kvsetkeys/kvsetkeys.sty
Package: kvsetkeys 2022-10-05 v1.19 Key value parser (HO)
)) (c:/texlive/2024/texmf-dist/tex/latex/zref/zref-abspage.sty
Package: zref-abspage 2023-09-14 v2.35 Module abspage for zref (HO)
(c:/texlive/2024/texmf-dist/tex/latex/zref/zref-base.sty
Package: zref-base 2023-09-14 v2.35 Module base for zref (HO)
```

```
(c:/texlive/2024/texmf-dist/tex/generic/infwarerr/infwarerr.sty
Package: infwarerr 2019/12/03 v1.5 Providing info/warning/error messages
(HO)
) (c:/texlive/2024/texmf-dist/tex/generic/kvdefinekeys/kvdefinekeys.sty
Package: kvdefinekeys 2019-12-19 v1.6 Define keys (HO)
) (c:/texlive/2024/texmf-dist/tex/generic/pdftexcmds/pdftexcmds.sty
Package: pdftexcmds 2020-06-27 v0.33 Utility functions of pdfTeX for
LuaTeX (HO
)
Package pdftexcmds Info: \pdf@primitive is available.
Package pdftexcmds Info: \pdf@ifprimitive is available.
Package pdftexcmds Info: \pdfdraftmode found.
) (c:/texlive/2024/texmf-dist/tex/generic/etexcmds/etexcmds.sty
Package: etexcmds 2019/12/15 v1.7 Avoid name clashes with e-TeX commands
(HO)
) (c:/texlive/2024/texmf-dist/tex/latex/auxhook/auxhook.sty
Package: auxhook 2019-12-17 v1.6 Hooks for auxiliary files (HO)
)
Package zref Info: New property list: main on input line 767.
Package zref Info: New property: default on input line 768.
Package zref Info: New property: page on input line 769.
)
\c@abspage=\count275
Package zref Info: New property: abspage on input line 67.
) (c:/texlive/2024/texmf-dist/tex/latex/needspace/needspace.sty
Package: needspace 2010/09/12 v1.3d reserve vertical space
)
\mdf@templength=\skip71
\c@mdf@globalstyle@cnt=\count276
\mdf@skipabove@length=\skip72
\mdf@skipbelow@length=\skip73
\mdf@leftmargin@length=\skip74
\mdf@rightmargin@length=\skip75
\mdf@innerleftmargin@length=\skip76
\mdf@innerrightmargin@length=\skip77
\mdf@innertopmargin@length=\skip78
\mdf@innerbottommargin@length=\skip79
\mdf@splittopskip@length=\skip80
\mdf@splitbottomskip@length=\skip81
\mdf@outermargin@length=\skip82
\mdf@innermargin@length=\skip83
\mdf@linewidth@length=\skip84
\mdf@innerlinewidth@length=\skip85
\mdf@middlelinewidth@length=\skip86
\mdf@outerlinewidth@length=\skip87
\mdf@roundcorner@length=\skip88
\mdf@footenotedistance@length=\skip89
\mdf@userdefinedwidth@length=\skip90
\mdf@needspace@length=\skip91
\mdf@frametitleaboveskip@length=\skip92
\mdf@frametitlebelowskip@length=\skip93
\mdf@frametitlerulewidth@length=\skip94
\mdf@frametitleleftmargin@length=\skip95
\mdf@frametitlerightmargin@length=\skip96
```

```
\mdf@shadowsize@length=\skip97
\mdf@extratopheight@length=\skip98
\mdf@subtitleabovelinewidth@length=\skip99
\mdf@subtitlebelowlinewidth@length=\skip100
\mdf@subtitleaboveskip@length=\skip101
\mdf@subtitlebelowskip@length=\skip102
\mdf@subtitleinneraboveskip@length=\skip103
\mdf@subtitleinnerbelowskip@length=\skip104
\mdf@subsubtitleabovelinewidth@length=\skip105
\mdf@subsubtitlebelowlinewidth@length=\skip106
\mdf@subsubtitleaboveskip@length=\skip107
\mdf@subsubtitlebelowskip@length=\skip108
\mdf@subsubtitleinneraboveskip@length=\skip109
\mdf@subsubtitleinnerbelowskip@length=\skip110
(c:/texlive/2024/texmf-dist/tex/latex/mdframed/md-frame-0.mdf
File: md-frame-0.mdf 2013/07/01\ 1.9b: md-frame-0
)
\mdf@frametitlebox=\box61
\mdf@footnotebox=\box62
\mdf@splitbox@one=\box63
\mdf@splitbox@two=\box64
\mdf@splitbox@save=\box65
\mdfsplitboxwidth=\skip111
\mdfsplitboxtotalwidth=\skip112
\mdfsplitboxheight=\skip113
\mdfsplitboxdepth=\skip114
\mdfsplitboxtotalheight=\skip115
\mdfframetitleboxwidth=\skip116
\mdfframetitleboxtotalwidth=\skip117
\mdfframetitleboxheight=\skip118
\mdfframetitleboxdepth=\skip119
\mdfframetitleboxtotalheight=\skip120
\mdffootnoteboxwidth=\skip121
\mdffootnoteboxtotalwidth=\skip122
\mdffootnoteboxheight=\skip123
\mdffootnoteboxdepth=\skip124
\mdffootnoteboxtotalheight=\skip125
\mdftotallinewidth=\skip126
\mdfboundingboxwidth=\skip127
\mdfboundingboxtotalwidth=\skip128
\mdfboundingboxheight=\skip129
\mdfboundingboxdepth=\skip130
\mdfboundingboxtotalheight=\skip131
\mdf@freevspace@length=\skip132
\mdf@horizontalwidthofbox@length=\skip133
\mdf@verticalmarginwhole@length=\skip134
\mdf@horizontalspaceofbox=\skip135
\mdfsubtitleheight=\skip136
\mdfsubsubtitleheight=\skip137
\c@mdfcountframes=\count277

****** mdframed patching \endmdf@trivlist

****** -- success******
```

```
\mdf@envdepth=\count278
\c@mdf@env@i=\count279
\c@mdf@env@ii=\count280
\c@mdf@zref@counter=\count281
Package zref Info: New property: mdf@pagevalue on input line 895.
) (c:/texlive/2024/texmf-dist/tex/latex/titlesec/titlesec.sty
Package: titlesec 2023/10/27 v2.16 Sectioning titles
\ttl@box=\box66
\beforetitleunit=\skip138
\aftertitleunit=\skip139
\ttl@plus=\dimen154
\ttl@minus=\dimen155
\ttl@toksa=\toks32
\titlewidth=\dimen156
\titlewidthlast=\dimen157
\titlewidthfirst=\dimen158
) (c:/texlive/2024/texmf-dist/tex/latex/koma-script/scrextend.sty
Package: scrextend 2023/07/07 v3.41 KOMA-Script package (extend other
classes w
ith features of KOMA-Script classes)
(c:/texlive/2024/texmf-dist/tex/latex/koma-script/scrkbase.sty
Package: scrkbase 2023/07/07 v3.41 KOMA-Script package (KOMA-Script-
dependent b
asics and keyval usage)
(c:/texlive/2024/texmf-dist/tex/latex/koma-script/scrbase.sty
Package: scrbase 2023/07/07 v3.41 KOMA-Script package (KOMA-Script-
independent
basics and keyval usage)
(c:/texlive/2024/texmf-dist/tex/latex/koma-script/scrlfile.sty
Package: scrlfile 2023/07/07 v3.41 KOMA-Script package (file load hooks)
(c:/texlive/2024/texmf-dist/tex/latex/koma-script/scrlfile-hook.sty
Package: scrlfile-hook 2023/07/07 v3.41 KOMA-Script package (using LaTeX
hooks)

(c:/texlive/2024/texmf-dist/tex/latex/koma-script/scrlogo.sty
Package: scrlogo 2023/07/07 v3.41 KOMA-Script package (logo)
)))
Applying: [2021/05/01] Usage of raw or classic option list on input line
252.
Already applied: [0000/00/00] Usage of raw or classic option list on
input line
 368.
))
Package scrextend Info: unexpected definition of `\@makefnmark'.
(scrextend)             Trying to patch it on input line 1762.
Package scrextend Info: patch seems to be successfull on input line 1762.
)

LaTeX Font Warning: Font shape `T1/cmr/m/n' in size <7.5> not available
(Font)              size <7> substituted on input line 69.

(c:/texlive/2024/texmf-dist/tex/latex/tools/calc.sty
Package: calc 2023/07/08 v4.3 Infix arithmetic (KKT,FJ)
```

```
\calc@Acount=\count282
\calc@Bcount=\count283
\calc@Adimen=\dimen159
\calc@Bdimen=\dimen160
\calc@Askip=\skip140
\calc@Bskip=\skip141
LaTeX Info: Redefining \setlength on input line 80.
LaTeX Info: Redefining \addtolength on input line 81.
\calc@Ccount=\count284
\calc@Cskip=\skip142
) (c:/texlive/2024/texmf-dist/tex/latex/geometry/geometry.sty
Package: geometry 2020/01/02 v5.9 Page Geometry
(c:/texlive/2024/texmf-dist/tex/generic/iftex/ifvtex.sty
Package: ifvtex 2019/10/25 v1.7 ifvtex legacy package. Use iftex instead.
)
\Gm@cnth=\count285
\Gm@cntv=\count286
\c@Gm@tempcnt=\count287
\Gm@bindingoffset=\dimen161
\Gm@wd@mp=\dimen162
\Gm@odd@mp=\dimen163
\Gm@even@mp=\dimen164
\Gm@layoutwidth=\dimen165
\Gm@layoutheight=\dimen166
\Gm@layouthoffset=\dimen167
\Gm@layoutvoffset=\dimen168
\Gm@dimlist=\toks33
) (c:/texlive/2024/texmf-dist/tex/latex/preprint/authblk.sty
Package: authblk 2001/02/27 1.3 (PWD)
\affilsep=\skip143
\@affilsep=\skip144
\c@Maxaffil=\count288
\c@authors=\count289
\c@affil=\count290
) (c:/texlive/2024/texmf-dist/tex/latex/footmisc/footmisc.sty
Package: footmisc 2023/07/05 v6.0f a miscellany of footnote facilities
\FN@temptoken=\toks34
\footnotemargin=\dimen169
\@outputbox@depth=\dimen170
Package footmisc Info: Declaring symbol style bringhurst on input line
696.
Package footmisc Info: Declaring symbol style chicago on input line 704.
Package footmisc Info: Declaring symbol style wiley on input line 713.
Package footmisc Info: Declaring symbol style lamport-robust on input
line 724.

Package footmisc Info: Declaring symbol style lamport* on input line 744.
Package footmisc Info: Declaring symbol style lamport*-robust on input
line 765
.
) (c:/texlive/2024/texmf-dist/tex/latex/fancyhdr/fancyhdr.sty
Package: fancyhdr 2024/07/23 v4.3.1 Extensive control of page headers and
foote
rs
```

```
\f@nch@headwidth=\skip145
\f@nch@O@elh=\skip146
\f@nch@O@erh=\skip147
\f@nch@O@olh=\skip148
\f@nch@O@orh=\skip149
\f@nch@O@elf=\skip150
\f@nch@O@erf=\skip151
\f@nch@O@olf=\skip152
\f@nch@O@orf=\skip153
) (c:/texlive/2024/texmf-dist/tex/generic/alphalph/alphalph.sty
Package: alphalph 2019/12/09 v2.6 Convert numbers to letters (HO)
(c:/texlive/2024/texmf-dist/tex/generic/intcalc/intcalc.sty
Package: intcalc 2019/12/15 v1.3 Expandable calculations with integers
(HO)
))
\c@authorfn=\count291
(c:/texlive/2024/texmf-dist/tex/latex/abstract/abstract.sty
Package: abstract 2009/06/08 v1.2a configurable abstracts
\abstitleskip=\skip154
\absleftindent=\skip155
\absrightindent=\skip156
\absparindent=\skip157
\absparsep=\skip158
)
Package newfloat Info: New float `keypoints' with options
`placement=t!,name=kp
t' on input line 291.
\c@keypoints=\count292
\newfloat@ftype=\count293
Package newfloat Info: float type `keypoints'=8 on input line 291.
(c:/texlive/2024/texmf-dist/tex/latex/enumitem/enumitem.sty
Package: enumitem 2019/06/20 v3.9 Customized lists
\labelindent=\skip159
\enit@outerparindent=\dimen171
\enit@toks=\toks35
\enit@inbox=\box67
\enit@count@id=\count294
\enitdp@description=\count295
) (c:/texlive/2024/texmf-dist/tex/latex/quoting/quoting.sty
Package: quoting 2014/01/28 v0.1c Consolidated environment for displayed
text
\quo@toppartop=\skip160
) (c:/texlive/2024/texmf-dist/tex/latex/sttools/stfloats.sty
Package: stfloats 2017/03/27 v3.3 Improve float mechanism and
baselineskip sett
ings
\@dblbotnum=\count296
\c@dblbotnumber=\count297
) (c:/texlive/2024/texmf-dist/tex/latex/booktabs/booktabs.sty
Package: booktabs 2020/01/12 v1.61803398 Publication quality tables
\heavyrulewidth=\dimen172
\lightrulewidth=\dimen173
\cmidrulewidth=\dimen174
\belowrulesep=\dimen175
```

```
\belowbottomsep=\dimen176
\aboverulesep=\dimen177
\abovetopsep=\dimen178
\cmidrulesep=\dimen179
\cmidrulekern=\dimen180
\defaultaddspace=\dimen181
\@cmidla=\count298
\@cmidlb=\count299
\@aboverulesep=\dimen182
\@belowrulesep=\dimen183
\@thisruleclass=\count300
\@lastruleclass=\count301
\@thisrulewidth=\dimen184
) (c:/texlive/2024/texmf-dist/tex/latex/tools/tabularx.sty
Package: tabularx 2023/12/11 v2.12a `tabularx' package (DPC)
\TX@col@width=\dimen185
\TX@old@table=\dimen186
\TX@old@col=\dimen187
\TX@target=\dimen188
\TX@delta=\dimen189
\TX@cols=\count302
\TX@ftn=\toks36
)
\enitdp@tablenotes=\count303
(c:/texlive/2024/texmf-dist/tex/latex/caption/caption.sty
Package: caption 2023/08/05 v3.6o Customizing captions (AR)
(c:/texlive/2024/texmf-dist/tex/latex/caption/caption3.sty
Package: caption3 2023/07/31 v2.4d caption3 kernel (AR)
\caption@tempdima=\dimen190
\captionmargin=\dimen191
\caption@leftmargin=\dimen192
\caption@rightmargin=\dimen193
\caption@width=\dimen194
\caption@indent=\dimen195
\caption@parindent=\dimen196
\caption@hangindent=\dimen197
Package caption Info: Standard document class detected.
)
\c@caption@flags=\count304
\c@continuedfloat=\count305
Package caption Info: rotating package is loaded.
Package caption Info: scrextend package is loaded.
\caption@addmargin@hsize=\dimen198
\caption@addmargin@linewidth=\dimen199
) (c:/texlive/2024/texmf-dist/tex/latex/natbib/natbib.sty
Package: natbib 2010/09/13 8.31b (PWD, AO)
\bibhang=\skip161
\bibsep=\skip162
LaTeX Info: Redefining \cite on input line 694.
\c@NAT@ctr=\count306
))
   defining Unicode char U+2009 (decimal 8201)
   defining Unicode char U+2264 (decimal 8804)
   defining Unicode char U+2265 (decimal 8805)
```

```
   defining Unicode char U+2248 (decimal 8776)
   defining Unicode char U+2011 (decimal 8209)
   defining Unicode char U+2013 (decimal 8211)
   defining Unicode char U+2014 (decimal 8212)
   defining Unicode char U+2019 (decimal 8217)
(./siunitx.sty
Package: siunitx 2024/01/01 Stub for compilation
) (c:/texlive/2024/texmf-dist/tex/latex/tools/xspace.sty
Package: xspace 2014/10/28 v1.13 Space after command names (DPC,MH)
) (c:/texlive/2024/texmf-dist/tex/latex/float/float.sty
Package: float 2001/11/08 v1.3d Float enhancements (AL)
\c@float@type=\count307
\float@exts=\toks37
\float@box=\box68
\@float@everytoks=\toks38
\@floatcapt=\box69
) (c:/texlive/2024/texmf-dist/tex/latex/placeins/placeins.sty
Package: placeins 2005/04/18  v 2.2
) (c:/texlive/2024/texmf-dist/tex/latex/hyperref/hyperref.sty
Package: hyperref 2024-07-10 v7.01j Hypertext links for LaTeX
(c:/texlive/2024/texmf-dist/tex/generic/pdfescape/pdfescape.sty
Package: pdfescape 2019/12/09 v1.15 Implements pdfTeX's escape features
(HO)
) (c:/texlive/2024/texmf-dist/tex/latex/hycolor/hycolor.sty
Package: hycolor 2020-01-27 v1.10 Color options for hyperref/bookmark
(HO)
) (c:/texlive/2024/texmf-dist/tex/latex/hyperref/nameref.sty
Package: nameref 2023-11-26 v2.56 Cross-referencing by name of section
(c:/texlive/2024/texmf-dist/tex/latex/refcount/refcount.sty
Package: refcount 2019/12/15 v3.6 Data extraction from label references
(HO)
) (c:/texlive/2024/texmf-
dist/tex/generic/gettitlestring/gettitlestring.sty
Package: gettitlestring 2019/12/15 v1.6 Cleanup title references (HO)
)
\c@section@level=\count308
) (c:/texlive/2024/texmf-dist/tex/generic/stringenc/stringenc.sty
Package: stringenc 2019/11/29 v1.12 Convert strings between diff.
encodings (HO
)
)
\@linkdim=\dimen256
\Hy@linkcounter=\count309
\Hy@pagecounter=\count310
(c:/texlive/2024/texmf-dist/tex/latex/hyperref/pd1enc.def
File: pd1enc.def 2024-07-10 v7.01j Hyperref: PDFDocEncoding definition
(HO)
Now handling font encoding PD1 ...
... no UTF-8 mapping file for font encoding PD1
)
\Hy@SavedSpaceFactor=\count311
(c:/texlive/2024/texmf-dist/tex/latex/hyperref/puenc.def
File: puenc.def 2024-07-10 v7.01j Hyperref: PDF Unicode definition (HO)
Now handling font encoding PU ...
```

```
... no UTF-8 mapping file for font encoding PU
)
Package hyperref Info: Option `colorlinks' set `true' on input line 4040.
Package hyperref Info: Hyper figures OFF on input line 4157.
Package hyperref Info: Link nesting OFF on input line 4162.
Package hyperref Info: Hyper index ON on input line 4165.
Package hyperref Info: Plain pages OFF on input line 4172.
Package hyperref Info: Backreferencing OFF on input line 4177.
Package hyperref Info: Implicit mode ON; LaTeX internals redefined.
Package hyperref Info: Bookmarks ON on input line 4424.
\c@Hy@tempcnt=\count312
LaTeX Info: Redefining \url on input line 4763.
\XeTeXLinkMargin=\dimen257
(c:/texlive/2024/texmf-dist/tex/generic/bitset/bitset.sty
Package: bitset 2019/12/09 v1.3 Handle bit-vector datatype (HO)
(c:/texlive/2024/texmf-dist/tex/generic/bigintcalc/bigintcalc.sty
Package: bigintcalc 2019/12/15 v1.5 Expandable calculations on big
integers (HO
)
))
\Fld@menulength=\count313
\Field@Width=\dimen258
\Fld@charsize=\dimen259
Package hyperref Info: Hyper figures OFF on input line 6042.
Package hyperref Info: Link nesting OFF on input line 6047.
Package hyperref Info: Hyper index ON on input line 6050.
Package hyperref Info: backreferencing OFF on input line 6057.
Package hyperref Info: Link coloring ON on input line 6060.
Package hyperref Info: Link coloring with OCG OFF on input line 6067.
Package hyperref Info: PDF/A mode OFF on input line 6072.
(c:/texlive/2024/texmf-dist/tex/latex/base/atbegshi-ltx.sty
Package: atbegshi-ltx 2021/01/10 v1.0c Emulation of the original atbegshi
package with kernel methods
)
\Hy@abspage=\count314
\c@Item=\count315
\c@Hfootnote=\count316
)
Package hyperref Info: Driver (autodetected): hpdftex.
(c:/texlive/2024/texmf-dist/tex/latex/hyperref/hpdftex.def
File: hpdftex.def 2024-07-10 v7.01j Hyperref driver for pdfTeX
(c:/texlive/2024/texmf-dist/tex/latex/base/atveryend-ltx.sty
Package: atveryend-ltx 2020/08/19 v1.0a Emulation of the original
atveryend pac
kage
with kernel methods
)
\HyAnn@Count=\count317
\Fld@listcount=\count318
\c@bookmark@seq@number=\count319
(c:/texlive/2024/texmf-dist/tex/latex/rerunfilecheck/rerunfilecheck.sty
Package: rerunfilecheck 2022-07-10 v1.10 Rerun checks for auxiliary files
(HO)
(c:/texlive/2024/texmf-dist/tex/generic/uniquecounter/uniquecounter.sty
```

```
Package: uniquecounter 2019/12/15 v1.4 Provide unlimited unique counter
(HO)
)
Package uniquecounter Info: New unique counter `rerunfilecheck' on input
line 2
85.
)
\Hy@SectionHShift=\skip163
)
LaTeX Font Info:    Trying to load font information for T1+lmr on input
line 92
.
(c:/texlive/2024/texmf-dist/tex/latex/lm/t1lmr.fd
File: t1lmr.fd 2015/05/01 v1.6.1 Font defs for Latin Modern
) (./main.aux)
\openout1 = `main.aux'.

LaTeX Font Info:    Checking defaults for OML/cmm/m/it on input line 92.
LaTeX Font Info:    ... okay on input line 92.
LaTeX Font Info:    Checking defaults for OMS/cmsy/m/n on input line 92.
LaTeX Font Info:    ... okay on input line 92.
LaTeX Font Info:    Checking defaults for OT1/cmr/m/n on input line 92.
LaTeX Font Info:    ... okay on input line 92.
LaTeX Font Info:    Checking defaults for T1/cmr/m/n on input line 92.
LaTeX Font Info:    ... okay on input line 92.
LaTeX Font Info:    Checking defaults for TS1/cmr/m/n on input line 92.
LaTeX Font Info:    ... okay on input line 92.
LaTeX Font Info:    Checking defaults for OMX/cmex/m/n on input line 92.
LaTeX Font Info:    ... okay on input line 92.
LaTeX Font Info:    Checking defaults for U/cmr/m/n on input line 92.
LaTeX Font Info:    ... okay on input line 92.
LaTeX Font Info:    Checking defaults for PD1/pdf/m/n on input line 92.
LaTeX Font Info:    ... okay on input line 92.
LaTeX Font Info:    Checking defaults for PU/pdf/m/n on input line 92.
LaTeX Font Info:    ... okay on input line 92.
LaTeX Info: Redefining \microtypecontext on input line 92.
Package microtype Info: Applying patch `item' on input line 92.
Package microtype Info: Applying patch `toc' on input line 92.
Package microtype Info: Applying patch `eqnum' on input line 92.
Package microtype Info: Applying patch `footnote' on input line 92.
Package microtype Info: Applying patch `verbatim' on input line 92.
Package microtype Info: Generating PDF output.
Package microtype Info: Character protrusion enabled (level 2).
Package microtype Info: Using default protrusion set `alltext'.
Package microtype Info: Automatic font expansion enabled (level 2),
(microtype)             stretch: 20, shrink: 20, step: 1, non-selected.
Package microtype Info: Using default expansion set `alltext-nott'.
LaTeX Info: Redefining \showhyphens on input line 92.
Package microtype Info: No adjustment of tracking.
Package microtype Info: No adjustment of interword spacing.
Package microtype Info: No adjustment of character kerning.
(c:/texlive/2024/texmf-dist/tex/latex/microtype/mt-cmr.cfg
File: mt-cmr.cfg 2013/05/19 v2.2 microtype config. file: Computer Modern
Roman
```

```
(RS)
)
LaTeX Font Info:    Redeclaring symbol font `operators' on input line 92.
LaTeX Font Info:    Encoding `OT1' has changed to `T1' for symbol font
(Font)              `operators' in the math version `normal' on input
line 92.
LaTeX Font Info:    Overwriting symbol font `operators' in version
`normal'
(Font)                  OT1/lmr/m/n --> T1/lmr/m/up on input line 92.
LaTeX Font Info:    Encoding `OT1' has changed to `T1' for symbol font
(Font)              `operators' in the math version `bold' on input line
92.
LaTeX Font Info:    Overwriting symbol font `operators' in version `bold'
(Font)                  OT1/lmr/bx/n --> T1/lmr/m/up on input line 92.
LaTeX Font Info:    Overwriting symbol font `operators' in version `bold'
(Font)                  T1/lmr/m/up --> T1/lmr/b/up on input line 92.
LaTeX Font Info:    Redeclaring math alphabet \mathbf on input line 92.
LaTeX Font Info:    Overwriting math alphabet `\mathbf' in version
`normal'
(Font)                  OT1/lmr/bx/n --> T1/lmr/b/up on input line 92.
LaTeX Font Info:    Overwriting math alphabet `\mathbf' in version `bold'
(Font)                  OT1/lmr/bx/n --> T1/lmr/b/up on input line 92.
LaTeX Font Info:    Redeclaring math alphabet \mathsf on input line 92.
LaTeX Font Info:    Overwriting math alphabet `\mathsf' in version
`normal'
(Font)                  OT1/lmss/m/n --> T1/lmss/m/up on input line 92.
LaTeX Font Info:    Overwriting math alphabet `\mathsf' in version `bold'
(Font)                  OT1/lmss/bx/n --> T1/lmss/m/up on input line 92.
LaTeX Font Info:    Redeclaring math alphabet \mathit on input line 92.
LaTeX Font Info:    Overwriting math alphabet `\mathit' in version
`normal'
(Font)                  OT1/lmr/m/it --> T1/lmr/m/it on input line 92.
LaTeX Font Info:    Overwriting math alphabet `\mathit' in version `bold'
(Font)                  OT1/lmr/bx/it --> T1/lmr/m/it on input line 92.
LaTeX Font Info:    Redeclaring math alphabet \mathtt on input line 92.
LaTeX Font Info:    Overwriting math alphabet `\mathtt' in version
`normal'
(Font)                  OT1/lmtt/m/n --> T1/lmtt/m/up on input line 92.
LaTeX Font Info:    Overwriting math alphabet `\mathtt' in version `bold'
(Font)                  OT1/lmtt/m/n --> T1/lmtt/m/up on input line 92.
LaTeX Font Info:    Overwriting math alphabet `\mathsf' in version `bold'
(Font)                  T1/lmss/m/up --> T1/lmss/b/up on input line 92.
LaTeX Font Info:    Overwriting math alphabet `\mathit' in version `bold'
(Font)                  T1/lmr/m/it --> T1/lmr/b/it on input line 92.
(c:/texlive/2024/texmf-dist/tex/context/base/mkii/supp-pdf.mkii
[Loading MPS to PDF converter (version 2006.09.02).]
\scratchcounter=\count320
\scratchdimen=\dimen260
\scratchbox=\box70
\nofMPsegments=\count321
\nofMParguments=\count322
\everyMPshowfont=\toks39
\MPscratchCnt=\count323
\MPscratchDim=\dimen261
```

```
\MPnumerator=\count324
\makeMPintoPDFobject=\count325
\everyMPtoPDFconversion=\toks40
) (c:/texlive/2024/texmf-dist/tex/latex/epstopdf-pkg/epstopdf-base.sty
Package: epstopdf-base 2020-01-24 v2.11 Base part for package epstopdf
Package epstopdf-base Info: Redefining graphics rule for `.eps' on input
line 4
85.
(c:/texlive/2024/texmf-dist/tex/latex/latexconfig/epstopdf-sys.cfg
File: epstopdf-sys.cfg 2010/07/13 v1.3 Configuration of (r)epstopdf for
TeX Liv
e
))
Package newfloat Info: `float' package detected.
*geometry* driver: auto-detecting
*geometry* detected driver: pdftex
*geometry* verbose mode - [ preamble ] result:
* driver: pdftex
* paper: a4paper
* layout: <same size as paper>
* layoutoffset:(h,v)=(0.0pt,0.0pt)
* modes: includefoot twoside
* h-part:(L,W,R)=(54.64pt, 488.22787pt, 54.64pt)
* v-part:(T,H,B)=(66.0pt, 745.04684pt, 34.0pt)
* \paperwidth=597.50787pt
* \paperheight=845.04684pt
* \textwidth=488.22787pt
* \textheight=715.04684pt
* \oddsidemargin=-17.62999pt
* \evensidemargin=-17.62999pt
* \topmargin=-47.76999pt
* \headheight=17.5pt
* \headsep=24.0pt
* \topskip=10.0pt
* \footskip=30.0pt
* \marginparwidth=48.0pt
* \marginparsep=10.0pt
* \columnsep=18.0pt
* \skip\footins=22.0pt plus 2.0pt
* \hoffset=0.0pt
* \voffset=0.0pt
* \mag=1000
* \@twocolumntrue
* \@twosidetrue
* \@mparswitchtrue
* \@reversemarginfalse
* (1in=72.27pt=25.4mm, 1cm=28.453pt)

Package caption Info: Begin \AtBeginDocument code.
Package caption Info: float package is loaded.
Package caption Info: hyperref package is loaded.
Package caption Info: End \AtBeginDocument code.
Package hyperref Info: Link coloring ON on input line 92.
(./main.out) (./main.out)
```

```
\@outlinefile=\write3
\openout3 = `main.out'.

\@gscitedetails=\box71
\@gscitedetailsheight=\skip164
\@gsheadbox=\box72
\@gsheadboxheight=\skip165
LaTeX Font Info:    Calculating math sizes for size <7.5> on input line
92.

LaTeX Font Warning: Font shape `T1/lmr/m/up' undefined
(Font)              using `T1/lmr/m/n' instead on input line 92.

LaTeX Font Info:    Trying to load font information for OML+lmm on input
line 9
2.
(c:/texlive/2024/texmf-dist/tex/latex/lm/omllmm.fd
File: omllmm.fd 2015/05/01 v1.6.1 Font defs for Latin Modern
)
LaTeX Font Info:    Trying to load font information for OMS+lmsy on input
line
92.
(c:/texlive/2024/texmf-dist/tex/latex/lm/omslmsy.fd
File: omslmsy.fd 2015/05/01 v1.6.1 Font defs for Latin Modern
)
LaTeX Font Info:    Trying to load font information for OMX+lmex on input
line
92.
(c:/texlive/2024/texmf-dist/tex/latex/lm/omxlmex.fd
File: omxlmex.fd 2015/05/01 v1.6.1 Font defs for Latin Modern
)
LaTeX Font Info:    External font `lmex10' loaded for size
(Font)              <7.5> on input line 92.
LaTeX Font Info:    External font `lmex10' loaded for size
(Font)              <6.24973> on input line 92.
LaTeX Font Info:    External font `lmex10' loaded for size
(Font)              <5.24997> on input line 92.
LaTeX Font Info:    Trying to load font information for U+euf on input
line 92.

(c:/texlive/2024/texmf-dist/tex/latex/amsfonts/ueuf.fd
File: ueuf.fd 2013/01/14 v3.01 Euler Fraktur
) (c:/texlive/2024/texmf-dist/tex/latex/microtype/mt-euf.cfg
File: mt-euf.cfg 2006/07/03 v1.1 microtype config. file: AMS Euler
Fraktur (RS)

)
LaTeX Font Info:    Trying to load font information for U+eus on input
line 92.

(c:/texlive/2024/texmf-dist/tex/latex/amsfonts/ueus.fd
File: ueus.fd 2013/01/14 v3.01 Euler Script
) (c:/texlive/2024/texmf-dist/tex/latex/microtype/mt-eus.cfg
```

```
File: mt-eus.cfg 2006/07/28 v1.2 microtype config. file: AMS Euler Script
(RS)
)
LaTeX Font Info:    Trying to load font information for U+euex on input
line 92
.
(c:/texlive/2024/texmf-dist/tex/latex/amsfonts/ueuex.fd
File: ueuex.fd 2013/01/14 v3.01 Euler extra symbols
)

LaTeX Font Warning: Font shape `OML/cmm/m/it' in size <7.5> not available
(Font)              size <7> substituted on input line 92.

TextBlockOrigin set to 4pc+6.64pt x 4pc+6pt
<gigascience-logo.pdf, id=69, 99.37125pt x 33.12375pt>
File: gigascience-logo.pdf Graphic file (type pdf)
<use gigascience-logo.pdf>
Package pdftex.def Info: gigascience-logo.pdf  used on input line 109.
(pdftex.def)             Requested size: 118.86127pt x 39.6174pt.

Overfull \hbox (54.64pt too wide) in paragraph at lines 109--109
[][]
 []

LaTeX Font Info:    Calculating math sizes for size <14> on input line
109.
LaTeX Font Info:    External font `lmex10' loaded for size
(Font)              <14> on input line 109.
LaTeX Font Info:    External font `lmex10' loaded for size
(Font)              <11.66617> on input line 109.
LaTeX Font Info:    External font `lmex10' loaded for size
(Font)              <9.79996> on input line 109.
LaTeX Font Info:    Calculating math sizes for size <13> on input line
109.
LaTeX Font Info:    External font `lmex10' loaded for size
(Font)              <13> on input line 109.
LaTeX Font Info:    External font `lmex10' loaded for size
(Font)              <10.83287> on input line 109.
LaTeX Font Info:    External font `lmex10' loaded for size
(Font)              <9.09996> on input line 109.

LaTeX Font Warning: Font shape `OML/cmm/m/it' in size <13> not available
(Font)              size <12> substituted on input line 109.

LaTeX Font Info:    Trying to load font information for TS1+lmr on input
line 1
09.
(c:/texlive/2024/texmf-dist/tex/latex/lm/ts1lmr.fd
File: ts1lmr.fd 2015/05/01 v1.6.1 Font defs for Latin Modern
)
LaTeX Font Info:    External font `lmex10' loaded for size
(Font)              <9> on input line 109.
LaTeX Font Info:    External font `lmex10' loaded for size
(Font)              <7> on input line 109.
```

```
LaTeX Font Info:    External font `lmex10' loaded for size
(Font)              <5> on input line 109.
LaTeX Font Info:    Calculating math sizes for size <6.5> on input line
109.
LaTeX Font Info:    External font `lmex10' loaded for size
(Font)              <6.5> on input line 109.
LaTeX Font Info:    External font `lmex10' loaded for size
(Font)              <5.41643> on input line 109.
LaTeX Font Info:    External font `lmex10' loaded for size
(Font)              <4.54997> on input line 109.

LaTeX Font Warning: Font shape `OML/cmm/m/it' in size <6.5> not available
(Font)              size <6> substituted on input line 109.


LaTeX Font Warning: Font shape `OML/cmm/m/it' in size <5.41643> not
available
(Font)              size <5> substituted on input line 109.


LaTeX Font Warning: Font shape `OML/cmm/m/it' in size <4.54997> not
available
(Font)              size <5> substituted on input line 109.


Overfull \hbox (54.64pt too wide) in paragraph at lines 109--109
[][][]
 []

LaTeX Font Info:    External font `lmex10' loaded for size
(Font)              <8> on input line 109.
LaTeX Font Info:    External font `lmex10' loaded for size
(Font)              <6> on input line 109.
LaTeX Font Info:    Trying to load font information for T1+lmtt on input
line 1
09.
(c:/texlive/2024/texmf-dist/tex/latex/lm/t1lmtt.fd
File: t1lmtt.fd 2015/05/01 v1.6.1 Font defs for Latin Modern
)
Package microtype Info: Loading generic protrusion settings for font
family
(microtype)             `lmtt' (encoding: T1).
(microtype)             For optimal results, create family-specific
settings.
(microtype)             See the microtype manual for details.

Overfull \hbox (54.64pt too wide) in paragraph at lines 109--109
[][][]
 []

Package mdframed Info: mdframed works in twoside mode on input line 119.
Package mdframed Info: mdframed inside float
 mdframed uses option nobreak mdframed on input line 129.
Package mdframed Info: mdframed inside a box
```

```
 mdframed uses option nobreak mdframed on input line 129.

[1{c:/texlive/2024/texmf-
var/fonts/map/pdftex/updmap/pdftex.map}{c:/texlive/202
4/texmf-dist/fonts/enc/dvips/lm/lm-ec.enc}{c:/texlive/2024/texmf-
dist/fonts/enc
/dvips/lm/lm-ts1.enc}{c:/texlive/2024/texmf-dist/fonts/enc/dvips/lm/lm-
mathsy.e
nc}

 <./gigascience-logo.pdf>]
Underfull \hbox (badness 10000) in paragraph at lines 152--154
[]\T1/lmr/m/up/7.5 (+20) Complementing these general-purpose pipelines,
sev-era
l
 []

Underfull \hbox (badness 1701) in paragraph at lines 152--154
\T1/lmr/m/up/7.5 (+20) lightweight tools have emerged to ad-dress spe-
cific nee
ds in
 []

Underfull \hbox (badness 2359) in paragraph at lines 152--154
\T1/lmr/m/up/7.5 (+20) clas-si-fi-ca-tion, while the Crit-i-cal As-sess-
ment of
 Metagenome
 []

Underfull \hbox (badness 2150) in paragraph at lines 152--154
\T1/lmr/m/up/7.5 (+20) In-ter-pre-ta-tion Tax-on-omy (CAMI-TAX) [[]] im-
proves
ac-cu-racy
 []

Underfull \hbox (badness 1783) in paragraph at lines 152--154
\T1/lmr/m/up/7.5 (+20) com-bines con-sen-sus clas-si-fi-ca-tions from es-
tab-li
shed clas-si-fiers,
 []

Underfull \vbox (badness 10000) has occurred while \output is active []
```

```
[2]
<arquitetura.png, id=221, 357.335pt x 474.77374pt>
File: arquitetura.png Graphic file (type png)
<use arquitetura.png>
Package pdftex.def Info: arquitetura.png  used on input line 174.
(pdftex.def)             Requested size: 235.11394pt x 312.38812pt.




[3{c:/texlive/2024/texmf-dist/fonts/enc/dvips/lm/lm-mathit.enc}
<./arquitetura.
png>]
! Undefined control sequence.
l.206     \text
               {Error} \approx \sqrt{\frac{1}{s}},
The control sequence at the end of the top line
of your error message was never \def'ed. If you have
misspelled it (e.g., `\hobx'), type `I' and the correct
spelling (e.g., `I\hbox'). Otherwise just continue,
and I'll forget about whatever was undefined.


Underfull \hbox (badness 10000) in paragraph at lines 242--242
[]|\T1/lmr/bx/n/7 (+20) Sketch
 []


Underfull \hbox (badness 10000) in paragraph at lines 242--242
\T1/lmr/bx/n/7 (+20) Pa-ram-e-
 []


Underfull \hbox (badness 10000) in paragraph at lines 242--242
[]|\T1/lmr/bx/n/7 (+20) Sketch
 []


Underfull \hbox (badness 10000) in paragraph at lines 242--242
[]|\T1/lmr/bx/n/7 (+20) Hash
 []


Overfull \hbox (3.52805pt too wide) in paragraph at lines 242--242
[]|\T1/lmr/m/n/7 (-20) sketch1.msh|
 []


Underfull \hbox (badness 10000) in paragraph at lines 242--242
[]|\T1/lmr/m/n/7 (+20) k=21,
 []
```

```
Overfull \hbox (3.52805pt too wide) in paragraph at lines 242--242
[]|\T1/lmr/m/n/7 (-20) sketch2.msh|
 []


Underfull \hbox (badness 10000) in paragraph at lines 242--242
[]|\T1/lmr/m/n/7 (+20) k=21,
 []


Underfull \hbox (badness 10000) in paragraph at lines 242--242
[]|\T1/lmr/m/n/7 (+20) 883.25
 []


Overfull \hbox (3.52805pt too wide) in paragraph at lines 242--242
[]|\T1/lmr/m/n/7 (-20) sketch3.msh|
 []


Underfull \hbox (badness 10000) in paragraph at lines 242--242
[]|\T1/lmr/m/n/7 (+20) k=15,
 []


Underfull \hbox (badness 10000) in paragraph at lines 242--242
[]|\T1/lmr/m/n/7 (+20) 327.93
 []





[4{c:/texlive/2024/texmf-dist/fonts/enc/dvips/lm/lm-mathex.enc}]
! Undefined control sequence.
l.277     \text
               {Weight} = \text{Coverage} \times \text{Abundance},
The control sequence at the end of the top line
of your error message was never \def'ed. If you have
misspelled it (e.g., `\hobx'), type `I' and the correct
spelling (e.g., `I\hbox'). Otherwise just continue,
and I'll forget about whatever was undefined.

! Undefined control sequence.
l.277     \text{Weight} = \text
                               {Coverage} \times \text{Abundance},
The control sequence at the end of the top line
of your error message was never \def'ed. If you have
misspelled it (e.g., `\hobx'), type `I' and the correct
spelling (e.g., `I\hbox'). Otherwise just continue,
and I'll forget about whatever was undefined.

! Undefined control sequence.
```

```
l.277 ...xt{Weight} = \text{Coverage} \times \text
                                                  {Abundance},
The control sequence at the end of the top line
of your error message was never \def'ed. If you have
misspelled it (e.g., `\hobx'), type `I' and the correct
spelling (e.g., `I\hbox'). Otherwise just continue,
and I'll forget about whatever was undefined.

! Undefined control sequence.
l.283     \text
               {Confidence Score} = \prod_{i=1}^{n} \text{Confidence at
Rank...
The control sequence at the end of the top line
of your error message was never \def'ed. If you have
misspelled it (e.g., `\hobx'), type `I' and the correct
spelling (e.g., `I\hbox'). Otherwise just continue,
and I'll forget about whatever was undefined.

! Undefined control sequence.
l.283 ...Confidence Score} = \prod_{i=1}^{n} \text
                                                  {Confidence at Rank}_i,
The control sequence at the end of the top line
of your error message was never \def'ed. If you have
misspelled it (e.g., `\hobx'), type `I' and the correct
spelling (e.g., `I\hbox'). Otherwise just continue,
and I'll forget about whatever was undefined.


Underfull \vbox (badness 4144) has occurred while \output is active []



Underfull \hbox (badness 10000) in alignment at lines 321--321
[][][]
 []


Underfull \vbox (badness 4181) has occurred while \output is active []



[5]
Underfull \hbox (badness 1442) in paragraph at lines 373--374
\T1/lmr/m/up/7.5 (+20) calls (ERR14251410, Mi-croBench) were as-sem-bled
with F
lye
 []




Underfull \vbox (badness 3009) has occurred while \output is active []
```

```
[6]
<fig_f1_by_rank_lines.png, id=376, 831.105pt x 433.62pt>
File: fig_f1_by_rank_lines.png Graphic file (type png)
<use fig_f1_by_rank_lines.png>
Package pdftex.def Info: fig_f1_by_rank_lines.png  used on input line
417.
(pdftex.def)             Requested size: 239.23433pt x 124.81364pt.
<fig_accuracy_by_rank_lines.png, id=377, 831.105pt x 433.62pt>
File: fig_accuracy_by_rank_lines.png Graphic file (type png)
<use fig_accuracy_by_rank_lines.png>
Package pdftex.def Info: fig_accuracy_by_rank_lines.png  used on input
line 418
.
(pdftex.def)             Requested size: 239.23433pt x 124.81364pt.
<fig_l1_braycurtis_lines.png, id=380, 757.3896pt x 402.5439pt>
File: fig_l1_braycurtis_lines.png Graphic file (type png)
<use fig_l1_braycurtis_lines.png>
Package pdftex.def Info: fig_l1_braycurtis_lines.png  used on input line
427.
(pdftex.def)             Requested size: 239.23433pt x 127.1521pt.
<fig_contig_accuracy_heatmap.png, id=381, 722.7pt x 289.08pt>
File: fig_contig_accuracy_heatmap.png Graphic file (type png)
<use fig_contig_accuracy_heatmap.png>
Package pdftex.def Info: fig_contig_accuracy_heatmap.png  used on input
line 42
8.
(pdftex.def)             Requested size: 239.23433pt x 95.69223pt.



[7 <./fig_f1_by_rank_lines.png> <./fig_accuracy_by_rank_lines.png>]
<fig_wall_time_by_tool.png, id=399, 705.3552pt x 526.1256pt>
File: fig_wall_time_by_tool.png Graphic file (type png)
<use fig_wall_time_by_tool.png>
Package pdftex.def Info: fig_wall_time_by_tool.png  used on input line
482.
(pdftex.def)             Requested size: 234.34729pt x 174.8021pt.
<fig_peak_memory_by_tool.png, id=400, 628.749pt x 526.1256pt>
File: fig_peak_memory_by_tool.png Graphic file (type png)
<use fig_peak_memory_by_tool.png>
Package pdftex.def Info: fig_peak_memory_by_tool.png  used on input line
483.
(pdftex.def)             Requested size: 234.34729pt x 196.10043pt.


<mut.png, id=406, 467.7475pt x 276.03125pt>
File: mut.png Graphic file (type png)
<use mut.png>
Package pdftex.def Info: mut.png  used on input line 501.
(pdftex.def)             Requested size: 235.11394pt x 138.74814pt.
```

```
[8 <./fig_l1_braycurtis_lines.png> <./fig_contig_accuracy_heatmap.png>
<./fig_w
all_time_by_tool.png> <./fig_peak_memory_by_tool.png>]
<fig_case_top_taxa_panels_case_gut.png, id=426, 794.97pt x 231.264pt>
File: fig_case_top_taxa_panels_case_gut.png Graphic file (type png)
<use fig_case_top_taxa_panels_case_gut.png>
Package pdftex.def Info: fig_case_top_taxa_panels_case_gut.png  used on
input l
ine 542.
(pdftex.def)             Requested size: 234.34729pt x 68.17288pt.
<fig_case_top_taxa_panels_case_zymo.png, id=427, 794.97pt x 231.264pt>
File: fig_case_top_taxa_panels_case_zymo.png Graphic file (type png)
<use fig_case_top_taxa_panels_case_zymo.png>
Package pdftex.def Info: fig_case_top_taxa_panels_case_zymo.png  used on
input
line 547.
(pdftex.def)             Requested size: 234.34729pt x 68.17288pt.


<fig_zymogut_dual_correlation.png, id=434, 994.917pt x 480.1137pt>
File: fig_zymogut_dual_correlation.png Graphic file (type png)
<use fig_zymogut_dual_correlation.png>
Package pdftex.def Info: fig_zymogut_dual_correlation.png  used on input
line 5
92.
(pdftex.def)             Requested size: 488.22787pt x 235.60208pt.


[9 <./mut.png>]
Underfull \hbox (badness 10000) in alignment at lines 647--647
[][][]
 []



<fig_ablation_rank_stack.png, id=446, 578.16pt x 325.215pt>
File: fig_ablation_rank_stack.png Graphic file (type png)
<use fig_ablation_rank_stack.png>
Package pdftex.def Info: fig_ablation_rank_stack.png  used on input line
683.
(pdftex.def)             Requested size: 223.35751pt x 125.6374pt.
<fig_ablation_f1_by_rank.png, id=447, 650.43pt x 361.35pt>
File: fig_ablation_f1_by_rank.png Graphic file (type png)
<use fig_ablation_f1_by_rank.png>
Package pdftex.def Info: fig_ablation_f1_by_rank.png  used on input line
685.
(pdftex.def)             Requested size: 223.35751pt x 124.08693pt.

Underfull \vbox (badness 10000) has occurred while \output is active []
```

```
[10 <./fig_case_top_taxa_panels_case_gut.png>
<./fig_case_top_taxa_panels_case_
zymo.png> <./fig_zymogut_dual_correlation.png>]



[11 <./fig_ablation_rank_stack.png> <./fig_ablation_f1_by_rank.png>]
Underfull \hbox (badness 10000) in paragraph at lines 730--731
[]\T1/lmr/m/up/7.5 (+20) Other re-quire-ments: Docker or App-
tainer/Singularity
;
 []


Underfull \hbox (badness 1946) in paragraph at lines 740--741
[]\T1/lmr/m/up/7.5 (+20) The Mash sketch databases used for can-di-date
se-lec-
tion
 []





[12]
Underfull \hbox (badness 1668) in paragraph at lines 773--774
[] []\T1/lmr/bx/it/7.5 (+20) Sup-ple-men-tary Fig. S6\T1/lmr/m/it/7.5
(+20) .[]
\T1/lmr/m/up/7.5 (+20) Com-par-i-son of HYMET con-tig vs.
 []


Underfull \hbox (badness 1210) in paragraph at lines 781--782
[] []\T1/lmr/bx/it/7.5 (+20) Sup-ple-men-tary Fig. S10\T1/lmr/m/it/7.5
(+20) .[
]\T1/lmr/m/up/7.5 (+20) Genus-level abun-dance com-par-i-son
 []


Underfull \vbox (badness 1087) has occurred while \output is active []



(./main.bbl
Underfull \hbox (badness 1577) in paragraph at lines 23--28
\T1/lmr/m/up/7.5 (+20) se-quence clas-si-fi-ca-tion us-ing ex-act align-
ments.
 Genome
 []


Underfull \hbox (badness 10000) in paragraph at lines 23--28
\T1/lmr/m/up/7.5 (+20) bi-ol-ogy 2014;15(3):1--12.  [][]$\T1/lmtt/m/n/7.5
https
```

```
 : / / doi . org / 10 . 1186 /
 []



[13]
Underfull \vbox (badness 10000) has occurred while \output is active []




Underfull \hbox (badness 2035) in paragraph at lines 289--293
\T1/lmr/m/up/7.5 (+20) Ba-sic lo-cal align-ment search tool.  Jour-nal of
molec
-u-
 []


Underfull \vbox (badness 10000) has occurred while \output is active []



[14]
Underfull \vbox (badness 10000) has occurred while \output is active []




Underfull \vbox (badness 10000) has occurred while \output is active []



[15]
Underfull \hbox (badness 10000) in paragraph at lines 651--654
\T1/lmr/m/up/7.5 (+20) 2025.  [][]$\T1/lmtt/m/n/7.5 https : / / www . ebi
. ac
. uk / metagenomics / api / v1 /
 []

)



[16

]
enddocument/afterlastpage: lastpage setting LastPage.
(./main.aux)
 ***********
LaTeX2e <2024-06-01> patch level 2
L3 programming layer <2020/03/25>
 ***********

LaTeX Font Warning: Size substitutions with differences
```

```
(Font)              up to 1.0pt have occurred.


LaTeX Font Warning: Some font shapes were not available, defaults
substituted.

Package rerunfilecheck Info: File `main.out' has not changed.
(rerunfilecheck)             Checksum:
851206F2AAC23F374675E2C9BBFC8690;2544.
 )
Here is how much of TeX's memory you used:
 20422 strings out of 473583
 342486 string characters out of 5732343
 1971908 words of memory out of 5000000
 42355 multiletter control sequences out of 15000+600000
 843334 words of font info for 409 fonts, out of 8000000 for 9000
 1141 hyphenation exceptions out of 8191
 123i,13n,121p,2062b,734s stack positions out of
10000i,1000n,20000p,200000b,200000s
<c:/texlive/2024/texmf-
dist/fonts/type1/public/amsfonts/euler/euex8.pfb><c:/t
exlive/2024/texmf-
dist/fonts/type1/public/amsfonts/euler/eusm7.pfb><c:/texlive/
2024/texmf-dist/fonts/type1/public/lm/lmbx10.pfb><c:/texlive/2024/texmf-
dist/fo
nts/type1/public/lm/lmbx12.pfb><c:/texlive/2024/texmf-
dist/fonts/type1/public/l
m/lmbx6.pfb><c:/texlive/2024/texmf-
dist/fonts/type1/public/lm/lmbx7.pfb><c:/tex
live/2024/texmf-
dist/fonts/type1/public/lm/lmbx8.pfb><c:/texlive/2024/texmf-dis
t/fonts/type1/public/lm/lmbx9.pfb><c:/texlive/2024/texmf-
dist/fonts/type1/publi
c/lm/lmbxi10.pfb><c:/texlive/2024/texmf-
dist/fonts/type1/public/lm/lmbxo10.pfb>
<c:/texlive/2024/texmf-
dist/fonts/type1/public/lm/lmex10.pfb><c:/texlive/2024/t
exmf-dist/fonts/type1/public/lm/lmmi6.pfb><c:/texlive/2024/texmf-
dist/fonts/typ
e1/public/lm/lmmi8.pfb><c:/texlive/2024/texmf-
dist/fonts/type1/public/lm/lmr10.
pfb><c:/texlive/2024/texmf-
dist/fonts/type1/public/lm/lmr12.pfb><c:/texlive/202
4/texmf-dist/fonts/type1/public/lm/lmr5.pfb><c:/texlive/2024/texmf-
dist/fonts/t
ype1/public/lm/lmr6.pfb><c:/texlive/2024/texmf-
dist/fonts/type1/public/lm/lmr7.
pfb><c:/texlive/2024/texmf-
dist/fonts/type1/public/lm/lmr8.pfb><c:/texlive/2024
/texmf-dist/fonts/type1/public/lm/lmr9.pfb><c:/texlive/2024/texmf-
dist/fonts/ty
pe1/public/lm/lmri7.pfb><c:/texlive/2024/texmf-
dist/fonts/type1/public/lm/lmri8
```

.pfb><c:/texlive/2024/texmf-
dist/fonts/type1/public/lm/lmsy8.pfb><c:/texlive/20
24/texmf-dist/fonts/type1/public/lm/lmtt8.pfb>
Output written on main.pdf (16 pages, 2060257 bytes).
PDF statistics:
 826 PDF objects out of 1000 (max. 8388607)
 722 compressed objects within 8 object streams
 171 named destinations out of 1000 (max. 500000)
 152775 words of extra memory for PDF output out of 154059 (max.
10000000)

Manuscript

Click here to access/download;Manuscript;HYMET_manuscript_R2.pdf

GigaScience, 2023, 1–16

doi: xx.xxxx/xxxx
Manuscript in Preparation
Paper

PAPER

HYMET: A Hybrid Metagenomic Pipeline for Accurate and Efficient Taxonomic Classification

Inês Martins[1,*], Jorge Miguel Silva[1,*] and João Rafael Almeida[1]

[1]IEETA/DETI, LASI, University of Aveiro, Aveiro, Portugal

[*]inesbrancomartins@ua.pt; jorge.miguel.ferreira.silva@ua.pt

Abstract

Background: Reliable taxonomic classification of metagenomic sequences remains constrained by high mutation rates, fragmented assemblies, and large heterogeneous reference databases. HYMET (Hybrid Metagenomic Tool) was developed to overcome these challenges through a two-stage hybrid design combining adaptive Mash-based screening with Minimap2 alignment and a coverage-weighted Lowest Common Ancestor (LCA) classifier. Its sample-adaptive thresholds and on-the-fly reference database construction enable efficient, domain-agnostic classification while maintaining accuracy across divergent genomes.
Results: Across seven CAMI assembly datasets in contig mode, HYMET achieved a mean F1 of 83.89%, with genus-level F1 of 76.75% and species-level F1 of 60.18%, while averaging 115.93 s runtime and a mean peak memory of 6.24 GB. Performance remained stable under mutation rates up to 30% for most domains (F1 $\geq$ 0.8), with viral sequences showing the expected decline (F1 $\approx$ 0.5 at 30%). Read and contig inputs produced nearly identical results when sharing reference caches, and real-world datasets confirmed robustness with the human gut metagenome reproduced typical anaerobic profiles, while in the ZymoBIOMICS mock community HYMET recovered all bacterial members; a further ground-truth evaluation on the ZymoBIOMICS Gut Microbiome Standard (D6331) yielded near-perfect genus-level concordance (Pearson $r = 0.998$, Bray–Curtis = 0.04) across bacteria, fungi, and archaea.
Conclusions: HYMET achieves a practical balance of accuracy, efficiency, and scalability for metagenomic classification. Its adaptive candidate selection, alignment-anchored taxonomy, and reproducible reference caching collectively enhance performance across domains. HYMET source code is fully available at https://github.com/ieeta-pt/HYMET.

Key words: Metagenomics, taxonomic classification, *k*-mer screening, alignment-based methods, computational efficiency, mutation resistance, hybrid pipeline.

1 Introduction

A significant challenge in metagenomics is the development of accurate methods for the taxonomic classification of organisms within a sample [1, 2]. Despite the creation of numerous general-purpose and specialized metagenomic tools, several significant issues persist. Particularly, computational demands pose a major constraint, as tools often require substantial memory and processing power, leading to impractical execution times for large datasets [3, 4, 1]. The sheer volume of metagenomic datasets demands highly efficient algorithms that can operate within reasonable requirements of compute power, which is particularly problematic when dealing with millions of sequencing reads [5, 6, 7, 8]. Furthermore, taxonomic assignment remains a critical challenge in metagenomic analysis, especially at lower taxonomic levels [8]. This issue is exacerbated by the limitations of reference databases, which often exhibit significant sampling bias towards well-studied organisms, while underrepresenting species that are difficult to culture in laboratory settings [3, 1]. This discrepancy results in high rates of unclassified or misclassified reads, especially in complex environmental samples [9, 2, 10]. Furthermore, the lack of standardized benchmarking protocols and datasets, hinder objective comparisons of tool performance, as researchers frequently test tools on non-uniform datasets with inconsistent evaluation metrics [10, 11, 12]. Addressing these issues is crucial for improving our understanding of complex microbial communities and developing efficient, user-friendly software solutions to analyze the enormous amounts of data generated by metagenomic research [3, 1]. These collective challenges directly motivate our core research question:

> *How can a next-generation metagenomic classification tool be designed and implemented to accurately identify taxa across all domains while maintaining high performance and efficiency?*

Compiled on: February 27, 2026.
Draft manuscript prepared by the author.

Key Points

HYMET integrates Mash-based adaptive screening and Minimap2 alignment with a weighted Lowest Common Ancestor (LCA) classification, dynamically tailoring reference databases to each metagenomic dataset for improved efficiency and accuracy.

Evaluated across diverse CAMI benchmarks, HYMET achieves robust F1-scores (mean across ranks: 83.89%, genus: 76.75%, species: 60.18%) while maintaining rapid runtimes (average 116 s) and moderate memory usage (mean peak 6.2 GB, sample-dependent).

HYMET demonstrates strong resilience to genetic mutations (up to 30%), maintaining high accuracy for most biological domains (F1 $\geq$ 0.8), though viral classification accuracy decreases at higher mutation rates (F1 $\approx$ 0.5 at 30%).

Real-world evaluations confirm HYMET's practical applicability: gut microbiome profiles closely matched expected anaerobic bacterial taxa, while ZymoBIOMICS mock-community results accurately identified bacterial constituents, despite missing yeast species under current parameters, and genus-level classification on the ZymoBIOMICS Gut Microbiome Standard (D6331) reached near-perfect agreement with the ground truth ($r = 0.998$).

Resource-efficient design enables reproducible and scalable runs, the tool requires only 2.82 GB for installation, dynamically manages reference cache footprints (10–50 GB typical), and offers full reproducibility via Bioconda, container images, and versioned databases deposited on Zenodo.

HYMET (Hybrid Metagenomic Tool) was conceived in response to the recurrent bottlenecks observed when processing large and diverse metagenomic datasets, where existing profilers either failed to capture divergent taxa or consumed excessive resources. Its design draws inspiration from practical experience with real microbial communities, where rapid screening and selective reference database construction often proved more effective than relying on static, monolithic databases. HYMET follows this principle by integrating adaptive reference selection with precise alignment and a weighted taxonomic resolver. This hybrid, sample-aware approach enables the tool to dynamically tailor its search space to each dataset, thereby reducing memory usage and execution time while preserving classification accuracy across domains.

Specifically, HYMET introduces three main innovations. First, an adaptive Mash Screen step selects candidate references on-the-fly, ensuring that downstream analysis focuses only on the most relevant genomes under a fixed resource budget. Second, the selected references are combined into a temporary, sample-specific database that supports accurate alignment even in the presence of mutations or incomplete references. Third, a coverage-weighted lowest common ancestor (LCA) algorithm integrates the breadth and depth of alignment evidence to improve taxonomic consistency, particularly at lower ranks. Together, these components allow HYMET to outperform static index-based approaches in both speed and precision, especially for complex or previously unseen samples.

2 Background

In recent years, we have witnessed remarkable progress in metagenomics, particularly in the development of computational tools for taxonomic classification and functional analysis [2, 1, 3]. A dominant trend in current methodologies is the integration of established classification techniques into end-to-end pipelines, which streamline the entire analytical workflow, from raw sequencing data to biologically interpretable results [13]. Currently, the state-of-the-art landscape is populated by a rich ecosystem of interconnected tools, each offering unique capabilities and complementary approaches that collectively advance the field's analytical power. Among these, SnakeMAGs [14] stands out for its specialized focus on reconstructing prokaryotic genomes from Illumina sequencing reads, while SqueezeMeta [15] offers a fully automated and comprehensive solution for metagenomic data analysis [16, 13]. The first tool uses the Genome Taxonomy Database (GTDB) toolkit [17] for taxonomic assignment, leveraging conserved marker genes for analysis. On the other hand, SqueezeMeta uses DIAMOND [18] for alignment and the Lowest Common Ancestor (LCA) algorithm for taxonomic assignment [15].

Complementing these general-purpose pipelines, several lightweight tools have emerged to address specific needs in taxonomic assignment. The Basic Sequence Taxonomy Annotator (BASTA) [19] also employs the LCA algorithm for efficient sequence classification, while the Critical Assessment of Metagenome Interpretation Taxonomy (CAMITAX) [4] improves accuracy through the integration of multiple classification strategies for microbial genome assignment, including genome distance-based classification using Mash [20], Centrifuge [1] and Kaiju [21], that determines the interval-union LCA of gene-level assignments and 16S rRNA gene-based classification employing a naive Bayesian classifier method using Dada2 [22]. For more robust taxonomic profiling, the Taxonomy Analysis by Multiple Assignment (TAMA) tool [23] combines consensus classifications from established classifiers, including Kraken [8], CLARK [24], and Centrifuge, leveraging their complementary strengths.

In addition to these workflows, widely used read-level classifiers include KrakenUniq, Ganon (and ganon2), Centrifuge/Centrifuger, Taxor, and compositional MinHash methods such as sourmash gather [25, 26, 27, 28, 29, 30]. These tools are often embedded within workflows (for example, CAMITAX integrates Centrifuge/Kaiju and TAMA combines Kraken, CLARK, and Centrifuge). In our CAMI contig benchmark we therefore include representative stand-alone baselines that accept contig inputs and emit per-contig labels (Kraken 2, Centrifuge, Ganon 2, and sourmash gather) while citing [29] as a state-of-the-art long-read read-level classifier that falls outside this contig-based evaluation.

The field has also seen the development of specialized tools that target specific metagenomic applications. Viral genomics is particularly well served by PhaBOX [31, 32] for viral contig characterization and ViWrap [33] for prediction of viral-host relationship, both providing valuable information on viral diversity and ecological interactions [34, 35, 36]. The first tool, PhaBOX, developed by Shang et al., combines gene prediction and alignment (DIAMOND) with taxonomic classification by semi-supervised learning method (PhaGCN [32]), based on sequence similarities and cluster sharing networks, and final assignments using the LCA. ViWrap, on the other hand, uses machine learning and sequence similarity searches to identify viral sequences and BLAST [37] to identify best hits against databases for taxonomic annotation and host prediction. For the analysis of the microbial community, PhyloFlash [38], developed by Gruber-Vodicka et al., offers unique capabilities through its small subunit ribosomal RNA (SSU rRNA)-based approach, enabling both metagenomic profiling and high-resolution phylogenetic studies [39]. In the critical area of antimicrobial resistance surveillance, MegaPath-Nano [40] has emerged as an important tool for the comprehensive detection of resistance genes, directly supporting public health monitoring efforts; it couples hash-based k-mer mapping with Minimap2's seed-chain-extend *local* alignment model [41], thereby covering broad and potentially divergent sequence segments [42, 43].

Related alignment-based profilers adopt a two-stage design. For example, Metalign [44] first applies CMash to pre-filter the reference by containment and then aligns reads with Minimap2 to produce the profile.

Despite this technological progress, significant challenges impede the broader implementation of metagenomic tools in clinical and research settings. Implementation barriers represent a primary obstacle, with inadequate documentation and complex installation procedures frequently compromising tool accessibility and user adoption [45]. Computational constraints further limit practical application, as excessive memory and storage requirements hinder scalability. This is exemplified by SqueezeMeta, which stages more than 500 GB of on-disk reference data and exhibits prohibitively long processing times [15], and BASTA, whose dependence on BLAST-based alignments creates computational bottlenecks that render it inefficient for time-sensitive analyses [19, 37]. A fundamental limitation stems from reference database dependencies rather than inherent tool restrictions. For instance, TAMA demonstrates robust classification capabilities in principle, but its default bacterial reference database necessarily limits its taxonomic scope to bacterial identification while requiring hundreds of gigabytes of disk space for its bundled indices [23]. Similarly, independent evaluations of MegaPath-Nano confirm its strong performance in the detection of prokaryotic antimicrobial resistance, but note a reduced sensitivity when analyzing higher eukaryotes [40, 42]. This pattern of taxonomic bias is further evidenced in specialized tools such as PhaBOX and ViWrap, which, while excelling in virome analysis, lack versatility for broader metagenomic applications [33, 31]. PhyloFlash's reliance on small subunit rRNA analysis makes it fundamentally unsuitable for viral identification, as viruses lack ribosomal RNA genes [38].

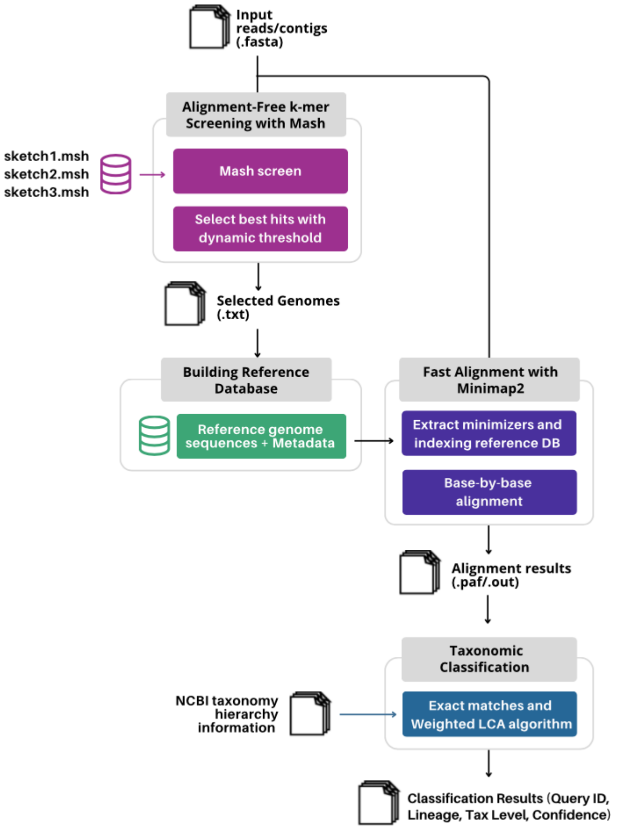

Figure 1. Overview of HYMET architecture.

3 Materials and Methods

3.1 HYMET workflow overview

HYMET, illustrated in Figure 1, is driven by a unified Python CLI (`hymet` from Bioconda or `bin/hymet` in a source checkout) that orchestrates Mash (k-mer screening), Minimap2 (alignment), and the weighted-LCA classifier. For reproducible installs we provide container images (Docker and Apptainer/Singularity) and a Bioconda package (Conda/Mamba). Legacy Perl components are bundled for reproducibility but are not the primary entry point. HYMET is designed for shotgun metagenomes in contig or genome form. It can classify 16S/SSU rRNA sequences when they occur in assembled contigs or shotgun reads, but targeted 16S rRNA amplicon libraries are out of scope and are better analyzed with rRNA-centric profilers (e.g., phyloFlash [38]). Installation and configuration details are provided in Supplementary Sections 1–4.

Alignment-Free k-mer Screening

The initial phase of HYMET utilizes Mash Screen [20] for rapid k-mer-based screening against pre-computed MinHash reference sketch databases [46, 47, 48, 49, 50]. Mash employs containment scores, as defined in Equation 1, to assess the proportion of a reference genome present in a query sequence [51, 52, 53]. The containment index $c_k(a, b)$ is estimated as:

$$c_k(a, b) \approx \frac{|S(A) \cap \pi(B)|}{|S(A)|}, \tag{1}$$

where $S(A)$ is the sketch of the reference genome A, and $\pi(B)$ represents the k-mers of the query sequence B. The containment index ranges from 0.0 to 1.0, with values closer to 1.0 indicating a higher proportion of k-mers from the reference genome present in the query. This metric is crucial for tasks such as contamination screening, reference genome selection, and the discovery of novel genomes, as it provides a rapid and unbiased estimate of sequence representation [46, 47, 51, 49, 54]. In this work, the screening process was optimized for computational efficiency by enabling parallel processing and applying a stringent 90% similarity threshold to retain only high-confidence matches, filtering out low-quality alignments. This containment-based approach prioritizes likely taxonomic candidates, reducing the search space and computational load for subsequent alignment-based stages [20, 46, 52]. As previously mentioned, to enable this screening, Mash relies on sketched databases, which are compact representations of genomic sequences. These databases are built using consistent k-mer hashing with MurmurHash3 [20, 55, 47, 56], which allows efficient comparison of query sequences against large collections of reference genomes.

HYMET requires fast per-reference containment ranking on unassembled reads under a fixed memory budget. Mash Screen provides fixed-size MinHash sketches, direct read-set → reference containment estimates, and per-hit p-values, which integrate cleanly with our adaptive cut-off and candidate budgeting. In contrast, sourmash uses scaled FracMinHash with an iterative gather (set-cover) procedure aimed at mixture decomposition rather than per-reference ranking, and CMash (as in Metalign [44]) focuses on multi-k containment estimation, which we do not require here. Consequently, HYMET applies a dynamic, sample-adaptive containment threshold that by default targets about 3.25 candidates per input sequence (rounded; minimum of five) and enforces a floor threshold of 0.70, reducing the search space while preserving recall. The pre-filter is modular and could be replaced by sourmash or CMash without altering downstream alignment; we chose Mash for its simplicity, stable memory, and native significance testing [30, 57, 44].

Sketch and k-mer Size. The construction of these sketch databases involves two key parameters: the k-mer size (k) and the sketch size (s). The choice of k-mer size is essential as it balances sensitivity and specificity. Smaller k-mers increase sensitivity for divergent genomes but may lead to random collisions, while larger k-mers reduce collisions but may miss subtle variations [58, 20, 47, 51, 50].

The optimal k-mer size is calculated as:

$$k = \log_{|\Sigma|}\left(\frac{n(1-q)}{q}\right), \quad (2)$$

where $|\Sigma|$ is the alphabet size (4 for nucleotides), n is the genome size, and q is the desired probability of observing a random k-mer. For example, smaller genomes (e.g. viruses) and highly variable taxa require smaller k-mer sizes (e.g. k-mer=15) to ensure specificity, while larger genomes (e.g. vertebrates) benefit from moderate k-mer sizes (e.g. k-mer=21) to balance sensitivity and computational efficiency [53, 58].The sketch size, which refers to the number of unique min-hashes retained for genomic sequence representation, also plays a critical role in determining the accuracy of distance and containment estimates [58, 20, 50, 52]. The error associated with containment estimation for a given sketch size, s, is proportional to:

$$Error \approx \sqrt{\frac{1}{s}}, \quad (3)$$

indicating that larger sketch sizes improve precision, but at the expense of greater computational resources [58]. For instance, smaller or highly fragmented genomes typically require larger sketch sizes to ensure sufficient genomic information is captured, while larger or less fragmented genomes can achieve accurate containment estimates with smaller sketch sizes. This adaptive approach is supported by empirical evidence, with studies demonstrating that a sketch size of $s = 1000$ is generally adequate for obtaining precise similarity estimates in well-assembled genomes [20]. In fact, B. D. Ondov *et al.* established $s = 1000$ and k=21 as the default parameters in Mash, as they provide precise similarity estimates for well-assembled genomes [20]. However, for more divergent genomes, increasing the sketch size (e.g. $s = 5000$) can improve accuracy by capturing a more representative subset of genomic content [58, 50, 59, 53].

Reference Sketched Databases. Following these design principles, we implemented a comprehensive database strategy combining both established public resources and a custom-built collection:

- **RefSeq Nucleotide Release 88:** Pre-built sketch distributed by the Mash project [58], comprising organisms from RefSeq nucleotide release 88, compressed using $k = 21$ and $s = 1000$. This sketch serves as the primary screening database, totalling 1.2 GB.
- **GTDB r202 Assembly Set and NCBI Complete Genomes Database:** Combines 89 675 genomes from GTDB r202 and NCBI RefSeq (viruses, fungi, and bacteria/archaea), compressed with $k = 21$ and $s = 1000$ [56].
- **Custom Reference Database:** Enhances representation of underrepresented taxa by including 19 505 up-to-date genomes from NCBI RefSeq. For smaller genomes (e.g., archaea, fungi, protozoa, viruses), sketches were generated with $k = 15$ and $s = 5000$, while larger genomes (e.g., vertebrates, plants, invertebrates) used the default parameters ($k = 21$, $s = 1000$) [50].

These databases were grouped on the basis of shared hash seed values and parameters to optimize the screening efficiency. Table 1 summarizes their characteristics. All databases are publicly available through our project repository. For reproduction, detailed instructions are provided in Supplementary Material Section 2, Subsection "Reproducing Sketched Databases".

Table 1. Reference sketched databases. Different hash seed values reflect the provenance of each sketch set. RefSeq88 sketch from Mash with hash seed 0, GTDB and custom sketches generated locally using default hash seed 42.

Sketch	Content	Sketch Parameters	Sketch Size	Hash Seed
sketch1.msh	RefSeq nucleotide release 88	k=21, s=1000	1.2 GB	0
sketch2.msh	GTDB r202 Assembly Set, NCBI Complete Genomes Database + Custom databases (vertebrates, plants, invertebrates)	k=21, s=1000	883.25 MB	42
sketch3.msh	Custom databases (fungi, protozoa, archaea, virus)	k=15, s=5000	327.93 MB	42

Modular Reference Database Download

Candidate selection with dynamic threshold. After running Mash Screen, the output can be extensive, potentially including a large number of candidate genomes with varying degrees of similarity to the query sequences. Downloading and analyzing this entire list would be computationally inefficient and could introduce noise into subsequent analyses. On the other hand, setting an arbitrarily high static threshold might exclude important reference genomes, leading to incomplete coverage of the query sequences. To address these challenges, HYMET introduces a dynamic Mash-Screen thresholding mechanism to identify the most relevant candidate genomes and to create a targeted, input-specific database. The algorithm iteratively lowers the containment threshold in 0.02 steps until a minimum candidate count is reached (about 3.25 per input sequence, rounded; minimum five), or until a floor of 0.70 is reached.

Genome Retrieval. Following the selection of candidate genomes, their format was analyzed to enable efficient mapping and retrieval. These genomes were identified using RefSeq (GCF) and Genbank Assembly Genomes (GCA) accession numbers, unique identifiers assigned by NCBI. Thus, the NCBI Assembly database [60] was selected as the primary resource for constructing the reference database [10, 61]. To optimize the process, summary files from the NCBI Assembly database were downloaded, providing efficient access to metadata. A custom script was developed to map candidate genomes to these files using accession numbers. This script extracted the base accession number (e.g. "000169215") to ensure compatibility between different assembly versions (e.g.: GCF_000169215.1, GCF_000169215.2), preventing retrieval failures due to version updates [60].

During the initial mutation study (conducted with the systematic-review harness), genomes were retrieved from the NCBI FTP service and decompressed locally. In all subsequent benchmarks and case studies in this manuscript, genomes were retrieved over HTTPS from NCBI and decompressed locally. The downloader uses bounded retries with exponential backoff and records failures in the run logs. To enhance efficiency, the script employed ThreadPoolExecutor for parallel downloads, allowing up to 64 concurrent threads. The taxonomy IDs (TaxID) of the assembly files were stored alongside the accession numbers and sequence identifiers, creating a comprehensive reference linking each genome to its taxonomic and sequence-level information [60, 61].

HYMET is designed to operate efficiently in network-constrained or offline environments. Users can supply a local directory containing mirrored NCBI assembly summaries to enable species-level candidate deduplication without requiring internet connectivity. Reference data are cached locally, and users have the option to preload these caches with curated FASTA files along with their corresponding sequence-to-TaxID mappings. Indices are automatically updated during subsequent analyses, ensuring reproducibility and consistency even in offline scenarios. Additional details can be found in "Reference retrieval policy and fallbacks" of the Supplementary Material.

Fast Alignment

In the second processing stage, HYMET employs Minimap2 for efficient and precise sequence alignment. This choice was motivated by Minimap2's adaptive scoring system and its seed-chain-extend *local* alignment strategy, which enables accurate mapping even with highly divergent sequences or incomplete reads, making the pipeline particularly resilient to common metagenomic challenges such as mutation-rich or fragmented samples [40, 41]. The pipeline uses minimizers to index reference sequences, enabling the rapid identification of alignment regions [62]. For contig/genome inputs we use the `-x asm10` preset, optimized for genome-to-genome alignment (approximately 10% divergence, or 90% identity) [63, 41]; for read inputs we use Minimap2's short-read preset `-x sr`. The results are saved in a PAF file, providing essential alignment details such as sequence IDs, lengths, positions, and mapping quality [41, 64].

Taxonomic Assignment

HYMET uses a hybrid taxonomic assignment strategy, combining the LCA algorithm with a weighted approach based on alignment coverage [23, 8, 24]. For exact matches, the reference's taxonomic lineage is directly assigned with a confidence score of 1.0. On the other hand, for non-exact matches, the weights for each TaxID are calculated according to:

$$Weight = Coverage \times Abundance, \tag{4}$$

where *Coverage* is the proportion of the query aligned with the reference and *Abundance* is the reference's frequency in the dataset. HYMET then computes a weighted consensus lineage across ranks and reports a single representative TaxID (the taxon with the highest cumulative weight across the supporting alignments). The confidence score is derived as the product of the per-rank consensus fractions.

$$ConfidenceScore = \prod_{i=1}^{n} ConfidenceatRank_i, \tag{5}$$

where n is the number of ranks. This ensures higher consistency across ranks results in higher confidence scores. The final output includes the query identifier, a taxonomic lineage (kingdom to strain), the most specific rank, the representative NCBI TaxID, and a confidence score (0.0 to 1.0), reflecting the reliability of the classification [24, 65, 66]. The tab-delimited file `classified_sequences.tsv` contains the columns: Query, Lineage, Taxonomic Level, TaxID, Confidence.

All analyses, including tool evaluation, development, and validation, were conducted on a high-performance Linux-based virtual machine with 2 TB storage and 250 GB RAM. HYMET's performance was assessed using precision and F1 score metrics for classifying organisms across the three domains of life, considering taxonomic levels (kingdom to species) and mutation rates (0% to 30%). The analysis also examined the relationship between F1 scores, execution time, and resource usage (CPU and memory), ensuring a complete evaluation of precision and efficiency. The same methodology was applied to the other current state-of-the-art tools described in Section 1 to ensure a consistent comparison. Benchmarking was performed by comparing HYMET with general-purpose cross-domain workflows that produce per-read taxonomic assignments. Component-level short-read classifiers (e.g., KrakenUniq, Ganon, Centrifuge/Centrifuger) and compositional search methods (e.g., sourmash gather) were out of scope for head-to-head benchmarking; their behavior is represented through the workflows that include them. Detailed instructions for reproducing the benchmarking of these tools are provided in Supplementary Section 5.

Table 2. Composition of the test and validation dataset

**Domain/Group**	**Number of GCFs**	**Size (GB)**
Viruses	1 498	0.05
Other Vertebrates	43	2.83
Vertebrate Mammals	23	2.29
Protozoa	12	0.03
Plants	19	1.02
Invertebrates	43	1.14
Fungi	63	0.15
Bacteria	24 271	7.23
Archaea	231	0.05
**Total**	**26 203**	**14.76**

3.2 Test and Validation Dataset

The test dataset was derived from the NCBI RefSeq Assembly database (last modified: 13 October 2024), chosen for its curated and validated sequences [67, 10, 68]. Assembly summary files for all biological domains and viruses were downloaded and 10% of the entries were randomly selected based on GCF accession numbers. For each GCF, 10% of its genome sequences were further sampled to ensure proportional representation and mimic the fragmentation of metagenomic data [69]. This approach resulted in a diverse and representative dataset, as detailed in Table 2. For dataset replication, complete instructions and scripts are provided in the Supplementary Material Section 3, Subsection “Replicating the Benchmark Dataset”.

3.3 CAMI Benchmarking Design

To assess the performance of HYMET alongside other baseline metagenomic classification tools, we performed standardized benchmarking using the CAMI (Critical Assessment of Metagenome Interpretation) datasets. Specifically, we selected seven publicly available contig assemblies representing various levels of complexity and ecological contexts. These include the Low, Medium, and High Complexity communities from CAMI I, the Mouse Gut, Marine, and Strain Madness panels from CAMI II, and the CAMI reference sample_0 (details are provided in Table 3).

We executed all tools strictly in contig mode to maintain consistent conditions across evaluations. To ensure fair comparisons, our benchmarking pipeline standardized input staging, fixed the number of computational threads, and maintained consistent input/output management. Additionally, it captured computational metrics such as wall-clock and CPU time, as well as peak memory usage.

The evaluation methodology adhered closely to established CAMI conventions. We reported metrics across taxonomic ranks from superkingdom down to species level, including both profile-based distances (L1 total variation and Bray–Curtis distance) and precision, recall, and F1-score for presence/absence classification (with a minimum abundance threshold of 0.1%). Contig-level accuracy was determined by directly comparing predicted taxonomic IDs (TaxIDs) with the CAMI-provided ground truth. Results were summarized individually for each sample and aggregated by taxonomic rank across all seven datasets.

Tools included in the benchmarking process were HYMET, Kraken 2, Centrifuge, Ganon 2, TAMA, SqueezeMeta, ViWrap, MegaPath-Nano, BASTA, CAMITAX, MetaPhlAn 4, sourmash gather, phyloFlash, SnakeMAGs, and PhaBOX. Each tool was configured using its recommended database or default settings suitable for contig-based inputs. The specific software versions and database sources utilized are fully documented in the supplementary materials.

Table 3. CAMI benchmark samples and inputs used in the benchmark. All evaluations are contig based and scored against CAMI truth profiles and contig maps.

Sample ID	CAMI panel	Complexity/context	Input	Truth assets
cami_i_lc	CAMI I	Low complexity community	contigs	profile + contig truth
cami_i_mc	CAMI I	Medium complexity community	contigs	profile + contig truth
cami_i_hc	CAMI I	High complexity community	contigs	profile + contig truth
cami_ii_mousegut	CAMI II	Mouse gut metagenome	contigs	profile + contig truth
cami_ii_marine	CAMI II	Marine metagenome	contigs	profile + contig truth
cami_ii_strainmadness	CAMI II	Strain Madness (strain variation)	contigs	profile + contig truth
cami_sample_0	CAMI reference	Reference assembly	contigs	profile + contig truth

3.4 HYMET Contig vs Read Evaluation

To assess how input modality affects HYMET's performance, each CAMI assembly dataset listed in Table 3 was analyzed under two conditions: first, using assembled contigs, and second, using synthetic reads generated from these same datasets. Both modes followed the same general workflow: initial sketch-based screening for candidate references, construction or reuse of reference caches, sequence alignment, and weighted lowest-common-ancestor taxonomic assignment. Conditions such as computational threading, file handling, and benchmarking instrumentation remained consistent between modes. The primary distinction between the two analyses involved the alignment parameters and input handling. For the contig mode, HYMET employed alignment settings optimized specifically for longer, genome-to-genome comparisons. In contrast, the read mode processed single-end reads with alignment parameters tailored for shorter, fragmented sequences. Importantly, steps like candidate selection, reference caching, and taxonomic classification remained unchanged, ensuring comparability across modalities. For each dataset and input modality, the benchmarking framework calculated CAMI performance metrics at all taxonomic ranks, including L1 total variation, Bray–Curtis dissimilarity, precision, recall, and F1 scores. Additionally, wall-clock time and peak memory usage were recorded. Results were first summarized individually per sample, then aggregated as averages across all seven datasets to enable a balanced comparison of performance between contig and read inputs.

3.5 Case-study Design: Gut, Zymo, and ZymoGut

To complement the CAMI benchmarks with real samples, we conducted three case studies that reflect common metagenomic contexts: a human gut metagenome assembly from MGnify, the ZymoBIOMICS mock community assembly curated by the Loman Lab, and the ZymoBIOMICS Gut Microbiome Standard (D6331), a manufactured gut mock community comprising 21 strains across 15 species of bacteria, fungi, and archaea. All three were processed in contig mode using the same workflow in Section 3.1, with a fixed number of threads, shared taxonomy inputs, and identical reference caching policy. This ensured that any differences in outcomes arise from sample biology rather than methodological drift.

The Zymo mock community provides a laboratory-defined composition and a canonical set of reference genomes. Ground truth was established at two levels: (i) contig-level labels by mapping assembled contigs to the curated Zymo reference panel and assigning each contig to a species TaxID; and (ii) a CAMI-style abundance profile used to compute rank-wise precision, recall, F1, and abundance distances (L1 total variation and Bray–Curtis). The human gut assembly does not have a strict gold standard; evaluation therefore emphasized plausibility of dominant taxa and concordance with public annotations, together with resource measurements.

The ZymoGut D6331 standard provides a more complex, gut-relevant composition with known ground truth, bridging the gap between the descriptive gut evaluation and the rigorous quantitative Zymo mock assessment. Oxford Nanopore SUP basecalls (ERR14251410, MicroBench) were assembled with Flye in metagenome mode (`--nano-hq --meta`), yielding 535 contigs. Ground truth was established by mapping assembled contigs to the manufacturer's reference genomes (minimap2, `-x asm5`); a CAMI-style abundance profile was derived from contig counts.

For all samples, we recorded wall-clock time and peak resident memory for the complete pipeline. Table 4 summarizes the inputs and available truth assets.

In addition to the main Zymo case study, we conducted a targeted reference-ablation protocol to probe robustness to incomplete databases, an increasingly common scenario as public catalogues lag behind newly observed strains. Using the same reference panel employed in the case analysis, we progressively withheld the indexed sequences for each of the ten constituent organisms (TaxIDs 562, 28901, 1423, 1639, 1351, 1280, 1613, 287, 4932, 5207) at 0%, 25%, 50%, 75%, and 100% removal levels. After each removal step we rebuilt the reference index and repeated the full HYMET classification pipeline without changing any parameters. For every level we captured the distribution of contig assignments by taxonomic rank (species or strain, genus, family, and higher) alongside the standard rank-wise profile metrics (precision, recall, F1, L1 total variation, Bray–Curtis) and runtime measurements. This procedure isolates the impact of reference incompleteness while keeping the sample and analytical settings fixed, thereby reflecting realistic deployments in which key genomes are missing or outdated.

4 Results

Benchmark Scope and Replicates

We report results over seven CAMI assembly datasets (Table 3): CAMI I low/medium/high complexity; CAMI II mouse gut, marine, and strain madness; and the CAMI reference sample_0. Each dataset was processed once per tool under fixed threads, yielding one run per sample per tool; no technical replicates were used. Rank-wise summaries reflect means across the seven datasets. The manifest that enumerates these samples is versioned in the repository.

For HYMET's modality comparison, the same seven datasets were analysed twice: (i) contigs as provided; and (ii) deterministic synthetic reads created by windowing contigs into 250 bp slices with a 125 bp minimum tail. Aside from input handling and Minimap2 presets, all pipeline stages (candidate selection, cache construction, alignment, weighted-LCA) and resource controls were identical; again, one run per sample per mode (no replicates).

The mutation-sweep experiment spans nine higher-level groups (Viruses, Archaea, Bacteria, Fungi, Plants, Protozoa, Invertebrates, Vertebrate Mammals, Other Vertebrates). For each group, we generated one mutated contig set per rate between 0% and 30% using reproducible seeds (substitutions with short indels), then computed precision/recall/F1 by rank from contig-level truth. No technical replicates were used in this sweep.

Real-data evaluations comprise three case studies (MGnify gut assembly, ZymoBIOMICS mock community, and ZymoBIOMICS Gut Microbiome Standard D6331), each run once in contig mode using the same workflow as above (Section 3.1).

Table 4. Case-study samples and inputs. The Zymo mock community and ZymoGut standard are evaluated against curated contig-level labels and a CAMI-style profile; the gut assembly is assessed descriptively in the absence of a ground-truth profile.

Sample ID	Source	Context	Input	Truth assets
zymo_mc	ZymoBIOMICS mock community (Loman Lab assembly)	Even bacterial/fungal mix	contigs	contig labels + CAMI profile
gut_case	MGnify MGYS00006849; SRS9791096; SRR15489027; ERZ24911249; MGYA00794604	Human stool metagenome	contigs	none (top-taxa comparison)
zymogut	ZymoBIOMICS Gut Microbiome Standard D6331 (MicroBench; Flye assembly of ONT SUP reads, ERR14251410)	Gut mock community (bacteria + fungi + archaea)	contigs	contig labels + CAMI profile

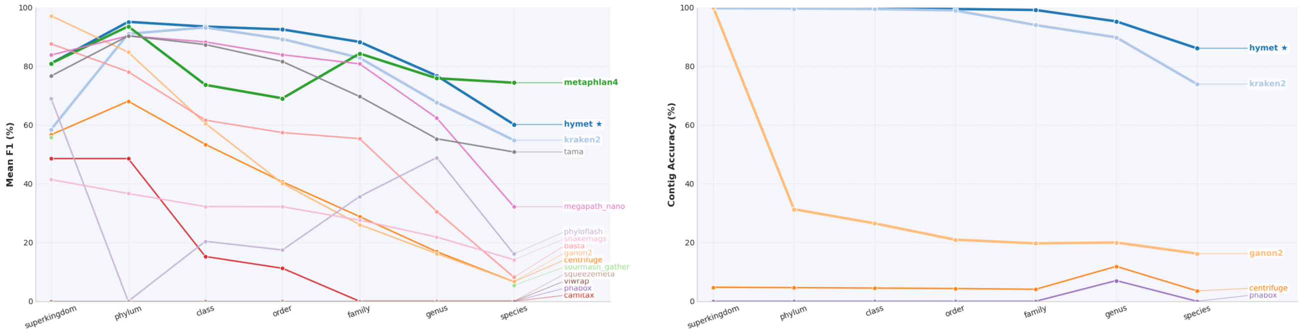

Figure 2. Left: mean F1 by rank (superkingdom to species) across seven datasets. Right: mean contig-level accuracy by rank.

CAMI Benchmark

All quantitative evaluations in this revision are contig based. HYMET also accepts read inputs via the unified CLI (Minimap2 `sr` preset), but a dedicated raw-read benchmark is deferred to a follow-up to avoid mixing modalities. Against contemporary baselines, HYMET maintains high F1 across ranks and taxonomic groups (Supplementary Figure S4); many competitors lose precision and recall at lower ranks, whereas HYMET preserves balanced performance into genus and species, particularly for microbes and small eukaryotes. Viruses remain the most sensitive to divergence, with modest drops at intermediate ranks, but species-level accuracy remains competitive.

We benchmarked HYMET and several contig-mode classification tools using diverse CAMI assembly datasets. Figures 2 and 3 summarize the classification performance at different taxonomic ranks across these tools.

Figure 2 (top) shows that HYMET consistently outperforms most other tools at various taxonomic ranks, particularly from family up to class. While MetaPhlAn 4 achieves high species-level accuracy, it underperforms at intermediate ranks. Index-based classifiers like Kraken 2 and Centrifuge demonstrate reduced accuracy at lower taxonomic ranks due to lower recall rates. Figure 2 (bottom) highlights the stability and consistency of HYMET's contig-level accuracy across ranks, contrasting sharply with tools relying solely on k-mer indices, which exhibit more variability at lower ranks. Only tools that emit per-contig classifications (HYMET, Kraken 2, Centrifuge, Ganon 2, etc.) appear in the contig-accuracy panel because marker-based profilers and read-focused workflows do not produce contig-level outputs.

Profile-distance metrics reinforce HYMET's advantage. As shown in Figure 3 (top), HYMET attains the lowest Bray–Curtis dissimilarity at all ranks and the lowest or near-lowest L1 at most ranks (with genus, species, and superkingdom showing narrow leads by MegaPath-Nano, MetaPhlAn 4, and phyloFlash, respectively). This indicates tighter abundance estimates than competing profilers overall. The heatmap in Figure 3 (bottom) illustrates how this translates into rank-wise accuracy breadth, particularly below the family level where index-only tools lose recall. The heatmap is restricted to tools that emit per-contig assignments (HYMET and the index-only classifiers Kraken 2, Centrifuge, and Ganon 2) because marker-based profilers (e.g., MetaPhlAn 4) and pipeline workflows (e.g., TAMA, SqueezeMeta) report profiles only and do not provide per-contig labels.

Table 5. CAMI profile-distance summary (mean across taxonomic ranks and assemblies). Lower values indicate improved alignment with CAMI truth profiles.

Tool	Mean L1 (pct pts)	Mean Bray–Curtis (%)	Mean F1 (%)
HYMET	36.35	33.88	83.89
MetaPhlAn 4	45.21	78.34	78.83
Kraken 2	45.21	78.96	76.76
TAMA	44.76	77.17	73.11
MegaPath-Nano	45.10	77.95	74.53

Table 6. CAMI summary: mean F1 (%) at genus, species, and across all ranks (Avg F1), mean precision/recall at species, mean wall time (s) and mean peak memory (GB) across seven datasets (canonical multi-tool suite; tight candidate cap).

Tool	Genus F1	Species F1	Avg F1	Species Precision	Species Recall	Wall time	Peak GB
HYMET	76.75	60.18	83.89	62.59	62.00	115.93	6.24
MetaPhlAn 4	75.90	74.38	78.83	75.46	77.92	146.54	18.76
Kraken 2	67.68	54.81	76.76	69.40	47.19	39.86	10.95
TAMA	55.31	50.81	73.11	79.52	40.14	55.61	16.88
MegaPath-Nano	62.38	32.19	74.53	45.63	25.54	24.11	10.20

Table 5 complements these plots: HYMET lowers mean L1 deviation by roughly nine percentage points relative to Kraken 2 and MetaPhlAn 4 while retaining the strongest average F1. Tools optimized for speed, such as MegaPath-Nano, exhibit higher profile distances despite competitive F1 values, highlighting the trade-off between coarse abundance estimates and precise community reconstruction.

Table 6 summarizes these performance trends. Overall, HYMET delivered the highest genus-level F1 score (76.75%), a competitive species-level F1 score (60.18%), and the strongest overall average across ranks (83.89%) driven by balanced precision and recall (62.59% / 62.00%). MetaPhlAn 4 excelled at species-level accuracy (74.38%, precision 75.46%, recall 77.92%) but lagged at intermediate ranks, yielding a lower overall F1 average (78.83%). Kraken 2 and MegaPath-Nano completed faster but showed reduced sensitivity at lower ranks (species F1: 54.81% and 32.19%; averages 76.76% and 74.53%) with skewed precision/recall. TAMA balanced precision (79.52%) against higher memory usage (16.88 GB) and obtained an overall average of 73.11%. Together with Figure 2, these results show that HYMET's hybrid design sustains recall into lower ranks without sacrificing

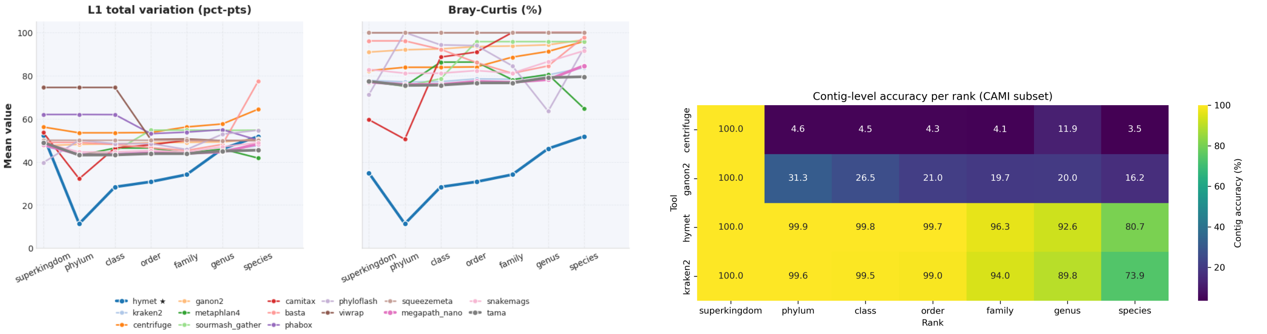

Figure 3. Left: mean L1 total variation and Bray–Curtis dissimilarity by rank across the seven CAMI assembly datasets. Right: per-rank CAMI accuracy heatmap for the profilers benchmarked.

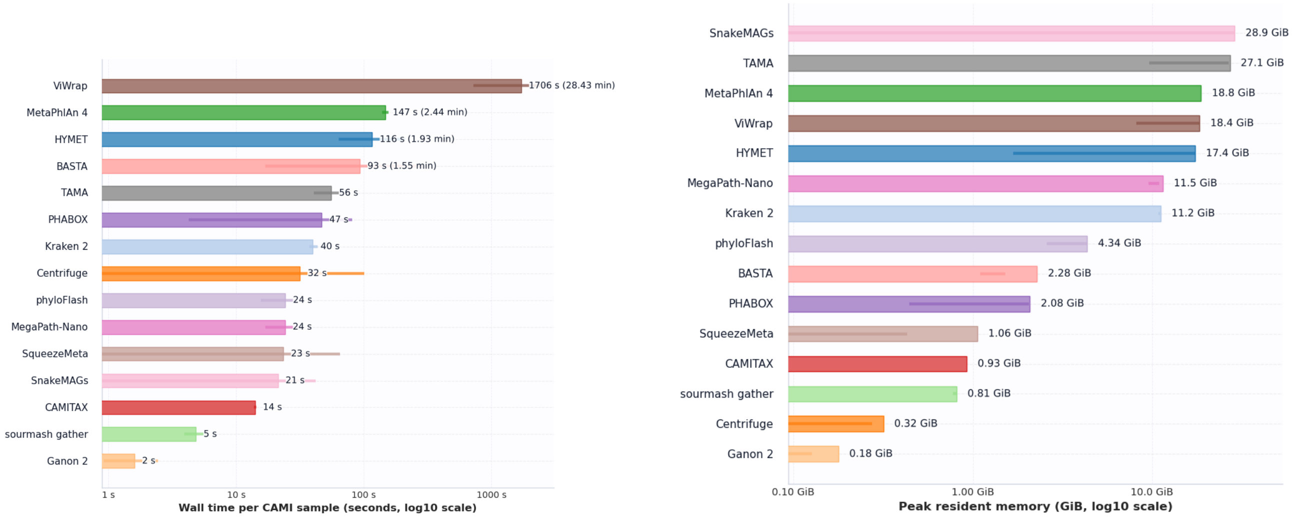

Figure 4. CAMI resource envelopes. Left: aggregated wall-clock time per tool. Right: peak resident memory per tool. Both across the seven datasets (canonical multi-tool suite; tight candidate cap).

precision or inflating resource costs. Per-dataset breakdowns are provided in Supplementary Table S18.

Computational Efficiency

We report resource usage for two CAMI suites that share the same datasets but differ in the candidate-reference budget. In the canonical multi-tool suite (tight candidate cap with species deduplication), HYMET builds smaller caches and averages approximately 116 seconds wall-clock and 6.2 GB peak resident memory across the seven datasets (Figure 4; Table 6). Baselines in this suite illustrate distinct speed–memory–accuracy trade-offs (e.g., MetaPhlAn 4: 147 s, 18.8 GB; Kraken 2: 40 s, 11.0 GB; MegaPath-Nano: 24 s, 10.2 GB; TAMA: 56 s, 16.9 GB).

To isolate input-modality effects, the HYMET-only reads-vs-contigs suite deliberately expands the candidate budget so both modes reuse the same, larger cache. Under this regime, HYMET's contig runs average 361 seconds and 17.37 GB peak memory, while the synthetic-read runs average 334 seconds and 17.36 GB (Supplementary Figure S7; Table 7). The near-identical memory stems from the shared cache; the modest runtime delta arises primarily from Minimap2 presets (`asm10` for contigs vs `sr` for reads), not from differences in the search space.

The case studies align with the expanded-budget envelope. The MGnify gut assembly and the ZymoBIOMICS mock community each completed in 4–5 minutes with 17.4 GB peak memory on the reference machine (Table 10), consistent with the reads-vs-contigs suite and illustrating predictable behavior on real assemblies. In practice, users can choose between these profiles by selecting a tighter candidate cap (minutes-scale, lower memory) or an expanded budget (longer runs with larger caches that can improve sensitivity). Install footprint remains 2.82 GB; dynamically downloaded references typically add 10–50 GB (disk-footprint details are provided in the Supplementary Material).

Mutation Resilience

Beyond classification accuracy on unmodified references, robustness to sequence divergence is relevant for real-world metagenomes. HYMET's performance proves exceptionally stable under varying mutation rates (0–30%), outperforming all benchmarked tools in both accuracy and consistency (Figure 5). Viral classification shows a progressive decline at extreme mutations (F1 scores approximately 0.5 at thirty percent), while archaea, invertebrates and fungi maintain F1 scores over 0.9. Other groups show only minor, non-significant reductions, staying above 0.8 (Supplementary Figure S5). This contrasts with competing tools, where their scores decline as the mutation rate increases (Supplementary Figures S1–S2).

HYMET Read vs Contig Modes

Supplementary Figures S6 and S7 provide a detailed comparison between HYMET's contig-based workflow and its synthetic-read workflow across CAMI assembly datasets, highlighting differences in rank-wise performance and computational resource usage. Aggregated metrics that support these visual comparisons are summarized comprehensively in Table 7.

As illustrated in Supplementary Figure S6, both workflows exhibit closely matched F1 scores across taxonomic ranks. However, the synthetic-read approach demonstrates modestly improved abundance distance metrics at intermediate and lower ranks, as evidenced by lower L1 total variation and Bray–Curtis dissimilarity scores.

The resource usage depicted in Supplementary Figure S7 confirms negligible differences between the two workflows in terms of wall-clock time and memory consumption. This similarity arises because both

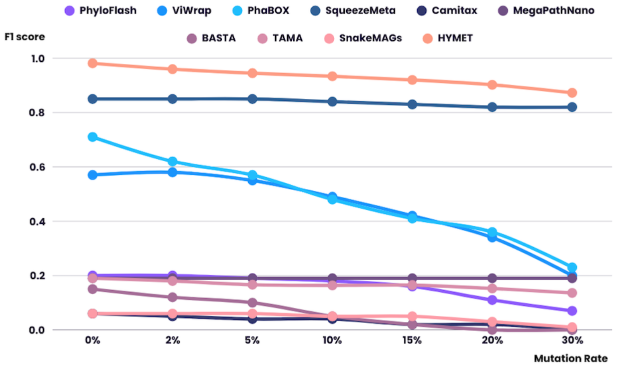

Figure 5. Performance of the state-of-the-art tools and HYMET as the mutation rate increases. The x-axis represents the mutation rate (ranging from 0% to 30%), while the y-axis shows the F1 score. Each curve on the graph corresponds to a different tool.

Table 7. HYMET contig vs read comparison across seven CAMI assembly datasets: mean precision/recall/F1, mean L1 total variation (percentage points), Bray–Curtis (%), wall-clock time (s), and peak resident memory (GB) (HYMET-only suite; expanded candidate budget).

Mode	Precision	Recall	F1	L1	Bray–Curtis	Wall time	Peak GB
HYMET (contigs)	82.15	89.90	84.41	34.55	32.09	361.19	17.37
HYMET (reads)	80.05	90.57	83.37	28.63	26.49	333.61	17.36

modes employ identical downstream reference database construction, alignment strategies, and classification methodologies.

Table 7 quantifies these observations, showing that while the synthetic-read workflow achieves comparable recall and overall F1 scores relative to the contig-based workflow (F1: 83.37 vs. 84.41), it provides slightly improved abundance distances (L1: 28.63 vs. 34.55; Bray–Curtis: 26.49 vs. 32.09). Furthermore, both workflows demonstrate similar resource efficiency in terms of execution time (333.61 vs. 361.19 seconds) and memory usage (17.36 vs. 17.37 GB), illustrating the robustness of HYMET's pipeline across different input modalities.

Gut, Zymo, and ZymoGut Case-study Results

To extend the evaluation beyond CAMI benchmarks, we analyzed three real-world metagenomic datasets: the human gut assembly from MGnify, the ZymoBIOMICS mock community from the Loman Lab, and the ZymoBIOMICS Gut Microbiome Standard (D6331). Figure 6 illustrates the primary taxonomic profiles for the gut and Zymo samples; detailed abundance heatmaps are provided in Supplementary Figures S8 and S9. Computational resource usage for all three analyses is summarized in Table 10.

At the contig level, HYMET assigned species/strain labels to 82.82% of gut contigs; in the Zymo mock, HYMET produced species-level labels for 75.00% of predicted contigs (54/72), and among truth-matched contigs (n=61) exactly matched the curated species for 44.26% (27/61; 56.25% of species-assigned), consistent with genus-level substitutions.

In the human gut sample, HYMET identified a microbiome predominantly composed of Bacillota (Firmicutes; 82.09%), followed by significant contributions from Pseudomonadota (9.59%) and Actinomycetota (8.17%) at the phylum level. At the class level, Clostridia strongly dominated (74.54%), particularly represented by the orders Lachnospirales (36.68%) and Eubacteriales (20.87%), with Lachnospiraceae notably prevalent at the family level. These findings reflect typical adult gut microbiomes characterized by obligate anaerobic bacteria. Among the identified species, the most abundant were *Clostridia bacterium UC5.1-1D4* (12.44%), *[Clostridium] scindens* (9.49%), *Coprococcus phoceensis* (6.85%), *Longicatena caecimuris* (6.53%), *Roseburia intestinalis* (6.21%), and *Ruthenibacterium lactatiformans* (5.61%). Additionally, lower-level signals such as *Escherichia sp. KTE172* (5.47%) were also detected (Figure 6(a)). The gut community heatmap highlighted dense abundance clusters within the Lachnospiraceae and Oscillospiraceae families, reinforcing these results (Supplementary Figure S8).

Table 8. Zymo bacterial species: truth (renormalized within Bacteria) versus HYMET profile. Notes indicate genus-level matches where the exact species differs.

Species	Within-Bacteria (%)		Note
	Truth	HYMET	
Escherichia coli	18.15	22.22	exact
Salmonella enterica	21.20	12.50	exact
Bacillus subtilis	16.33	15.28	genus match (B. spizizenii)
Listeria monocytogenes	12.12	13.89	exact
Enterococcus faecalis	11.54	8.33	exact
Staphylococcus aureus	11.23	9.72	exact
Limosilactobacillus fermentum	8.23	6.94	exact
Pseudomonas aeruginosa	1.20	4.17	genus match (Pseudomonas sp.)

The Zymo mock community comprises ten known organisms, including eight bacterial species and two yeasts (*Saccharomyces cerevisiae* and *Cryptococcus neoformans*). Although yeasts represent 46.80% of the truth profile, in this run HYMET did not recover the eukaryotic component, and the reported profile is restricted to bacterial taxa (53.20%). Within bacteria, HYMET accurately identified major species such as *Escherichia coli* (22.22%), *Listeria monocytogenes* (13.89%), and *Salmonella enterica* (12.50%). Minor genus-level substitutions occurred: *Bacillus* spizizenii in place of *B. subtilis*, and *Pseudomonas* sp. in place of *P. aeruginosa*. Table 8 compares the bacterial abundances (renormalized within bacteria) to HYMET's predictions, showing six exact species matches and two genus-level substitutions. The Zymo abundance patterns concentrate within Enterobacterales and Bacillales, consistent with Figure 6(b) and Supplementary Figure S9.

The ZymoGut D6331 assembly (535 contigs, 534 classified) was evaluated against contig-derived ground truth spanning 15 species from three domains of life. At the genus level, HYMET achieved a Pearson correlation of $r = 0.998$ and a Bray–Curtis dissimilarity of 0.04 (L1 total variation = 0.08), indicating near-perfect abundance estimation. Of 14 expected genera, 11 were correctly detected (78.6% recall). The three dominant genera, *Escherichia* (34.4%), *Candida* (30.8%), and *Saccharomyces* (29.0%), were classified within 1.5 percentage points of the truth. The three undetected genera (*Clostridioides*, *Lactobacillus*, *Roseburia*) were each represented by $\leq$1 contig in the Flye assembly, making their detection stochastic at the assembly level. Notably, HYMET correctly identified taxa from all three domains (Bacteria, Eukaryota, Archaea), including *Methanobrevibacter* at 1.5% abundance (Table 9, Figure 7).

Species-level accuracy was lower ($r = 0.130$), driven by misassignment between closely related sister species within the same genus. *Candida albicans* contigs (165 in truth) were predominantly classified as *C. dubliniensis* (155 contigs), and *Saccharomyces cerevisiae* contigs (155 in truth) were assigned to *S. paradoxus* (128 contigs). This sister-species confusion is a well-documented limitation across metagenomics classifiers for organisms sharing >95% average nucleotide identity and does not affect genus-level assignments: zero contigs were assigned to an incorrect genus (Supplementary Figure S10).

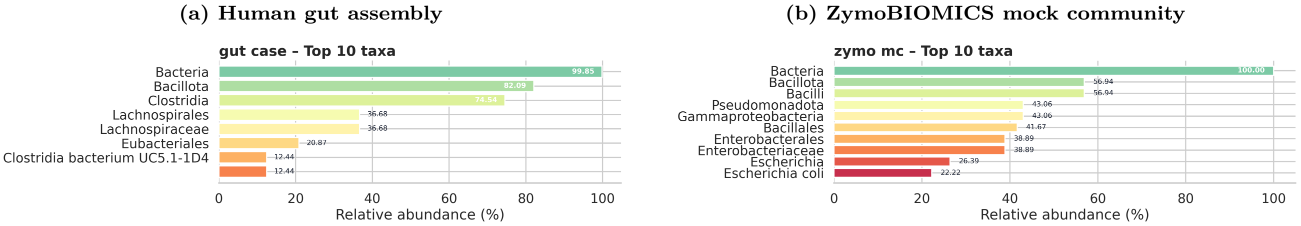

Figure 6. Top-taxa profiles for two of the three real-world case studies. (a) Human gut assembly: most abundant ranks from superkingdom through species. (b) ZymoBIOMICS mock community: most abundant ranks from superkingdom through species. The ZymoGut D6331 case study is presented separately in Figure 7.

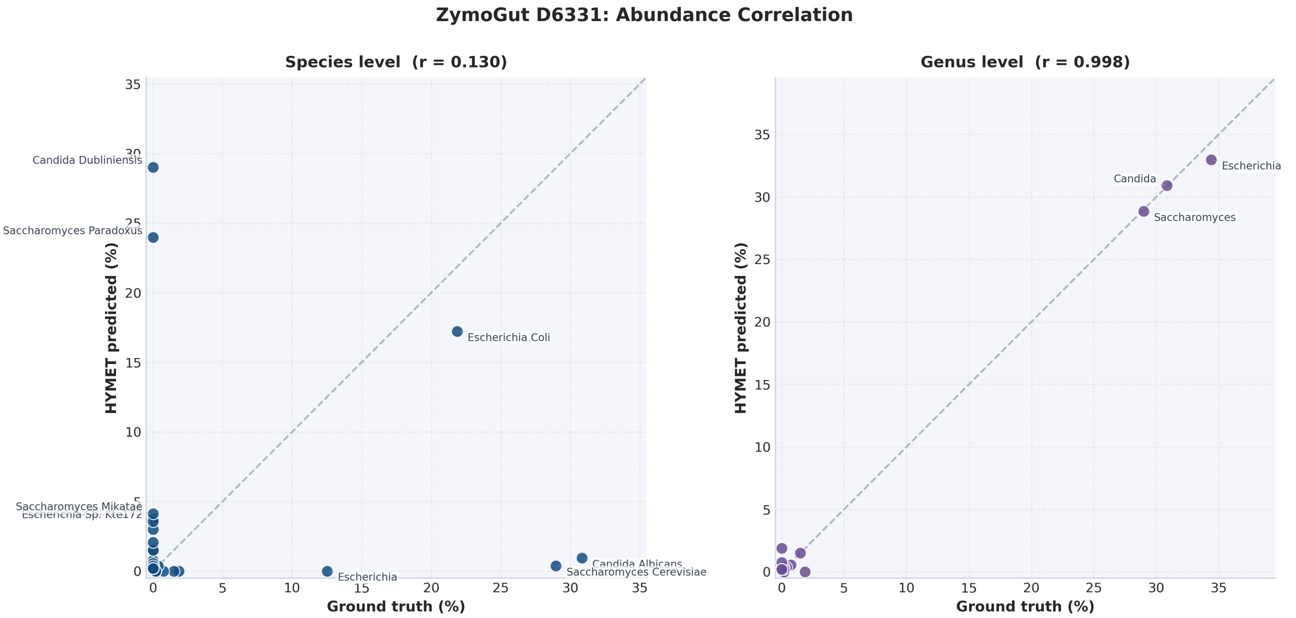

Figure 7. Abundance correlation between ground truth and HYMET predictions for the ZymoGut D6331 mock community. Left: species-level ($r = 0.130$), showing sister-species confusion (e.g., *C. albicans* misclassified as *C. dubliniensis*). Right: genus-level ($r = 0.998$), where all major genera fall on the identity line. Dashed line indicates perfect agreement.

Table 9. ZymoGut D6331 genus-level comparison: ground truth (contig-derived) versus HYMET predictions. Diff is predicted minus truth in percentage points.

Genus	Truth (%)	HYMET (%)	Diff (pp)
Escherichia	34.39	32.96	−1.43
Candida	30.84	30.90	+0.06
Saccharomyces	28.97	28.84	−0.13
Clostridioides	1.87	0.00	−1.87
Methanobrevibacter	1.50	1.50	+0.00
Prevotella	0.75	0.56	−0.19
Bifidobacterium	0.37	0.37	+0.00
Akkermansia	0.19	0.19	+0.00
Bacteroides	0.19	0.19	+0.00
Faecalibacterium	0.19	0.19	+0.00
Fusobacterium	0.19	0.37	+0.19
Veillonella	0.19	0.19	+0.00

Computationally, all three datasets exhibited similar performance metrics. HYMET completed the analyses efficiently in approximately 4–5 minutes each, with peak memory usage consistently around 17.4 GB, showcasing the robustness and scalability of the method across diverse metagenomic contexts (Table 10).

5 Zymo Ablation Results

To assess robustness to incomplete references, we ran an ablation study on the Zymo dataset using the canonical suite, progressively removing species/strain-level entries and re-indexing at each level. Across ablation levels, runtime and memory remained stable (∼4.2–4.5 minutes, ∼17.4 GB). Classification quality degrades as expected at finer ranks: the share of species/strain assignments falls from 75.00% to 64.52%, while higher/unknown rises from 25.00% to 35.48%; genus F1 drops from 88.89% to 58.82% and species F1 from 57.14% to 0.00% at full ablation.

Table 10. Case-study runtime and memory summary on the reference machine.

Sample	Wall time (s)	Peak RSS (GB)
gut_case	258.32	17.41
zymo_mc	255.73	17.42

Table 11. Zymo ablation summary. Levels indicate the ablation proportion; totals are number of contigs classified.

Level (%)	Total classified	Species/strain (%)	Higher/unknown (%)	Genus F1 (%)	Species F1 (%)
0	72	75.00	25.00	88.89	57.14
25	69	72.46	27.54	88.89	57.14
50	71	73.24	26.76	88.89	43.48
75	66	66.67	33.33	73.68	13.79
100	62	64.52	35.48	58.82	0.00

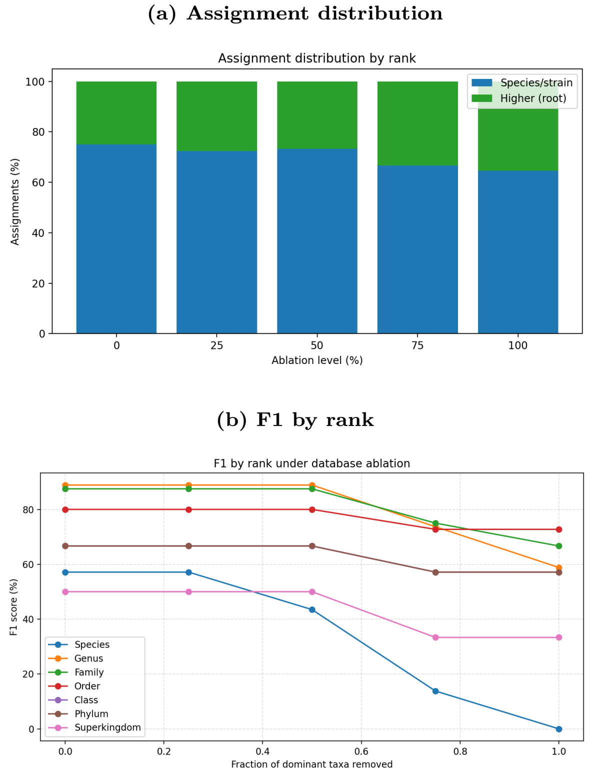

Figure 8. Zymo ablation experiment. (a) Distribution of contig assignments by rank group across ablation levels; species/strain share decreases while higher/unknown rises. (b) F1 by taxonomic rank under progressive reference removal (0%–100%).

Figure 8(a) shows a gradual reweighting from species/strain toward higher ranks as references are withheld: species/strain drops from 75.00% to 64.52%, while higher/unknown rises from 25.00% to 35.48%; the number of classified contigs declines modestly ($72 \to 62$). Genus and family contributions remain stable through 50% ablation and only drift at $\geq$75%, indicating HYMET backs off to appropriate higher ranks rather than emitting incorrect fine-grained labels when exact references are missing.

Rank-wise accuracy (Figure 8(b)) remains flat at coarse levels, while genus F1 stays high through 50% (88.89%) and then declines at 75% (73.68%) and 100% (58.82%). Species F1 degrades from 57.14% to 0.00% under full ablation, reflecting the intended removal of discriminative references. Throughout, runtime and memory are effectively unchanged (~4.2–4.5 minutes; ~17.4 GB), isolating reference incompleteness as the driver of accuracy loss rather than compute differences.

6 Discussion

HYMET effectively addresses significant challenges in metagenomic analysis by integrating adaptive MinHash-based pre-filtering with precise alignment and a coverage-weighted Lowest Common Ancestor (LCA) classification. This hybrid design builds upon previous approaches such as Metalign's combination of CMash and Minimap2, yet it uniquely introduces an adaptive candidate selection method, dynamically generated cross-domain reference databases, and an evidence-weighted LCA step. Compared to fixed-threshold methods, such as CAMITAX, HYMET's adaptive strategy enhances sensitivity to divergent organisms, avoiding the exclusion of potentially relevant candidates and ensuring broader domain applicability [4, 70].

Our evaluations across diverse CAMI benchmarks demonstrate HYMET's robust accuracy, computational efficiency, and stable performance across taxonomic ranks. Specifically, HYMET achieved a mean F1 of 83.89% across ranks, a strong genus-level F1 of 76.75%, and a competitive species-level F1 of 60.18%, while maintaining low computational resource demands (average runtime of 116 seconds and a mean peak memory of approximately 6.2 GB across samples, ranging from under 2 GB for low-complexity panels to approximately 17 GB for the most diverse communities). Unlike marker-based tools such as MetaPhlAn 4, which excel at species-level assignments but sacrifice intermediate-rank accuracy and per-contig labeling capabilities, HYMET balances precision and recall consistently across multiple ranks, which is crucial for downstream metagenomic workflows such as genome binning [71, 15, 23].

HYMET's adaptive cache management strategy allows predictable and flexible resource usage. Both contig and synthetic-read analyses converged in memory usage when sharing reference caches, differing slightly only in runtime due to alignment parameter presets. Practically, users can thus optimize for rapid exploratory analyses or deeper comparative studies across related samples by adjusting candidate cache parameters without compromising accuracy.

Importantly, HYMET demonstrates resilience against genetic mutations, maintaining robust performance (F1 $\geq$ 0.8) for diverse taxonomic groups even at mutation rates up to 30%. While viral sequences experienced a notable decrease in accuracy (F1 $\approx$ 0.5 at 30%), this likely reflects genuine biological and database limitations rather than methodological shortcomings. The stability in performance arises primarily from the optimized choice of screening parameters (smaller k-mers and larger sketch sizes for divergent genomes) and the robust seed-chain-extend alignment method employed by Minimap2 [20, 41].

The ZymoBIOMICS case study provided additional insight into HYMET's limitations. While bacterial taxa were accurately classified (six exact matches, two genus-level substitutions), the method failed to recover yeast species, which represented a significant proportion of the mock community. The systematic ablation experiment further clarified this behavior, showing HYMET appropriately adjusts assignments to higher taxonomic ranks when species-level references are incomplete or missing. This indicates that future versions could benefit significantly from domain-specific adjustments to candidate thresholds, particularly enhancing sensitivity towards underrepresented groups like yeasts. The ZymoGut D6331 evaluation reinforced these findings on a more complex, gut-relevant community: genus-level classification was near-perfect ($r = 0.998$, Bray–Curtis = 0.04) across bacteria, fungi, and archaea, while species-level accuracy was limited by sister-species confusion between organisms sharing >95% nucleotide identity (*C. albicans*/*C. dubliniensis*, *S. cerevisiae*/*S. paradoxus*), a well-documented challenge across state-of-the-art classifiers.

A notable operational challenge identified was related to reference database completeness and retrieval reliability. The hybrid reference databases, combining older public sketches with newly generated local sketches, exhibited a non-negligible retrieval failure rate (6.04%), significantly impacting classification accuracy, particularly at lower taxonomic levels. This emphasizes the importance of maintaining continuously updated, comprehensive databases, systematically refreshing manifests, and implementing robust checksum and fallback protocols to mitigate data retrieval issues [67, 17].

HYMET demonstrated robust accuracy across bacteria, archaea, fungi, and small eukaryotes, emphasizing its suitability across diverse microbial communities. However, challenges remain in classifying more complex eukaryotic organisms due to inherent genome complexity, substantial intra-species diversity, and biases towards well-studied taxa in reference databases [72]. Similarly, the polyphyletic and rapidly evolving nature of viral genomes underscores the importance of regularly updated, comprehensive databases to maintain accurate viral taxonomic classification [73, 74].

Future improvements should focus on directly addressing identified limitations: enhancing domain-specific candidate thresholds (particularly for viruses and eukaryotes), implementing scheduled updates of sketch databases enriched with underrepresented taxa, refining the weighted LCA with explicit rules for resolving multi-mapped alignments and establishing minimum evidence thresholds. Integrating uncertainty quantification methods, such as bootstrap-

ping or replicate analyses, will further enhance the reliability and practical applicability of HYMET.

7 Conclusion

HYMET advances metagenomic classification by integrating adaptive MinHash screening, precise Minimap2 alignment, and a coverage-weighted Lowest Common Ancestor algorithm. Evaluations across diverse CAMI benchmarks demonstrate that HYMET achieves robust accuracy, consistently outperforming or matching existing methods at multiple taxonomic ranks, notably with an average F1 score of 83.89%, including 76.75% at the genus level and 60.18% at the species level. This high performance is maintained even under substantial genetic divergence, indicating strong mutation resilience.

Real-world validations further confirm HYMET's practical value, successfully identifying expected bacterial communities from human gut and ZymoBIOMICS mock samples, and achieving near-perfect genus-level accuracy on the ZymoGut D6331 gut mock community. Computationally efficient and lightweight, HYMET's adaptive and dynamic caching strategy ensures reproducible and resource-predictable analyses suitable for diverse deployment contexts.

While current results illustrate excellent overall accuracy and scalability, future enhancements could include domain-specific reference optimizations, explicit handling of multi-mapping ambiguities, and integration of machine learning classifiers to further improve lower-rank discrimination. Such developments would position HYMET as a versatile platform, supporting reliable, efficient, and scalable metagenomic investigations across various biological contexts.

8 Availability of source code and requirements

- Project name: HYMET (Hybrid Metagenomic Tool)
- Project home page: `https://github.com/ieeta-pt/HYMET`
- bio.tools ID: hymet
- RRID: SCR_026916
- Operating system(s): Linux
- Programming language: Python (primary), Perl (legacy), Bash
- Other requirements: Docker or Apptainer/Singularity; Conda/Mamba
- License: MIT.

9 Data Availability

The supplementary material describes the full reproducibility workflow (environment setup, scripts, execution logs, and additional figures). Digital resources used in the manuscript are listed below.

- HYMET source code, CAMI/reads-versus-contigs/case manifests, runtime logs, and aggregated TSV/figure outputs are versioned in the project repository (see availability of source code section; `results/`, `bench/`, and `case/` subdirectories).
- The Syst_Review repository retains the systematic benchmark harness for third-party tools, including installation recipes and evaluation scripts [75].
- The Mash sketch databases used for candidate selection (`sketch1.msh`, `sketch2.msh`, `sketch3.msh`) are deposited at Zenodo [76]; checksums are mirrored in the repository (`sketch\_sha256.txt`).
- Numerical data underlying Figures 2–8 and Supplementary Figures S6–S10 are deposited at Zenodo [77], with a detailed README describing each file.
- CAMI contig assemblies used in the benchmark are enumerated in `bench/cami_manifest.tsv`; running `bench/fetch_cami.sh` downloads the official CAMI I sample_0 bundle from the Publisso mirror [78], extracts the required assets into `/data/cami/`, caches the archive, and regenerates the lightweight subsets (`cami_i_*`, `cami_ii_*`) via `tools/generate_cami_subsets.py` so every benchmark run starts from the same inputs.
- The test and validation dataset (26,203 genomes, 14.76 GB) was derived from the NCBI RefSeq Assembly database [79]; assembly summary files and scripts for replication are provided in Supplementary Material Section 3.
- Reference genomes for dynamic database construction are retrieved from the NCBI Assembly database [80] using GCF and GCA accession numbers identified through Mash screening. GTDB r202 genomes included in the sketch databases are available from the Genome Taxonomy Database [81].
- Case-study inputs can be fetched with `case/fetch_case_data.sh`, which retrieves the Zymo mock assembly [82] and the MGnify gut assembly [83] before staging them under `/data/case/`. The manifest `case/manifest.tsv` records the same paths for reference.
- Zymo ablation outputs (candidate lists, cached references, evaluation tables, figures) are versioned under `results/ablation/canonical/run_20251031T191804Z/`; the workflow can be rerun with `case/run_ablation.sh` as documented in Supplementary Section 6.
- ZymoGut D6331 case-study inputs comprise Oxford Nanopore SUP basecalls from the MicroBench collection (ENA accession `ERR14251410`) assembled with Flye, and manufacturer reference genomes from the Zymo Research D6331 RefSeq package. Ground-truth labels and evaluation scripts are provided in `case/zymogut/`; analysis outputs are versioned under `results/cases/zymogut/`.

10 Additional Files

Supplementary Tab. S1. On-disk resource footprint (install + references) for every benchmarked tool in the canonical CAMI environment.

Supplementary Tab. S2. Provenance and composition of the reference databases used by each tool, including shared corpora and official releases.

Supplementary Tab. S3 to S10. Per-domain benchmarking tables (viruses, archaea, bacteria, fungi, protozoa, plants, invertebrates, vertebrates) reporting precision, recall, and F1 across taxonomic ranks at 0% mutation.

Supplementary Tab. S11 to S17. HYMET-only benchmarking tables summarizing precision, recall, and F1 across taxonomic ranks under mutation rates from 0% to 30%.

Supplementary Tab. S18. Per-dataset evaluation summary (Avg F1, Genus F1, Species F1) for the five principal tools across each of the seven CAMI assembly datasets.

Supplementary Tab. S19. Full genus-level comparison table for the ZymoGut D6331 case study, listing all 14 expected and 8 false-positive genera with truth and predicted abundances.

Supplementary Fig. S1 and S2. Line plots showing how tool performance varies with mutation rate across taxonomic levels for each biological domain.

Supplementary Fig. S3. Scatter plots relating execution time (hours) to F1 score (0.0–1.0) for all evaluated tools, presented per domain.

Supplementary Fig. S4. F1 scores at 0% mutation for all tools from the mutation-study tool set, grouped by taxonomic domain.

Supplementary Fig. S5. HYMET per-domain mutation resilience: F1 across taxonomic levels (kingdom to species) for each of nine

biological groups under mutation rates 0%–30%.

Supplementary Fig. S6. Comparison of HYMET contig vs. synthetic-read workflows: mean F1 by rank and abundance distances (L1, Bray–Curtis).

Supplementary Fig. S7. Resource usage comparison between HYMET contig and read workflows: wall-clock time and peak memory.

Supplementary Fig. S8. Abundance heatmap for the human gut case study, showing relative abundance across taxonomic ranks.

Supplementary Fig. S9. Abundance heatmap for the ZymoBIOMICS mock community case study, showing relative abundance across taxonomic ranks.

Supplementary Fig. S10. Genus-level abundance comparison (Cleveland dot plot) for the ZymoGut D6331 case study, showing ground truth and HYMET predicted abundances for all detected genera.

10.1 List of abbreviations

BASTA: BAsic Sequence Taxonomy Annotation;
BLAST: Basic Local Alignment Search Tool;
CAMI: Critical Assessment of Metagenome Interpretation;
CAMITAX: CAMI TAXonomy (tool for taxon labels);
CMash: Containment MinHash;
CPU: Central Processing Unit;
CSV: Comma-Separated Values;
DOI: Digital Object Identifier;
F1: Harmonic mean of precision and recall;
FCT: Fundação para a Ciência e a Tecnologia;
GCA: GenBank assembly accession;
GCF: RefSeq assembly accession;
GTDB: Genome Taxonomy Database;
HYMET: Hybrid Metagenomic Tool;
LCA: Lowest Common Ancestor;
NCBI: National Center for Biotechnology Information;
PAF: Pairwise mApping Format;
RefSeq: NCBI Reference Sequence database;
RSS: Resident Set Size;
SSU rRNA: Small Subunit ribosomal RNA;
TAMA: Taxonomy Analysis pipeline for metagenome using Meta-Analysis;
TaxID: Taxonomy Identifier;
TSV: Tab-Separated Values.

Ethics Approval and Consent to Participate

Not applicable. This study involved only computational analyses of publicly available genomic data; no human or animal subjects were involved.

Consent for Publication

Not applicable.

Competing Interests

The authors declare that they have no competing interests.

Funding

This work has received funding from the FCT (Foundation for Science and Technology) under unit 00127-IEETA and through the project Advanced Genomic Data Processing in Portuguese FEGA Node (ref. 2023.14342.CPCA.A1; DOI: 10.54499/2023.14342.CPCA.A1). J.M.S. has received funding from the European Commission under grant agreement 101081813 (Genomic Data Infrastructure).

Author Contributions

Using the CRediT taxonomy: **J.M.S.**: Writing – original draft, Writing – review & editing, Software, Methodology, Validation, Visualization, Data curation. **I.M.**: Writing – original draft, Software, Methodology, Data curation. **J.R.A.**: Supervision. All authors read and approved the final manuscript.

Acknowledgements

The authors thank the CAMI initiative for providing standardised benchmark datasets and the NCBI, GTDB, and Loman Lab for maintaining the public reference resources used in this study.

References

1. Kim D, Song L, Breitwieser FP, Salzberg SL. Centrifuge: rapid and sensitive classification of metagenomic sequences. Genome research 2016;26(12):1721–1729. https://doi.org/10.1101/gr.210641.116.
2. Simon HY, Siddle KJ, Park DJ, Sabeti PC. Benchmarking metagenomics tools for taxonomic classification. Cell 2019;178(4):779–794. https://doi.org/10.1016/j.cell.2019.07.010.
3. Wood DE, Salzberg SL. Kraken: ultrafast metagenomic sequence classification using exact alignments. Genome biology 2014;15(3):1–12. https://doi.org/10.1186/gb-2014-15-3-r46.
4. Bremges A, Fritz A, McHardy AC. CAMITAX: Taxon labels for microbial genomes. GigaScience 2020;9(1):giz154. https://doi.org/10.1093/gigascience/giz154.
5. Kim N, Ma J, Kim W, Kim J, Belenky P, Lee I. Genome-resolved metagenomics: a game changer for microbiome medicine. Experimental & Molecular Medicine 2024;56(7):1501–1512. https://doi.org/10.1038/s12276-024-01262-7.
6. Mallawaarachchi V, Lin Y. Accurate binning of metagenomic contigs using composition, coverage, and assembly graphs. Journal of Computational Biology 2022;29(12):1357–1376. https://doi.org/10.1089/cmb.2022.0262.
7. Ayling M, Clark MD, Leggett RM. New approaches for metagenome assembly with short reads. Briefings in bioinformatics 2020;21(2):584–594. https://doi.org/10.1093/bib/bbz020.
8. Wood DE, Lu J, Langmead B. Improved metagenomic analysis with Kraken 2. Genome biology 2019;20:1–13. https://doi.org/10.1186/s13059-019-1891-0.
9. Lema NK, Gemeda MT, Woldesemayat AA. Recent Advances in Metagenomic Approaches, Applications, and Challenges. Current Microbiology 2023;80(11):347. https://doi.org/10.1007/s00284-023-03451-5.
10. Martins IB, Miguel Silva J, Almeida JR. A comprehensive study of databases to assess the reliability of metagenomic tools. In: 2024 IEEE Conference on Computational Intelligence in Bioinformatics and Computational Biology (CIBCB); 2024. p. 1–6. https://doi.org/10.1109/CIBCB58642.2024.10702118.
11. Xu R, Rajeev S, Salvador LC. The selection of software and database for metagenomics sequence analysis impacts the outcome of microbial profiling and pathogen detection. Plos one

2023;18(4):e0284031. https://doi.org/10.1371/journal.pone.0284031.
12. Breitwieser FP, Lu J, Salzberg SL. A review of methods and databases for metagenomic classification and assembly. Briefings in bioinformatics 2019;20(4):1125–1136. https://doi.org/10.1093/bib/bbx120.
13. Kieser S, Brown J, Zdobnov EM, Trajkovski M, McCue LA. ATLAS: a Snakemake workflow for assembly, annotation, and genomic binning of metagenome sequence data. BMC bioinformatics 2020;21:1–8. https://doi.org/10.1186/s12859-020-03585-4.
14. Tadrent N, Dedeine F, Hervé V. SnakeMAGs: a simple, efficient, flexible and scalable workflow to reconstruct prokaryotic genomes from metagenomes. F1000Research 2022;11. https://doi.org/10.12688/f1000research.128091.2.
15. Tamames J, Puente-Sánchez F. SqueezeMeta, a highly portable, fully automatic metagenomic analysis pipeline. Frontiers in microbiology 2019;9:425882. https://doi.org/10.3389/fmicb.2018.03349.
16. Clarke EL, Taylor LJ, Zhao C, Connell A, Lee JJ, Fett B, et al. Sunbeam: an extensible pipeline for analyzing metagenomic sequencing experiments. Microbiome 2019;7:1–13. https://doi.org/10.1186/s40168-019-0658-x.
17. Chaumeil PA, Mussig AJ, Hugenholtz P, Parks DH, GTDB-Tk: a toolkit to classify genomes with the Genome Taxonomy Database. Oxford University Press; 2020. https://doi.org/10.1093/bioinformatics/btz848.
18. Buchfink B, Xie C, Huson DH. Fast and sensitive protein alignment using DIAMOND. Nature methods 2015;12(1):59–60. https://doi.org/10.1038/nmeth.3176.
19. Kahlke T, Ralph PJ. BASTA–Taxonomic classification of sequences and sequence bins using last common ancestor estimations. Methods in Ecology and Evolution 2019;10(1):100–103. https://doi.org/10.1111/2041-210X.13095.
20. Ondov BD, Treangen TJ, Melsted P, Mallonee AB, Bergman NH, Koren S, et al. Mash: fast genome and metagenome distance estimation using MinHash. Genome biology 2016;17:1–14. https://doi.org/10.1186/s13059-016-0997-x.
21. Menzel P, Ng KL, Krogh A. Fast and sensitive taxonomic classification for metagenomics with Kaiju. Nature communications 2016;7(1):11257. https://doi.org/10.1038/ncomms11257.
22. Callahan BJ, McMurdie PJ, Rosen MJ, Han AW, Johnson AJA, Holmes SP. DADA2: High-resolution sample inference from Illumina amplicon data. Nature methods 2016;13(7):581–583. https://doi.org/10.1038/nmeth.3869.
23. Sim M, Lee J, Lee D, Kwon D, Kim J. TAMA: improved metagenomic sequence classification through meta-analysis. BMC bioinformatics 2020;21:1–17. https://doi.org/10.1186/s12859-020-3533-7.
24. Ounit R, Wanamaker S, Close TJ, Lonardi S. CLARK: fast and accurate classification of metagenomic and genomic sequences using discriminative k-mers. BMC genomics 2015;16(1):1–13. https://doi.org/10.1186/s12864-015-1419-2.
25. Breitwieser FP, Baker DN, Salzberg SL. KrakenUniq: confident and fast metagenomics classification using unique k-mer counts. Genome Biology 2018;19(1):198. https://doi.org/10.1186/s13059-018-1568-0.
26. Piro VC, Dadi TH, Seiler E, Reinert K, Renard BY. ganon: precise metagenomics classification against large and up-to-date sets of reference sequences. Bioinformatics 2020 Jul;36(Suppl_1):i12–i20. https://doi.org/10.1093/bioinformatics/btaa458.
27. Piro VC, Reinert K. ganon2: up-to-date and scalable metagenomics analysis. NAR Genomics and Bioinformatics 2025 07;7(3):lqaf094. https://doi.org/10.1093/nargab/lqaf094.
28. Song L, Langmead B. Centrifuger: lossless compression of microbial genomes for efficient and accurate metagenomic sequence classification. Genome Biology 2024;25(1):106. https://doi.org/10.1186/s13059-024-03244-4.
29. Ulrich JU, Renard BY. Fast and space-efficient taxonomic classification of long reads with hierarchical interleaved XOR filters. Genome Research 2024;34(6):914–924. https://doi.org/10.1101/gr.278623.123.
30. Brown CT, Irber L. sourmash: a library for MinHash sketching of DNA. Journal of Open Source Software 2016;1(5):27. https://doi.org/10.21105/joss.00027.
31. Shang J, Peng C, Liao H, Tang X, Sun Y. PhaBOX: a web server for identifying and characterizing phage contigs in metagenomic data. Bioinformatics Advances 2023;3(1):vbad101. https://doi.org/10.1093/bioadv/vbad101.
32. Shang J, Jiang J, Sun Y. Bacteriophage classification for assembled contigs using graph convolutional network. Bioinformatics 2021;37(Supplement_1):i25–i33. https://doi.org/10.1093/bioinformatics/btab293.
33. Zhou Z, Martin C, Kosmopoulos JC, Anantharaman K. ViWrap: A modular pipeline to identify, bin, classify, and predict viral–host relationships for viruses from metagenomes. Imeta 2023;2(3):e118. https://doi.org/10.1101/2023.01.30.526317.
34. Auslander N, Gussow AB, Benler S, Wolf YI, Koonin EV. Seeker: alignment-free identification of bacteriophage genomes by deep learning. Nucleic acids research 2020;48(21):e121–e121. https://doi.org/10.1093/nar/gkaa856.
35. Gałan W, Bąk M, Jakubowska M. Host taxon predictor-a tool for predicting taxon of the host of a newly discovered virus. Scientific reports 2019;9(1):3436. https://doi.org/10.1038/s41598-019-39847-2.
36. Jiang G, Zhang J, Zhang Y, Yang X, Li T, Wang N, et al. DCiPatho: deep cross-fusion networks for genome scale identification of pathogens. Briefings in Bioinformatics 2023;24(4):bbad194. https://doi.org/10.1093/bib/bbad194.
37. Altschul SF, Gish W, Miller W, Myers EW, Lipman DJ. Basic local alignment search tool. Journal of molecular biology 1990;215(3):403–410. https://doi.org/10.1016/S0022-2836(05)80360-2.
38. Gruber-Vodicka HR, Seah BK, Pruesse E. phyloFlash: rapid small-subunit rRNA profiling and targeted assembly from metagenomes. Msystems 2020;5(5):10–1128. https://doi.org/10.1128/mSystems.00920-20.
39. Truong DT, Franzosa EA, Tickle TL, Scholz M, Weingart G, Pasolli E, et al. MetaPhlAn2 for enhanced metagenomic taxonomic profiling. Nature methods 2015;12(10):902–903. https://doi.org/10.1038/nmeth.3589.
40. Lui WW, Leung AW, Leung HC, Xin Y, Teng JL, Woo PC, et al. MegaPath-Nano: Accurate Compositional Analysis and Drug-level Antimicrobial Resistance Detection Software for Oxford Nanopore Long-read Metagenomics. In: 2020 IEEE International Conference on Bioinformatics and Biomedicine (BIBM) IEEE; 2020. p. 329–336. https://doi.org/10.1109/BIBM49941.2020.9313313.
41. Li H. Minimap2: pairwise alignment for nucleotide sequences. Bioinformatics 2018;34(18):3094–3100. https://doi.org/10.1093/bioinformatics/bty191.
42. Liang X, Zhang J, Kim Y, Ho J, Liu K, Keenum I, et al. ARGem: a new metagenomics pipeline for antibiotic resistance genes: metadata, analysis, and visualization. Frontiers in Genetics 2023;14:1219297. https://doi.org/10.3389/fgene.2023.1219297.
43. Prosperi M, Marini S. Karga: Multi-platform toolkit for k-mer-based antibiotic resistance gene analysis of high-throughput sequencing data. In: 2021 IEEE EMBS International Conference on Biomedical and Health Informatics (BHI) IEEE; 2021. p. 1–4. https://doi.org/10.1109/BHI50953.2021.9508479.
44. LaPierre N, Alser M, Eskin E, Koslicki D, Mangul S. Metalign: efficient alignment-based metagenomic profiling via containment min hash. Genome Biology 2020;21(1):242. https://doi.org/10.1186/s13059-020-02159-0.
45. Olawoye IB, Frost SD, Happi CT. The Bacteria Genome Pipeline

(BAGEP): an automated, scalable workflow for bacteria genomes with Snakemake. PeerJ 2020;8:e10121. https://doi.org/10.7717/peerj.10121.

46. Ondov BD, Starrett GJ, Sappington A, Kostic A, Koren S, Buck CB, et al. Mash Screen: high-throughput sequence containment estimation for genome discovery. Genome biology 2019;20:1–13. https://doi.org/10.1186/s13059-019-1841-x.
47. Baker DN, Langmead B. Dashing: fast and accurate genomic distances with HyperLogLog. Genome biology 2019;20:1–12. https://doi.org/10.1186/s13059-019-1875-0.
48. Besta M, Kanakagiri R, Mustafa H, Karasikov M, Rätsch G, Hoefler T, et al. Communication-efficient jaccard similarity for high-performance distributed genome comparisons. In: 2020 IEEE International Parallel and Distributed Processing Symposium (IPDPS) IEEE; 2020. p. 1122–1132. https://doi.org/10.1109/IPDPS47924.2020.00118.
49. Zhao X. BinDash, software for fast genome distance estimation on a typical personal laptop. Bioinformatics 2019;35(4):671–673. https://doi.org/10.1093/bioinformatics/bty651.
50. Katz LS, Griswold T, Morrison SS, Caravas JA, Zhang S, den Bakker HC, et al. Mashtree: a rapid comparison of whole genome sequence files. Journal of Open Source Software 2019;4(44):10–21105. https://doi.org/10.21105/joss.01762.
51. Broder AZ. On the resemblance and containment of documents. In: Proceedings. Compression and Complexity of SEQUENCES 1997 (Cat. No. 97TB100171) IEEE; 1997. p. 21–29. https://doi.org/10.1109/SEQUEN.1997.666900.
52. Pierce NT, Irber L, Reiter T, Brooks P, Brown CT. Large-scale sequence comparisons with sourmash. F1000Research 2019;8:1006. https://doi.org/10.12688/f1000research.19675.1.
53. Hernández-Salmerón JE, Moreno-Hagelsieb G. FastANI, Mash and Dashing equally differentiate between Klebsiella species. PeerJ 2022;10:e13784. https://doi.org/10.7717/peerj.13784.
54. Hera MR, Liu S, Wei W, Rodriguez JS, Ma C, Koslicki D. Metagenomic functional profiling: to sketch or not to sketch? Bioinformatics 2024;40(Supplement_2):ii165–ii173. https://doi.org/10.1093/bioinformatics/btae397.
55. Wu W, Li B, Chen L, Gao J, Zhang C. A review for weighted minhash algorithms. IEEE Transactions on Knowledge and Data Engineering 2020;34(6):2553–2573. https://doi.org/10.1109/TKDE.2020.3021067.
56. Sánchez-Reyes A, Fernández-López M. Sketched reference databases for genome-based taxonomy and comparative genomics. Brazilian Journal of Biology 2022;84:e256673. https://doi.org/10.1590/1519-6984.256673.
57. Liu S, Koslicki D. CMash: fast, multi-resolution estimation of k-mer-based Jaccard and containment indices. Bioinformatics 2022 06;38:i28–i35. https://doi.org/10.1093/bioinformatics/btac237.
58. Team MD, Mash Tutorials; 2023. Accessed: 2025-01-10. https://mash.readthedocs.io/en/latest/tutorials.html.
59. Irber L, Brooks PT, Reiter T, Pierce-Ward NT, Hera MR, Koslicki D, et al. Lightweight compositional analysis of metagenomes with FracMinHash and minimum metagenome covers. bioRxiv 2022;. https://doi.org/10.1101/2022.01.11.475838.
60. Kitts PA, Church DM, Thibaud-Nissen F, Choi J, Hem V, Sapojnikov V, et al. Assembly: a resource for assembled genomes at NCBI. Nucleic acids research 2016;44(D1):D73–D80. https://doi.org/10.1093/nar/gkv1226.
61. Schoch CL, Ciufo S, Domrachev M, Hotton CL, Kannan S, Khovanskaya R, et al. NCBI Taxonomy: a comprehensive update on curation, resources and tools. Database 2020;2020:baaa062. https://doi.org/10.1093/database/baaa062.
62. Li H. Minimap and miniasm: fast mapping and de novo assembly for noisy long sequences. Bioinformatics 2016;32(14):2103–2110. https://doi.org/10.1093/bioinformatics/btw152.
63. Dong J, Liu X, Sadasivan H, Sitaraman S, Narayanasamy S. mm2-gb: GPU accelerated minimap2 for long read dna mapping. In: Proceedings of the 15th ACM International Conference on Bioinformatics, Computational Biology and Health Informatics; 2024. p. 1–9. https://doi.org/10.1145/3698587.3701366.
64. Langmead B, Wilks C, Antonescu V, Charles R. Scaling read aligners to hundreds of threads on general-purpose processors. Bioinformatics 2019;35(3):421–432. https://doi.org/10.1093/bioinformatics/bty648.
65. Rosen G, Garbarine E, Caseiro D, Polikar R, Sokhansanj B. Metagenome Fragment Classification Using N-Mer Frequency Profiles. Advances in bioinformatics 2008;2008(1):205969. https://doi.org/10.1155/2008/205969.
66. Liu B, Gibbons T, Ghodsi M, Treangen T, Pop M. Accurate and fast estimation of taxonomic profiles from metagenomic shotgun sequences. Genome biology 2011;12:1–27. https://doi.org/10.1186/1471-2164-12-S2-S4.
67. Pruitt KD, Tatusova T, Maglott DR. NCBI reference sequences (RefSeq): a curated non-redundant sequence database of genomes, transcripts and proteins. Nucleic acids research 2007;35(suppl_1):D61–D65. https://doi.org/10.1093/nar/gkl842.
68. O'Leary NA, Wright MW, Brister JR, Ciufo S, Haddad D, McVeigh R, et al. Reference sequence (RefSeq) database at NCBI: current status, taxonomic expansion, and functional annotation. Nucleic acids research 2016;44(D1):D733–D745. https://doi.org/10.1093/nar/gkv1189.
69. Sayers EW, Beck J, Bolton EE, Bourexis D, Brister JR, Canese K, et al. Database resources of the national center for biotechnology information. Nucleic acids research 2021;49(D1):D10. https://doi.org/10.1093/nar/gkaa892.
70. Jesus TF, Ribeiro-Gonçalves B, Silva DN, Bortolaia V, Ramirez M, Carriço JA. Plasmid ATLAS: plasmid visual analytics and identification in high-throughput sequencing data. Nucleic acids research 2019;47(D1):D188–D194. https://doi.org/10.1093/nar/gky1073.
71. Blanco-Míguez A, Beghini F, Cumbo F, McIver LJ, Thompson KN, Zolfo M, et al. Extending and improving metagenomic taxonomic profiling with uncharacterized species using MetaPhlAn 4. Nature Biotechnology 2023;41(11):1633–1644. https://doi.org/10.1038/s41587-023-01688-w.
72. Burki F, Roger AJ, Brown MW, Simpson AG. The new tree of eukaryotes. Trends in ecology & evolution 2020;35(1):43–55. https://doi.org/10.1016/j.tree.2019.08.008.
73. Simmonds P, Adams MJ, Benkő M, Breitbart M, Brister JR, Carstens EB, et al. Virus taxonomy in the age of metagenomics. Nature Reviews Microbiology 2017;15(3):161–168. https://doi.org/10.1038/nrmicro.2016.177.
74. Harris HM, Hill C. A place for viruses on the tree of life. Frontiers in Microbiology 2021;11:604048. https://doi.org/10.3389/fmicb.2020.604048.
75. Martins IB, Silva JM, Almeida JR. Syst_Review: Systematic benchmark harness for metagenomic classification tools; 2024. GitHub. https://github.com/inesbmartins02/Syst_Review.
76. Silva JM, Martins IB, Almeida JR. HYMET Mash Sketch Databases (sketch1.msh, sketch2.msh, sketch3.msh); 2025. Zenodo. https://doi.org/10.5281/zenodo.17428354.
77. Silva JM, Martins IB, Almeida JR. HYMET Numerical Data underlying Figures 2–8 and Supplementary Figures S6–S10; 2025. Zenodo. https://doi.org/10.5281/zenodo.18772511.
78. CAMI Challenge. CAMI I Sample 0 Contig Assemblies; 2017. https://frl.publisso.de/data/frl:6421672/dataset/2017.12.29_11.37.26_sample_0_contigs.tar.
79. NCBI. NCBI RefSeq Genomes FTP; 2025. https://ftp.ncbi.nlm.nih.gov/genomes/refseq/.
80. NCBI. NCBI Assembly Database; 2025. https://www.ncbi.nlm.nih.gov/assembly/.
81. Genome Taxonomy Database. GTDB: Genome Taxonomy Database; 2025. https://gtdb.ecogenomic.org/.

82. Loman Lab. ZymoBIOMICS Mock Community Nanopore Assembly; 2019. http://nanopore.s3.climb.ac.uk/mockcommunity/v3/7cd60d3b-eafb-48d1-9aab-c8701232f2f8.ctg.cns.fa.
83. MGnify. MGnify Gut Metagenome Analysis MGYA00794604; 2025. https://www.ebi.ac.uk/metagenomics/api/v1/analyses/MGYA00794604/file/ERZ24911249_FASTA.fasta.gz.

Click here to access/download
Supplementary Material
main.log

Click here to access/download
Supplementary Material
main.out

Click here to access/download
Supplementary Material
main.aux

Click here to access/download
Supplementary Material
HYMET_Supplementary_Material.pdf

Click here to access/download
Supplementary Material
supplementary_main.tex

Manuscript Click here to access/download;Personal Cover;coverletter.pdf

Jorge Miguel Ferreira da Silva
IEETA/DETI, University of Aveiro
Aveiro, Portugal
jorge.miguel.ferreira.silva@ua.pt
+351 234 370 500

09/05/2025

Editor-in-Chief
GigaScience

Dear Editor,

I am pleased to submit our manuscript entitled **"HYMET: A Hybrid Metagenomic Pipeline for Accurate and Efficient Taxonomic Classification"** for consideration in *GigaScience*. Authored by Inês Martins, João Rafael Almeida, and myself, the work introduces HYMET, a lightweight pipeline that combines rapid k-mer screening with precise alignment to achieve robust taxonomic classification across all biological domains.

Metagenomic studies are often limited by the computational burden of large reference databases, the bias inherent in those resources, and the diminished accuracy of existing tools when confronted with highly mutated or fragmented sequences. HYMET overcomes these barriers through a two-stage workflow: first, *Mash Screen* rapidly filters candidate genomes, and then *Minimap2* refines the search with high-fidelity alignments. A dynamic thresholding algorithm tailors the reference set to each sample, and a weighted lowest-common-ancestor scheme delivers reliable lineage calls down to the species level. In benchmarks encompassing 26 203 genomes (14.76 GB) from Bacteria, Archaea, Eukarya, and viruses, HYMET achieved mean F1 scores above 0.9 and retained performance greater than 0.8 even at 30 % simulated mutation. Typical analyses complete in under two hours on standard workstations, with installation requiring only 2.82 GB and a transient reference cache of 10–50 GB—an efficiency advantage of one to two orders of magnitude over current state-of-the-art pipelines. All source code, sketched reference databases, test data, and analysis scripts are openly available under permissive licences, ensuring full reproducibility.

These contributions align directly with *GigaScience*'s commitment to disseminating reproducible, data-intensive research. The manuscript is original, not under review elsewhere, and all authors have approved its submission. No ethical approvals are required, as the study is entirely computational, and there are no competing interests to declare.

Thank you for considering our work. We believe this paper will be of significant interest to the journal's readership, providing both a methodological advance and a freely accessible resource for the metagenomics community. We look forward to your response and would be happy to supply any additional information you may require.

Sincerely,

Jorge Miguel Ferreira da Silva

Manuscript

Click here to access/download;Graphical Abstract;abstract.pdf

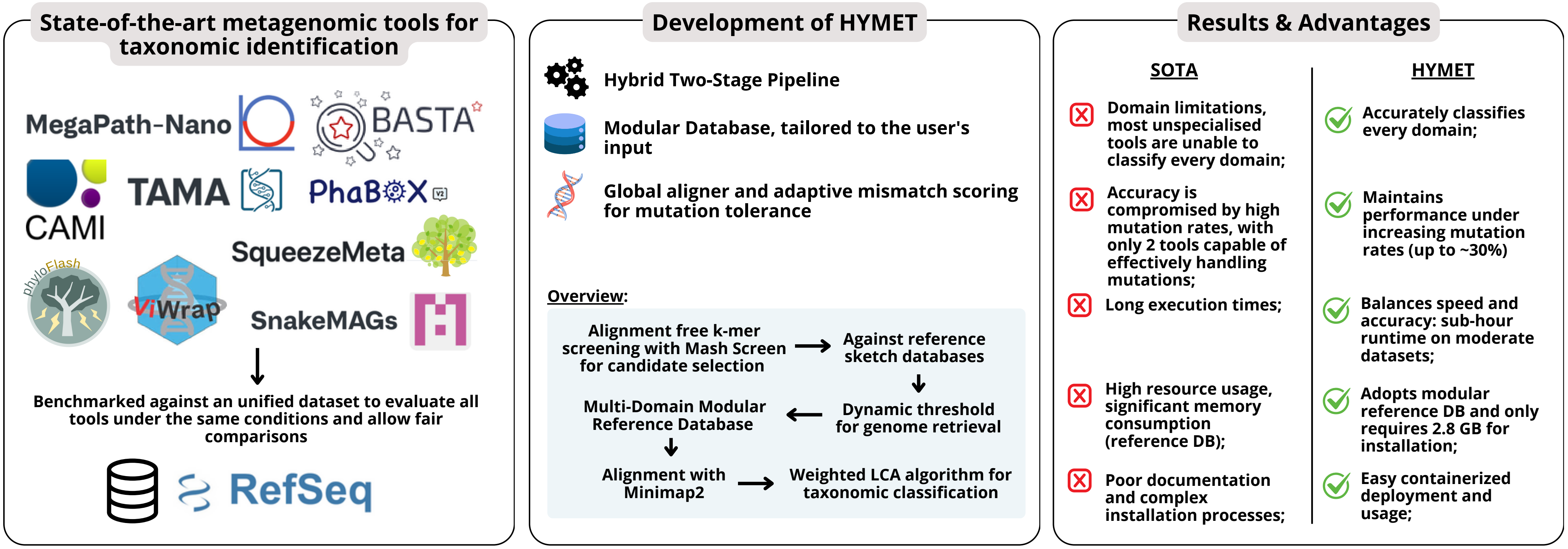

Supplement: giag024_GIGA-D-25-00184_Revision_2 [file giag024_giga-d-25-00184_revision_2.pdf]
