# Supplementary material for: HYMET: a hybrid metagenomic pipeline for accurate and efficient taxonomic classification
Source: Gigascience. 2026 Mar 2;15:giag024. doi: 10.1093/gigascience/giag024 (PMC13042306; doi:10.1093/gigascience/giag024)
Supplement: giag024_Authors_Response_To_Reviewer_Comments_original_submission [file giag024_authors_response_to_reviewer_comments_original_submission.pdf]

Author's Response To Reviewer Comments

HYMET (GIGA-D-25-00184R1) – Response to Reviewers
We appreciate the comments, which have contributed to improving the clarity, completeness, and robustness of our work. In the revised version, we have addressed the points raised, providing detailed responses, implementing manuscript edits, and, where applicable, enhancing the HYMET codebase to ensure consistency between the description and the tool's actual capabilities. We believe these revisions have substantially strengthened the manuscript, ensuring it meets the standards of GigaScience. We hope it is now suitable for publication.

Reviewer #1
The authors present a metagenomic classification workflow -- HYMET, which integrates k-mer screening (Mash) and alignment-based method (Minimap2) to reduce computational resources. Although it's not the first tool to employ this strategy in this field, HYMET offers some innovations, including a dynamic threshold method for candidate genome selection and a hybrid taxonomic assignment strategy.

Response:

We thank the reviewer for their constructive comments and careful evaluation of our work. We have carefully addressed the points raised in the review and revised the manuscript accordingly. We believe that these changes have strengthened the clarity and impact of the paper, and we hope that it is now suitable for publication.

Major:

Comment:
Metalign (10.1186/s13059-020-02159-0) adopts a highly similar strategy of pre-filtering with CMash and alignment with Minimap2. The manuscript does not mention this.

Response:

We agree. Metalign uses CMash pre-filtering followed by Minimap2. We now cite and discuss it. We also clarify what is new in HYMET, being the use of Mash Screen with a dynamic, sample-adaptive threshold, on-the-fly database construction across all domains, and weighted-LCA taxonomic assignment.
Comment:
- The chosen competitors (most are workflows) do not cover the widely used and state-of-the-art tools, such as KrakenUniq, Ganon, Sourmash gather, Taxor, Centrifuger, etc.

Response:
Agreed. We now cite KrakenUniq, Ganon/ganon2, Centrifuge and the recent Centrifuger, Taxor, and sourmash gather, and we state why they were not benchmarked directly. Our comparisons target end-to-end, cross-domain workflows; component-level short-read classifiers are represented via pipelines already in our set (e.g., CAMITAX uses Centrifuge/Kaiju; TAMA combines Kraken/CLARK/Centrifuge). Furthermore, we now compare many of these tools in a direct comparisson in CAMI section with HYMET, and added a brief scope note and references.
Comment:
- Is there a specific reason to use Mash for pre-filtering? Does it perform better than Sourmash, CMash, etc?

Response:

We agree that clarifying the choice of Mash is important. We chose Mash Screen primarily because it directly estimates containment between unassembled read sets and reference genomes, uses compact fixed-size sketches, and provides built-in statistical significance testing. These features integrate into HYMET's adaptive containment threshold approach, simplifying our strategy of selecting candidates per

query. Other similar tools such as sourmash typically employ scaled MinHash sketches and iterative set-cover methods, making them better suited for compositional decomposition tasks rather than directly prioritizing references by containment scores for downstream alignment. CMash (as used by Metalign) provides multi-resolution containment estimates across multiple k-values simultaneously. However, this capability was not required in HYMET, as we already handle separate sketch sets for different k-values and adjust containment thresholds dynamically per sample.

Comment:
- There's only one test dataset (test and validation dataset seems to be the same one), no real sequencing data. Moreover, the test dataset is simulated by introducing only point mutations, no structure variations. Widely used test datasets can be more convincing, such as those from the CAMI challenge.

Response:
We have expanded the evaluation to include the CAMI challenge subsets (seven samples across six tools) with full metrics. We also report real world case studies (MGnify gut contigs and the Zymo mock community) with runtime and taxonomic.
Comment:
- The program is not easy to use.
--- Though it's installable with conda, users still need to clone the code and run it within the directory.

Response:
We agree and have refactored HYMET to facilitate this issue. We unified Python CLI (bin/hymet); and it now allows directory-independent execution (e.g. hymet run/bench/case) and wraps batch processing, so cloning into a specific path is no longer required.
Comment:
--- The main entry is a Perl script named 'main.pl', and users need to manually type in the input directory, which prevents batch processing.

Response:

The maintained entry point is now the Python CLI (bin/hymet), which accepts explicit --contigs, --out, and --threads parameters and delegates to the same workflow wrappers used in the manuscript's experiments. main.pl is kept only for legacy compatibility and is flagged as such in the documentation.

Comment:
--- Though the docs say it accepts both read and contig as input, the tutorial says the input directory needs to contain .fna files.

Response:
Thank you for highlighting this ambiguity. The maintained entry point is the Python CLI, which supports both contig and read inputs. Contig runs accept a single multi FASTA (e.g., .fna/.fa/.fasta), while reads runs are supported via the harness and single sample CLI using Minimap2's sr preset. We updated the Supplementary to include an explicit reads example and to clarify the preset and other reproducibility knobs.

Minor:
Comment:
- Table 1: Why use different seed values for different databases? Did it affect the accuracy?

Response:
We thank the reviewer for pointing this out. The use of different seed values is due to how the reference sketches were obtained: the RefSeq88 sketch was downloaded directly from the Mash project, which provides its sketches built with seed 0, while we generated the GTDB and custom databases locally using Mash's default seed 42. Importantly, Mash Screen always hashes queries according to the seed embedded in each sketch, and each sketch file we use is internally consistent. Given the large sketch sizes (1000–5000 hashes), minor differences in seed selection have negligible impact on accuracy, and any minor stochastic variation is resolved during downstream alignment. Thus, the choice of different seeds across

databases does not meaningfully affect HYMET's performance.
To clarify this, we have added a brief note to the manuscript indicating the reason behind the seed differences.

Comment:
- Table 3: CPU usage is less informative, and exact memory occupation should be used, rather than percentage.

Response: The CAMI benchmark and both case studies (gut and Zymo) now report wall clock time and peak RSS (GB), providing clearer and more comparable measures of resource usage.
Comment:
- Downloading reference genomes might be slow and even problematic in some regions around the world, due to the connection speed and connectivity.

Response:
We agree. HYMET now minimizes network transfers and supports constrained connectivity. First, it screens locally against compact Mash sketches, so only a small set of candidate genomes is fetched. Second, every downloaded reference bundle is cached under a stable key and reused across runs, including the concatenated FASTA and the Minimap2 index. Third, the workflow supports mirrors and preloading: users can point candidate deduplication at a local mirror of the NCBI assembly summaries, and pre-seed a cache entry with a curated FASTA and a sequence-to-TaxID map using the provided preload helper, after which indices rebuild locally. The downloader itself uses parallel fetches with retries and exponential backoff and logs any failures. Together, these measures reduce transfer when a network is available and enable reproducible, fully offline runs once caches are populated.

Comment:
- The output should output TaxIDs for further analysis, which is more stable than lineages.

Response:
We agree, HYMET now emits TaxID. We highlighted this in the documentation and ensured the downstream converters keep the TaxID column alongside the lineage strings.

Reviewer #2

Martins et al. present HYMET, a novel method for fast and accurate taxonomic classification across all domains of life. Taxonomic classification remains a central challenge in metagenomics, and HYMET has the potential to contribute meaningfully. However, the current benchmarking is not sufficiently robust to support claims of superiority, and several critical issues should be addressed prior to publication.

Response:
We thank the reviewer for the constructive feedback. We have addressed the issues raised and revised the manuscript to improve clarity and robustness. We believe the paper has been strengthened and is now suitable for publication.
Comment:
* The rationale for sampling 10% of genome sequences to simulate fragmentation should be explained. Performance should be evaluated at varying genome fractions (e.g., 10%, 20%, 30%), reflecting more realistic diversity in metagenomic datasets. The authors should also consider benchmarking on simulated data derived from public metagenomic contigs to better emulate real-world conditions.

Response: We thank the reviewer for this suggestion. Our goal with the 10% choice was to emulate contig-level fragmentation and heterogeneity while keeping the build and evaluation pipeline reproducible and tractable. To that end, we subsample at two stages: (i) we select 10% of assemblies, then (ii) we select 10% of each assembly's sequences. We have documented the exact steps and scripts used (including the filtering stage) so the sampling can be reproduced precisely. Evaluations at additional fractions (e.g., 20% and 30%) are planned. In this revision we focus on the consolidated CAMI suite and two real-data case studies (MGnify gut contigs and the Zymo mock community), which together provide a stronger view of real-world performance than simulated data alone. We note that the case harness already

accepts public metagenomic contigs directly via a manifest, so future experiments that derive simulated sets from public contigs can be integrated without changes to the pipeline.
Comment:
* The manuscript does not clarify how mutation rates (0% to 30%) were introduced. If mutations were randomly generated, this should be stated explicitly. Simulations should ideally reflect evolutionary processes to better mirror biological variation.

Response:
We appreciate the point and now state this explicitly. For the mutation sweep, we used a deterministic simulator that applies independent per-base substitutions at the target rate (0–30%), with optional short (1–3 bp) insertions/deletions, all under a fixed random seed for reproducibility. This produces controlled divergence for robustness testing and is not intended to emulate evolutionary processes or tree-based substitution models. Our primary evidence relies on CAMI ground-truth datasets and real case studies, the mutation sweep complements these by stress-testing tolerance to divergence.
Comment:
* Commonly used methods like MetaPhlAn and Kraken should be included in comparative evaluations to contextualize HYMET's performance within the current landscape of tools.

Response:
We now addressed via the expanded CAMI benchmarking suite, which now includes MetaPhlAn4, kraken2, ganon2, centrifuge, sourmash gather, and HYMET with publicly available metrics.
Comment:
* For a fair comparison with other tools, a standardized database should be used wherever possible. For methods that support user-defined databases, such as Kraken, the authors should construct and apply the same reference database. A supplementary table summarizing the database composition for each benchmarked tool (e.g., number of genomes, species, domains) should be included to ensure transparency and facilitate reproducibility.

Response:
We now include a concise supplementary table summarizing each tool's database provenance, build date, and size, for tools that require their own official databases, we note deviations. HYMET's sketch checksums and Zenodo record (doi: 10.5281/zenodo.17428354) are also documented.

Comment:
* The manuscript references a single representative dataset (Table 2) for benchmarking. To demonstrate robustness, the authors should evaluate HYMET across multiple representative datasets with varied genome content.

Response: We now adress this issue by the expanded CAMI runs and the real-data case studies described in the revised Results section.

Comment:
* Benchmarking is currently based solely on the authors' own simulated data. This is insufficient to validate performance claims. Standard datasets such as those from the CAMI challenge should be incorporated.

Response: Thank you for the comment, we have adress this through the CAMI challenge evaluations, whose outputs and figures are now part of the results package.
Comment:
* A case study applying HYMET to a real-world metagenomic dataset would significantly strengthen the manuscript and demonstrate practical utility.

Response: Thanks you for the comment. We have now Added the MGnify gut and Zymo mock community case studies with visual summaries and runtime stats.
Comment:
* The impact of database incompleteness on HYMET's performance should be evaluated, as real-world databases rarely contain all possible genomes.

Response: Thank you for your comment. To adress this issue we have conducted curated-reference ablation experiments that quantify classification retention when key taxa are removed.

Comment:
* The manuscript's organization needs improvement. The Results section is too brief and lacks detail about the benchmarking experiments. The number of samples and replicates per parameter combination should be clearly described within the Results, not just in the Methods or Supplementary Materials. The "HYMET Workflow" section should be moved under Materials and Methods.

Response:
Thank you for the comment. We’ve reorganized and clarified as requested. The HYMET Workflow is now under Materials and Methods. The Results now include a concise Benchmark Scope and Replicates subsection that states: CAMI (7 assemblies), one run per sample per tool (no technical replicates), HYMET contigs vs reads on the same 7 assemblies, one run per mode, mutation sweep (0–30%) one run per rate per group, two case studies (MGnify gut, Zymo), one run each. We also reference the versioned manifest and harness in the Results for traceability.

Minor Comments

Comment:
* The abstract's opening sentence ("Metagenomics has revolutionized...") is generic and does not frame the specific problem of taxonomic classification. It should be revised or removed.

Response:
We revised the abstract’s opening to frame the specific problem of metagenomic taxonomic classification and HYMET’s contribution, removing generic phrasing.

Comment:
* The Introduction should include a brief rationale for why HYMET could outperform existing methods and summarize its core innovations. A note on the inspiration behind HYMET's design would also add clarity.

Response:
We tightened the Introduction to motivate HYMET’s design and summarize key innovations (adaptive Mash Screen, on the fly reference construction, weighted LCA resolver).
Comment:
* The manuscript is overly wordy. For example, the opening sentence of the Introduction does not add meaningful context and can be removed. The text should be streamlined to improve readability.

Response:
We streamlined the Introduction by removing generic lead ins and tightening wording.
Comment:
* The potential for HYMET to classify 16S rRNA sequencing data should be discussed.

Response:
HYMET targets assembled contigs and whole genomes; while 16S segments within contigs can be classified, targeted 16S amplicon profiling is better served by 16S specific tools (e.g., phyloFlash). We added a brief scope note to the manuscript explaining this.
Comment:
* As Figure 1 indicates that HYMET can accept both contigs and reads, the tool's performance should be evaluated separately for each input type.

Response:
We agree that input types should be assessed separately. The canonical benchmark in this revision is contig-based (CAMI panels and case studies), and we state this explicitly in the Results. In addition, we performed an internal HYMET-only comparison of the two input paths, contigs vs reads, using the harness read-mode (FASTQ staging, Mash screening, Minimap2 short-read alignment, weighted-LCA). Using synthetic reads derived from the CAMI assemblies to preserve ground truth, we observed near-identical

contig-level accuracies but differences in profile-level F1 at species, consistent with abundance estimation from read counts. This supports keeping cross-tool comparisons on contigs while providing a HYMET-vs-HYMET(reads) side-by-side view for transparency.

Reviewer #3
I would like to thank the authors for developing a well-structured and thoughtfully implemented tool, and for ensuring the work is fully reproducible through the inclusion of accessible datasets and code. While the manuscript is already strong and suitable for publication, I would like to offer a few suggestions that, in my opinion, could further enhance the clarity, robustness, and impact of the work.

Response:
We thank the reviewer for the positive assessment of our work and for recognizing the reproducibility and structure of the manuscript. We have carefully considered the suggestions provided and incorporated revisions to improve clarity. We believe the manuscript has benefited from these adjustments and hope is now suitable for publication.
Comment:
1. The validation dataset was chosen from curated sequences. The F1 scores for these are quite high for most of the tested groups. Could you provide an environmental or human microbiome sample to showcase real-world applicability with a broader application?

Response:
Addressed via the MGnify gut metagenome case study (plus the Zymo mock community), whose outputs and figures accompany the revised manuscript.
Comment:
2. Could you provide or propose strategies to improve classification at genus/species level, especially for high-mutation or underrepresented taxa?

Response:
At lower ranks, we recommend a practical set of steps to recover genus/species calls in difficult settings. First, soften the pre-filter so that more divergent candidates proceed to alignment; this helps when sequences carry many mutations. Second, augment the reference set with a small curated panel for the taxa of interest so that close representatives are available during alignment. Third, adjust the reporting thresholds of the weighted-LCA so that low-abundance but internally consistent evidence is retained, while still defaulting to genus-level reporting whenever species-level support is insufficient. Finally, use the ablation analysis to identify concrete gaps and focus curation where it measurably improves recall. Together, these measures improve sensitivity for high-mutation and underrepresented clades while maintaining precision through alignment confirmation and conservative calling.

Comment:
3. Genome retrieval failure is mentioned for some of the genomes. Could you propose an alternative strategy or a backup plan for genome downloads when RefSeq access fails, either automated retry policies or alternative sources.

Response:
Yes, we now adopted a more resilient retrieval policy. HYMET consults both RefSeq and GenBank catalogues and retrieves assemblies over HTTPS; transient errors are handled with bounded retries and exponential backoff, with failures recorded in the run logs. For constrained or offline environments, users can point to mirrored assembly summaries and pre-seed the reference cache with small curated panels so the pipeline proceeds without network access; indices are rebuilt on the next run. Persistent failures no longer block classification when sufficient references remain available. Information was added to the Supplementary Material.

Comment:
4. Is it possible to add a Singularity-Aptainer installation procedure? That would extend the use to HPC where the admin privileges are not necessary as in Docker.

Response:
Yes. We added a Singularity/Apptainer workflow for HPC use, the repository now ships a definition file (Singularity.def) mirroring the Docker image, and we document a simple build/exec procedure so HYMET can run without root privileges on clusters. The container exposes the same hymet CLI as the Bioconda and Docker installs, so inputs/outputs and caching behave identically.

Editorial Manager®

Cookie Preference Center

We use cookies which are necessary to make our site work. We may also use additional cookies to analyze, improve and personalize our content and your digital experience. For more information, see our Cookie Policy.

You may choose not to allow some types of cookies. However, blocking some types may impact your experience of our site and the services we are able to offer. See the different category headings below to find out more or change your settings.

Allow all

Manage Consent Preferences

Strictly Necessary Cookies

Always active

These cookies are necessary for the website to function and cannot be switched off in our systems. They are usually only set in response to actions made by you which amount to a request for services, such as setting your privacy preferences, logging in or filling in forms. You can set your browser to block or alert you about these cookies, but some parts of the site will not then work.

Functional Cookies

These cookies enable the website to provide enhanced functionality and personalisation. They may be set by us or by third party providers whose services we

have added to our pages. If you do not allow these cookies then some or all of these services may not function properly.

Performance Cookies

These cookies allow us to count visits and traffic sources so we can measure and improve the performance of our site. They help us to know which pages are the most and least popular and see how visitors move around the site.

Targeting Cookies

These cookies may be set through our site by our advertising partners. They may be used by those companies to build a profile of your interests and show you relevant adverts on other sites. If you do not allow these cookies, you will experience less targeted advertising.

Cookie List

Clear

Apply Cancel
Consent Leg.Interest

Confirm my choices

Powered by onetrust
