# Supplementary material for: HYMET: a hybrid metagenomic pipeline for accurate and efficient taxonomic classification
Source: Gigascience. 2026 Mar 2;15:giag024. doi: 10.1093/gigascience/giag024 (PMC13042306; doi:10.1093/gigascience/giag024)
Supplement: giag024_Authors_Response_To_Reviewer_Comments_revision_1 [file giag024_authors_response_to_reviewer_comments_revision_1.pdf]

Author's Response To Reviewer Comments

HYMET (GIGA-D-25-00184R1) – Response to Reviewers 2nd Review
We appreciate the comments, which improved the clarity, completeness, and robustness of our work. We addressed all points through detailed responses, manuscript edits, and enhancements to the HYMET codebase, aligning the description with the tool’s capabilities. We believe the manuscript is now ready for publishing.

Reviewer #1
I appreciate the effort the authors had taken on the software, results and manuscript. However, some issues remain.

Response:
We addressed all remaining concerns through manuscript revisions and software enhancements, as detailed below.

Major:
Comment:
- In the Background, the reason for not benchmarking Taxor and Ganon2 is not convincing, as they are not embedded within CAMITAX nor TAMA, and they were published recently (2024 and 2025). Taxor is designed for long-read classification that is similar to contig classification in HYMET. Ganon2 works mainly for short-read classification. While in the Result section, Ganon2 is compared.

Response:
We agree the previous wording was confusing. We revised the Background and Methods to distinguish benchmarked classifiers from those only discussed. Ganon2 is now included as a stand-alone baseline in the CAMI contig benchmark. Taxor is designed for read-level long-read classification and falls outside the contig-based evaluation scope, so we cite it but do not benchmark it directly.
Comment:
- The accuracy evaluation in the human gut assembly lacks the ground-truth. "gut microbiome profiles closely matched expected anaerobic bacterial taxa" is too arbitrary.
The sequencing data of ZymoBIOMICS Gut Microbiome Standard can be an alternative (https://github.com/Kirk3gaard/MicroBench?tab=readme-ov-file#zymogut).

Response:
We added the ZymoBIOMICS Gut Microbiome Standard (D6331) as a third case study with known ground truth. HYMET achieved near-perfect genus-level classification (Pearson r = 0.998, Bray-Curtis = 0.04) across 534 contigs spanning bacteria, fungi, and archaea. 11 of 14 expected genera were detected; the three missed genera each had ≤1 contig in the assembly. Species-level confusion between sister species (C. albicans/dubliniensis, S. cerevisiae/paradoxus) is a known limitation for organisms with >95% ANI. A new main-text figure and genus-level comparison table are in the revised manuscript.

Comment:
- It's still not easy to run a simple job with HYMET. After the installation with Conda following the docs (readme.md and reproducibility.md), it took me about **1 hour** to make a contig classification job run, with multiple trials even after checking docs and source code. It's highly recommended to ask a colleague to install it from scratch and do some tests, to make sure that the tool could work as expected.

```
$ hymet run --contig t.fasta --out . --threads 16
[hymet] (/home/xxx/app/miniconda3/envs/hymet/share/hymet) $
/home/xxx/app/miniconda3/envs/hymet/share/hymet/run_hymet_cami.sh
ERROR: missing data/detailed_taxonomy.tsv
```

```
# `detailed_taxonomy.tsv` seems to be generated by make_refset_taxonomy.py.

# resutls_summary.md provides a command to generate it
python bench/tools/make_refset_taxonomy.py \
--fasta bench/refsets/combined_subset.fasta \
--taxonkit-db taxonomy_files \
--output data/detailed_taxonomy.tsv

# But later I realised the file would be overwritten, and a file with only the header row would stop the error. So it's a bug.
```

Response:
The missing detailed_taxonomy.tsv was a bug: the pipeline expected the file but did not create it during initialisation. This is now fixed. A new hymet init subcommand handles first-run setup (NCBI taxonomy dumps, hierarchy file, required stubs) so that hymet run works without manual steps. The CLI also now prints clear error messages when prerequisites are missing. We updated the README and Supplementary Material to reflect the new workflow, and had colleagues unfamiliar with the tool test a fresh install end-to-end.

Minor:

Comment:
- Abstract and other places, "reference construction" should be "reference database construction" or "index building".

Response:
Fixed. We now use "reference database construction" instead of "reference construction" throughout.

Comment:
- Abstract and Result: "seven CAMI assemblies" are ambiguous (seven genomes?), it could be "CAMI assembly datasets".

Response:
Fixed. We now use “CAMI assembly datasets” throughout to avoid confusion with individual genome assemblies.
Comment:
- In Table 1 and the response to reviewers, it said RefSeq 88 is used in sketch1.msh (directly downloaded from the Mash project). While in the section "Reference Sketched Database", it's RefSeq Nucleotide Release 228.

Response:
Good catch. The “Release 228” mention referred to the pool for dynamic alignment updates, not the static screening baseline. We revised the Methods to make this explicit: HYMET screens against RefSeq Release 88 sketches, supplemented by custom databases (sketch3.msh) for modern diversity. The text now reads “RefSeq Release 88 (Base Screen) + Custom Augmentation”.
Comment:
- In the context of sketching databases, the term "seed" can be rephrased as "hash seed" to avoid confusion with the "seed sequence" used in alignment tools such as Blast and Minimap2.

Response:
We agree. We replaced "seed" with "hash seed" in the manuscript text, Table 1 caption, and column header.

Comment:

- There are too many figures and tables in the main text, and most of the text is too small to read.

Response:
We agree. We relocated six figures to the Supplementary Material (now Figs S4–S9) and merged related panels into composites (Figs 6 and 7), bringing the main-text count from 15 to 8. The extra figure (Fig. 8) covers the ZymoGut D6331 ground-truth evaluation. All remaining figures use full-width layouts.

Comment:
- Supplementary Figure 2 and 3: The texts in the figures are hard to read.

Response:
We increased the resolution and display width of Supplementary Figures S1–S3 to full page width. Captions now list the tools and domains shown in each panel.
Comment:
- Each run creates a new cache directory, such as data/downloaded_genomes/cache/51e459af820785d66646fcaab7c6da69688975da, and assembly summary files of GenBank (1.4 GB) and refseq (200 MB) will be repeatedly downloaded to download_cache/. It's unnecessary and would cost a lot of time for a slow internet connection. Even after setting `--cache-root`, it still creates a new cache directory with a new cache key and fails to use the existing cache.

Response:
The root cause was that the cache key included transient path components, so a new cache directory was created every run even with the same candidate set. HYMET now computes a stable cache key from the sorted accession list, so repeated runs reuse the existing cache. The --cache-root flag is properly propagated to all stages. Assembly summary files are also cached locally and only refreshed with --force-download, avoiding the repeated GenBank (1.4 GB) and RefSeq (200 MB) downloads. Documented in the Supplementary Material.
Comment:

- In the abstract and discussion, it is said in the benchmark, HYMET's peak memory usage is ~6.2GB. However, in the benchmark, there are only 922 reference genomes are used. In my test with a contig (with the 3 default skeches databases), the prefiltering step with Mash would consume ~13 GB.

Response:
The ~6.2 GB figure is the mean across the seven CAMI samples. Peak memory is sample-dependent: five low-complexity panels run under 2.3 GB, while CAMI II marine and strain madness reach ~17.4 GB due to larger candidate sets. The reviewer’s ~13 GB observation with all three default sketches is consistent with this. We revised the abstract, key points, and discussion to report 6.2 GB explicitly as a mean and note the sample-dependent range (under 2 GB to ~17 GB).

Reviewer #2

Most of the concerns have been addressed. However, a few minor issues remain:

Response:
We addressed the remaining points as detailed below.
Comment:
There are too many figures (15 in total). It would be better to merge related figures into composite panels to improve readability.

Response:
We agree. We merged related figures into composite panels and reduced the main-text count from 15 to 8 (the additional figure addresses the ZymoGut ground-truth evaluation).

Comment:
The evaluation metrics for each independent benchmark dataset should be clearly presented.

Response:
We now provide per-dataset metrics in Supplementary Table S18, reporting Avg F1, Genus F1, and Species F1 for each of the seven CAMI samples individually. The full per-sample, per-rank breakdown is also versioned in the project repository (results/cami/canonical/RUN_0/tables/).

Comment:
The Results section is still somewhat confusing, despite the good performance. Consider reorganizing it so that each subsection clearly conveys a specific conclusion.

Response:
We reorganized the Results. The CAMI benchmark now leads with classification accuracy and profile quality, followed by Computational Efficiency, Mutation Resilience, input-modality comparison, and real-data case study subsections.

Comment:
Figure 2 is not referenced in the text and should be properly cited.

Response:
Thank you for catching this. Figure 2 (F1 scores across taxonomic groups at 0% mutation) is now explicitly cited in the CAMI Benchmark subsection of the Results.

Editorial Manager®

Cookie Preference Center

We use cookies which are necessary to make our site work. We may also use additional cookies to analyze, improve and personalize our content and your digital experience. For more information, see our Cookie Policy.

You may choose not to allow some types of cookies. However, blocking some types may impact your experience of our site and the services we are able to offer. See the different category headings below to find out more or change your settings.

Allow all

Manage Consent Preferences

Strictly Necessary Cookies

Always active

These cookies are necessary for the website to function and cannot be switched off in our systems. They are usually only set in response to actions made by you which amount to a request for services, such as setting your privacy preferences, logging in or filling in forms. You can set your browser to block or alert you about these cookies, but some parts of the site will not then work.

Functional Cookies

These cookies enable the website to provide enhanced functionality and personalisation. They may be set by us or by third party providers whose services we have added to our pages. If you do not allow these cookies then some or all of these services may not function properly.

Performance Cookies

These cookies allow us to count visits and traffic sources so we can measure and improve the performance of our site. They help us to know which pages are the most and least popular and see how visitors move around the site.

Targeting Cookies

These cookies may be set through our site by our advertising partners. They may be used by those companies to build a profile of your interests and show you relevant adverts on other sites. If you do not allow these cookies, you will experience less targeted advertising.

Cookie List

Clear

Apply Cancel

Consent Leg.Interest

Confirm my choices

Powered by onetrus
