# Supplementary material for: HYMET: a hybrid metagenomic pipeline for accurate and efficient taxonomic classification
Source: Gigascience. 2026 Mar 2;15:giag024. doi: 10.1093/gigascience/giag024 (PMC13042306; doi:10.1093/gigascience/giag024)
Supplement: giag024_Reviewer_1_Report_original_submission — Reviewer 1 -- 6/8/2025 [file giag024_reviewer_1_report_original_submission.pdf]

Reviewer Report

Title: HYMET: A Hybrid Metagenomic Pipeline for Accurate and Efficient Taxonomic Classification

Version: Original Submission **Date:** 6/8/2025

Reviewer name: Reviewer 1

Reviewer Comments to Author:

The authors present a metagenomic classification workflow -- HYMET, which integrates k-mer screening (Mash) and alignment-based method (Minimap2) to reduce computational resources. Although it's not the first tool to employ this strategy in this field, HYMET offers some innovations, including a dynamic threshold method for candidate genome selection and a hybrid taxonomic assignment strategy.Major:- Metalign (10.1186/s13059-020-02159-0) adopts a highly similar strategy of pre-filtering with CMash and alignment with Minimap2. The manuscript does not mention this.- The chosen competitors (most are workflows) do not cover the widely used and state-of-the-art tools, such as KrakenUniq, Ganon, Sourmash gather, Taxor, Centrifuger, etc.- Is there a specific reason to use Mash for pre-filtering? Does it perform better than Sourmash, CMash, etc?- There's only one test dataset (test and validation dataset seems to be the same one), no real sequencing data. Moreover, the test dataset is simulated by introducing only point mutations, no structure variations. Widely used test datasets can be more convincing, such as those from the CAMI challenge.- The program is not easy to use.--- Though it's installable with conda, users still need to clone the code and run it within the directory.--- The main entry is a Perl script named 'main.pl', and users need to manually type in the input directory, which prevents batch processing.--- Though the docs say it accepts both read and contig as input, the tutorial says the input directory needs to contain .fna files.Minor:- Table 1: Why use different seed values for different databases? Did it affect the accuracy?- Please use more distinct colours in Figures 2 and 3.- Table 3: CPU usage is less informative, and exact memory occupation should be used, rather than percentage.- Downloading reference genomes might be slow and even problematic in some regions around the world, due to the connection speed and connectivity.- The output should output TaxIDs for further analysis, which is more stable than lineages.

Level of Interest

Please indicate how interesting you found the manuscript: Choose an item.

Quality of Written English

Please indicate the quality of language in the manuscript: Choose an item.

Declaration of Competing Interests

Please complete a declaration of competing interests, considering the following questions:

- Have you in the past five years received reimbursements, fees, funding, or salary from an organisation that may in any way gain or lose financially from the publication of this manuscript, either now or in the future?
- Do you hold any stocks or shares in an organisation that may in any way gain or lose financially from the publication of this manuscript, either now or in the future?
- Do you hold or are you currently applying for any patents relating to the content of the manuscript?
- Have you received reimbursements, fees, funding, or salary from an organization that holds or has applied for patents relating to the content of the manuscript?
- Do you have any other financial competing interests?
- Do you have any non-financial competing interests in relation to this paper?

If you can answer no to all of the above, write 'I declare that I have no competing interests' below. If your reply is yes to any, please give details below.

I declare that I have no competing interests.

I agree to the open peer review policy of the journal. I understand that my name will be included on my report to the authors and, if the manuscript is accepted for publication, my named report including any attachments I upload will be posted on the website along with the authors' responses. I agree for my report to be made available under an Open Access Creative Commons CC-BY license (http://creativecommons.org/licenses/by/4.0/). I understand that any comments which I do not wish to be included in my named report can be included as confidential comments to the editors, which will not be published.

Choose an item.

To further support our reviewers, we have joined with Publons, where you can gain additional credit to further highlight your hard work (see: https://publons.com/journal/530/gigascience). On publication of this paper, your review will be automatically added to Publons, you can then choose whether or not to claim your Publons credit. I understand this statement.

Yes Choose an item.
