# Supplementary material for: HYMET: a hybrid metagenomic pipeline for accurate and efficient taxonomic classification
Source: Gigascience. 2026 Mar 2;15:giag024. doi: 10.1093/gigascience/giag024 (PMC13042306; doi:10.1093/gigascience/giag024)
Supplement: giag024_Reviewer_1_Report_revision_1 — Reviewer 1 -- 11/12/2025 [file giag024_reviewer_1_report_revision_1.pdf]

Reviewer Report

Title: HYMET: A Hybrid Metagenomic Pipeline for Accurate and Efficient Taxonomic Classification

Version: Revision 1 **Date:** 11/12/2025

Reviewer name: Reviewer 1

Reviewer Comments to Author:

I appreciate the effort the authors had taken on the software, results and manuscript. However, some issues remain.Major- In the Background, the reason for not benchmarking Taxor and Ganon2 is not convincing, as they are not embedded within CAMITAX nor TAMA, and they were published recently (2024 and 2025). Taxor is designed for long-read classification that is similar to contig classification in HYMET. Ganon2 works mainly for short-read classification. While in the Result section, Ganon2 is compared.- The accuracy evaluation in the human gut assembly lacks the ground-truth. "gut microbiome profiles closely matched expected anaerobic bacterial taxa" is too arbitrary.The sequencing data of ZymoBIOMICS Gut Microbiome Standard can be an alternative (https://github.com/Kirk3gaard/MicroBench?tab=readme-ov-file#zymogut).- It's still not easy to run a simple job with HYMET. After the installation with Conda following the docs (readme.md and reproducibility.md), it took me about **1 hour** to make a contig classification job run, with multiple trials even after checking docs and source code. It's highly recommended to ask a colleague to install it from scratch and do some tests, to make sure that the tool could work as expected.```$ hymet run --contig t.fasta --out . --threads 16[hymet] (/home/xxx/app/miniconda3/envs/hymet/share/hymet) $ /home/xxx/app/miniconda3/envs/hymet/share/hymet/run_hymet_cami.shERROR: missing data/detailed_taxonomy.tsv# `detailed_taxonomy.tsv` seems to be generated by make_refset_taxonomy.py.# resutls_summary.md provides a command to generate itpython bench/tools/make_refset_taxonomy.py \--fasta bench/refsets/combined_subset.fasta \--taxonkit-db taxonomy_files \--output data/detailed_taxonomy.tsv# But later I realised the file would be overwritten, and a file with only the header row would stop the error. So it's a bug.```Minor:- Abstract and other places, "reference construction" should be "reference database construction" or "index building".- Abstract and Result: "seven CAMI assemblies" are ambiguous (seven genomes?), it could be "CAMI assembly datasets".- In Table 1 and the response to reviewers, it said RefSeq 88 is used in sketch1.msh (directly downloaded from the Mash project). While in the section "Reference Sketched Database", it's RefSeq Nucleotide Release 228.- In the context of sketching databases, the term "seed" can be rephrased as "hash seed" to avoid confusion with the "seed sequence" used in alignment tools such as Blast and Minimap2.- There are too many figures and tables in the main text, and most of the text is too small to read.- Supplementary Figure 2 and 3: The texts in the figures are hard to read.- Each run creates a new cache directory, such as data/downloaded_genomes/cache/51e459af820785d66646fcaab7c6da69688975da, and assembly summary files of GenBank (1.4 GB) and refseq (200 MB) will be repeatedly downloaded to download_cache/. It's unnecessary and would cost a lot of time for a slow internet connection. Even after setting `--cache-root`, it still creates a new cache directory with a new cache key and fails to use

the existing cache.- In the abstract and discussion, it is said in the benchmark, HYMET's peak memory usage is ~6.2GB. However, in the benchmark, there are only 922 reference genomes are used. In my test with a contig (with the 3 default skeches databases), the prefiltering step with Mash would consume ~13 GB.

Level of Interest

Please indicate how interesting you found the manuscript: Choose an item.

Quality of Written English

Please indicate the quality of language in the manuscript: Choose an item.

Declaration of Competing Interests

Please complete a declaration of competing interests, considering the following questions:

- Have you in the past five years received reimbursements, fees, funding, or salary from an organisation that may in any way gain or lose financially from the publication of this manuscript, either now or in the future?
- Do you hold any stocks or shares in an organisation that may in any way gain or lose financially from the publication of this manuscript, either now or in the future?
- Do you hold or are you currently applying for any patents relating to the content of the manuscript?
- Have you received reimbursements, fees, funding, or salary from an organization that holds or has applied for patents relating to the content of the manuscript?
- Do you have any other financial competing interests?
- Do you have any non-financial competing interests in relation to this paper?

If you can answer no to all of the above, write 'I declare that I have no competing interests' below. If your reply is yes to any, please give details below.

I declare that I have no competing interests.

I agree to the open peer review policy of the journal. I understand that my name will be included on my report to the authors and, if the manuscript is accepted for publication, my named report including any attachments I upload will be posted on the website along with the authors' responses. I agree for my report to be made available under an Open Access Creative Commons CC-BY license (http://creativecommons.org/licenses/by/4.0/). I understand that any comments which I do not wish to be included in my named report can be included as confidential comments to the editors, which will not be published.

Choose an item.

To further support our reviewers, we have joined with Publons, where you can gain additional credit to further highlight your hard work (see: https://publons.com/journal/530/gigascience). On publication of this paper, your review will be automatically added to Publons, you can then choose whether or not to claim your Publons credit. I understand this statement.

Yes Choose an item.
