# Supplementary material for: HYMET: a hybrid metagenomic pipeline for accurate and efficient taxonomic classification
Source: Gigascience. 2026 Mar 2;15:giag024. doi: 10.1093/gigascience/giag024 (PMC13042306; doi:10.1093/gigascience/giag024)
Supplement: giag024_Reviewer_2_Report_original_submission — Reviewer 2 -- 6/16/2025 [file giag024_reviewer_2_report_original_submission.pdf]

Reviewer Report

Title: HYMET: A Hybrid Metagenomic Pipeline for Accurate and Efficient Taxonomic Classification

Version: Original Submission **Date:** 6/16/2025

Reviewer name: Reviewer 2

Reviewer Comments to Author:

Martins et al. present HYMET, a novel method for fast and accurate taxonomic classification across all domains of life. Taxonomic classification remains a central challenge in metagenomics, and HYMET has the potential to contribute meaningfully. However, the current benchmarking is not sufficiently robust to support claims of superiority, and several critical issues should be addressed prior to publication.* The rationale for sampling 10% of genome sequences to simulate fragmentation should be explained. Performance should be evaluated at varying genome fractions (e.g., 10%, 20%, 30%), reflecting more realistic diversity in metagenomic datasets. The authors should also consider benchmarking on simulated data derived from public metagenomic contigs to better emulate real-world conditions.* The manuscript does not clarify how mutation rates (0% to 30%) were introduced. If mutations were randomly generated, this should be stated explicitly. Simulations should ideally reflect evolutionary processes to better mirror biological variation.* Commonly used methods like MetaPhlAn and Kraken should be included in comparative evaluations to contextualize HYMET's performance within the current landscape of tools.* For a fair comparison with other tools, a standardized database should be used wherever possible. For methods that support user-defined databasesâ€"such as Krakenâ€"the authors should construct and apply the same reference database. A supplementary table summarizing the database composition for each benchmarked tool (e.g., number of genomes, species, domains) should be included to ensure transparency and facilitate reproducibility.* The manuscript references a single representative dataset (Table 2) for benchmarking. To demonstrate robustness, the authors should evaluate HYMET across multiple representative datasets with varied genome content.* Benchmarking is currently based solely on the authors' own simulated data. This is insufficient to validate performance claims. Standard datasets such as those from the CAMI challenge should be incorporated.* A case study applying HYMET to a real-world metagenomic dataset would significantly strengthen the manuscript and demonstrate practical utility.* The impact of database incompleteness on HYMET's performance should be evaluated, as real-world databases rarely contain all possible genomes.* The manuscript's organization needs improvement. The Results section is too brief and lacks detail about the benchmarking experiments. The number of samples and replicates per parameter combination should be clearly described within the Resultsâ€"not just in the Methods or Supplementary Materials. The "HYMET Workflow" section should be moved under Materials and Methods.Minor Comments* The abstract's opening sentence ("Metagenomics has revolutionized...") is generic and does not frame the specific problem of taxonomic classification. It should be revised or removed.* The Introduction should include a brief rationale for why HYMET could outperform existing methods and summarize its core innovations. A note on the inspiration behind HYMET's design would also add clarity.* The manuscript is overly wordy. For example, the opening sentence of the Introduction does not add meaningful context

and can be removed. The text should be streamlined to improve readability.* The potential for HYMET to classify 16S rRNA sequencing data should be discussed.* As Figure 1 indicates that HYMET can accept both contigs and reads, the tool's performance should be evaluated separately for each input type.

Level of Interest

Please indicate how interesting you found the manuscript: Choose an item.

Quality of Written English

Please indicate the quality of language in the manuscript: Choose an item.

Declaration of Competing Interests

Please complete a declaration of competing interests, considering the following questions:

- Have you in the past five years received reimbursements, fees, funding, or salary from an organisation that may in any way gain or lose financially from the publication of this manuscript, either now or in the future?
- Do you hold any stocks or shares in an organisation that may in any way gain or lose financially from the publication of this manuscript, either now or in the future?
- Do you hold or are you currently applying for any patents relating to the content of the manuscript?
- Have you received reimbursements, fees, funding, or salary from an organization that holds or has applied for patents relating to the content of the manuscript?
- Do you have any other financial competing interests?
- Do you have any non-financial competing interests in relation to this paper?

If you can answer no to all of the above, write 'I declare that I have no competing interests' below. If your reply is yes to any, please give details below.

I declare that I have no competing interests

I agree to the open peer review policy of the journal. I understand that my name will be included on my report to the authors and, if the manuscript is accepted for publication, my named report including any attachments I upload will be posted on the website along with the authors' responses. I agree for my report to be made available under an Open Access Creative Commons CC-BY license (http://creativecommons.org/licenses/by/4.0/). I understand that any comments which I do not wish to be included in my named report can be included as confidential comments to the editors, which will not be published.

Choose an item.

To further support our reviewers, we have joined with Publons, where you can gain additional credit to further highlight your hard work (see: https://publons.com/journal/530/gigascience). On publication of this paper, your review will be automatically added to Publons, you can then choose whether or not to claim your Publons credit. I understand this statement.

Yes Choose an item.
